# Synthesis of Novel Triazine-Based Chalcones and 8,9-dihydro-7*H*-pyrimido[4,5-*b*][1,4]diazepines as Potential Leads in the Search of Anticancer, Antibacterial and Antifungal Agents

**DOI:** 10.3390/ijms25073623

**Published:** 2024-03-23

**Authors:** Leydi M. Moreno, Jairo Quiroga, Rodrigo Abonia, María del P. Crespo, Carlos Aranaga, Luis Martínez-Martínez, Maximiliano Sortino, Mauricio Barreto, María E. Burbano, Braulio Insuasty

**Affiliations:** 1 Grupo de Investigación de Compuestos Heterocíclicos, Departamento de Química, Universidad del Valle, Cali 760042, Colombia; jairo.quiroga@correounivalle.edu.co (J.Q.); rodrigo.abonia@correounivalle.edu.co (R.A.); 2 Grupo de Biotecnología e Infecciones Bacterianas, Departamento de Microbiología, Universidad del Valle, Cali 760042, Colombia; maria.crespo.ortiz@correounivalle.edu.co; 3 Grupo de Microbiología y Enfermedades Infecciosas, Departamento de Microbiología, Universidad del Valle, Cali 760042, Colombia; mauricio.barreto@correounivalle.edu.co (M.B.); maria.e.burbano@correounivalle.edu.co (M.E.B.); 4 Grupo de Investigación en Química y Biotecnología (QUIBIO), Facultad de Ciencias Básicas, Universidad Santiago de Cali, Cali 760035, Colombia; carlos.aranaga00@usc.edu.co; 5 Grupo de Investigación Traslacional en Enfermedades Infecciosas, Escuela de Biomedicina, Universidad de Córdoba, 14014 Córdoba, Spain; 6 Unidad de Microbiología Clínica, Hospital Universitario Reina Sofía, Instituto Maimónides de Investigación Biomédica de Córdoba (IMIBIC), Departamento de Química Agrícola, Edafología y Microbiología, Universidad de Córdoba, 14004 Córdoba, Spain; luis.martinez.martinez.sspa@juntadeandalucia.es; 7 Área de Farmacognosia, Facultad de Ciencias Bioquímicas y Farmacéuticas, Universidad Nacional de Rosario, Suipacha 531, Rosario 2000, Argentina; msortino@fbioyf.unr.edu.ar

**Keywords:** 1,3,5-triazines, chalcones, diazepines, anticancer activity, antibacterial activity, antifungal activity, cytotoxicity

## Abstract

This study presents the synthesis of four series of novel hybrid chalcones (**20,21**)**a**–**g** and (**23,24**)**a**–**g** and six series of 1,3,5-triazine-based pyrimido[4,5-*b*][1,4]diazepines (**28**–**33**)**a**–**g** and the evaluation of their anticancer, antibacterial, antifungal, and cytotoxic properties. Chalcones **20b**,**d**, **21a,b**,**d**, **23a**,**d**–**g**, **24a**–**g** and the pyrimido[4,5-*b*][1,4]diazepines **29e**,**g**, **30g**, **31a**,**b**,**e**–**g**, **33a**,**b**,**e**–**g** exhibited outstanding anticancer activity against a panel of 60 cancer cell lines with GI_50_ values between 0.01 and 100 μM and LC_50_ values in the range of 4.09 μM to >100 μM, several of such derivatives showing higher activity than the standard drug 5-fluorouracil (5-FU). On the other hand, among the synthesized compounds, the best antibacterial properties against *N. gonorrhoeae*, *S. aureus* (ATCC 43300), and *M. tuberculosis* were exhibited by the pyrimido[4,5-*b*][1,4]diazepines (MICs: 0.25–62.5 µg/mL). The antifungal activity studies showed that triazinylamino-chalcone **29e** and triazinyloxy-chalcone **31g** were the most active compounds against *T. rubrum* and *T. mentagrophytes* and *A. fumigatus*, respectively (MICs = 62.5 μg/mL). Hemolytic activity studies and in silico toxicity analysis demonstrated that most of the compounds are safe.

## 1. Introduction

Cancer and infectious diseases caused by the drug resistance of bacteria and fungi are one of the main causes of death worldwide, and this requires highly selective and efficient treatments with low toxicity. Around 10 million people died from cancer in 2020 worldwide [1,2], and according to Pan American Health Organization (PAHO) it is estimated that this value would increase to 57% by 2040 [3]. Likewise, infections by resistant bacteria cause around 700,000 deaths annually worldwide, of which 230,000 deaths are due to multi-resistant tuberculosis [4].

The synthesis of new compounds based on low molecular weight nitrogen-heterocyclic fragments remains a successful strategy and one of significant interest in the discovery of new therapeutic agents. These fragments are present in a large number of drugs and bioactive molecules, which can establish different types of chemical interactions (hydrogen bonds, π-stacking interactions, among others) with biological systems [5,6,7,8,9]. Thus, 1,3,5-triazine-, pyrimidine-, and diazepine heterocyclic moieties are present in diverse molecules exhibiting multiple biological properties acting as antioxidants [10,11], anti-HIV [12,13,14], anticonvulsants [15,16], antimicrobials [17,18,19], anticancer [20,21,22,23], among others [24,25].

**Figure 1:**
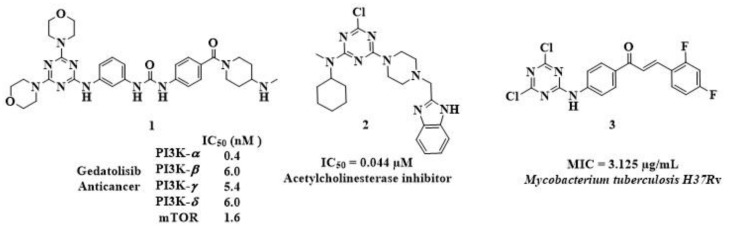
1,3,5-Triazine hybrids with biological properties.

Particularly, 1,3,5-triazine is a heterocyclic molecule of wide synthetic versatility, which has the possibility of functionalizing in positions 2, 4, and 6, allowing it to easily modulate the physicochemical and biological activity of their derivatives [26]. The fusion of triazine with other heterocyclic moieties and α,β-unsaturated ketones (molecular hybrids) has generated derivatives with valuable biological properties [7,9,27,28,29,30,31,32,33,34] (Figure 1), including the anticancer drug gedatolisib [35,36,37,38] used in the treatment of breast cancer. Its mechanism of action is based on the inhibition of kinases PI3K and mTOR, thus promoting cell apoptosis [39]. Another example is the triazine derivative **2,** which is a potential multi-target agent for the treatment of Alzheimer’s disease; this compound exhibited a IC_50_ of 0.044 µM against AChE, which is better than donepezil (IC_50_ = 0.052 µM). Triazine-chalcone hybrid **3** demonstrated potential antitubercular activity (anti-TB) against *Mycobacterium tuberculosis* H37Rv. 

α,β-unsaturated carbonyl compounds, also known as chalcones, are important structural scaffolds for natural medicine. They are widely distributed in nature (i.e., fruits, vegetables, and spices) and are precursors for the biosynthesis of flavonoids and isoflavonoids in plants [40,41]. Chalcones have generated significant interest due to their biological properties, such as anticancer [32,42], antibacterial [34,43], antifungal [44], antimalarial [45], anti-inflammatory [46], and neuroprotective [47] activities. Several chalcone-based drugs have been approved for clinical use, including methochalcone (choleretic) [48] and sofalcone (antiulcer) [49]. In previous studies, we reported the synthesis of triazine-based chalcones with outstanding anticancer properties comparable to the drug 5-fluorouracil (thymidylate synthase (TS) inhibitor) [50,51]. In silico studies determined that the anticancer activity exhibited by these compounds could be related to the inhibition of the enzyme TS [52].

Diazepine rings fused to a benzene rings (benzodiazepines) or heteroaromatic rings have shown not only anxiolytic properties, which they are especially known for, but also anticancer [20], antioxidant [53], antimicrobial [54], and anti-inflammatory [55] properties. Particularly, pyrimido-diazepine scaffolds have been proven successful as antimicrobial and anticancer agents [54,56,57,58,59,60,61]. Their anticancer action mechanism involves the inhibition of Aurora A, Aurora B, and Kinase Insert Domain-containing Receptor (KDR) [60], receptor tyrosine kinases such as Flt3, and c-Kit [62], extracellular-signal-regulated kinase 5 (ERK5), and leucine rich repeat kinase 2 (LRRK2) [63].

The union of pharmacophoric fragments to generate molecular hybrids has been an attractive and useful strategy in medicinal chemistry to generate lead molecules with potential biological properties [64,65,66,67]. Stimulated by valuable bioactive properties of 1,3,5-triazine, chalcone, and diazepine derivatives, and based on molecular hybridization approach, in this study, we are reporting the synthesis of 1,3,5-triazine-based chalcone- and diazepine hybrids through a simple and versatile synthetic pathway. In vitro screening tests were used for determining the anticancer, antibacterial, antifungal, and cytotoxic profiles of the novel compounds synthesized, which showed outstanding results.

## 2. Results and Discussion

### 2.1. Chemistry

Initially, using a three-step synthetic sequence, the trisubstituted triazine precursors **12**–**15** were synthesized by aromatic nucleophilic substitution reactions (Ar_N_S) from 2,4,6-trichloro-1,3,5-triazine **4** [50,68,69,70,71,72,73,74,75] (Table 1). Looking for structural diversity, substituents of aliphatic and aromatic nature and functional groups capable of forming hydrogen bonding were incorporated. Various reaction parameters were explored for each compound and Table 1 shows the optimized reaction conditions that allowed these precursors to be obtained in high yields. To assure the monosubstitution of a chlorine atom to prepare the precursors **5**–**7**, it was imperative to perform the reactions at low temperature (−5–0 °C). The second substitutions were carried out at room temperature, except for **11**, since the trisubstituted product was favored; at low temperature it was obtained as the only product. Sodium carbonate (20%) was used as a hydrogen chloride acceptor. As the reactions progressed, the medium acidified to a point where they no longer progressed, therefore, the addition of the base was done slowly throughout the reaction and always maintaining a neutral pH. The trisubstituted derivatives were synthesized under heating at reflux (for **13**) and at room temperature (for **12**, **14**, and **15**). Initially, for the synthesis of triazines **12** and **13**, dioxane and DMF were tested as solvents. In both tests, an excess of ethylenediamine (1.5 equiv.) and stirring at room temperature was used; however, under these reaction conditions complex mixtures of products were obtained. The use of ethylenediamine as a reaction medium allowed the obtaining of compounds **12** and **13** with good yields.

The structures of precursors were confirmed by FTIR, ^1^H, and ^13^C NMR and mass spectra data (Supplementary Material).

**Scheme 1:**
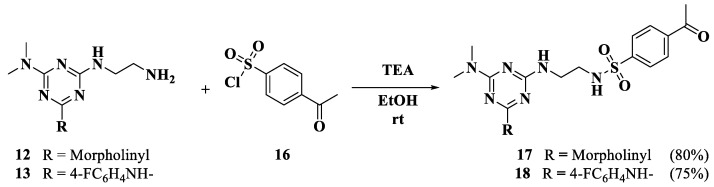
Synthesis of triazine-based sulfonamides **17**,**18**.

Subsequently, trisubstituted precursors **12** and **13** were reacted with 4-acetylbenzenesulfonyl chloride **16** under stirring at room temperature in ethanol and using TEA as a base to generate sulfonamides **17** and **18**, respectively (Scheme 1).

**Scheme 2:**
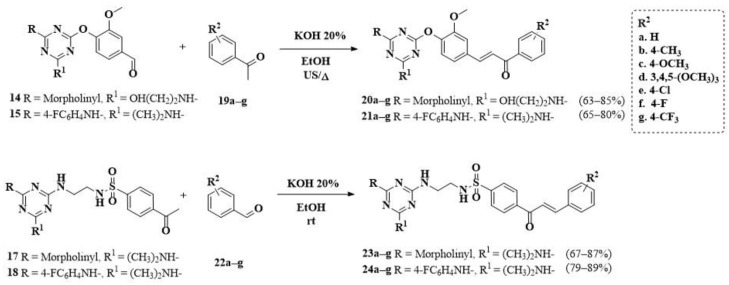
Synthesis of triazinyloxy-chalcones (**20,21**)**a**–**g** and triazinylamino-chalcones **(23,24**)**a**–**g.**

Using carbonyl precursors **14**,**15** and **17**,**18** as starting materials, the triazinyloxy-chalcones (**20,21**)**a**–**g** and triazinylamino-chalcones (**23,24**)**a**–**g** were obtained by Claisen–Schmidt condensation reactions with acetophenones **19a**–**g** and benzaldehydes **22a**–**g**, respectively (Scheme 2). These chalcones were obtained in the range of 65% to 93% yield and their structures were elucidated by FTIR, ^1^H, and ^13^C NMR and mass spectrometry (Supplementary Material). To illustrate the main spectroscopic characteristics of these compounds, chalcone **23f** was taken as a reference. The mass spectrum confirms the formation of this compound by presenting the molecular ion peak at *m*/*z* 555, which corresponds to its expected mass. The ^1^H NMR spectrum run at 400 MHz in CDCl_3_ shows two doublets at 7.84 and 8.01 ppm (*J* = 8.4 Hz) corresponding to the protons of the *para*-substituted 4-acylbenzenesulfonyl moiety. A triplet and a double doublet are observed at 7.12 (*J* = 8.6 Hz) and 7.64 ppm (*J* = 8.6, 5.4 Hz), respectively, corresponding to the protons of the *para*-F-substituted ring. Finally, at 7.39 and 7.78 ppm, two doublets are observed (*J* = 15.7 Hz) corresponding to the vinylic protons of the α,β-unsaturated moiety, confirming that the new double bond formed in product **23f** possesses an *E* configuration.

**Scheme 3:**
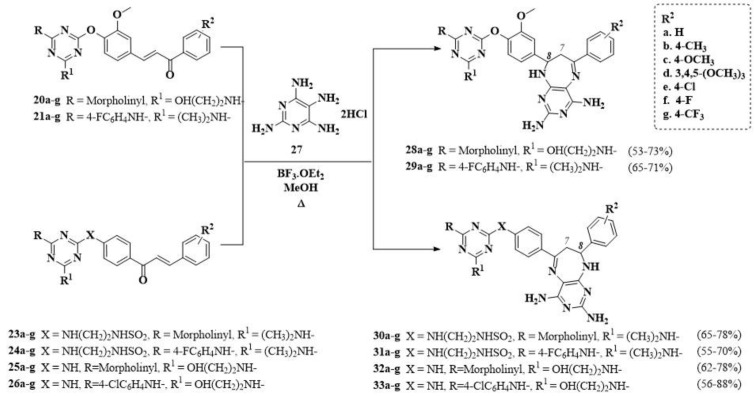
Synthesis of the target triazinyloxy- and triazinylamino-pyrimido[4,5-*b*][1,4]diazepines (**28**–**33**)**a**–**g**.

The final target products (i.e., triazinyloxy- and triazinylamino-pyrimido[4,5-*b*][1,4]diazepines) (**28**–**31**)**a**–**g** (Scheme 3) were obtained with high regioselectivity by reaction of chalcones (**20,21**)**a**–**g** and (**23,24**)**a**–**g**, respectively, with an excess of 2,4,5,6-tetraaminopyrimidine dihydrochloride **27** (1,4-dinucleophile) under stirring in refluxing methanol and using BF_3_∙OEt_2_ as catalyst. In the same way, diazepines (**32,33**)**a**–**g** were obtained starting from the chalcones (**25,26**)**a**–**g** synthesized elsewhere [50]. Reaction yields ranged from 50% to 90% and all synthesized diazepines were characterized by FTIR, ^1^H, ^13^C NMR, and mass spectrometry (Supplementary Material). Particularly, the ^1^H NMR spectrum (run in DMSO-*d_6_*) of product **32e** shows the signals corresponding to the N-H (a singlet at 7.00 ppm) and the diastereotopic protons (AMX system) of the diazepinic ring formed. The signal assigned to the H_7A_ proton appears as a doublet at 2.75 ppm with coupling constant ^2^*J*_AM_ = 14.2 Hz; the signal for the H_7M_ proton appears as a doublet at 3.77 ppm with coupling constants ^2^*J*_AM_ = 14.2 Hz and ^3^*J*_MX_ = 6.0 Hz, while the signal corresponding to the H_8X_ proton appears as an unresolved doublet at 5.05 ppm, confirming the formation of the new diazepine moiety. Additionally, in the ^13^C NMR spectrum, the C-7 and C-8 carbon atoms of the diazepine ring were observed at 38.2 ppm and 57.0 ppm, while a molecular ion peak at *m/z* 602:604 with an isotopic profile [M]^+^:[M + 2]^+^ 18:6 was observed in the mass spectrum agreeing with the formation of the structure **32e**.

### 2.2. Biological Activity Studies

#### 2.2.1. Anticancer Activity

**Figure 2:**
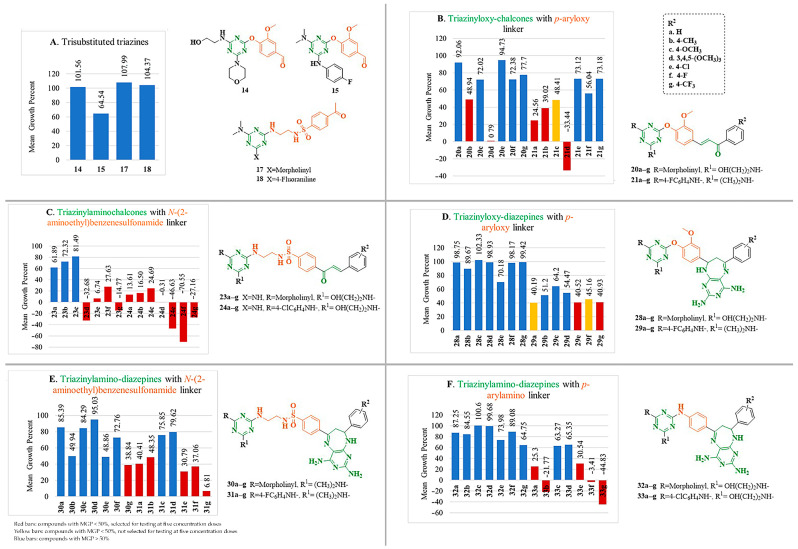
Bar chart of the mean growth percent (MGP) against 60 cancer cell lines for the trisubstituted triazines **14**–**15** and **17**–**18**, chalcones (**20**,**21**)**a**–**g** and (**23**,**24**)**a**–**g**, and diazepines (**28**–**33**)**a**–**g**.

All the synthesized trisubstituted triazines **14**,**15** and **17**,**18**, chalcones (**20**,**21**)**a**–**g** and (**23**,**24**)**a**–**g**, and diazepines (**28**–**33**)**a**–**g** were evaluated through in vitro assays at one dose of 10 μM against 60 cancer cell lines of nine cancer types (*Leukemia*, *Lung*, *Colon*, *Melanoma*, *Renal*, *Prostate*, *CNS*, *Ovarian*, and *Breast* cancer) by the National Cancer Institute (NCI) [76]. The results were reported as a graph of growth percentages (PC) available for analysis through the COMPARE program, which permits us to know the inhibition of growth (i.e., %IG = 100 − %PC) and lethality (%PC values less than 0). Additionally, the mean of the growth percentages (MGP) of each compound against all the 60 cancer cell lines is also reported, which is used as one of the selection criteria to continue with tests at five concentration doses. An MGP value less than 50% or with negative values indicates that the compound exhibits outstanding anticancer activity. Figure 2 shows the bar charts of the MGP values for all compounds evaluated. These diagrams are separated according to the linker (*p*-aryloxy, *N*-(2-aminoethyl)benzenesulfonamide or *p*-arylamine; orange moiety) between the triazine ring and the α,β-unsaturated carbonyl system or the diazepine ring (green moiety). In red and yellow background, compounds with MGP < 50% are highlighted; in red are the compounds that were evaluated at five concentration doses.

None of the trisubstituted triazine precursors showed remarkable anticancer activity; however, several triazinylamino- and triazinyloxy-chalcones from these precursors enhanced their activity, such as **20b,d**, **21a**–**d**, **23d**–**g**, and **24a**–**g**, (Figure 2A–C). It should be noted that the triazinylamino-chalcones that contain *N*-(2-aminoethyl)benzenesulfonamide moiety **24a**–**g** as a linker exhibited outstanding activity with MGP values below 25% and even with negative values. If the latter are compared with chalcones **21a**–**g**, which have the dimethylamino and 4-fluoroanilino substituents on the triazine ring in common, it can be noted that triazinylamino-chalcones **24a**–**g** are more active than those containing the *p*-aryloxy linker (**21a**–**g**), except for derivative **21d**. This suggests that the *N*-(2-aminoethyl)benzenesulfonamide moiety potentiates anticancer activity. In contrast, the MGP of the chalcone series **23a**–**g** evidenced that the presence of the 4-fluoroanilino substituent enhances the activity of the chalcones containing the *N*-(2-aminoethyl)benzenesulfonamide moiety except for **23d** (R^2^ = 3,4,5-(OCH_3_)_3_).

Within the family of diazepines containing the *N*-(2-aminoethyl)benzenesulfonamide linker **31a**–**g** it was observed that compounds **31a,b,e,f,g** exhibited potent activity. The most active diazepines **31a**–**g** and **33a**–**g** (moiety in common: R = Cl or F) coincide with the same substituents R^2^ = **a**:-H, **b**:-CH_3_, **e**:-Cl, **f**:-F, **g**:-CF_3_. On the other hand, comparing the MGP values of the diazepines **31a**–**g** with those of their precursors **24a**–**g**, the latter exhibited higher activity. The series of diazepines containing the *p*-aryloxy moiety **28a**–**g** did not show a MGP < 50%, while their analogous diazepines **29a,e,f,g** did show MGP values less than 50%, with **29e,g** being selected for five-dose assays.

Chalcones containing the *N*-(2-aminoethyl)benzenesulfonamide moiety **24a**–**g** were the only ones having activity in the entire series (**a**–**g**) and better activity when compared to chalcones **21a**–**g** containing the *p*-aryloxy linker with 4-F/Cl-anilino substituent. Likewise, the aromatic substituents 4-Cl-anilino and 4-F-anilino on the triazine ring enhanced the activity of chalcones and diazepines in most cases compared to the morpholino substituent (compounds **24a**–**c**,**e**–**g, 29a**–**g**, and **33a**–**g** were more active than **23a**–**c**,**e**–**g**, **28a**–**g**, and **32a**–**g**, respectively). Regarding the R^2^ substituents in chalcones and diazepines, there was not observed a marked chemical pattern that can be related to anticancer activity.

Based on the above results, the most active compounds were evaluated by the NCI at five concentration doses for their cytostatic (GI_50_) and cytotoxic (LC_50_) activity against 60 cancer cell lines (see Supplementary Material). Table 2 highlights the four cell lines that were most sensitive to each derivative (ordered from lowest to highest GI_50_) and contrasts the GI_50_ values with those of the antineoplastic standard drug 5-fluorouracil (5-FU, thymidylate synthase inhibitor). Analysis of these data showed that compounds **20b,d**, **21a**,**b**,**d**, **23a,d**–**g**, **24a**–**g**, **29e,g**, **30g**, **31a**,**b**,**e**–**g**, **33a**,**b**,**e**–**g** exhibited significant cytostatic activity with GI_50_ values between <0.01–100 μM and cytotoxic activity with LC_50_ values between 4.09 µM to >100 µM, against all cancer cell lines. Chalcones **20d**, **21d**, and **24f** and diazepine **33g** showed the lowest GI_50_ range values (highlighted in green), indicating that they were highly active for all cell lines. The latter points out that chalcones **20d**, **21a**, **21d**, **23a**, **23d**, **24c**, **24d** and diazepines **29e**,**g**, **30g**, **31a**–**b**,**e**–**g**, **33a**–**b**,**e**–**g** showed higher activity against several cell lines than the standard drug 5-FU (Table 2, highlighted in pink). Remarkably, diazepine **33a** exhibited the best anticancer activity, with a GI_50_ value < 10 nM against the MOLT-4 cell line of the *Leukemia* panel. The above results demonstrate that the triazine-based chalcone/diazepine hybrids **20b**,**d**, **21a**,**b**,**d**, **23a**,**d**–**g**, **24a**–**g**, **29e**,**g**, **30g**, **31a**,**b**,**e**–**g**, **33a**,**b**,**e**–**g** are important hits and a starting point for further optimization of their anticancer activity.

#### 2.2.2. Antibacterial Activity

The antibacterial activity of trisubstituted triazines **14**,**15** and **17**,**18**, chalcones (**20**,**21**)**a**–**g** and (**23**,**24**)**a**–**g**, and diazepines (**28**–**33**)**a**–**g** was tested against gram-positive (*Staphylococcus aureus* (ATCC 25923, ATCC 43300, and VISA strains), and gram-negative bacteria (*Pseudomonas aeruginosa ATCC*, *Klebsiella pneumoniae* ATCC 700603, BAA1645, *Escherichia coli* ATCC 25922, and *Neisseria gonorrhoeae* ATCC 49226). The anti-TB effect of the compounds was assessed on *Mycobacterium tuberculosis* ATCC 27294. None of the evaluated compounds showed inhibitory effects on *P. aeruginosa*, *K. pneumoniae*, and *E. coli*. Diazepine **33g** was active against *S. aureus* ATCC 43300, a methicillin resistant strain (MRSA), with MIC = 31.25 µg/mL (tetracycline MIC = 0.05 µg/mL, range: 0.05–0.25 µg/mL).

Additionally, we found that triazinylamino- and triazinyloxy-diazepines **28a**–**g**, **29a**,**c**,**d**,**f**, **30d**, **31a**–**f**, **32a**,**b**,**f**,**g**, **33a**,**b**,**c**,**f** showed inhibition against *N. gonorrhoeae* with MIC values between 0.25 and 62.5 µg/mL (Table 3). This could suggest that the pyrimido[4,5-*b*][1,4]diazepine moiety may be associated to the activity against *N. gonorrhoeae* [80]. Moreover, diazepines containing the *p*-aryloxy linker **28a**–**g** and *N*-(2-aminoethyl)benzenesulfonamide linker **31a**–**f** showed inhibition with MIC values between 0.25–500 µg/mL and 0.5–8 µg/mL, being, the triazinylamino-diazepine **31f** the compound with the highest activity against *N. gonorrhoeae* with a MIC value 0.25 µg/mL, which is similar to that for penicillin[81]. These findings are relevant because *N. gonorrhoeae* is the second most prevalent sexually transmitted bacterial infection worldwide, and has developed resistance to the first line treatment and has emerged as a thread for public health [82,83]. Some of the evaluated compounds could become molecules for future development in this regard.

##### Antitubercular Activity

**Figure 3:**
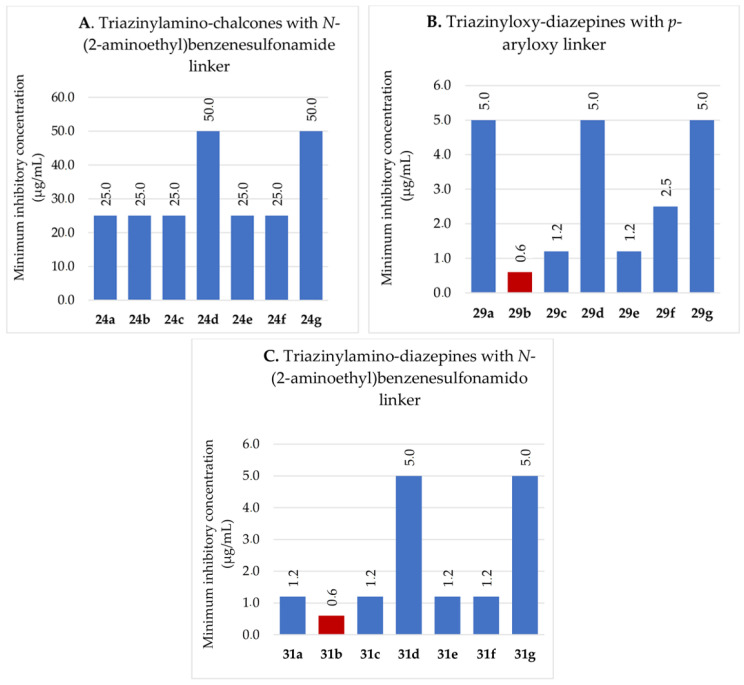
Bar chart of the In vitro Minimum Inhibitory Concentration (MIC) values for the triazinylamino-chalcones **24a**–**g** and triazinyloxy- and triazinylamino-pyrimido[4,5-*b*][1,4]diazepines **29a**–**g** and **31a**–**g**, against *M. tuberculosis* ATCC 27294. Control: Isoniazid (0.1 µg/mL).

It was determined that the triazinylamino-chalcones **24a**–**g** exhibited anti-TB activity inhibiting the growth of *M. tuberculosis* H37Rv at concentrations between 25 and 50 µg/mL (Figure 3A). However, as it was observed for *N. gonorrhoeae*, the most inhibitory compounds were those with a pyrimido[4,5-*b*][1,4]diazepine core, being **29a**–**g** and **31a**–**g** series the most active with MIC values between 0.6 and 5 µg/mL (Figure 3B,C). Previous studies have confirmed the anti-TB activity of some pyrimido-diazepine derivatives [84]. In addition, it has been shown that some compounds containing this heterocyclic system can inhibit the action of tyrosine [62] and serine-threonine kinases [85]. Unlike most bacteria, which use histidine kinases as major components in signal transduction, *M. tuberculosis* possesses 11 serine-threonine kinases, two of which (PknA and PknB) have been shown to be essential for its growth in vitro [85]. Therefore, the activity observed for compounds **29a**–**g** and **31a**–**g** could be due to the inhibition of one of these proteins. In addition to the pyrimido[4,5-*b*][1,4]diazepine moiety, the 4-fluoroanilino substituent seems to play a crucial role in the interaction with the target, since compounds with morpholino as substituent did not displayed activity. As shown in Figure 3B,C, compounds **29b** and **31b** exhibited the most outstanding anti-TB activity both containing a methyl group as R^2^ substituent, which could favor Van der Waals interactions, giving stability to the molecule when interacting with the target.

#### 2.2.3. Antifungal Activity

Several of the obtained triazine derivatives were evaluated against sensitive fungal species comprising of two yeasts (*Candida albicans* (ATCC 10231), *Cryptococcus neoformans* (ATCC 32264)), three dermatophytes (*Microsporum gypseum* (CCC 115), *Trichophyton rubrum* (CCC 134-2000), *Trichophyton mentagrophytes* (CCC 202-2000)), and three *Aspergillus* fungi ((*A. fumigatus* (ATCC 26934), *A. niger* (ATCC 9029), and *A. flavus* (ATCC 9170)). The minimum inhibitory concentration (MIC) and the minimum fungicidal concentration (MFC) of these compounds were determined by the M27-A3 and M38-A8 microdilution method (CLSI) [86,87].

Two compounds (**29e** and **31g**) exhibited outstanding antifungal activity with MICs = 62.5 μg/mL. Thus, triazinyloxy-chalcone **29e** was active against *T. rubrum*, while triazinylamino-chalcone **31g** was active against *T. mentagrophytes* and *A. fumigatus*. These chalcones contain 4-chloro/4-fluorophenylamine and dimethylamine as substituents attached to the triazine ring, and their linker moieties have both H-bond donors and acceptors. This suggests that this structural design can be further tested for the optimization of its antifungal activity. On the other hand, triazine **17**, chalcones **23e**, **24c**,**e**, **21d**, and diazepines **30d**,**g**, **29f**,**g** showed marginal activity (MIC = 125 μg/mL) against various fungal species (Table 4).

#### 2.2.4. In Silico Physicochemical Parameter Predictions

The physicochemical features of the most active compounds were determined by in silico analysis using the SwissADME platform [89] (Table 5). All the active compounds showed violations of the Lipinski rule of five and the pharmacokinetics need improvement as gastrointestinal absorption and cytochrome interactions was poor. However, the synthetic accessibility indicates the feasibility for analogues synthesis of these compounds.

#### 2.2.5. Hemolytic Activity

The ability of the compounds showing better anticancer, antibacterial, and antifungal activity (**17**, **20b,d**, **21a,b,d,f**, **23a**–**d**, **24a**–**g, 28a**–**g, 29a,c,d,f**, **30a,b,d,e,g**, **31a**–**g**, **32a,b,f,g** and **33a**–**c,e**–**g**) to induce hemolysis in human red blood cells (huRBC) was evaluated following the spectrophotometric cytotoxicity method [90]. Table 6 reports the hemolytic activity obtained for these compounds evaluated at 200 μg/mL. Most of the compounds showed low hemolytic activity (<25%,) suggesting that they have low membrane interactions and toxicity. Only diazepines **29d**, **28g**, and **33b** induced membrane lysis with >75% hemolysis.

#### 2.2.6. Toxicity Studies

Toxicity, median lethal dose (LD_50_), and toxicity classification were predicted for the six most active compounds of each activity studied: **33a** (anticancer), **29b**, **31b**, and **31f** (antibacterial) and **29e** and **31g** (antifungal) using Protox II [91] (Table 7). Most compounds (five/six) showed predicted immunotoxicity, whereas **29b**, **31f**, and **31g** showed predicted carcinogenicity and **31f**, **31g**, and **33a** may have cytotoxic effects; however, these alerts were predicted with low likelihood (cs < 0.7). None of the compounds were predicted as mutagenic or hepatotoxic chemicals. Four out of six compounds were classified as low toxic (class 5).

Additionally, compounds **20b**,**d**, **21a**,**b**,**d**, **23d**, **30d**,**e**,**g**, **33e** were tested for toxicity in the *Galleria mellonella* model. Nine out of ten tested compounds showed LD_50_ ≥ 325 mg/Kg and eight of them LD_50_ ≥ 650 mg/Kg. Although some compounds (**21a**, **30d**, **30g**, **21d**) induced mortality at the higher doses, it was only ≤25%. This suggests that most compounds are likely to have a very low toxic nature.

## 3. Materials and Methods

All solvent and reagents were obtained from commercial sources and were used without purification. Thin layer chromatography analyses were performed on 0.2 mm pre-coated aluminium plates of silica gel 60 F254 (Merck, Darmstadt, Hesse, Germany). Melting points were taken on a Stuart SMP10 melting point device (Cole-Parmer Ltd., Stone, Staffordshire, UK) and are uncorrected. FTIR spectra were determined on an IRAffinity-1 spectrophotometer (Shimadzu, Columbia, MD, USA). ^1^H and ^13^C NMR spectra were measured on a Bruker 400 Ultrashield Avance II spectrometer (Bruker, Billerica, MA, USA) operating at 400 and 100 MHz, respectively, using DMSO-ɗ_6_ and CDCl_3_ as solvents and TMS as internal standard. Mass spectra were obtained on a Shimadzu-GCMS-QP2010 spectrometer (Shimadzu, Kyoto, Honshu, Japan) equipped with a Rxi-1HT GC Capillary Column (30 m, 0.25 mm ID, 0.25 um df, phase: dimethyl polysiloxane) and operating at 70 eV. Elemental analyses were performed on a Thermo Finnigan Flash EA1112 CHN elemental analyzer (Thermo Fischer Scientific Inc., Madison, WI, USA) and the values are within ±0.4% of the theoretical values.

### 3.1. Chemistry

#### 3.1.1. General Procedure for the Synthesis of Monosubstituted Triazines (**5**–**7**)

The synthesis of derivatives **5** [68,69,70] and **6** [71,72] was reported in previous studies. Monosubstituted triazine **7** was obtained as follows: A solution of 4-hydroxy-3-methoxybenzaldehyde (13 mmol) in acetone (20 mL) was added slowly to a solution of 2,4,6-trichloro-1,3,5-triazine **4** (35.1 mmol) in acetone (35 mL) at −5–0 °C. The mixture was neutralized with 20% Na_2_CO_3_. The content was poured onto crushed ice, filtered, and washed with water. The required equivalents of the reagents and reaction time are reported in Table 1.

#### 3.1.2. General Procedure for the Synthesis of Disubstituted Triazines (**8**–**11**)

The synthesis of derivatives **8** [74], **9** [71] and **11** [73] was reported in previous studies. Disubstituted triazine **10** was obtained as follows: 4-hydroxy-3-methoxybenzaldehyde (13 mmol)was added slowly to a solution of monosubstituted triazine **6** (13 mmol) in acetone (35 mL) at room temperature. The mixture was neutralized with 20% Na_2_CO_3_ and after 3 h the content was poured onto crushed ice, filtered, and washed with water. The required equivalents of the reagents, the solvent used, the temperature and the reaction time were reported in Table 1.

#### 3.1.3. Procedure for the Synthesis of Trisubstituted Triazine (**12**)

A mixture of disubstituted triazine **8** (6.2 mmol) and ethylenediamine (99.2 mmol) was stirred at room temperature for 18 h. The mixture was dissolved in chloroform and washed with water. The organic phase was dried with MgSO_4_, filtered, and concentrated under reduced pressure.

#### 3.1.4. Procedure for the Synthesis of Trisubstituted Triazine (**13**)

A mixture of disubstituted triazine **9** (5.6 mmol) and ethylenediamine (89.6 mmol) was refluxed for 4 h. The mixture was poured onto crushed ice, filtered, and washed with water.

#### 3.1.5. Procedure for the Synthesis of Trisubstituted Triazine (**14**)

A mixture of disubstituted triazine **10** (3 mmol) and dimethylamine (3 mmol) in dioxane (15 mL) was stirred at room temperature for 1 h. The mixture was poured onto crushed ice, filtered, and washed with water.

#### 3.1.6. Procedure for the Synthesis of Trisubstituted Triazine (**15**)

A mixture of disubstituted triazine **11** (3 mmol) and ethanolamine (4.5 mmol) in dioxane (15 mL) was stirred at room temperature for 24 h. The mixture was poured onto crushed ice, filtered, and washed with water.

#### 3.1.7. General Procedure for the Synthesis of Trisubstituted Triazines (**17**,**18**)

A mixture of the respective trisubstituted triazine **12**/**13** (4 mmol), 4-acetylbenzenesulfonyl chloride **16** (4.8 mmol) and TEA (0.5 mL) in ethanol (15 mL) was stirred at room temperature for 24 h. The reaction crude was dissolved in chloroform and washed with water. The organic phase was dried with MgSO_4_, filtered, and concentrated under reduced pressure. The product was then purified by column chromatography on silica gel employing 10:1 of ethyl acetate:hexane as eluent.

#### 3.1.8. General Procedure for the Synthesis of Chalcones (**23**,**24a**–**g**)

A mixture of the respective trisubstituted triazine **17**/**18** (0.22 mmol), the respective benzaldehyde **22a**–**g** (0.27 mmol), and 0.2 mL potassium hydroxide (20%) in ethanol (3 mL) was stirred at room temperature for 3–8 h. The solid thus formed was filtered and washed with cold ethanol.

#### 3.1.9. General Procedure for the Synthesis of Chalcones (**20a**–**g**)

A mixture of the trisubstituted triazine **14** (0.27 mmol), the respective acetophenone **19a**–**g** (0.22 mmol) and 150 µL of a solution of potassium hydroxide (20%) in ethanol (3 mL) were sonicated (US) for 6–8 h. The content of **20a-c,e,g** was filtered, and washed with cold ethanol. The content of **20d** and **20f** were dissolved in chloroform and washed with water. The organic phase was dried with MgSO_4_, filtered, and concentrated under reduced pressure. The product was then purified by column chromatography on silica gel employing 20:1 of dichloromethane:methanol as eluent.

#### 3.1.10. General Procedure for the Synthesis of Chalcones (**21a**–**g**)

A mixture of the trisubstituted triazine **15** (0.26 mmol), the respective acetophenone **19a**–**g** (0.22 mmol), and 150 µL of a solution of potassium hydroxide (20%) in ethanol (3 mL) was heated under reflux for 2–5 h. The content was dissolved in chloroform and washed with water. The organic phase was dried with MgSO_4_, filtered, and concentrated under reduced pressure. The products were purified by column chromatography on silica gel employing 2:1 hexane:ethyl acetate as eluent.

#### 3.1.11. General Procedure for the Synthesis of the 8,9-dihydro-7H-pyrimido[4,5-*b*][1,4]Diazepines (**28**–**33**)**a**–**g**

A mixture of the respective chalcone (**20**,**21**)**a**–**g** and (**23**–**26**)**a**–**g** (0.5 mmol), 2,4,5,6-tetraaminopyrimidine dihydrochloride **27** (1.5 mmol) and BF_3_∙OEt_2_ (0.25 mL) in methanol (10 mL) was heated under reflux for 3–8 h. After cooling to room temperature, the content was quenched with NH_4_OH 6% until neutralization. The content was dissolved in chloroform and washed with water. The organic phase was dried with MgSO_4_, filtered, and concentrated under reduced pressure. The product was purified by column chromatography on silica gel employing 10:1 dichloromethane:methanol as eluent.

#### 3.1.12. Anticancer Activity

The human cancer cell lines of the cancer screening panel were grown in an RPMI–1640 medium containing 5% fetal bovine serum and 2 mM L–glutamine. For a typical screening experiment, cells were inoculated into 96–well microtiter plates. After cell inoculation, the microtiter plates were incubated at 37 °C, 5% CO_2_, 95% air, and 100% relative humidity for 24 h prior to the addition of the tested compounds. After 24 h, two plates of each cell line were fixed in situ with TCA to represent a measurement of the cell population for each cell line at the time of sample addition (Tz). The samples were solubilized in dimethyl sulfoxide (DMSO) at 400–fold the desired final maximum test concentration and stored frozen prior to use. At the time of compound addition, an aliquot of frozen concentrate was thawed and diluted to twice the desired final maximum test concentration with complete medium containing 50 μg/mL gentamicin. An additional four 10–fold or 1/2 log serial dilutions were made to provide a total of five drug concentrations plus the control. Aliquots of 100 μL of these different sample dilutions were added to the appropriate microtiter wells already containing 100 μL of medium, resulting in the required final sample concentrations. After the tested compounds were added, the plates were incubated for an additional 48 h at 37 °C, 5% CO_2_, 95% air, and 100% relative humidity. For adherent cells, the assay was terminated by the addition of cold TCA. Cells were fixed in situ by the gentle addition of 50 μL of cold 50% (*w*/*v*) TCA (final concentration, 10% TCA) and incubated for 60 min at 4 °C. The supernatant was discarded, and plates were washed five times with tap water and air dried. Sulforhodamine B (SRB) solution (100 μL) at 0.4% (*w*/*v*) in 1% acetic acid was added to each well, and plates were incubated for 10 min at room temperature. After staining, unbound dye was removed by washing five times with 1% acetic acid and the plates were air dried. Bound stain was subsequently solubilized with 10 mM trizma base, and the absorbance was read on an automated plate reader at a wavelength of 515 nm. Using the seven absorbance measurements [time zero (Tz), control growth in the absence of drug, and test growth in the presence of drug at the five concentration levels (Ti)], the percentage growth was calculated at each of the drug concentrations levels. Percentage growth inhibition was calculated as: [(Ti − TZ)/(C − TZ)] × 100 for concentrations for which Ti > Tz, and [(Ti − TZ)/TZ] × 100 for concentrations for which Ti < Tz. Two dose−response parameters were calculated for each compound. Growth inhibition of 50% (GI_50_) was calculated from [(Ti − TZ)/(C − TZ)] × 100 = 50, which is the drug concentration resulting in a 50% lower net protein increase in the treated cells (measured by SRB staining) as compared to the net protein increase seen in the control cells and the LC_50_ (concentration of drug resulting in a 50% reduction in the measured protein at the end of the drug treatment as compared to that at the beginning), indicating a net loss of cells; calculated from [(Ti − TZ)/TZ] × 100 = −50). Values were calculated for each of these two parameters if the level of activity is reached; however, if the effect was not reached or was exceeded, the value for that parameter was expressed as greater or less than the maximum or minimum concentration tested [76,77,78,79].

#### 3.1.13. Antibacterial Activity

Stock solutions (100 mg/mL) of the respective compounds were prepared in dimethylsulfoxide (DMSO) and diluted to a final concentration of 500 µg/mL. An initial screening for bacterial inhibition was performed by the agar diffusion method. Briefly, sterile Mueller Hinton agar was prepared in Petri dishes and inoculated with a bacterial suspension prepared in trypticase soy broth (TSB) and adjusted to 1.5 × 10^8^ CFU/mL (0.08–0.1 OD at 600 nm) [92]. 6 mm diameter wells were drilled into the agar, and 10 µL of each compound (stock solution) was deposited into each well. DMSO and TSB were included as negative controls. Gentamicin and trimethoprim sulfamethoxazole were included as a positive control for growth inhibition. Derivatives showing growth inhibition were tested at least twice before being selected for the microdilution test. For *N. gonorrhoeae*, the agar diffusion method was also used for screening with some modifications. For this method, 200 µL of a bacterial suspension (1.5 × 10^8^ CFU/mL) was inoculated onto Gonococcal Agar (GC) supplemented with 1% isovitalex, and then compounds were added to wells as mentioned above and incubated at 35–36.5 °C in 5% CO_2_ for 48 h. Penicillin and tetracycline were used as controls [93].

Antitubercular screening was carried performed using the broth microdilution method [94]. Briefly, 100 µL of Middlebrook 7H9 culture medium supplemented with 10% Middlebrook OADC and test compounds at a concentration of 100 µg/mL were placed in clear U-bottom polystyrene 96-well microplates. Wells with culture medium without compounds were prepared as growth control. Bacterial suspensions were prepared from Lowenstein Jensen agar cultures and adjusted to 5 × 10^6^ CFU/mL. Once the required density was reached, a 1:20 dilution was realized in Middlebrook 7H9 culture medium supplemented with 10% Middlebrook OADC and 100 µL were inoculated into the wells containing culture medium supplemented with the compounds. Microplates were incubated at 37 °C for 14–21 days. After time, a visual reading of the growth in each well was performed. Those wells in which no growth was observed were taken as positive for anti-TB activity.

*Microdilution test:* The Minimum Inhibitory Concentration was determined for those compounds with reproducible and visible antibacterial inhibition in the screening test. The bacterial suspensions were adjusted with Mueller Hinton Broth (MHB) to a concentration of 5 × 10^5^ to 8 × 10^5^. The stock solution of the new compounds was diluted in MHB containing 5% DMSO and 0.1% Tween 80 and added to 90 µL of inoculum. The microplates were incubated for 24 h at 35–37 °C. The determination of the MIC of the new compounds that presented anti-TB activity was developed as described in the previous section, including decreasing concentrations of each compound. The MIC was defined as the lowest concentration with visible inhibition of bacterial growth, and/or detected using Resazurin (125 µg/mL). Isoniazid was included as growth inhibition controls. The experiments were performed in duplicate.

For the analysis of inhibition against *N. gonorrhoeae*, compounds that showed growth inhibition on evaluative screening were then tested for MIC on agar plates as described by the Center for Disease Control Prevention and the Clinical and Laboratory Standards Institute with modifications [93]. Briefly, GC agar supplemented with 1% isovitalex was prepared with increasing concentrations of the new compounds and inoculated with 10 µL of a bacterial suspension (1 × 10^4^ CFU). The lowest concentration of the compound that inhibited bacterial growth was determined as the MIC. Bacterial growth was examined and verified using the oxidase test. The experiments were performed in duplicate in at least two independent assays.

#### 3.1.14. Antifungal Activity

Broth microdilution techniques were performed in 96-well microplates according to the Reference Method for Broth Dilution Antifungal Susceptibility Testing of Yeasts, document M27-A3 [86] and of Filamentous Fungi M38-A8 [87]. For assay, stock solutions of each compound in DMSO (maximum concentration 1%) were added to test wells and diluted with RPMI-1640, to final concentrations of 250–0.98 µg/mL. An inoculum suspension (100 µL) was added to each well (final well volume = 200 µL). One growth control well (containing medium, inoculum, and the same amount of DMSO as used for each compound) and one sterility control well (sample, medium, and sterile water instead of inoculum) were included for each fungus tested. Microtiter plates were incubated in a dark, humid chamber at 30 °C for the time necessary for each fungus. Amphotericin B, Terbinafine, Fluconazole and Itraconazole were used as a positive control. The tests were performed in triplicate.

#### 3.1.15. Hemolytic Activity

Compounds that showed activity were evaluated for their ability to induce hemolysis following the cytotoxicity method by spectrophotometry. The method was adapted from Conceição et al. [90] with modifications. 240 µL of human red blood (huRBC) adjusted at 5% of hematocrit in phosphate-buffered saline were placed in each well of a 96-well plate and subsequently exposed to 200 µg/mL of selected compounds (10 µL of a 5 mg/mL solution of test compound dissolved in 5% DMSO, 0.1% Mueller Hinton broth Tween-80). As a positive control for hemolytic activity, 10 µL of 1% sodium dodecyl sulfate was added. As a negative control, the medium without the compounds to be tested was employing. Free hemoglobin was measured after 24 h of incubation at 37 °C by spectrophotometry (420 nm Cytation 3M, BioteK). Non-specific absorbance was subtracted from a blank. The determinations were made in triplicate in at least two independent experiments.

#### 3.1.16. Toxicity Studies In Vivo

Compounds biologically active were tested for toxicity in the *Galleria mellonella* model. *G mellonella* larvae were cultivated in the laboratory and healthy, beige larvae weighing 150–200 mg, were selected for toxicity assays. Groups of ten six- instar larvae were inoculated with 10 µL or 20 µL of each compound at concentration equivalent to 650 mg/Kg and incubated at 37 °C in darkness. The larval survival was monitored every 24 h for 5 days to determine the half lethal doses (LD_50_). The larvae were initially injected 30 mg/Kg and if most larvae (≥60%) survived after five days, the assay was performed using higher doses up to 650 mg/Kg [95].

## 4. Conclusions

Four new trisubstituted triazines **12**–**15** and **17**,**18**, four triazinyloxy- and triazinylamino-chalcone **20**–**21a**–**g** and **23**–**24a**–**g** series and six pyrimido[4,5-*b*][1,4]diazepine **28**–**33a**–**g** series were efficiently synthesized in successive reaction stages under mild conditions. The In vitro anticancer activity analyzes against 60 human cancer cells revealed that seventeen chalcones (**20b**,**d**, **21a**,**b**,**d**, **23a**,**d**–**g**, **24a**–**g**) and thirteen pyrimido[4,5-*b*][1,4]diazepines (**29e**,**g**, **30g**, **31a**,**b**,**e**–**g, 33a,b,e**–**g**) exhibited remarkable activity with GI_50_ values between 0.01–100 μM and LC_50_ between 4.09 μM to > 100 μM, being chalcones **20d**, **21a**, **21d**, **23a**, **23d**, **24c**, **24d** and diazepines **29e**, **29g, 30g, 31a**–**b,e**–**g**, **33a**–**b,e**–**g** more active against several cell lines than the standard drug 5-FU. The antibacterial activity studies showed that the triazinyloxy- and triazinylamino-pyrimido[4,5-*b*][1,4]diazepine hybrids exhibited the best growth inhibition profiles. Compound **33g** was active against *S. aureus* (ATCC 43300) with a MIC = 31.25 µg/mL and derivatives **29a**–**g** and **31a**–**g** exhibited outstanding activity against *M. tuberculosis* with MICs = 0.6–5 µg/mL, being compounds **29b** and **31b** the most active of the series. Among the active diazepines against *N. gonorrhoeae* (**28a**–**g**, **29a**,**c**,**d**,**f**, **30d**, **31a**–**f**, **32a**,**b**,**f**,**g**, **33a**,**b**,**c**,**f**) compound **31f** stands out, which showed activity comparable to that of the drug penicillin and low hemolytic activity. Regarding to the antifungal activity, triazinylamino-chalcone **29e** was active against *T. rubrum* and triazinyloxy-chalcone **31g** was active against *T. mentagrophytes* and *A. fumigatus* (MIC = 62.5 μg/mL, in all three cases). The low toxicity of most of the above compounds suggests that they are safe and non-toxic. These interesting biological profiles exhibited by synthesized 1,3,5-triazine-based chalcone/diazepine hybrids could offer an excellent framework for the development of potent anticancer, antibacterial, and antifungal agents through optimization processes.

## Figures and Tables

**Table 1:** Reaction conditions toward the synthesis of trisubstituted triazine precursors **12**–**15**.

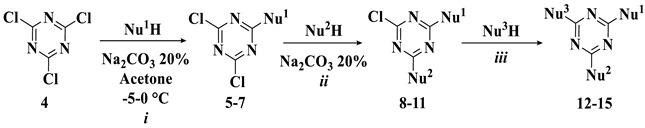			
Monosubstituted precursors 5–7			
Compound	Nu_1_H	*i* time, equivalents	Yield (%)
**5** [68,69,70]	Morpholine	2 h, **4**:Nu_1_H (1:1)	88
**6** [71,72]	4-Fluoroaniline	20 min, **4**:Nu_1_H (1:1)	97
**7**	4-Hydroxy-3-methoxybenzaldehyde	4 h, **4**:Nu_1_H (2.7:1)	96
**Disubstituted precursors 8**–**11**			
**Compound**	**Nu_2_H**	***ii*** **temperature, time, equivalents**	
**8** [74]	Dimethylamine	rt, 4 h, **5**:Nu_2_H (1:1)	90
**9** [71]	Dimethylamine	rt, 4 h, **6**:Nu_2_H (1:1.5)	77
**10**	4-Hydroxy-3-methoxybenzaldehyde	rt, 3 h, **6**:Nu_2_H (1:1)	91
**11** [73]	Morpholine	−5–0 °C, 7 h, **7**:Nu_2_H (1.2:1)	88
**Trisubstituted precursors 12**–**15**			
**Compound**	**Nu_3_H**	***iii*** **solvent, temperature, time, equivalents**	
**12**	Ethylenediamine	Solvent free, rt, 18 h, **8**:Nu_3_H (1:16)	78
**13**	Ethylenediamine	Solvent free, reflux, 4 h, **9**:Nu_3_H (1:16)	73
**14**	Dimethylamine	Dioxane, rt, 1 h, **10**:Nu_3_H (1:1)	83
**15**	Ethanolamine	Dioxane, rt, 24 h, **11**:Nu_3_H (1:1.5)	86

**Table 2:** Anticancer activity (GI_50_ ^a^ and LC_50_ ^b^) exhibited by compounds **20b**,**d**, **21a**,**b**,**d**, **23a**,**d**–**g**, **24a**–**g**, **29e**,**g**, **30g**, **31a**,**b**,**e**–**g**, **33a**,**b**,**e**–**g**, against the four most sensitive cancer cell lines ^c^ and compared to the GI_50_ values of the standard drug 5-FU.

Compound	Panel Name	Most Sensitive Cell Line	GI_50_ ^a^ (μM)	LC_50_ ^b^ (μM)	Range GI_50_^d^ (μM)	GI_50_ 5-FU (μM) (NS 18893) ^e^
**20b**	Melanoma	LOX IMVI	0.43	4.09	0.43–100	0.25
	Leukemia	SR	1.06	>100		0.02
	Renal cancer	UO-31	1.49	-		1.43
	Breast cancer	MCF7	1.50	>100		0.08
**20d**	Melanoma	LOX IMVI	0.64	4.99	0.64–4.72	0.25
	Leukemia	SR	0.78	>100		0.02
	Colon cancer	HCT-116	1.53	6.78		0.23
	Leukemia	HL-60(TB)	1.54	>100		2.30
**21a**	Melanoma	LOX IMVI	1.60	6.23	1.60–48.60	0.25
	Colon cancer	SW-620	1.81	8.70		0.93
	Non-small cell lung cancer	HOP-92	2.08	>100		77.98
	Leukemia	MOLT-4	2.56	>100		0.35
**21b**	Melanoma	LOX IMVI	1.84	6.69	1.84–51.90	0.25
	Colon cancer	HCT-116	2.28	24.40		0.23
	Colon cancer	SW-620	2.36	20.10		0.93
	Leukemia	MOLT-4	2.80	>100		0.35
**21d**	Leukemia	MOLT-4	0.47	>100	0.47–8.42	0.35
	Melanoma	LOX IMVI	0.48	4.11		0.25
	CNS cancer	U251	0.50	9.59		0.91
	Leukemia	SR	0.70	>100		0.02
**23a**	Colon cancer	HCT-116	1.82	7.10	1.82–20.20	0.23
	Melanoma	LOX IMVI	1.97	8.68		0.25
	Colon cancer	KM12	2.00	8.93		0.21
	Leukemia	K-562	2.19	>100		3.98
**23d**	Renal cancer	RXF 393	1.22	8.63	1.22–14.30	2.61
	Leukemia	RPMI-8226	1.39	>100		0.04
	Leukemia	K-562	1.46	>100		3.58
	CNS cancer	U251	1.54	5.54		0.91
**23e**	Melanoma	LOX IMVI	1.71	6.13	1.71–17.90	0.25
	Colon cancer	HCT-15	2.07	9.93		0.11
	Breast cancer	MCF7	2.37	34.60		0.08
	Leukemia	SR	2.52	>100		0.02
**23f**	Melanoma	LOX IMVI	1.63	6.19	1.63–16.10	0.25
	Leukemia	RPMI-8226	1.91	>100		0.04
	Colon cancer	HCT-15	2.15	13.70		0.11
	Ovarian cancer	IGROV1	2.27	25.40		1.22
**23g**	Melanoma	LOX IMVI	1.68	5.94	1.68–17.80	0.25
	Breast cancer	MCF7	1.68	6.63		0.08
	Leukemia	RPMI-8226	1.77	>100		0.04
	Ovarian cancer	IGROV1	1.77	6.97		1.22
**24a**	Colon cancer	HCT-116	1.72	6.10	1.72–14.60	0.23
	Breast cancer	MCF7	1.99	8.23		0.08
	Colon cancer	HCT-15	2.04	7.25		0.11
	Colon cancer	HCC-2998	2.35	17.90		0.05
**24b**	Colon cancer	HCT-116	1.75	6.17	1.75–17.40	0.23
	Breast cancer	MCF7	2.01	25.20		0.08
	Colon cancer	HT29	2.38	16.20		0.18
	Leukemia	RPMI-8226	2.73	>100		0.04
**24c**	Colon cancer	HT29	1.83	7.63	1.83–17.10	0.18
	Colon cancer	HCT-116	1.86	7.07		0.23
	Breast cancer	MCF7	1.93	46.10		0.08
	Non-small cell lung cancer	NCI-H522	2.55	67.10		7.28
**24d**	Renal cancer	RXF 393	1.45	6.74	1.45–21.80	2.61
	Colon cancer	HCC-2998	1.62	5.52		0.05
	CNS cancer	SF-539	1.71	5.62		0.06
	Melanoma	LOX IMVI	1.71	5.93		0.25
**24e**	Colon cancer	HCT-116	1.66	3.11	1.66–12.80	0.23
	Colon cancer	HT29	2.15	5.72		0.18
	Breast cancer	MCF7	2.28	6.33		0.08
	Leukemia	RPMI-8226	2.47	7.11		0.04
**24f**	Colon cancer	HCT-116	1.60	3.01	1.60–8.39	0.23
	Melanoma	LOX IMVI	1.66	3.07		0.25
	Renal cancer	SN12C	1.70	3.36		0.50
	Ovarian cancer	OVCAR-3	1.74	3.24		0.02
**24g**	Colon cancer	HCT-116	1.68	3.13	1.68–17.40	0.23
	Breast cancer	MCF7	1.70	3.56		0.08
	Colon cancer	HCC-2998	1.77	3.61		0.05
	Melanoma	LOX IMVI	1.86	3.84		0.25
**29e**	Renal cancer	RXF 393	2.46	>100	2.46–100	2.61
	Non-small cell lung cancer	HOP-92	3.00	>100		77.98
	CNS cancer	SNB-75	3.24	>100		78.70
	Renal cancer	CAKI-1	3.38	>100		0.07
**29g**	Renal cancer	RXF 393	1.22	34.10	1.22–100	2.61
	Renal cancer	CAKI-1	1.65	>100		0.07
	Leukemia	MOLT-4	1.71	>100		0.35
	Colon cancer	HCT-116	1.76	7.56		0.23
**30g**	Non-small cell lung cancer	HOP-92	7.48	>100	7.48–100	77.98
	Renal cancer	RXF 393	10.30	>100		2.61
	Leukemia	RPMI-8226	11.20	>100		0.04
	Breast cancer	MDA-MB-468	13.70	69.50		6.61
**31a**	Non-small cell lung cancer	HOP-92	2.18	9.96	2.18–99.70	77.98
	Renal cancer	RXF 393	2.55	8.95		2.61
	Leukemia	MOLT-4	3.11	>100		0.35
	Leukemia	RPMI-8226	3.32	>100		0.04
**31b**	Melanoma	LOX IMVI	2.09	5.11	2.09–78.80	0.25
	Leukemia	MOLT-4	2.20	7.53		0.35
	Renal cancer	RXF 393	2.27	9.12		2.61
	Colon cancer	HT29	2.27	4.65		0.18
**31e**	Melanoma	LOX IMVI	1.77	3.55	1.77–72.70	0.25
	Breast cancer	MDA-MB-468	1.92	4.92		0.07
	Renal cancer	RXF 393	2.27	8.75		2.61
	Non-small cell lung cancer	HOP-92	2.53	13.20		77.98
**31f**	Breast cancer	MDA-MB-468	2.15	6.24	2.15–96.00	6.61
	Leukemia	MOLT-4	2.45			0.35
	Renal cancer	RXF 393	2.48			2.61
	CNS cancer	SNB-75	2.53	74.00		78.70
**31g**	CNS cancer	SNB-75	2.13	86.00	2.13–86.00	78.70
	Renal cancer	RXF 393	2.57	18.80		2.61
	Breast cancer	MDA-MB-468	2.67	11.70		6.61
	Leukemia	MOLT-4	2.74	86.00		0.35
**33a**	Leukemia	MOLT-4	<0.01	>100	0.01–17.70	0.35
	Leukemia	HL-60(TB)	0.32	>100		2.51
	Leukemia	SR	0.55			0.02
	Leukemia	CCRF-CEM	0.95	>100		9.79
**33b**	Non-small cell lung cancer	HOP-92	1.30	6.30	1.30–16.70	77.98
	Leukemia	K-562	1.34	24.90		3.58
	Leukemia	MOLT-4	1.38	>100		0.35
	Renal cancer	RXF 393	1.45			2.61
**33e**	Leukemia	K-562	0.71	>100	0.71–14.80	3.58
	Leukemia	MOLT-4	0.79	>100		0.35
	Leukemia	SR	0.91	>100		0.02
	Leukemia	CCRF-CEM	1.14	>100		9.79
**33f**	Leukemia	K-562	0.49	>100	0.49–12.90	3.58
	Leukemia	MOLT-4	0.53	>100		0.35
	Leukemia	SR	0.67	>100		0.02
	Leukemia	CCRF-CEM	0.75	>100		9.79
**33g**	Leukemia	K-562	0.64	>100	0.64–2.55	3.58
	Leukemia	MOLT-4	0.89	>100		0.35
	Colon cancer	HCT-116	1.05	5.54		0.23
	Renal cancer	RXF 393	1.14	5.48		2.61

^a^ GI_50_ shows compound concentration resulting in a 50% diminution in the net protein increase (as measured by SRB staining) in control cells during the drug incubation, determined at five concentration levels (100, 10, 1.0, 0.1, and 0.01 µM). GI_50_ values highlighted in pink (of our compounds) are lower than GI_50_ values of 5-FU. ^b^ LC_50_ is a parameter of cytotoxicity that reflects the molar concentration needed to kill 50% of the cells. ^c^ Data obtained from NCI’s in vitro disease-oriented human cancer cell lines screen in µM [76,77,78,79]. ^d^ Range of GI_50_ values against the 60 cancer cell lines, entries highlighted in green show the lowest GI_50_ ranges. ^e^ Activity values against human cancer cell lines shown by 5-FU correspond to those reported by: https://dtp.cancer.gov/dtpstandard/cancerscreeningdata/index.jsp, accessed on 12 October 2023.

**Table 3:** Minimum inhibitory concentration (MIC) values for triazine derivatives active against *N. gonorrhoeae* ATCC 49226.

Compound	MIC (μg/mL)	Compound	MIC (μg/mL)	Compound	MIC (μg/mL)
**15**	≥500	**29c**	62.5	**32b**	4
**21f**	500	**29d**	16.12	**32d**	500
**23d**	>500	**29e**	≥500	**32e**	1000
**24e**	500	**29f**	0.5	**32f**	2
**28a**	4	**29g**	≥500	**32g**	4
**28b**	4	**30d**	16.12	**33a**	1
**28c**	0.5	**31a**	8	**33b**	0.5
**28d**	2	**31b**	8	**33c**	2
**28e**	8	**31c**	8	**33f**	1
**28f**	8	**31d**	500	Penicillin ^a^	0.25 (0.25–1.0)
**28g**	8	**31e**	0.5	Tetracycline ^a^	1.00 (0.25–1.0)
**29a**	2	**31f**	0.25		
**29b**	≥500	**32a**	8		

^a^ Penicillin and tetracycline were used as control drugs, MIC range values for this strain are indicated in parenthesis.

**Table 4:** Minimum inhibitory concentrations (MIC) and minimum fungicidal concentrations (MFC) of triazine derivatives with MIC < 250 μg/mL against fungal species ^a^.

Compounds	MIC ^b^ (μg/mL)/MFC (μg/mL) ^c^							
	*Ca*	*Cn*	*Mg*	*Tr*	*Tm*	*Afu*	*Ani*	*Afl*
**17**	>250	>250	>250	**125**	>250	>250	>250	>250
**21d**	>250	>250	>250	**125**	>250	>250	**125**	>250
**23e**	250	250	>250	**125**	>250	>250	>250	>250
**24c**	>250	>250	>250	**125**	>250	>250	>250	>250
**24e**	>250	>250	>250	>250	**125**	>250	>250	>250
**29c**	>250	>250	**125**	>250	>250	>250	>250	>250
**29e**	>250	>250	>250	**62.5**	>250	>250	>250	>250
**29f**	>250	>250	**125**	>250	>250	>250	>250	>250
**29g**	>250	>250	**125**	>250	>250	>250	>250	>250
**30d**	>250	>250	>250	**125**	>250	>250	>250	>250
**30g**	**125**	**125**	>250	250	>250	>250	>250	>250
**31g**	>250	>250	>250	250	**62.5**	**62.5**	>250	>250
Amphotericin B ^d^	0.5	0.25	0.125	0.075	0.075	0.50	0.50	0.50
Terbinafine ^d^	-	-	0.04	0.01	0.025	-	-	-
Fluconazole ^d^	0.03	0.25	-	-	-	-	-	-
Itraconazole ^d^	0.5	-	-	-	-	-	-	-

^a^ MIC: Minimum concentration that inhibits 100% of the growth of the fungi ^b^ MFC: Lowest concentration that produced <3 colonies (approximately 99 to 99.5% lethal activity). ^c^ *Ca: Candida albicans; Cn: Cryptococcus neoformans; Mg: Microsporum gypseum; Tr: Trichophyton rubrum; Tm: Trichophyton mentagrophytes; Afu: Aspergillus fumigatus; Ani: Aspergillus niger; Afl: Aspergillus flavus.* ^d^ First-line drugs used for the treatment of diseases caused by fungi [88]. The data in bold indicate the most notable MICs values.

**Table 5:** Physicochemical properties, ADME, and medicinal chemistry predictions of most active compounds.

Compounds	29b	29e	31b	31f	31g	33a	Expected Value
MW	621.67	697.79	642.09	675.64	751.76	609.08	<500
PSA	174.61	222.73	174.61	174.61	222.73	197.20	<140
HBA	9	10	9	12	13	7	<10
HBD	4	6	4	4	6	7	<5
RB	8	11	8	9	12	9	<10
Log P	4.64	3.38	4.23	4.64	3.89	3.36	0–5
Log S (ESOL)	−6.89	−6.59	−7.19	−7.46	−7.16	−6.38	>−6
Lipinski violations	2	3	2	2	3	3	-
GI absorption	Low	Low	Low	Low	Low	Low	-
BBB permeant	No	No	No	No	No	No	-
Pgp substrate	No	No	No	No	No	No	-
CYP1A2 inhibitor	No	No	No	No	No	No	-
CYP2C19 inhibitor	No	No	No	No	No	No	-
CYP2C9 inhibitor	No	No	No	No	No	No	-
CYP2D6 inhibitor	No	No	No	No	No	No	-
CYP3A4 inhibitor	Yes	Yes	No	No	Yes	Yes	-
Synthetic Accessibility	5.31	5.62	5.18	5.34	5.64	5.03	<6
Bioavailability score	0.17	0.17	0.17	0.17	0.17	0.17	>0.1

**Table 6:** Results of the hemolytic activity for the synthesized triazine derivatives that showed significant biological activity.

Compound	% Hemolysis	Compound	% Hemolysis	Compound	% Hemolysis
**17**	1.6	**24g**	0.8	**31a**	1
**20b**	3.3	**28a**	3	**31b**	1.7
**20d**	1.2	**28b**	5	**31c**	1.2
**21a**	2	**28c**	7	**31d**	0.6
**21b**	13	**28d**	1	**31e**	2.3
**21d**	8	**28e**	5	**31f**	2.4
**21f**	0.8	**28f**	2	**31g**	3
**23a**	0.7	**28g**	100	**32a**	2
**23b**	1.8	**29a**	3	**32b**	1
**23c**	0	**29c**	2.9	**32f**	5
**23d**	0.2	**29d**	75	**32g**	2
**24a**	10.6	**29f**	0	**33a**	8
**24b**	1.2	**30a**	0.6	**33b**	100
**24c**	0.1	**30b**	0.2	**33c**	5
**24d**	2.3	**30d**	11	**33e**	1.2
**24e**	9.1	**30e**	2.7	**33f**	5
**24f**	9.9	**30g**	2.4	**33g**	22

**Table 7:** In silico toxicity studies of compounds **29e**, **29b**, **31b**, **31f**, **31g**, **33a**.

Compound	29b	29e	31b	31f	31g	33a
Hepatotoxicity	Inactive 0.56	Inactive 0.52	Inactive 0.52	Inactive 0.56	Inactive 0.56	Inactive 0.70
Immunotoxicity	Inactive 0.52	Active 0.99	Active 0.99	Active 0.73	Active 0.84	Active 0.57
Carcinogenicity	Active 0.51	Inactive 0.61	Inactive 0.61	Active 0.51	Active 0.51	Inactive 0.62
Mutagenicity	Inactive 0.71	Inactive 0.52	Inactive 0.51	Inactive 0.73	Inactive 0.73	Inactive 0.62
Cytotoxicity	Inactive 0.50	Inactive 0.56	Inactive 0.55	Active 0.50	Active 0.50	Active 0.54
LD_50_(mg/Kg)	3000	900	3000	3000	900	2958
Toxicity class ^a^	5	4	5	5	4	5

^a^ Class I: fatal if swallowed (LD_50_ ≤ 5), II: fatal if swallowed (5 < LD_50_ ≤ 50), III: toxic if swallowed (50 < LD_50_ ≤ 300), IV: harmful if swallowed (300 < LD_50_ ≤ 2000), V: may be harmful if swallowed (2000 < LD_50_ ≤ 5000), VI: non-toxic (LD_50_ > 5000).

## Data Availability

Data available in manuscript and the Supplementary Materials.

## Supplementary Materials

The following supporting information can be downloaded at: https://www.mdpi.com/article/10.3390/ijms25073623/s1.
