# Supplementary material for: Synthesis of Novel Triazine-Based Chalcones and 8,9-dihydro-7H-pyrimido[4,5-b][1,4]diazepines as Potential Leads in the Search of Anticancer, Antibacterial and Antifungal Agents
Source: Int J Mol Sci. 2024 Mar 23;25(7):3623. doi: 10.3390/ijms25073623 (PMC11012124; doi:10.3390/ijms25073623)
Supplement: Supplementary file 1 [file ijms-25-03623-s001.zip › ijms-2744748-supplementary.pdf]

Synthesis of Novel Triazine-Based Chalcones and 8,9-dihydro-7*H*-pyrimido[4,5-*b*][1,4]diazepines as Potential Leads in the Search of Anticancer, Antibacterial and Antifungal Agents

Leydi M. Moreno [1,*], Jairo Quiroga [1], Rodrigo Abonia [1], María del P. Crespo [2,3], Carlos Aranaga [4,5], Luis Martínez-Martínez [6], Maximiliano Sortino [7], Mauricio Barreto [3], María E. Burbano [3] and Braulio Insuasty [1,*]

1 Grupo de Investigación de Compuestos Heterocíclicos, Departamento de Química, Universidad del Valle, Cali 760042, Colombia; jairo.quiroga@correounivalle.edu.co (J.Q.); rodrigo.abonia@correounivalle.edu.co (R.A.)

2 Grupo de Biotecnología e Infecciones Bacterianas, Departamento de Microbiología, Universidad del Valle, Cali 760042, Colombia; maria.crespo.ortiz@correounivalle.edu.co

3 Grupo de Microbiología y Enfermedades Infecciosas, Departamento de Microbiología, Universidad del Va-lle, Cali 760042, Colombia; mauricio.barreto@correounivalle.edu.co (M.B.); maria.e.burbano@correounivalle.edu.co (M.E.B.)

4 Grupo de Investigación en Química y Biotecnología (QUIBIO), Facultad de Ciencias Básicas, Universidad Santiago de Cali, Cali 760035, Colombia; carlos.aranaga00@usc.edu.co

5 Grupo de Investigación Traslacional en Enfermedades Infecciosas, Escuela de Biomedicina, Universidad de Córdoba, 14014 Córdoba, Spain

6 Unidad de Microbiología Clínica, Hospital Universitario Reina Sofía, Instituto Maimónides de Investigación Biomédica de Córdoba (IMIBIC), Departamento de Química Agrícola, Edafología y Microbiología, Universidad de Córdoba, 14004 Córdoba, Spain; luis.martinez.martinez.sspa@juntadeandalucia.es

7 Área de Farmacognosia, Facultad de Ciencias Bioquímicas y Farmacéuticas, Universidad Nacional de Ro-sario, Suipacha 531, Rosario 2000, Argentina; msortino@fbioyf.unr.edu.ar

* Correspondence: leydi.moreno@correounivalle.edu.co (L.M.M.); braulio.insuasty@correounivalle.edu.co (B.I.)

Supporting Information

Characterization of monosubstituted triazines (**5-7**) S2
Characterization of disubstituted triazines (**8-11**) S2
Characterization of trisubstituted triazines (**12-15**, **17-18**) S3
Characterization of triazinyloxy-chalcones (**20a-g**) S5
Characterization of triazinyloxy-chalcones (**21a-g**) S8
Characterization of triazinylamino-chalcones (**23a-g**) S10
Characterization of triazinylamino-chalcones (**24a-g**) S13
Characterization of triazinyloxy-diazepines (**28a-g**) S16
Characterization of triazinyloxy-diazepines (**29a-g**) S18
Characterization of triazinylamino-diazepines (**30a-g**) S22
Characterization of triazinylamino-diazepines (**31a-g**) S25
Characterization of triazinylamino-diazepines (**32a-g**) S29
Characterization of triazinylamino-diazepines (**33a-g**) S31
NMR spectra of synthetized compounds S35
In vitro anticancer activity expressed as GI_{50} and LC_{50} (μM) S112
References S122

Characterization of monosubstituted triazines (5-7)

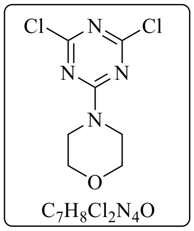

4-(4,6-dichloro-1,3,5-triazin-2-yl)morpholine (***5***)[1–3]. White solid. 88% yield; mp 161-162 °C. FT-IR (ATR): ν (cm^{-1}) 2970-2862 (C-H), 1578 (C=N), 1229 and 1158 (C-O). ^1H NMR (400 MHz, DMSO-d_6) δ ppm 3.63 (t, J = 4.0 Hz, 4H, CH_2), 3.75 (t, J = 4.0 Hz, 4H, CH_2). ^{13}C NMR (100 MHz, DMSO-d_6) δ ppm 44.8 (CH_2), 46.0 (CH_2), 164.0 (C), 169.6 (C). MS (70 eV) m/z (%): 234: 236: 238 [M^+]:[M + 2]$^+$:[M + 4]$^+$ (26/16/3), 219 (30), 189 (41), 177 (47), 149 (99), 87 (100).

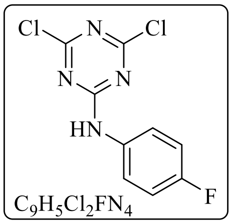

4,6-dichloro-N-(4-fluorophenyl)-1,3,5-triazin-2-amine (***6***)[4,5]. Beige solid. 97% yield; mp 180-182 °C. FT-IR (ATR): ν (cm^{-1}) 3387 (N-H), 3119 (=C-H), 1562 and 1502 (C=N and C=C). ^1H NMR (400 MHz, DMSO-d_6) δ ppm 7.24 (t, J = 8.9 Hz, 2H, Ar-H), 7.56-7.64 (m, 2H, Ar-H), 11.14 (bs, 1H, NH). ^{13}C NMR (100 MHz, DMSO-d_6) δ ppm 115.7 (d, $^2J_{CF}$ = 22.1 Hz, CH), 123.6 (d, $^3J_{CF}$ = 8.5 Hz, CH), 133.1 (C), 150.0 (C), 154.3 (C), 159.12 (d, $^1J_{CF}$ = 239.3 Hz). MS (70 eV) *m/z* (%): 258:260:262 [M^+]:[M + 2]$^+$:[M + 4]$^+$ (100/61/11), 223 (33), 162 (54), 136 (89), 109 (49), 95 (71).

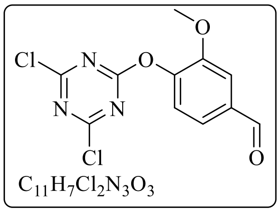

4-((4,6-dichloro-1,3,5-triazin-2-yl)oxy)-3-methoxybenzaldehyde (***7***). White solid. 96% yield; mp >250 °C. FT-IR (ATR) ν (cm^{-1}) 1690 (C=O), 1524 (C=N), 1273 and 1147 (C-O). ^1H NMR (400 MHz, DMSO-d_6) δ ppm 3.87 (s, 3H, OCH_3), 7.50 (d, J = 8.1 Hz, 1H, Ar-H), 7.59-7.62 (m, 1H, Ar-H), 7.65 (d, J = 1.7 Hz, 1H, Ar-H), 9.99 (s, 1H, CHO). ^{13}C NMR (100 MHz, DMSO-d_6) δ ppm 56.4 (CH_3), 112.6 (CH), 123.5 (CH), 123.9 (CH), 135.7 (C), 143.7 (C), 150.0 (C), 151.4 (C), 161.1 (C), 192.1 (C). MS (70 eV) *m/z* (%): 299:301:303 [M^+]:[M + 2]$^+$:[M + 4]$^+$ (27/18/3), 264 (100), 228 (10), 207 (10), 119 (25), 87 (39). Anal. Calcd. for $C_{11}H_7Cl_2N_3O_3$: C, 44.03; H, 2.35; N, 14.00; Found: C, 43.99; H, 2.31; N, 14.02.

Characterization of disubstituted triazines (8-11)

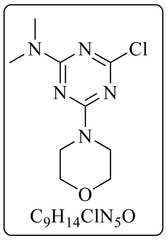

4-chloro-N,N-dimethyl-6-morpholino-1,3,5-triazin-2-amine (***8***)[6]. White solid. 90% yield; mp 92-94 °C. FT-IR (ATR): ν (cm^{-1}) 2949-2854 (C-H), 1561 (C=N), 1242 and 1198 (C-O). ^1H NMR (400 MHz, $CDCl_3$) δ ppm 3.09 (s, 3H, CH_3), 3.13 (s, 3H, CH_3), 3.69 (t, J = 4.5 Hz, 4H, CH_2), 3.77 (t, J = 4.5 Hz, 4H, CH_2). ^{13}C NMR (100 MHz, $CDCl_3$) δ ppm 36.5 (CH_3), 43.9 (CH_2), 66.8 (CH_2), 164.5 (C), 165.1 (C), 169.4 (C). MS (70 eV) m/z (%): 243: 245 [M^+]:[M + 2]$^+$ (94/32), 228 (27), 212 (86), 198 (100), 170 (33).

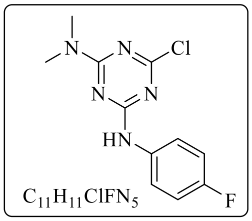

6-chloro-N^2-(4-fluorophenyl)-N^4,N^4-dimethyl-1,3,5-triazine-2,4-diamine (***9***)[4]. White solid. 77% yield; mp 130-133 °C. FT-IR (ATR): ν (cm^{-1}) 3346 (N-H), 2924 (C-H), 1533 and 1494 (C=N and C=C). ^1H NMR (400 MHz, DMSO-d_6) δ ppm 3.06-3.17 (m, 6H, CH_3), 7.14 (t, J = 8.7 Hz, 2H, Ar-H), 7.61-7.79 (m, 2H, Ar-H), 10.04 (bs, 1H, NH). ^{13}C NMR (100 MHz, DMSO-d_6) δ ppm 36.1 (CH_3), 36.4 (CH_3), 115.1 (d, $^2J_{CF}$ = 22.3 Hz, CH), 121.68 (d, J = 11.0 Hz, CH), 135.3 (C), 157.9 (d, $^1J_{CF}$ = 240.6 Hz, C), 163.1 (C), 164.6 (C), 168.0 (C). MS (70 eV) m/z (%): 267: 269 [M^+]:[M + 2]$^+$ (100/34), 252 (25), 238 (17), 232 (15), 223 (15), 162 (23), 136 (30).

*4-((4-chloro-6-((4-fluorophenyl)amino)-1,3,5-triazin-2-yl)oxy)-3-methoxybenzaldehyde (**10**).* Beige solid. 91% yield; mp 185-188 °C. FT-IR (ATR): ν (cm^{-1}) 3278 (N-H), 1678 (C=O), 1562 (C=N), 1508 (C=C), 1283 and 1152 (C-O). ^{1}H NMR (400 MHz, DMSO-d_6) δ ppm 3.84 (s, 3H, OCH_3), 6.99 (t, J = 8.7 Hz, 2H, Ar-H), 7.37 (dd, J = 8.5, 4.9 Hz, 2H, Ar-H), 7.52 (t, J = 6.7 Hz, 1H, Ar-H), 7.64 (d, J = 8.2 Hz, 1H, Ar-H), 7.70 (bs, 1H, Ar-H), 10.02 (s, 1H, CHO), 10.88 (bs, 1H, NH). ^{13}C NMR (100 MHz, DMSO-d_6) δ ppm 56.2 (CH_3), 112.3 (CH), 115.12 (d, $^2J_{CF}$ = 22.5 Hz, CH), 122.3 (d, $^3J_{CF}$ = 8.0 Hz, CH), 123.5 (CH), 123.7 (CH), 133.8 (d, $^4J_{CF}$ = 2.0 Hz, C), 135.4 (C), 145.0 (C), 151.5 (C), 158.5 (d, $^1J_{CF}$ = 241.4 Hz, C), 164.6 (C), 170.0 (C), 170.2 (C), 192.1 (C). MS (70 eV) *m/z* (%): 374:376 [M^+]:[M + 2]$^+$ (30/10), 343 (45), 339 (100), 282 (18), 264 (14), 203 (27), 163 (91), 135 (30). Anal. Calcd. for $C_{17}H_{12}ClFN_4O_3$: C, 54.49; H, 3.23; N, 14.95; Found: C, 54.50; H, 3.19; N, 14.80.

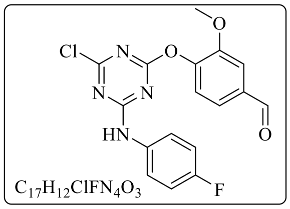

*4-((4-chloro-6-morpholino-1,3,5-triazin-2-yl)oxy)-3-methoxybenzaldehyde (**11**)*[7]. White solid. 88% yield; mp 193-195 °C. FT-IR (ATR) ν (cm^{-1}), 1690 (C=O), 1582 (C=N), 1492 (C=C), 1265 and 1159 (C-O). ^{1}H NMR (400 MHz, DMSO-d_6) δ ppm 3.53-3.61 (m, 4H, CH_2), 3.64 (t, J = 4.0 Hz, 2H, CH_2), 3.74 (t, J = 4.0 Hz, 2H, CH_2), 3.84 (s, 3H, OCH_3), 7.47 (d, J = 8.1 Hz, 1H, Ar-H), 7.60-7.66 (m, 2H, Ar-H), 9.99 (s, 1H, CHO). ^{13}C NMR (100 MHz, DMSO-d_6) δ ppm 43.8 (CH_2), 44.1 (CH_2), 56.2 (CH_3), 65.4 (CH_2), 65.6 (CH_2), 112.3 (CH), 123.3 (CH), 123.7 (CH), 135.1 (C), 144.8 (C), 151.5 (C), 164.8 (C), 169.6 (C), 170.2 (C), 192.0 (C). MS (70 eV) *m/z* (%): 350:352 [M^+]:[M + 2]$^+$ (11/4), 319 (39), 315 (100), 185 (4), 119 (12), 77 (13).

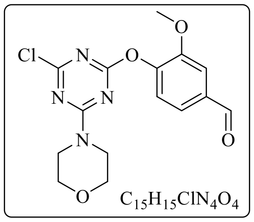

Characterization of trisubstituted triazines (12-15, 17-18)

*N^2-(2-aminoethyl)-N^4,N^4-dimethyl-6-morpholino-1,3,5-triazine-2,4-diamine (**12**).* White oil. 78% yield. FT-IR (ATR): ν (cm^{-1}) 3384 and 3337 (N-H), 2966 (C-H), 1525 (C=N), 1242 and 1113 (C-O). ^{1}H NMR (400 MHz, $CDCl_3$) δ ppm 1.73 (bs, 2H, NH_2), 2.84 (t, J = 5.9 Hz, 2H, CH_2), 3.05 (s, 6H, CH_3), 3.41 (q, J = 5.8 Hz, 2H, CH_2), 3.63-3.76 (m, 8H, CH_2), 5.09 (bs, 1H, NH). ^{13}C NMR (100 MHz, $CDCl_3$) δ ppm 36.0 (CH_3), 42.1 (CH_2), 43.7 (CH_2), 43.8 (CH_2), 67.0 (CH_2), 165.3 (C), 165.8 (C), 166.7 (C). MS (70 eV) m/z (%): 267 (3), 250 (5), 237 (73), 225 (100), 193 (30), 138 (44). Anal. Calcd. for $C_{11}H_{21}N_7O$: C, 49.42; H, 7.92; N, 36.68; Found: C, 49.40; H, 7.95; N, 36.59.

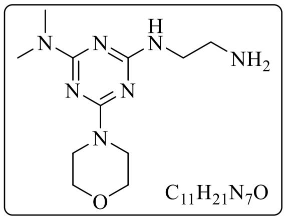

*6-chloro-N^2-(4-fluorophenyl)-N^4,N^4-dimethyl-1,3,5-triazine-2,4-diamine (**13**).* White solid. 73% yield; mp 160-162 °C. FT-IR (ATR): ν (cm^{-1}) 3350 and 3267 (N-H), 2951 (C-H), 1529 and 1500 (C=N and C=C). ^{1}H NMR (400 MHz, $CDCl_3$) δ ppm 1.58 (bs, 2H, NH_2), 2.88 (t, J = 5.7 Hz, 2H, CH_2), 3.11 (bs, 6H, CH_3), 3.40-3.50 (m, 2H, CH_2), 6.12 (bs, 1H, NH), 6.95 (t, J = 8.5 Hz, 2H, Ar-H), 7.42-7.80 (m, 3H, Ar-H, NH). ^{13}C NMR (100 MHz, $CDCl_3$) δ ppm 36.3 (CH_3), 41.8 (CH_2), 43.6 (CH_2), 115.3 (d, $^2J_{CF}$ = 21.7 Hz, CH), 121.5 (d, $^3J_{CF}$ = 11.4 Hz, CH), 135.9 (C), 158.4 (d, $^4J_{CF}$ = 238.4 Hz, C), 164.3

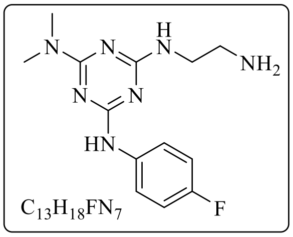

(C), 165.8 (C), 166.4 (C). MS (70 eV) m/z (%): 291 (8), 261 (66), 249 (100), 233 (24), 162 (16), 137 (15). Anal. Calcd. for $C_{13}H_{18}FN_7$: C, 53.60; H, 6.23; N, 33.66; Found: C, 53.57; H, 6.14; N, 33.62.

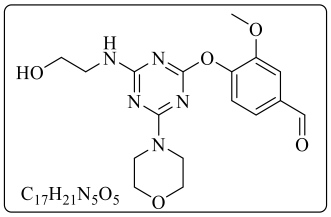

*4-((4-((2-hydroxyethyl)amino)-6-morpholino-1,3,5-triazin-2-yl)oxy)-3-methoxybenzaldehyde (**14**).* White solid. 86% yield; mp 183-185 °C. FT-IR (ATR) ν (cm^{-1}) The signal is not observed (NH), 3335 (OH), 2969 (C-H), 1650 (C=O), 1593 (C=N), 1269 and 1164 (C-O). ^{1}H NMR (400 MHz, DMSO-d_6) δ ppm 3.09-3.17 (m, 1H, CH_2), 3.24-3.31 (m, 1H, CH_2), 3.33-3.40 (m, 1H, CH_2), 3.41-3.58 (m, 9H, CH_2), 3.80 (s, 3H, OCH_3), 4.71 (bs, 1H, OH), 7.13-7.25 (m, 1H, Ar-H), 7.33 (t, J = 6.8 Hz, 1H, Ar-H), 7.51-7.60 (m, 2H, Ar-H, NH), 9.93 (s, 1H, CHO). ^{13}C NMR (100 MHz, DMSO-d_6) δ ppm 43.3 (CH_2), 43.6 (CH_2), 56.2 (CH_3), 59.7 (CH_2), 66.1 (CH_2), 112.3 (CH), 124.0 (CH), 124.0 (CH), 134.7 (C), 146.3 (C), 152.3 (C), 165.9 (C), 166.9 (C), 170.1 (C), 192.4 (C). MS (70 eV) *m/z* (%): 375 (27), 344 (100), 330 (20), 318 (17), 286 (9), 151 (13). Anal. Calcd. for $C_{17}H_{21}N_5O_5$: C, 54.39; H, 5.64; N, 18.66; Found: C, 54.35; H, 5.59; N, 18.59.

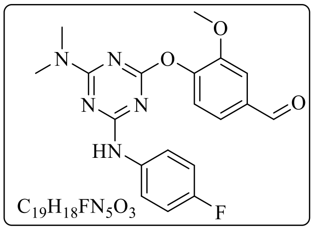

*4-((4-(dimethylamino)-6-((4-fluorophenyl)amino)-1,3,5-triazin-2-yl)oxy)-3-methoxybenzaldehyde (**15**).* Beige solid. 83% yield; mp 175-177 °C. FT-IR (ATR): ν (cm^{-1}) 3275 (N-H), 3119 (=C-H), 1709 (C=O), 1552 (C=N), 1504 (C=C), 1263 and 1147 (C-O). ^{1}H NMR (400 MHz, DMSO-d_6) δ ppm 2.99 (s, 3H, CH_3), 3.11 (s, 3H, CH_3), 3.83 (s, 3H, OCH_3), 6.71-7.23 (m, 2H, Ar-H), 7.43 (d, J = 7.97 Hz, 2H, Ar-H), 7.57-7.78 (m, 3H, Ar-H), 9.61 (bs, 1H, NH), 10.00 (s, 1H, CHO). ^{13}C NMR (100 MHz, DMSO-d_6) δ ppm 35.7 (CH_3), 36.1 (CH_3), 56.0 (CH_3), 111.9 (CH), 114.8 (d, $^2J_{CF}$ = 20.0 Hz, CH), 121.4 (d, $^3J_{CF}$ = 7.8 Hz, CH), 123.8 (CH), 123.9 (CH), 134.6 (C), 135.9 (C), 146.1 (C), 152.1 (C), 157.4 (d, $^1J_{CF}$ = 239.0 Hz, C), 164.6 (C), 166.2 (C), 169.8 (C), 192.1 (C). MS (70 eV) m/z (%): 383 (77), 352 (37), 273 (100), 233 (9), 216 (15), 163 (21). Anal. Calcd. for $C_{19}H_{18}FN_5O_3$: C, 59.53; H, 4.73; N, 18.27; Found: C, 59.57; H, 4.69; N, 18.30.

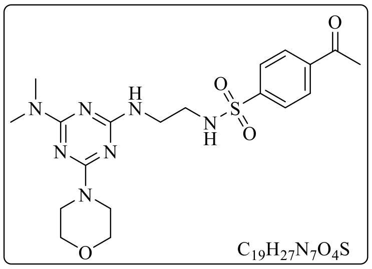

*4-acetyl-N-(2-((4-(dimethylamino)-6-morpholino-1,3,5-triazin-2-yl)amino)ethyl)benzenesulfonamide (**17**).* White oil. 80% yield. FT-IR (ATR): ν (cm^{-1}) 3396 (N-H), 2957 (C-H), 1687 (C=O), 1524 (C=N), 1157 (S=O). ^{1}H NMR (400 MHz, $CDCl_3$) δ ppm 2.62 (s, 3H, CH_3), 2.99-3.22 (m, 8H, CH_3, CH_2), 3.40-3.52 (m, 2H, CH_2), 3.71 (bs, 9H, CH_2, NH), 5.07 (bs, 1H, NH), 7.26 (sa, 1H, NH), 7.80 (d, J = 8.1 Hz, 2H, Ar-H), 7.97 (d, J = 8.1 Hz, 2H, Ar-H). ^{13}C NMR (100 MHz, $CDCl_3$) δ ppm 26.8 (CH_3), 36.3 (CH_3), 40.2 (CH_2), 43.6 (CH_2), 43.7 (CH_2), 45.9 (CH_2), 127.2 (CH), 128.8 (CH), 134.5 (C), 139.7 (C), 143.9 (C), 164.4 (C), 164.7 (C), 196.8 (C). MS (70 eV) m/z (%): 449 (80), 419 (19), 404 (16), 266 (100), 237 (100). Anal. Calcd. for $C_{19}H_{27}N_7O_4S$: C, 50.77; H, 6.05; N, 21.81; S, 7.13 Found: C, 50.72; H, 5.99; N, 21.83; S, 7.09.

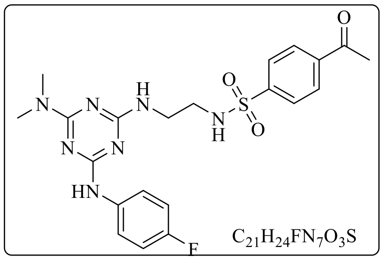

4-acetyl-N-(2-((4-(dimethylamino)-6-((4-fluorophenyl)amino)-1,3,5-triazin-2-yl)amino)ethyl)benzenesulfonamide (***18***). White oil. 75% yield. FT-IR (ATR): ν (cm^{-1}) 3377 (N-H), 2987 (C-H), 1687 (C=O), 1585 (C=N), 1208 (S=O). ^{1}H NMR (400 MHz, $CDCl_3$) δ ppm 2.03 (bs, 1H, NH), 2.55 (s, 3H, CH_3), 3.10 (bs, 6H, CH_3), 3.15-3.25 (m, 2H, CH_2), 3.38-3.54 (m, 2H, CH_2), 5.34 (bs, 1H, NH), 6.95 (t, J = 8.3 Hz, 2H, Ar-H), 7.22 (bs, 1H, NH), 7.47 (dd, J = 7.6, 4.6 Hz, 2H, Ar-H), 7.83 (d, J = 8.2 Hz, 2H, Ar-H), 7.95 (d, J = 8.2 Hz, 2H, Ar-H). ^{13}C NMR (100 MHz, $CDCl_3$) δ ppm 26.9 (CH_3), 36.5 (CH_3), 40.4 (CH_2), 45.1 (CH_2), 115.4 (d, $^2J_{CF}$ = 22.3 Hz, CH), 121.7 (d, $^3J_{CF}$ = 9.0 Hz, CH), 127.2 (CH), 128.9 (CH), 135.3 (C), 139.8 (C), 144.0 (C), 158.6 (d, $^1J_{CF}$ = 242.0 Hz, C), 163.7 (C), 165.3 (C), 166.4 (C), 197.0 (C). MS (70 eV) m/z (%): 473 (14), 290 (74), 261 (100), 233 (14), 183 (10), 137 (22). Anal. Calcd. for $C_{21}H_{24}FN_7O_3S$: C, 53.27; H, 5.11; N, 20.71; S, 6.77 Found: C, 53.13; H, 5.10; N, 20.68; S, 6.75.

Characterization of triazinyloxy-chalcones (20a-g)

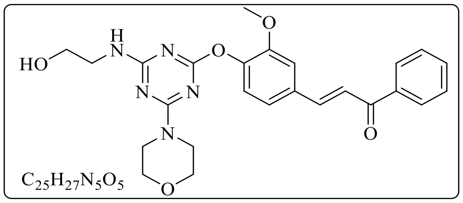

*(E)-3-(4-((4-((2-hydroxyethyl)amino)-6-morpholino-1,3,5-triazin-2-yl)oxy)-3-methoxyphenyl)-1-phenylprop-2-en-1-one (**20a**)*. Beige solid. 83% yield; mp 178-180 °C. FT-IR (ATR): ν (cm^{-1}) 3439 (N-H), 3269 (O-H), 3138 (=C-H), 1662 (C=O), 1597 and 1500 (C=N and C=C). ^{1}H NMR (400 MHz, DMSO-d_6) δ ppm 3.10-3.20 (m, 1H, CH_2), 3.24-3.30 (m, 1H, CH_2), 3.41-3.49 (m, 2H, CH_2), 3.51-3.73 (m, 8H, CH_2), 3.84 (s, 3H, OCH_3), 4.60 (bs, 1H, OH), 7.19 (t, J = 7.6 Hz, 1H, Ar-H), 7.27 (t, J = 5.4 Hz, 1H, Ar-H), 7.46 (d, J = 7.6 Hz, 1H, Ar-H), 7.59 (t, J = 8.1 Hz, 2H, Ar-H), 7.64-7.71 (m, 2H, Ar-H, NH), 7.75 (d, J = 15.6 Hz, 1H, CH), 7.93 (d, J = 15.6 Hz, 1H, CH), 8.17 (d, J = 7.6 Hz, 2H, Ar-H). ^{13}C NMR (100 MHz, DMSO-d_6) δ ppm 43.1 (CH_2), 43.3 (CH_2), 56.1 (CH_3), 59.4 (CH_2), 65.9 (CH_2), 112.6 (CH), 121.8 (CH), 122.4 (CH), 123.4 (CH), 128.5 (CH), 128.8 (CH), 132.9 (C), 133.1 (CH), 137.6 (C), 143.0 (CH), 143.8 (C), 151.7 (C), 165.8 (C), 166.6 (C), 170.1 (C), 189.2 (C). MS (70 eV) *m/z* (%): 477 (66), 446 (92), 417 (10), 391 (9), 253 (42), 105 (100). Anal. Calcd. for $C_{25}H_{27}N_5O_5$: C, 62.88; H, 5.70; N, 14.67; Found: C, 62.79; H, 5.65; N, 14.70.

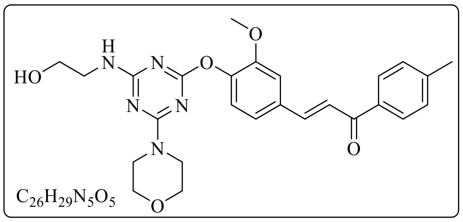

*(E)-3-(4-((4-((2-hydroxyethyl)amino)-6-morpholino-1,3,5-triazin-2-yl)oxy)-3-methoxyphenyl)-1-(p-tolyl)prop-2-en-1-one (**20b**)*. Beige solid. 85% yield; mp 206-208 °C. FT-IR (ATR): ν (cm^{-1}) 3457 (N-H), 3263 (O-H), 3139 (=C-H), 1659 (C=O), 1594 and 1504 (C=N and C=C). ^{1}H NMR (400 MHz, DMSO-d_6) δ ppm 2.41 (s, 3H, CH_3), 3.10-3.21 (m, 1H, CH_2), 3.23-3.31 (m, 1H, CH_2), 3.42-3.49 (m, 2H, CH_2), 3.49-3.70 (m, 8H, CH_2), 3.84 (s, 3H, OCH_3), 4.59 (bs, 1H, OH), 7.18 (t, J = 7.6 Hz, 1H, Ar-H), 7.26 (t, J = 5.4 Hz, 1H, Ar-H), 7.39 (d, J = 7.9 Hz, 2H, Ar-H), 7.44 (d, J = 7.6 Hz, 1H), 7.66 (bs, 1H, NH), 7.73 (d, J = 15.3 Hz, 1H, CH), 7.92 (d, J = 15.3 Hz, 1H, CH), 8.08 (d, J = 7.9 Hz, 2H, Ar-H). ^{13}C NMR (100 MHz, DMSO-d_6) δ ppm 21.2 (CH_3), 43.1 (CH_2), 43.3 (CH_2), 56.1 (CH_3), 59.4 (CH_2), 65.9 (CH_2), 112.5 (CH), 121.8 (CH), 122.3 (CH), 123.4 (CH), 128.7 (CH), 129.3 (CH), 133.0 (C), 135.1 (C), 143.0 (CH), 143.4 (C), 143.6 (C), 151.7 (C), 165.8 (C), 166.6 (C), 170.1 (C), 188.6 (C). MS (70

eV) *m/z* (%): 491 (16), 460 (21), 375 (15), 344 (64), 267 (27), 119 (100). Anal. Calcd. for $C_{26}H_{29}N_5O_5$: C, 63.53; H, 5.95; N, 14.25; Found: C, 63.49; H, 5.97; N, 14.23.

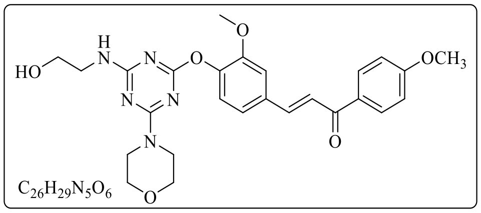

*(E)-3-(4-((4-((2-hydroxyethyl)amino)-6-morpholino-1,3,5-triazin-2-yl)oxy)-3-methoxyphenyl)-1-(4-methoxyphenyl)prop-2-en-1-one (**20c**)*. Beige solid. 65% yield; mp 199-200 °C. FT-IR (ATR): ν (cm^{-1}) 3464 (N-H), 3258 (O-H), 3119 (=C-H), 1656 (C=O), 1591 and 1501 (C=N and C=C). ^{1}H NMR (400 MHz, DMSO-d_6) δ ppm 3.11-3.21 (m, 1H, CH_2), 3.24-3.31 (m, 1H, CH_2), 3.41-3.49 (m, 2H, CH_2), 3.51-3.72 (m, 8H, CH_2), 3.84 (s, 3H, OCH_3), 3.87 (s, 3H, OCH_3), 4.59 (bs, 1H, OH), 7.10 (d, *J* = 8.7 Hz, 2H, Ar-H), 7.18 (t, *J* = 7.6 Hz, 1H, Ar-H), 7.26 (t, *J* = 5.5 Hz, 1H, Ar-H), 7.44 (d, *J* = 7.6 Hz, 1H, Ar-H), 7.65 (bs, 1H, NH), 7.71 (d, *J* = 15.5 Hz, 1H, CH), 7.93 (d, *J* = 15.5 Hz, 1H, CH), 8.18 (d, *J* = 8.7 Hz, 2H, Ar-H). ^{13}C NMR (100 MHz, DMSO-d_6) δ ppm 43.1 (CH_2), 43.3 (CH_2), 55.6 (CH_3), 56.1 (CH_3), 59.4 (CH_2), 65.9 (CH_2), 112.5 (CH), 114.0 (CH), 121.8 (CH), 122.2 (CH), 123.4 (CH), 130.5 (C), 130.9 (CH), 133.0 (C), 142.9 (CH), 151.7 (C), 163.2 (C), 165.8 (C), 166.6 (C), 167.1 (C), 170.1 (C), 187.4 (C). MS (70 eV) *m/z* (%): 507 (33), 476 (40), 450 (9), 284 (83), 253 (17), 135 (100). Anal. Calcd. for $C_{26}H_{29}N_5O_6$: C, 61.53; H, 5.76; N, 13.80; Found: C, 61.52; H, 5.80; N, 13.76.

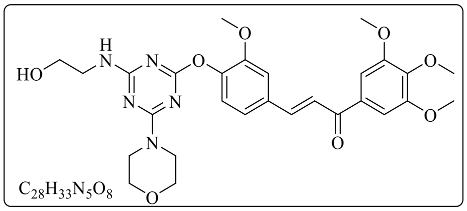

*(E)-3-(4-((4-((2-hydroxyethyl)amino)-6-morpholino-1,3,5-triazin-2-yl)oxy)-3-methoxyphenyl)-1-(3,4,5-trimethoxyphenyl)prop-2-en-1-one (**20d**)*. Yellow solid. 63% yield; mp 193-195 °C. FT-IR (ATR): ν (cm^{-1}) 3439 (N-H), 3291 (O-H), 3187 (=C-H), 1650 (C=O), 1583 and 1502 (C=N and C=C). ^{1}H NMR (400 MHz, DMSO-d_6) δ ppm 3.11-3.20 (m, 1H, CH_2), 3.24-3.30 (m, 1H, CH_2), 3.40-3.49 (m, 2H, CH_2), 3.59 (s, 8H, CH_2), 3.78 (s, 3H, OCH_3), 3.83 (s, 3H, OCH_3), 3.91 (s, 6H, OCH_3), 4.58 (bs, 1H, OH), 7.20 (t, *J* = 7.8 Hz, 1H, Ar-H), 7.25 (t, *J* = 4.5 Hz, 1H, Ar-H), 7.42 (s, 2H, Ar-H), 7.50-7.56 (m, 1H, Ar-H), 7.62 (bs, 1H, NH), 7.75 (d, *J* = 15.5 Hz, 1H, CH), 7.90 (d, *J* = 15.5 Hz, 1H, C H). ^{13}C NMR (100 MHz, DMSO-d_6) δ ppm 43.1 (CH_2), 43.3 (CH_2), 56.1 (CH_3), 56.3 (CH_3), 59.4 (CH_2), 60.2 (CH_3), 65.9 (CH_2), 105.8 (CH), 106.3 (CH), 113.4 (CH), 121.9 (CH), 123.4 (CH), 133.0 (C), 142.1 (C), 142.9 (C), 143.6 (CH), 151.6 (C), 152.7 (C), 152.9 (C), 165.8 (C), 166.6 (C), 170.1 (C), 188.0 (C).MS (70 eV) *m/z* (%): 567 (11), 536 (10), 344 (100), 329 (56), 195 (26). Anal. Calcd. for $C_{28}H_{33}N_5O_8$: C, 59.25; H, 5.86; N, 12.34; Found: C, 59.30; H, 5.79; N, 12.29.

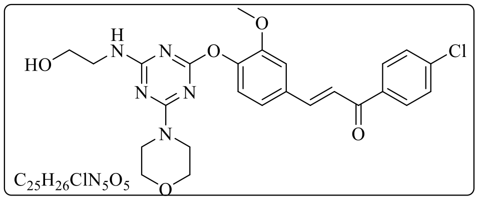

*(E)-1-(4-chlorophenyl)-3-(4-((4-((2-hydroxyethyl)amino)-6-morpholino-1,3,5-triazin-2-yl)oxy)-3-methoxyphenyl)prop-2-en-1-one (**20e**)*. Beige solid. 80% yield; mp 218-220 °C. FT-IR (ATR): ν (cm^{-1}) 3468 (N-H), 3263 (O-H), 3132 (=C-H), 1663 (C=O), 1597 and 1504 (C=N and C=C). ^{1}H NMR (400 MHz, DMSO-d_6) δ ppm 3.12-3.20 (m, 1H, CH_2), 3.21-3.30 (m, 1H, CH_2), 3.41-3.49 (m, 2H, CH_2), 3.49-3.70 (m, 8H, CH_2), 3.83 (s, 3H, OCH_3), 4.59 (bs, 1H, OH), 7.21 (t, *J* = 7.5 Hz, 1H, Ar-H), 7.26 (t, *J* = 6.0 Hz, 1H, Ar-H), 7.46 (d, *J* = 7.5 Hz, 1H, Ar-H), 7.65 (d, *J* = 8.2 Hz, 3H, Ar-H, NH), 7.76 (d, *J* = 15.5 Hz,

1H, CH), 7.92 (d, J = 15.5 Hz, 1H, CH), 8.19 (d, J = 8.2 Hz, 2H, Ar-H). ^{13}C NMR (100 MHz, DMSO-d_6) δ ppm 43.1 (CH_2), 43.3 (CH_2), 56.1 (CH_3), 59.4 (CH_2), 65.9 (CH_2), 112.6 (CH), 121.5 (CH), 122.5 (CH), 123.4 (CH), 128.9 (CH), 130.5 (CH), 132.8 (C), 136.3 (C), 138.1 (C), 143.2 (C), 144.3 (CH), 151.7 (C), 165.8 (C), 166.6 (C), 170.1 (C), 188.1 (C). MS (70 eV) m/z (%): 511:513 [M^+]:[M + 2]$^+$ (39/15), 480 (100), 452 (18), 425 (48), 287 (38), 139 (32). Anal. Calcd. for $C_{25}H_{26}ClN_5O_5$: C, 58.65; H, 5.12; N, 13.68; Found: C, 58.60; H, 5.09; N, 13.71.

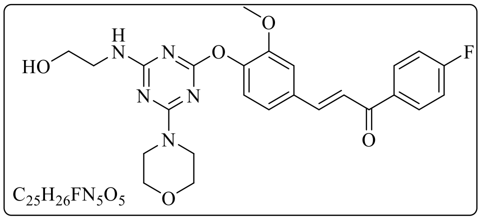

*(E)-1-(4-fluorophenyl)-3-(4-((4-((2-hydroxyethyl)amino)-6-morpholino-1,3,5-triazin-2-yl)oxy)-3-methoxyphenyl)prop-2-en-1-one **(20f)**.* Yellow solid. 78% yield; mp 193-195 °C. FT-IR (ATR): ν (cm^{-1}) Not observed (N-H), 3306 (O-H), 3125 (=C-H), 1662 (C=O), 1596 and 1504 (C=N and C=C). ^{1}H NMR (400 MHz, DMSO-d_6) δ ppm 3.11-3.19 (m, 1H, CH_2), 3.22-3.30 (m, 1H, CH_2), 3.43-3.48 (m, 2H, CH_2), 3.49-3.68 (m, 8H, CH_2), 3.83 (s, 3H, OCH_3), 4.60 (bs, 1H, OH), 7.17 (d, J = 8.0 Hz, 1H, Ar-H), 7.22-7.28 (m, 1H, Ar-H), 7.35-7.49 (m, 3H, Ar-H), 7.66 (bs, 1H, NH), 7.74 (d, J = 15.5 Hz, 1H, CH), 7.93 (d, J = 15.5 Hz, 1H, CH), 8.22-8.30 (m, 2H, Ar-H). ^{13}C NMR (100 MHz, DMSO-d_6) δ ppm 43.1 (CH_2), 43.3 (CH_2), 56.1 (CH_3), 59.4 (CH_2), 65.9 (CH_2), 112.6 (CH), 115.8 (d, $^2J_{CF}$ = 22.0 Hz, CH) , 121.6 (CH), 122.5 (CH), 123.5 (CH), 131.6 (d, $^3J_{CF}$ = 9.1 Hz, CH), 132.9 (C), 134.3 (C), 143.1 (C), 144.0 (C), 151.8 (CH), 163.9 (C), 166.4 (C), 166.7 (C), 170.1 (C), 187.8 (C). MS (70 eV) m/z (%): 495 (30), 464 (45), 272 (86), 255 (18), 183 (26), 123 (100). Anal. Calcd. for $C_{25}H_{26}FN_5O_5$: C, 60.60; H, 5.29; N, 14.13; Found: C, 60.58; H, 5.25; N, 14.08.

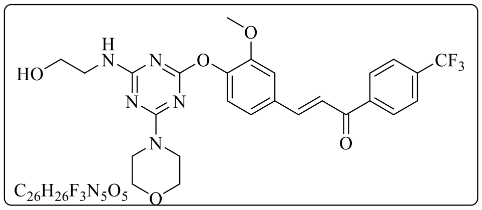

*(E)-3-(4-((4-((2-hydroxyethyl)amino)-6-morpholino-1,3,5-triazin-2-yl)oxy)-3-methoxyphenyl)-1-(4-(trifluoromethyl)phenyl)prop-2-en-1-one **(20g)**.* Beige solid. 75% yield; mp 188-189 °C. FT-IR (ATR): ν (cm^{-1}) Not observed(N-H), 3306 (O-H), 3119 (=C-H), 1666 (C=O), 1597 and 1501 (C=N and C=C). ^{1}H NMR (400 MHz, DMSO-d_6) δ ppm 3.11-3.22 (m, 1H, CH_2), 3.24-3.30 (m, 1H, CH_2), 3.38-3.48 (m, 2H, CH_2), 3.50-3.70 (m, 8H, CH_2), 3.83 (s, 3H, OCH_3), 4.60 (bs, 1H, OH), 7.20 (t, J = 7.1 Hz, 1H, Ar-H), 7.23-7.29 (m, 1H, Ar-H), 7.48 (d, J = 7.8 Hz, 1H, Ar-H), 7.68 (bs, 1H, NH), 7.79 (d, J = 15.6 Hz, 1H, CH), 7.88-7.98 (m, 3H, Ar-H, CH), 8.33 (d, J = 8.0 Hz, 2H Ar-H). ^{13}C NMR (100 MHz, DMSO-d_6) δ ppm 43.6 (CH_2), 43.8 (CH_2), 56.6 (CH_3), 59.9 (CH_2), 66.4 (CH_2), 113.3 (CH), 122.2 (CH), 123.0 (d, $^3J_{CF}$ = 11.6 Hz, CH), 124.0 (CH), 124,7 (d, d, $^2J_{CF}$ = 201.3 Hz, C) 126.2 (d, $^4J_{CF}$ = 4.0 Hz, CH), 126.2 (C), 129.8 (CH), 133.2 (C), 142.6 (q, $^1J_{CF}$ = 240.5 Hz, CF_3), 143.8 (C), 145.4 (CH), 152.3 (C), 166.3 (C), 167.2 (C), 170.6 (C), 189.3 (C). MS (70 eV) m/z (%): 545 (38), 514 (63), 344 (71), 321 (27), 173 (74), 145 (75). Anal. Calcd. for $C_{26}H_{26}F_3N_5O_5$: C, 57.25; H, 4.80; N, 12.84; Found: C, 57.20; H, 4.78; N, 12.80.

Characterization of triazinyloxy-chalcones (21a-g)

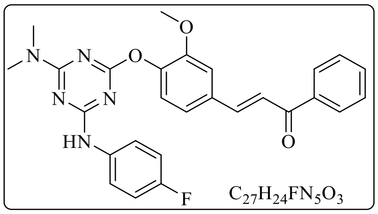

(E)-3-(4-((4-(dimethylamino)-6-((4-fluorophenyl)amino)-1,3,5-triazin-2-yl)oxy)-3-methoxyphenyl)-1-phenylprop-2-en-1-one ***(21a)***. Beige solid. 73% yield; mp 201-202 °C. FT-IR (ATR): ν (cm^{-1}) 3262 (N-H), 3119 (=C-H), 1676 (C=O), 1552 and 1504 (C=N and C=C). ^1H NMR (400 MHz, DMSO-d_6) δ ppm 3.01 (s, 3H, CH_3), 3.12 (s, 3H, CH_3), 3.85 (s, 3H, OCH_3), 6.80-7.16 (m, 2H, Ar-H), 7.25 (d, *J* = 8.1 Hz, 1H, Ar-H), 7.43 (d, *J* = 8.1 Hz, 1H, Ar-H), 7.50 (d, *J* = 8.1 Hz, 1H, Ar-H), 7.59 (t, *J* = 7.6 Hz, 2H, Ar-H), 7.65-7.75 (m, 3H, Ar-H), 7.79 (d, *J* = 15.6 Hz, 1H, CH), 7.99 (d, *J* = 15.6 Hz, 1H, CH), 8.19 (d, *J* = 7.6 Hz, 2H, Ar-H), 9.59 (bs, 1H, NH). ^{13}C NMR (100 MHz, DMSO-d_6) δ ppm 35.8 (CH_3), 36.2 (CH_3), 56.1 (CH_3), 112.5 (CH), 114.8 (d, $^2J_{CF}$ = 23.9 Hz, CH), 121.4 (d, $^3J_{CF}$ = 9.0 Hz, CH), 121.9 (CH), 122.6 (CH), 123.4 (CH), 123.8 (C), 128.6 (CH), 128.8 (CH), 133.1 (C), 133.2 (CH), 136.0 (C), 137.6 (C), 143.1 (CH), 143.8 (C), 151.8 (C), 164.7 (C), 166.3 (C), 170.8 (C), 171.2 (d, $^1J_{CF}$ = 235.0 Hz, C). MS (70 eV) m/z (%): 485 (69), 454 (27), 375 (100), 233 (11), 163 (8), 96 (40). Anal. Calcd. for $C_{27}H_{24}FN_5O_3$: C, 66.79; H, 4.98; N, 14.42; Found: C, 66.83; H, 5.02; N, 14.47.

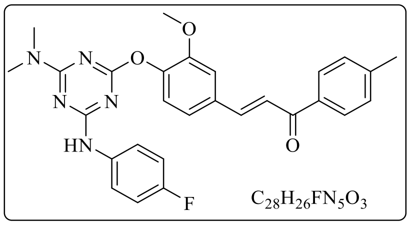

(E)-3-(4-((4-(dimethylamino)-6-((4-fluorophenyl)amino)-1,3,5-triazin-2-yl)oxy)-3-methoxyphenyl)-1-(p-tolyl)prop-2-en-1-one ***(21b)***. Beige solid. 76% yield; mp 206-208 °C. FT-IR (ATR): ν (cm^{-1}) 3252 (N-H), 3119 (=C-H), 1660 (C=O), 1549 and 1500 (C=N and C=C). ^1H NMR (400 MHz, DMSO-d_6) δ ppm 2.41 (s, 3H, CH_3), 3.01 (s, 3H, CH_3), 3.11 (s, 3H, CH_3), 3.84 (s, 3H, OCH_3), 6.85-7.15 (m, 2H, Ar-H), 7.23 (d, *J* = 8.1 Hz, 1H, Ar-H), 7.39 (d, *J* = 8.1 Hz, 2H, Ar-H), 7.48 (d, *J* = 8.1 Hz, 1H, Ar-H), 7.55-7.84 (m, 4H, Ar-H), 7.96 (d, *J* = 15.6 Hz, 1H, CH), 8.09 (d, *J* = 8.1 Hz, 2H, Ar-H), 9.55 (bs, 1H, NH). ^{13}C NMR (100 MHz, DMSO-d_6) δ ppm 21.2 (CH_3), 35.8 (CH_3), 36.1 (CH_3), 56.1 (CH_3), 112.5 (CH), 114.8 (d, $^2J_{CF}$ = 24.2 Hz, CH), 121.5 (d, $^3J_{CF}$ = 7.1 Hz, CH), 122.0 (CH), 122.4 (CH), 123.4 (CH), 123.8 (d, $^4J_{CF}$ = 3.8 Hz, C), 128.7 (CH), 129.4 (CH), 133.2 (C), 135.1 (C), 136.0 (C), 143.0 (C), 143.4 (C), 143.7 (CH), 151.8 (C), 164.7 (C), 166.3 (C), 168.88 (d, *J* = 243.3 Hz), 188.7 (C). MS (70 eV) m/z (%): 499 (67), 468 (28), 389 (100), 233 (17), 163 (27), 119 (58). Anal. Calcd. for $C_{28}H_{26}FN_5O_3$: C, 67.32; H, 5.25; N, 14.02; Found: C, 67.29; H, 5.20; N, 14.10.

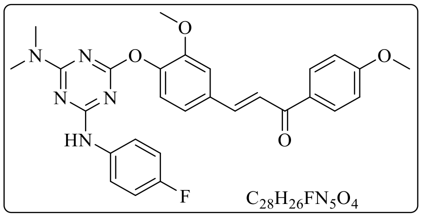

(E)-3-(4-((4-(dimethylamino)-6-((4-fluorophenyl)amino)-1,3,5-triazin-2-yl)oxy)-3-methoxyphenyl)-1-(4-methoxyphenyl)prop-2-en-1-one ***(21c)***. Beige solid. 72% yield; mp 224-226 °C. FT-IR (ATR): ν (cm^{-1}) 3336 (N-H), 3115 (=C-H), 1655 (C=O), 1585 and 1501 (C=N and C=C). ^1H NMR (400 MHz, DMSO-d_6) δ ppm 3.01 (s, 3H, CH_3), 3.12 (s, 3H, CH_3), 3.85 (s, 3H. OCH_3), 3.87 (s, 3H. OCH_3), 6.80-7.06 (m, 2H, Ar-H), 7.11 (d, *J* = 8.5 Hz, 2H, Ar-H), 7.24 (d, *J* = 8.0 Hz, 1H, Ar-H), 7.49 (d, *J* = 8.0 Hz, 1H, Ar-H), 7.54-7.85 (m, 4H, Ar-H), 7.99 (d, *J* = 15.4 Hz, 1H, CH), 8.20 (d, *J* = 8.5 Hz, 2H, Ar-H), 9.59 (bs, 1H, NH). ^{13}C NMR (100 MHz, DMSO-d_6) δ ppm 35.8 (CH_3), 36.2 (CH_3), 55.6 (CH_3), 56.1 (CH_3), 112.4 (CH), 114.1 (CH), 114.8 (d, $^2J_{CF}$ = 26.2 Hz, CH), 121.5 (d, $^3J_{CF}$ = 9.6 Hz, CH), 121.9 (CH), 122.4 (CH), 123.4 (CH), 130.5 (C), 131.0 (CH), 133.3 (C), 136.0 (C), 142.9 (CH), 143.0 (C), 151.7 (C), 163.3 (C), 164.7 (C), 166.3 (C), 168.80 (d, $^1J_{CF}$ = 260.4 Hz, C), 170.4 (C), 187.4 (C). MS (70 eV) m/z (%): 515 (62), 484

(28), 405 (100), 284 (13), 233 (13), 135 (60). Anal. Calcd. for $C_{28}H_{26}FN_5O_4$: C, 65.23; H, 5.08; N, 13.58; Found: C, 65.19; H, 5.01; N, 13.65.

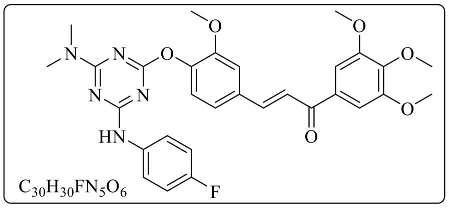

(E)-3-(4-((4-(dimethylamino)-6-((4-fluorophenyl)amino)-1,3,5-triazin-2-yl)oxy)-3-methoxyphenyl)-1-(3,4,5-trimethoxyphenyl)prop-2-en-1-one (***21d***). Beige solid. 65% yield; mp 217-219 °C. FT-IR (ATR): ν (cm^{-1}) 3259 (N-H), 3119 (=C-H), 1659 (C=O), 1590 and 1504 (C=N and C=C). ^{1}H NMR (400 MHz, DMSO-d_6) δ ppm 3.01 (s, 3H, CH_3), 3.12 (s, 3H, CH_3), 3.77 (s, 3H, OCH_3), 3.83 (s, 3H, OCH_3), 3.91 (s, 6H, OCH_3), 6.77-7.18 (m, 2H, Ar-H), 7.26 (d, J = 8.2 Hz, 1H, Ar-H), 7.44 (s, 2H, Ar-H), 7.46- 7.75 (m, 4H, Ar-H), 7.80 (d, J = 15.4 Hz, 1H, CH), 7.95 (d, J = 15.4 Hz, 1H, CH), 9.60 (bs, 1H, NH). ^{13}C NMR (100 MHz, DMSO-d_6) δ ppm 35.8 (CH_3), 36.2 (CH_3), 56.1 (CH_3), 56.3 (CH_3), 60.3 (CH_3), 106.3 (CH), 113.4 (CH), 114.8 (d, $^2J_{CF}$ = 21.7 Hz, CH), 121.4 (d, $^3J_{CF}$ = 9.3 Hz, CH), 121.5 (C), 121.9 (CH), 122.0 (CH) 123.4 (CH), 133.1 (C), 133.2 (C), 136.0 (d, $^4J_{CF}$ = 3.0 Hz, C) , 142.0 (C), 143.0 (C), 143.7 (CH), 151.7 (C), 153.0 (C), 164.7 (C), 166.2 (C), 168.81 (d, $^1J_{CF}$ = 252.8 Hz, C), 188.1 (C). MS (70 eV) m/z (%): 575 (69), 544 (27), 465 (100), 382 (24), 344 (36), 233 (23). Anal. Calcd. for $C_{30}H_{30}FN_5O_6$: C, 62.60; H, 5.25; N, 12.17; Found: C, 62.58; H, 5.22; N, 12.20.

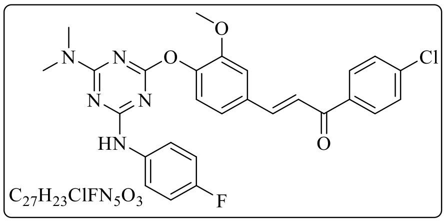

(E)-1-(4-chlorophenyl)-3-(4-((4-(dimethylamino)-6-((4-fluorophenyl)amino)-1,3,5-triazin-2-yl)oxy)-3-methoxyphenyl)prop-2-en-1-one (***21e***). Beige solid. 80% yield; mp 214-216 °C. FT-IR (ATR): ν (cm^{-1}) 3258 (N-H), 3119 (=C-H), 1660 (C=O), 1599 and 1500 (C=N and C=C). ^{1}H NMR (400 MHz, DMSO-d_6) δ ppm 3.01 (s, 3H, CH_3), 3.11 (s, 3H, CH_3), 3.84 (s, 3H, OCH_3), 6.81-7.16 (m, 2H, Ar-H), 7.25 (d, J = 8.1 Hz, 1H, Ar-H), 7.28-7.63 (m, 3H, Ar-H), 7.66 (d, J = 8.5 Hz, 2H, Ar-H), 7.73 (s, 1H, Ar-H), 7.80 (d, J = 15.5 Hz, 1H, CH), 7.97 (d, J = 15.5 Hz, 1H, Ar-H), 8.21 (d, J = 8.5 Hz, 2H, Ar-H), 9.59 (bs, 1H, NH). ^{13}C NMR (100 MHz, DMSO-d_6) δ ppm 35.7 (CH_3), 36.1 (CH_3), 56.1 (CH_3), 112.5 (CH), 114.8 (d, $^2J_{CF}$ = 24.3 Hz, CH), 121.4 (d, $^3J_{CF}$ = 9.1 Hz, CH), 121.5 (CH), 122.7 (CH), 123.4 (CH), 128.9 (CH), 130.1 (C), 130.5 (CH), 133.0 (C), 136.0 (d, $^4J_{CF}$ = 2.0 Hz, C), 136.3 (C), 138.2 (C), 143.2 (C), 144.3 (CH), 151.7 (C), 164.7 (C), 166.2 (C), 171.5 (d, $^1J_{CF}$ = 292.1 Hz), 188.1 (C). MS (70 eV) *m/z* (%): 519: 521 [M^+]:$[M + 2]^+$ (60/23), 488 (26), 409 (100), 233 (17), 163 (30), 139 (39). Anal. Calcd. for $C_{27}H_{23}ClFN_5O_3$: C, 62.37; H, 4.46; N, 13.47; Found: C, 62.41; H, 4.51; N, 13.44.

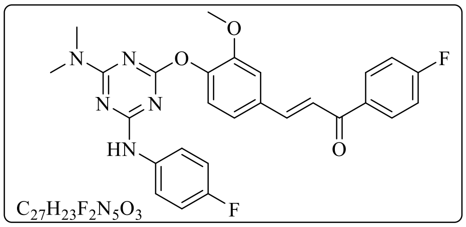

(E)-3-(4-((4-(dimethylamino)-6-((4-fluorophenyl)amino)-1,3,5-triazin-2-yl)oxy)-3-methoxyphenyl)-1-(4-fluorophenyl)prop-2-en-1-one (***21f***). Beige solid. 77% yield; mp 220-222 °C. FT-IR (ATR): ν (cm^{-1}) 3334 (N-H), 3078 (=C-H), 1723 (C=O), 1588 and 1504 (C=N and C=C). ^{1}H NMR (400 MHz, DMSO-d_6) δ ppm 3.01 (s, 3H, CH_3), 3.12 (s, 3H, CH_3), 3.84 (s, 3H, OCH_3), 6.79-7.19 (m, 2H, Ar-H), 7.25 (d, J = 8.1 Hz, 1H, Ar-H), 7.42 (t, J = 8.7 Hz, 2H, Ar-H), 7.48-7.77 (m, 4H, Ar-H), 7.80 (d, J = 15.7 Hz, 1H, CH), 7.99 (d, J = 15.7 Hz, 1H, CH), 8.28 (dd, J = 8.4, 5.7 Hz, 2H, Ar-H), 9.59 (bs, 1H, Ar-H). ^{13}C NMR (100 MHz,

DMSO-d_6) δ ppm 35.8 (CH_3), 36.2 (CH_3), 56.1 (OCH_3), 112.5 (CH), 114.8 (d, $^2J_{CF}$ = 24.3 Hz, CH), 115.9 (d, $^2J_{CF}$ = 21.4 Hz, CH), 121.4 (d, $^3J_{CF}$ = 7.5 Hz, CH), 121.6 (CH), 122.6 (CH), 123.4 (CH), 131.6 (d, $^3J_{CF}$ = 9.0 Hz, CH), 133.1 (C), 134.3 (d, $^4J_{CF}$ = 2.6 Hz, C), 135.9 (C), 136.02 (d, $^4J_{CF}$ = 2.2 Hz, C), 143.1 (C), 144.0 (CH), 151.8 (C), 164.7 (C), 165.1 (d, $^1J_{CF}$ = 238.8 Hz, C), 166.4 (C), 168.6 (d, $^1J_{CF}$ = 284.9 Hz, C), 187.7 (C). MS (70 eV) m/z (%): 503 (62), 472 (24), 393 (100), 233 (11), 163 (17), 123 (37). Anal. Calcd. for $C_{27}H_{23}F_2N_5O_3$: C, 64.41; H, 4.60; N, 13.91; Found: C, 64.38; H, 4.56; N, 13.98.

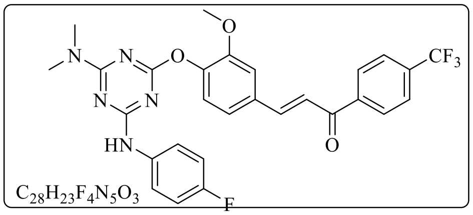

*(E)-3-(4-((4-(dimethylamino)-6-((4-fluorophenyl)amino)-1,3,5-triazin-2-yl)oxy)-3-methoxyphenyl)-1-(4-(trifluoromethyl)phenyl)prop-2-en-1-one (**21g**)*. Beige solid. 73% yield; mp 188-189 °C. FT-IR (ATR): ν (cm^{-1}) 3262 (N-H), 3125 (=C-H), 1661 (C=O), 1598 and 1501 (C=N and C=C). ^{1}H NMR (400 MHz, DMSO-d_6) δ ppm 3.01 (s, 3H, CH_3), 3.12 (s, 3H, CH_3), 3.85 (s, 3H, OCH_3), 6.83-7.18 (m, 2H, Ar-H), 7.27 (d, *J* = 8.1 Hz, 1H, Ar-H), 7.53 (d, *J* = 8.1 Hz, 1H, Ar-H), 7.58-7.79 (m, 3H, Ar-H), 7.84 (d, *J* = 15.6 Hz, 1H, CH), 7.91-8.03 (m, 3H, Ar-H, CH), 8.35 (d, *J* = 8.1 Hz, 2H, Ar-H), 9.60 (bs, 1H, NH).NMR ^{13}C (100 MHz, DMSO-d_6) δ ppm 35.8 (CH_3), 36.1 (CH_3), 56.1 (CH_3), 112.7 (CH), 114.8 (d, $^2J_{CF}$ = 26.0 Hz, CH), 121.4 (d, $^3J_{CF}$ = 9.0 Hz, CH), 121.7 (CH), 122.8 (CH), 123.4 (q, $^1J_{CF}$ = 271.0 Hz, CF_3), 123.5 (CH), 125.8 (d, $^4J_{CF}$ = 3.0 Hz, CH), 129.4 (CH), 132.3 (C), 132.6 (C), 132.9 (C), 136.0 (d, $^4J_{CF}$ = 3.0 Hz, C), 140.9 (C), 143.3 (C), 145.0 (CH), 151.8 (C), 164.7 (C), 166.3 (C), 168.9 (d, $^1J_{CF}$ = 230.0 Hz, C), 188.8 (C). MS (70 eV) m/z (%): 553 (63), 522 (23), 443 (100), 233 (14), 163 (19). Anal. Calcd. for $C_{28}H_{23}F_4N_5O_3$: C, 60.76; H, 4.19; N, 12.65; Found: C, 60.70; H, 4.09; N, 12.66.

Characterization of triazinylamino-chalcones (23a-g)

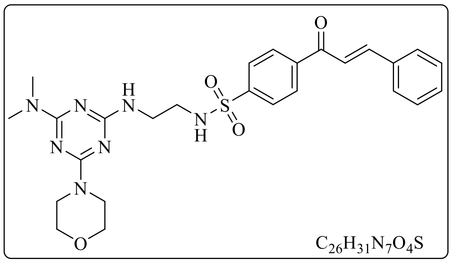

*4-cinnamoyl-N-(2-((4-(dimethylamino)-6-morpholino-1,3,5-triazin-2-yl)amino)ethyl)benzenesulfonamide (**23a**)*. White solid. 84% yield; mp 156-158 °C. FT-IR (ATR): ν (cm^{-1}) 3114 (N-H), 2987 (C-H), 1664 (C=O), 1606 and 1575 (C=N and C=C), 1157 (S=O). ^{1}H NMR (400 MHz, $CDCl_3$) δ ppm 2.95-3.21 (m, 8H, CH_3, CH_2), 3.48 (q, *J* = 5.8 Hz, 2H, CH_2), 3.71 (bs, 9H, CH_2, NH), 5.00 (bs, 1H, NH), 7.40-7.50 (m, 4H, Ar-H, CH), 7.65 (dd, *J* = 6.5, 2.6 Hz, 2H, Ar-H), 7.81 (d, *J* = 15.3 Hz, 1H, CH), 7.84 (d, *J* = 8.2 Hz, 2H, Ar-H), 8.02 (d, *J* = 8.2 Hz, 2H). ^{13}C NMR (100 MHz, $CDCl_3$) δ ppm 36.4 (CH_3), 40.3 (CH_2), 43.8 (CH_2), 46.1 (CH_2), 66.9 (CH_2), 121.6 (CH), 127.3 (CH), 128.7 (CH), 129.0 (CH), 129.2 (CH), 131.2 (CH), 134.6 (C), 141.2 (C), 143.6 (C), 146.3 (CH), 164.7 (C), 165.1 (C), 166.7 (C), 189.6 (C). MS (70 eV) m/z (%): 537 (48), 492 (5), 480 (5), 266 (100), 237 (98), 207 (31). Anal. Calcd. for $C_{26}H_{31}N_7O_4S$: C, 58.08; H, 5.81; N, 18.24; S, 5.96; Found: C, 58.10; H, 5.79; N, 18.22; S, 5.90.

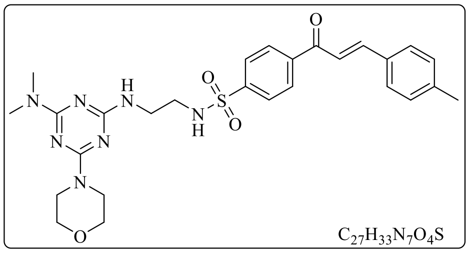

(E)-N-(2-((4-(dimethylamino)-6-morpholino-1,3,5-triazin-2-yl)amino)ethyl)-4-(3-(p-tolyl)acryloyl)benzenesulfonamide ***(23b)***. White solid. 80% yield; mp 165-167 °C. 3329 (N-H), 2987 (C-H), 1662 (C=O), 1566 and 1527 (C=N and C=C), 1152 (S=O). ^{1}H NMR (400 MHz, $CDCl_3$) δ ppm 2.40 (s, 3H, CH_3), 2.89-3.30 (m, 8H, CH_3, CH_2), 3.47 (q, *J* = 5.8 Hz, 2H, CH_2), 3.71 (bs, 9H, CH_2, NH), 4.99 (bs, 1H, NH), 7.23 (d, *J* = 7.9 Hz, 2H, Ar-H), 7.42 (d, *J* = 15.7 Hz, 1H, CH), 7.54 (d, *J* = 7.9 Hz, 2H, Ar-H), 7.79 (d, *J* = 15.7 Hz, 1H, CH), 7.83 (d, *J* = 8.4 Hz, 2H, Ar-H), 8.01 (d, *J* = 8.4 Hz, 2H, Ar-H). ^{13}C NMR (100 MHz, $CDCl_3$) δ ppm 21.7 (CH_3), 36.4 (CH_3), 40.3 (CH_2), 43.8 (CH_2), 46.1 (CH_2), 66.9 (CH_2), 120.6 (CH), 127.2 (CH), 128.8 (CH), 129.0 (CH), 130.0 (CH), 131.9 (C), 141.4 (C), 141.9 (C), 143.4 (C), 146.5 (CH), 164.7 (C), 165.2 (C), 166.8 (C), 189.6 (C). MS (70 eV) m/z (%): 551 (43), 523 (7), 266 (100), 237 (97), 207 (22). Anal. Calcd. for $C_{27}H_{33}N_7O_4S$: C, 58.79; H, 6.03; N, 17.77; S, 5.81; Found: C, 58.83; H, 6.07; N, 17.80; S, 5.85.

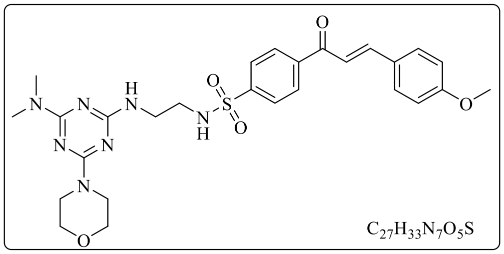

(E)-N-(2-((4-(dimethylamino)-6-morpholino-1,3,5-triazin-2-yl)amino)ethyl)-4-(3-(4-methoxyphenyl)acryloyl)benzenesulfonamide ***(23c)***. Yellow solid. 87% yield; mp 135-138 °C. FT-IR (ATR): ν (cm^{-1}) 3340 (N-H), 2968 (C-H), 1660 (C=O), 1564 and 1526 (C=N and C=C), 1151 (S=O). ^{1}H NMR (400 MHz, $CDCl_3$) δ ppm 2.93-3.21 (m, 8H, CH_3, CH_2), 3.47 (q, *J* = 5.8 Hz, 2H, CH_2), 3.70 (bs, 9H, CH_2, NH), 3.85 (s, 3H, OCH_3), 5.00 (bs, 1H, NH), 6.94 (d, *J* = 8.7 Hz, 2H, Ar-H), 7.33 (d, *J* = 15.6 Hz, 1H, CH), 7.60 (d, *J* = 8.7 Hz, 2H, Ar-H), 7.78 (d, *J* = 15.6 Hz, 1H, CH), 7.82 (d, *J* = 8.3 Hz, 2H, Ar-H), 8.00 (d, *J* = 8.3 Hz, 2H, Ar-H). ^{13}C NMR (100 MHz, $CDCl_3$) δ ppm 36.3 (CH_3), 40.3 (CH_2), 43.7 (CH_2), 46.0 (CH_2), 55.6 (OCH_3), 66.9 (CH_2), 114.7 (CH), 119.3 (CH), 127.2 (CH), 127.3 (C), 128.9 (CH), 130.6 (CH), 141.6 (C), 143.3 (C), 146.2 (CH), 162.2 (C), 164.7 (C), 165.2 (C), 166.7 (C), 189.6 (C). MS (70 eV) m/z (%): 567 (46), 539 (6), 509 (7), 266 (100), 237 (99), 207 (20). Anal. Calcd. for $C_{27}H_{37}N_7O_5S$: C, 57.13; H, 5.86; N, 17.27; S, 5.65; Found: C, 57.11; H, 5.80; N, 17.32; S, 5.66.

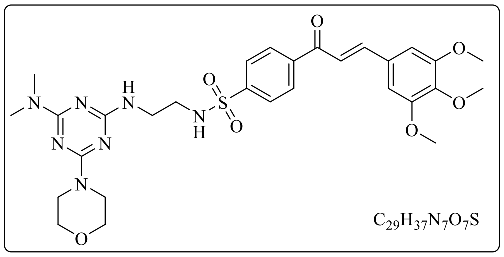

(E)-N-(2-((4-(dimethylamino)-6-morpholino-1,3,5-triazin-2-yl)amino)ethyl)-4-(3-(3,4,5-trimethoxyphenyl)acryloyl)benzenesulfonamide ***(23d)***. Yellow solid. 67% yield; mp 108-109 °C. FT-IR (ATR): ν (cm^{-1}) 3400 (N-H), 2970 (C-H), 1664 (C=O), 1564 and 1528 (C=N and C=C), 1157 (S=O). ^{1}H NMR (400 MHz, $CDCl_3$) δ ppm 2.99-3.19 (m, 8H, CH_3, CH_2), 3.47 (q, *J* = 5.3 Hz, 2H, CH_2), 3.70 (bs, 9H, CH_2, NH), 3.90 (s, 3H, OCH_3), 3.91 (s, 6H, OCH_3), 5.02 (bs, 1H, NH), 6.86 (s, 2H, Ar-H), 7.32 (d, *J* = 15.6 Hz, 1H, CH), 7.71 (d, *J* = 15.6 Hz, 1H, CH), 7.83 (d, *J* = 8.1 Hz, 2H, Ar-H), 8.00 (d, *J* = 8.1 Hz, 2H, Ar-H). ^{13}C NMR (100 MHz, $CDCl_3$) δ ppm 36.4 (CH_3), 40.3 (CH_2), 43.8 (CH_2), 46.0 (CH_2), 56.4 (CH_3), 61.1 (CH_3), 66.9 (CH_2), 106.1 (CH), 121.0 (CH), 127.2 (CH), 129.0 (CH), 130.0 (C), 141.1 (C), 141.3 (C), 143.5 (C), 146.6 (CH), 153.7 (C), 164.7 (C), 165.2 (C), 166.7 (C), 189.7 (C). MS (70 eV) m/z (%): 627 (41), 596 (5), 266 (100), 337

(93), 207 (25). Anal. Calcd. for $C_{29}H_{37}N_7O_7S$: C, 55.49; H, 5.94; N, 15.62; S, 5.11; Found: C, 55.53; H, 5.98; N, 15.59; S, 5.08.

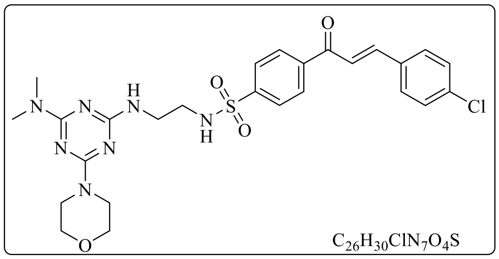

*(E)-4-(3-(4-chlorophenyl)acryloyl)-N-(2-((4-(dimethylamino)-6-morpholino-1,3,5-triazin-2-yl)amino)ethyl)benzenesulfonamide (**23e**).* Beige Oil. 83% yield. FT-IR (ATR): ν (cm^{-1}) 3410 (N-H), 2967 (C-H), 1663 (C=O), 1562 and 1525 (C=N and C=C), 1158 (S=O). ^1H NMR (400 MHz, $CDCl_3$) δ ppm 2.95-3.22 (m, 8H, CH_3, CH_2), 3.48 (q, J = 5.8 Hz, 2H, CH_2), 3.71 (bs, 9H, CH_2, NH), 5.01 (bs, 1H, NH), 7.40 (d, J = 8.5 Hz, 2H, Ar-H), 7.43 (d, J = 15.7 Hz, 1H ,CH), 7.58 (d, J = 8.5 Hz, 2H, Ar-H), 7.76 (d, J = 15.7 Hz, 1H, CH), 7.84 (d, J = 8.3 Hz, 2H, Ar-H), 8.01 (d, J = 8.3 Hz, 2H, Ar-H). ^{13}C NMR (100 MHz, $CDCl_3$) δ ppm 36.4 (CH_3), 40.3 (CH_2), 43.8 (CH_2), 46.1 (CH_2), 66.9 (CH_2), 122.0 (CH), 127.3 (CH), 129.0 (CH), 129.5 (CH), 129.9 (CH), 133.1 (C), 137.1 (C), 141.0 (C), 143.7 (C), 144.8 (CH), 164.6 (C), 165.2 (C), 166.7 (C), 189.2 (C). MS (70 eV) m/z (%): 571:573 [M^+]:[M + 2]$^+$ (16/7), 294 (15), 266 (64), 237 (69), 207 (21), 139 (37). Anal. Calcd. for $C_{26}H_{30}ClN_7O_4S$: C, 54.59; H, 5.29; Cl, 6.20; N, 17.14; S, 5.60; Found: C, 54.63; H, 5.33; Cl, 6.18; N, 17.12; S, 5.57.

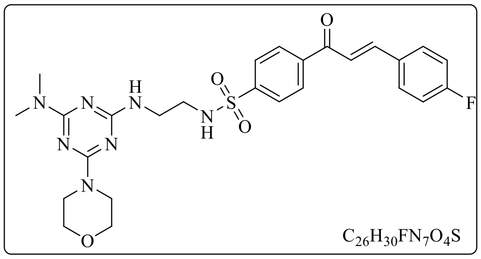

*(E)-N-(2-((4-(dimethylamino)-6-morpholino-1,3,5-triazin-2-yl)amino)ethyl)-4-(3-(4-fluorophenyl)acryloyl)benzenesulfonamide (**23f**).* White solid. 80% yield; mp 93-95 °C. FT-IR (ATR): ν (cm^{-1}) 3402 (N-H), 2970 (C-H), 1665 (C=O), 1563 and 1528 (C=N and C=C), 1157 (S=O). ^1H NMR (400 MHz, $CDCl_3$) δ ppm 2.94-3.20 (m, 8H, CH_3, CH_2), 3.48 (q, J = 5.8 Hz, 2H, CH_2), 3.71 (bs, 9H, CH_2, NH), 4.97 (bs, 1H, NH), 7.12 (t, J = 8.6 Hz, 2H, Ar-H), 7.39 (d, J = 15.7 Hz, 1H, CH), 7.64 (dd, J = 8.6, 5.4 Hz, 2H, Ar-H), 7.78 (d, J = 15.7 Hz, 1H, CH), 7.84 (d, J = 8.4 Hz, 2H, Ar-H), 8.01 (d, J = 8.4 Hz, 2H, Ar-H). ^{13}C NMR (100 MHz, $CDCl_3$) δ ppm 36.4 (CH_3), 40.3 (CH_2), 43.8 (CH_2), 46.2 (CH_2), 66.9 (CH_2), 116.4 (d, $^2J_{CF}$ = 22.0 Hz, CH), 121.3 (CH), 127.3 (CH), 129.0 (CH), 130.7 (d, $^3J_{CF}$ = 8.6 Hz, CH), 130.9 (d, $^4J_{CF}$ = 3.4 Hz, C), 130.9 (C), 141.1 (C), 143.6 (C), 145.0 (CH), 164.2 (d, $^1J_{CF}$ = 203.7 Hz, C), 164.7 (C), 166.9 (C), 189.3 (C). MS (70 eV) m/z (%): 555 (35), 510 (5), 266 (100), 237 (99), 207 (21), 138 (15). Anal. Calcd. for $C_{26}H_{30}FN_7O_4S$: C, 56.20; H, 5.44; N, 17.65; S, 5.77; Found: C, 56.16; H, 5.40; N, 17.58; S, 5.81.

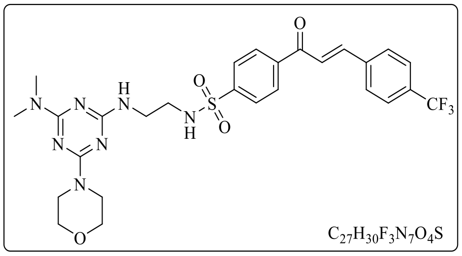

*(E)-N-(2-((4-(dimethylamino)-6-morpholino-1,3,5-triazin-2-yl)amino)ethyl)-4-(3-(4-(trifluoromethyl)phenyl)acryloyl)benzenesulfonamide (**23g**).* white oil. 76% yield. FT-IR (ATR): ν (cm^{-1}) 3430 (N-H), 2987 (C-H), 1665 (C=O), 1565 and 1526 (C=N and C=C), 1158 (S=O). ^1H NMR (400 MHz, $CDCl_3$) δ ppm 2.97-3.21 (m, 8H, CH_3, CH_2), 3.48 (q, J = 5.2 Hz, 2H, CH_2), 3.71 (bs, 9H, CH_2, NH), 4.99 (bs, 1H, NH), 7.53 (d, J = 15.5 Hz, 1H, CH), 7.69 (d, J = 8.1 Hz, 2H, Ar-H), 7.73-7.82 (m, 3H, Ar-H, CH), 7.86 (d, J = 8.3 Hz, 2H, Ar-H), 8.03 (d, J = 8.3 Hz, 2H, Ar-H).

^{13}C NMR (100 MHz, $CDCl_3$) δ ppm 36.4 (CH_3), 40.3 (CH_2), 43.8 (CH_2), 46.2 (CH_2), 66.9 (CH_2), 123.8 (CH), 126.17 (d, $^3J_{CF}$ = 3.7 Hz, CH), 127.4 (CH), 128.3 (q, $^1J_{CF}$ = 281.0 Hz, CF_3), 128.8 (CH), 129.1 (CH), 132.49 (d, $^2J_{CF}$ = 32.9 Hz, C), 138.0 (C), 140.7 (C), 144.0 (C), 144.2 (CH), 164.7 (C), 165.2 (C), 166.8 (C), 189.1 (C).MS (70 eV) m/z (%): 605 (8), 581 (11), 492 (9), 266 (38), 237 (40), 207 (16). Anal. Calcd. for $C_{27}H_{30}F_3N_7O_4S$: C, 53.55; H, 4.99; N, 16.19; S, 5.29; Found: C, 53.61; H, 5.02; N, 16.14; S, 5.27.

Characterization of triazinylamino-chalcones (24a-g)

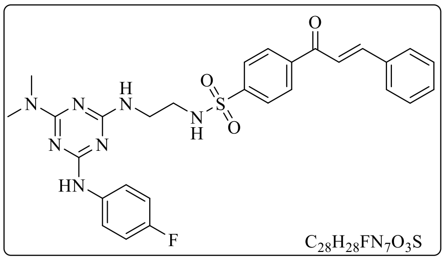

*4-cinnamoyl-N-(2-((4-(dimethylamino)-6-((4-fluorophenyl)amino)-1,3,5-triazin-2-yl)amino)ethyl)benzenesulfonamide (**24a**)*. White solid. 87% yield; mp 208-209 °C. FT-IR (ATR): ν (cm^{-1}) 3428 (N-H), 3061 (=C-H), 1655 (C=O), 1578 and 1503 (C=N and C=C), 1153 (S=O). ^1H NMR (400 MHz, DMSO-d_6) δ ppm 2.85-2.95 (m, 2H, CH_2), 3.01 (s, 6H, CH_3), 3.19-3.33 (m, 2H, CH_2), 3.63 (bs, 1H, NH), 6.50 (bs, 1H, NH), 7.03 (t, J = 7.8 Hz, 2H), 7.41-7.50 (m, 3H, Ar-H), 7.69-7.95 (m, 8H, Ar-H, CH), 8.10-8.19 (m, 2H, Ar-H), 8.93 (bs, 1H, NH). ^{13}C NMR (100 MHz, DMSO-d_6) δ ppm 35.7 (CH_3), 42.1 (CH_2), 44.1 (CH_2), 114.6 (d, $^2J_{CF}$ = 22.1 Hz, CH), 120.7 (d, $^3J_{CF}$ = 7.0 Hz, CH), 122.1 (CH), 126.4 (CH), 126.4 (CH), 128.6 (CH), 128.9 (CH), 130.7 (CH), 134.6 (C), 137.2 (C), 138.1 (C), 144.2 (CH), 148.3 (C), 156.84 (d, $^1J_{CF}$ = 237.3 Hz, C), 163.8 (C), 165.3 (C), 165.5 (C), 188.6 (C). MS (70 eV) m/z (%): 561 (4), 290 (36), 261 (100), 248 (26), 233 (17), 162 (13). Anal. Calcd. for $C_{28}H_{28}FN_7O_3S$: C, 59.88; H, 5.03; N, 17.46; S, 5.71; Found: C, 59.86; H, 4.99; N, 17.50; S, 5.68

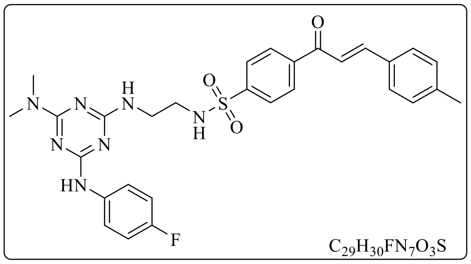

*(E)-N-(2-((4-(dimethylamino)-6-((4-fluorophenyl)amino)-1,3,5-triazin-2-yl)amino)ethyl)-4-(3-(p-tolyl)acryloyl)benzenesulfonamide (**24b**)*. White solid. 85% yield; mp 216-218 °C. FT-IR (ATR): ν (cm^{-1}) 3389 (N-H), 3065 (=C-H), 1653 (C=O), 1599 and 1505 (C=N and C=C), 1156 (S=O). ^1H NMR (400 MHz, DMSO-d_6) δ ppm 2.35 (s, 3H, CH_3), 2.87-2.97 (m, 2H, CH_2), 3.01 (s, 6H, CH_3), 3.20-3.32 (m, 2H, CH_2), 3.57 (bs, 1H, NH), 6.56 (bs, 1H, NH), 7.03 (t, J = 7.6 Hz, 2H, Ar-H), 7.27 (d, J = 7.8 Hz, 2H, Ar-H), 7.68-7.88 (m, 8H, Ar-H, CH), 8.11-8.20 (m, 2H, Ar-H), 8.93 (bs, 1H, NH). ^{13}C NMR (100 MHz, DMSO-d_6) δ ppm 21.1 (CH_3), 35.6 (CH_3), 41.4 (CH_2), 43.6 (CH_2), 114.6 (d, $^2J_{CF}$ = 22.5 Hz, CH), 120.7 (d, $^3J_{CF}$ = 7.3 Hz, CH), 121.0 (CH), 126.4 (CH), 128.6 (CH), 129.0 (CH), 129.5 (CH), 131.9 (C), 137.2 (C), 138.6 (C), 140.8 (C), 144.4 (CH), 147.8 (C), 156.86 (d, $^1J_{CF}$ = 237.5 Hz, C), 163.8 (C), 165.1 (C), 165.5 (C), 188.5 (C). MS (70 eV) m/z (%): 575 (3), 393 (6), 290 (26), 261 (100), 248 (32), 162 (16). Anal. Calcd. for $C_{29}H_{30}FN_7O_3S$: C, 60.51; H, 5.25; N, 17.03; S, 5.57; Found: C, 60.48; H, 5.26; N, 17.09; S, 5.63.

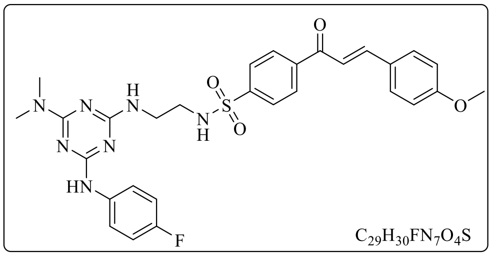

(E)-N-(2-((4-(dimethylamino)-6-((4-fluorophenyl)amino)-1,3,5-triazin-2-yl)amino)ethyl)-4-(3-(4-methoxyphenyl)acryloyl)benzenesulfonamide (***24c***). White solid. 85% yield; mp 226-227 °C. FT-IR (ATR): ν (cm^{-1}) 3431 (N-H), 3061 (=C-H), 1651 (C=O), 1597 and 1505 (C=N and C=C), 1146 (S=O). ^{1}H NMR (400 MHz, DMSO-d_6) δ ppm 2.91 (bs, 2H, CH_2), 3.01 (s, 6H, CH_3), 3.24 (bs, 2H, CH_2), 3.41 (bs, 1H, NH), 3.82 (s, 3H, OCH_3), 6.51 (bs, 1H, NH), 6.98-7.08 (m, 4H, Ar-H), 7.68-7.89 (m, 8H, Ar-H, CH), 8.09-8.19 (m, 2H, Ar-H), 8.93 (bs, 1H, NH). ^{13}C NMR (100 MHz, DMSO-d_6) δ ppm 35.6 (CH_3), 41.6 (CH_2), 44.2 (CH_2), 55.4 (CH_3), 114.4 (CH), 114.6 (d, $^2J_{CF}$ = 20.2 Hz, CH) 119.5 (CH), 120.7 (d, J = 6.6 Hz, CH), 126.4 (CH), 127.2 (C), 128.5 (CH), 130.9 (CH), 137.1 (C), 138.4 (C), 141.6 (C), 144.3 (CH), 156.85 (d, $^1J_{CF}$ = 236.2 Hz, C), 161.5 (C), 163.7 (C), 165.1 (C), 165.2 (C), 165. 6 (C), 188.4 (C). MS (70 eV) m/z (%): 591 (6), 563 (1), 304 (23), 290 (38), 261 (100), 248 (12), 137 (16). Anal. Calcd. for $C_{29}H_{30}FN_7O_4S$: C, 58.87; H, 5.11; N, 16.57; S, 5.42; Found: C, 58.91; H, 5.15; N, 16.48; S, 5.40.

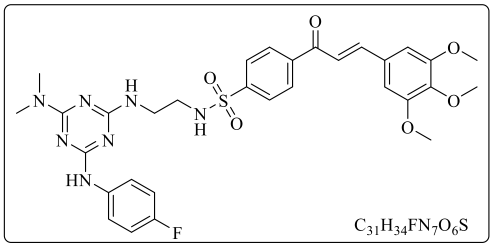

(E)-N-(2-((4-(dimethylamino)-6-((4-fluorophenyl)amino)-1,3,5-triazin-2-yl)amino)ethyl)-4-(3-(3,4,5-trimethoxyphenyl)acryloyl)benzenesulfonamide (***24d***). Yellow solid. 79% yield; mp 112-113 °C. FT-IR (ATR): ν (cm^{-1}) 3377 (N-H), 3092 (=C-H), 1659 (C=O), 1579 and 1498 (C=N and C=C), 1155 (S=O). ^{1}H NMR (400 MHz, DMSO-d_6) δ ppm 2.95-3.07 (m, 8H, CH_3, CH_2), 3.24-3.35 (m, 3H, CH_2, NH), 3.73 (s, 3H, OCH_3), 3.86 (s, 6H, OCH_3), 6.79 (bs, 1H, NH), 6.97-7.08 (m, 2H, Ar-H), 7.23 (s, 2H, Ar-H), 7.67-7.76 (m, 3H, Ar-H, CH), 7.85 (d, J = 15.6 Hz, 1H, CH), 7.95 (d, J = 7.4 Hz, 2H, Ar-H), 8.27 (d, J = 7.4 Hz, 2H, Ar-H), 8.99 (bs, 1H, NH). ^{13}C NMR (100 MHz, DMSO-d_6) δ ppm 35.7 (CH_3), 40.1 (CH_2), 41.9 (CH_2), 56.1 (CH_3), 60.1 (CH_3), 106.8 (CH), 114.7 (d, $^2J_{CF}$ = 22.1 Hz, CH), 120.9 (d, $^3J_{CF}$ = 6.7 Hz, CH), 121.0 (CH), 126.8 (CH), 129.2 (CH), 130.0 (C), 136.8 (C), 140.1 (C), 140.4 (C), 145.5 (CH), 153.1 (C), 157.01 (d, $^1J_{CF}$ = 238.6 Hz, C), 161.6 (C), 163.6 (C), 164.8 (C), 165.1 (C), 188.4 (C). MS (70 eV) m/z (%): 651 (1), 591 (1), 368 (10), 313 (9), 279 (9), 261 (8), 168 (4), 149 (100). Anal. Calcd. for $C_{31}H_{34}FN_7O_6S$: C, 57.13; H, 5.26; N, 15.04; S, 4.92; Found: C, 57.08; H, 5.20; N, 15.01; S, 5.01.

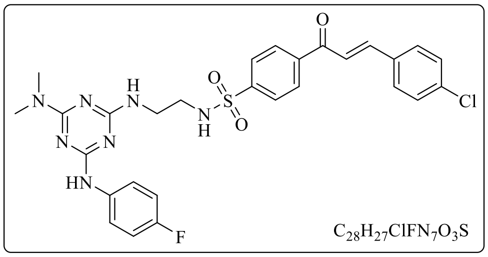

(E)-4-(3-(4-chlorophenyl)acryloyl)-N-(2-((4-(dimethylamino)-6-((4-fluorophenyl)amino)-1,3,5-triazin-2-yl)amino)ethyl)benzenesulfonamide (***24e***). White solid. 89% yield; mp 201-203 °C. FT-IR (ATR): ν (cm^{-1}) 3384 (N-H), 3096 (=C-H), 1655 (C=O), 1589 and 1504 (C=N and C=C), 1153 (S=O). ^{1}H NMR (400 MHz, DMSO-d_6) δ ppm 2.89 (bs, 2H, CH_2), 3.01 (s, 6H, CH_3), 3.23 (bs, 2H, CH_2), 3.50 (bs, 1H, NH), 6.48 (bs, 1H, NH), 7.03 (t, J = 7.3 Hz, 2H, Ar-H), 7.52 (d, J = 8.1 Hz, 2H, Ar-H), 7.68-7.85 (m, 5H, Ar-H, CH), 7.88-7.97 (m, 3H, Ar-H, CH), 8.14 (d, J = 8.1 Hz, 2H, Ar-H), 8.94 (bs, 1H, NH). ^{13}C NMR (100 MHz, DMSO-d_6) δ ppm 35.6 (CH_3), 42.0 (CH_2),

44.3 (CH_2), 114.6 (d, $^2J_{CF}$ = 23.1 Hz, CH), 120.7 (d, $^3J_{CF}$ = 6.9 Hz, CH), 122.8 (CH), 126.3 (CH), 128.5 (CH), 128.9 (CH), 130.6 (CH), 133.6 (C), 135.1 (C), 137.1 (C), 137.2 (C), 142.6 (CH), 156.84 (d, $^1J_{CF}$ = 237.8 Hz), 163.7 (C), 163.8 (C), 165.3 (C), 165.5 (C), 188.5 (C). MS (70 eV) m/z (%): 595:597 [M^+]:[M + 2]$^+$ (2/1), 473 (3), 413 (6), 290 (23), 261 (100), 245 (3). Anal. Calcd. for $C_{28}H_{27}ClFN_7O_3S$: C, 56.42; H, 4.57; N, 16.45; S, 5.38; Found: C, 56.39; H, 4.56; N, 16.40; S, 5.40.

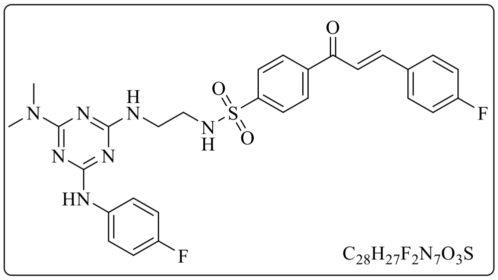

(E)-N-(2-((4-(dimethylamino)-6-((4-fluorophenyl)amino)-1,3,5-triazin-2-yl)amino)ethyl)-4-(3-(4-fluorophenyl)acryloyl)benzenesulfonamide ***(24f)***. White solid. 87% yield; mp 195-197 °C. FT-IR (ATR): ν (cm^{-1}) 3431 (N-H), 3109 (=C-H), 1662 (C=O), 1587 and 1507 (C=N and C=C), 1156 (S=O). ^{1}H NMR (400 MHz, DMSO-d_6) δ ppm 2.92 (bs, 2H, CH_2), 3.01 (s, 6H, CH_3), 3.25 (bs, 2H, CH_2), 3.62 (bs, 1H, NH), 6.54 (bs, 1H, NH), 6.97-7.09 (m, 2H, Ar-H), 7.29 (t, J = 8.4 Hz, 2H, Ar-H), 7.68-8.00 (m, 8H, Ar-H, CH), 8.11-8.22 (m, 2H, Ar-H), 8.93 (bs, 1H, NH). ^{13}C NMR (100 MHz, DMSO-d_6) δ ppm 35.6 (CH_3), 41.8 (CH_2), 44.2 (CH_2), 114.6 (d, ^2J = 22.1 Hz, CH), 115.9 (d, $^2J_{CF}$ = 21.3 Hz, CH), 120.7 (d, $^3J_{CF}$ = 6.8 Hz, CH), 121.9 (CH), 126.4 (CH), 128.7 (CH), 131.3 (d, $^3J_{CF}$ = 8.0 Hz, CH), 137.1 (C), 138.4 (C), 143.1 (CH), 149.2 (C), 156.85 (d, $^1J_{CF}$ = 237.6 Hz, C), 163.5 (d, $^1J_{CF}$ = 249.2 Hz, C), 163.7 (C), 165.2 (C), 165.3 (C), 165.5 (C), 188.5 (C). MS (70 eV) m/z (%): 579 (9), 290 (41), 261 (100), 233 (9), 137 (16), 109 (12). Anal. Calcd. for $C_{28}H_{27}F_2N_7O_3S$: C, 58.02; H, 4.70; N, 16.92; S, 5.53; Found: C, 57.99; H, 4.67; N, 16.98; S, 5.55.

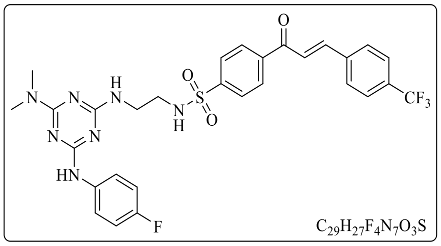

(E)-N-(2-((4-(dimethylamino)-6-((4-fluorophenyl)amino)-1,3,5-triazin-2-yl)amino)ethyl)4-(3-((trifluoromethyl)phenyl)acryloyl)benzenesulfonamide ***(24g)***. White solid. 83% yield; mp 219-221 °C. FT-IR (ATR): ν (cm^{-1}) 3389 (N-H), 3087 (=C-H), 1659 (C=O), 1581 and 1505 (C=N and C=C), 1153 (S=O). ^{1}H NMR (400 MHz, DMSO-d_6) δ ppm 2.89 (bs, 2H, CH_2), 3.01 (s, 6H, CH_3), 3.23 (bs, 2H, CH_2), 3.47 (bs, 1H, NH), 6.49 (bs, 1H, NH), 6.94-7.09 (m, 2H, Ar-H), 7.70-7.88 (m, 7H, Ar-H, CH), 8.04 (d, J = 15.7 Hz, 1H, CH), 8.10 (d, J = 7.9 Hz, 2H, Ar-H), 8.16 (d, J = 6.6 Hz, 2H, Ar-H), 8.95 (bs, 1H, NH). ^{13}C NMR (100 MHz, DMSO-d_6) δ ppm 35.6 (CH_3), 42.1 (CH_2), 44.6 (CH_2), 114.6 (d, $^2J_{CF}$ = 22.3 Hz, CH), 120.7 (d, $^3J_{CF}$ = 7.2 Hz, CH), 121.4 (C), 124.1 (q, $^1J_{CF}$ = 271.9 Hz, CF_3), 124.7 (CH), 125.7 (d, $^4J_{CF}$ = 3.0 Hz, CH), 126.5 (d, $^3J_{CF}$ = 9.0 Hz, CH), 128.7 (CH), 129.5 (CH), 130.1 (d, $^2J_{CF}$ = 31.6 Hz, C), 137.2 (C), 138.7 (C), 142.1 (CH), 156.90 (d, $^1J_{CF}$ = 250.2 Hz, C), 163.7 (C), 163.8 (C), 165.2 (C), 166.0 (C), 188.5 (C). MS (70 eV) m/z (%): 629 (7), 290 (49), 273 (11), 261 (100), 248 (12), 233 (12). Anal. Calcd. for $C_{29}H_{27}F_4N_7O_3S$: C, 55.32; H, 4.32; N, 15.57; S, 5.09; Found: C, 55.28; H, 4.35; N, 15.61; S, 5.02.

Characterization of triazinyloxy-diazepines (28a-g)

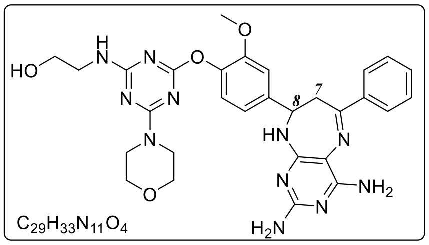

2-((4-(4-(2,4-diamino-6-phenyl-8,9-dihydro-7H-pyrimido[4,5-b][1,4]diazepin-8-yl)-2-methoxyphenoxy)-6-morpholino-1,3,5-triazin-2-yl)amino)ethane-1-ol ***(28a)***. Yellow solid. 57% yield; mp 97-100 °C. FT-IR (ATR): ν (cm^{-1}) 3474 (N-H), 3363 (O-H), 1580 and 1503 (C=N and C=C). ^1H NMR (400 MHz, DMSO-d_6) δ ppm 2.78 (d, J = 14.0 Hz, 1H, H-7), 3.04-3.12 (m, 1H, CH_2), 3.19-3.28 (m, 1H, CH_2), 3.33-3.65 (m, 13H, CH_2, OCH_3), 3.84-3.97 (m, 1H, H-7), 4.60 (bs, 1H, OH), 4.95-5.08 (m,1H, H-8), 5.74 (bs, 2H, NH_2), 6.24 (bs, 2H, NH_2), 6.66 (d, J = 7.6 Hz, 1H, Ar-H), 6.89 (d, J = 6.9 Hz, 1H, Ar-H), 6.97 (bs, 1H, NH), 7.09-7.33 (m, 5H, Ar-H, NH), 7.69 (t, J = 6.1 Hz, 2H, Ar-H). ^{13}C NMR (100 MHz, DMSO-d_6) δ ppm 38.7 (CH_2), 43.1 (CH_2), 43.3 (CH_2), 55.6 (CH_3), 56.6 (CH), 59.5 (CH_2), 65.9 (CH_2), 101.5 (C), 110.9 (CH), 118.0 (CH), 122.5 (CH), 126.2 (CH), 128.0 (CH), 128.2 (CH), 139.5 (C), 141.1 (C), 142.0 (C), 150.8 (C), 154.0 (C), 154.7 (C), 160.4 (C), 163.7 (C), 165.7 (C), 166.7 (C), 170.1 (C). MS (70 eV) *m/z* (%): 599 (3), 376 (70), 361 (32), 299 (7), 239 (10), 226 (100). Anal. Calcd. for $C_{29}H_{33}N_{11}O_4$: C, 58.09; H, 5.55; N, 25.69; Found: C, 58.11; H, 5.58; N, 25.73.

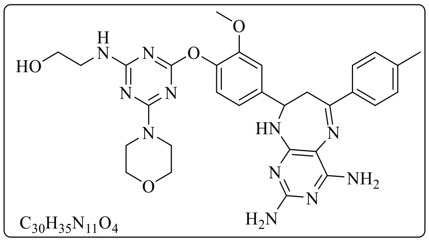

2-((4-(4-(2,4-diamino-6-(p-tolyl)-8,9-dihydro-7H-pyrimido[4,5-b][1,4]diazepin-8-yl)-2-methoxyphenoxy)-6-morpholino-1,3,5-triazin-2-yl)amino)ethan-1-ol ***(28b)***. Yellow solid. 53% yield; mp 197-199 °C. FT-IR (ATR): ν (cm^{-1}) 3482 (N-H), 3360 (O-H), 1565 and 1501 (C=N and C=C). ^1H NMR (400 MHz, DMSO-d_6) δ ppm 2.26 (s, 3H, CH_3), 2.70-2.80 (m, 1H, H-7), 3.04-3.13 (m, 1H, CH_2), 3.22-3.29 (m, 1H, CH_2), 3.30-3.67 (m, 13H, CH_2, OCH_3), 3.79-3.92 (m, 1H, H-7), 4.58 (bs, 1H, OH), 4.93-5.10 (m, 1H, H-8), 5.67 (bs, 2H, NH_2), 6.19 (bs, 2H, NH_2), 6.67 (d, J = 7.3 Hz, 1H, Ar-H), 6.88 (d, J = 6.5 Hz, 1H, Ar-H), 6.93-7.12 (m, 4H, Ar-H, NH), 7.14-7.23 (m, 1H, Ar-H), 7.54-7.64 (m, 2H, Ar-H). ^{13}C NMR (100 MHz, DMSO-d_6) δ ppm 20.7 (CH_3), 38.6 (CH_2), 43.1 (CH_2), 43.3 (CH_2), 55.6 (CH_3), 56.8 (CH), 59.5 (CH_2), 65.9 (CH_2), 101.5 (C), 111.0 (CH), 118.0 (CH), 122.4 (CH), 126.1 (CH), 128.6 (CH), 137.5 (C), 138.4 (C), 139.5 (C), 142.0 (C), 150.8 (C), 154.1 (C), 154.7 (C), 160.4 (C), 163.7 (C), 165.7 (C), 166.7 (C), 170.1 (C). MS (70 eV) *m/z* (%): 613 (3), 390 (86), 375 (39), 299 (7), 240 (100),166 (21). Anal. Calcd. for $C_{30}H_{35}N_{11}O_4$: C, 58.72; H, 5.75; N, 25.11; Found: C, 58.69; H, 5.70; N, 25.15.

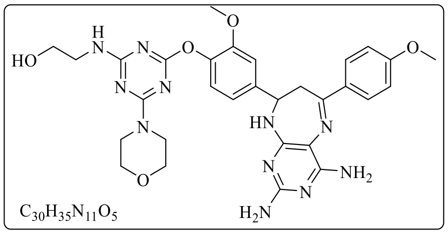

2-((4-(4-(2,4-diamino-6-(4-methoxyphenyl)-8,9-dihydro-7H-pyrimido[4,5-b][1,4]diazepin-8-yl)-2-methoxyphenoxy)-6-morpholino-1,3,5-triazin-2-yl)amino)ethan-1-ol ***(28c)****. Yellow solid*. 56% yield; mp 203-204 °C. FT-IR (ATR): ν (cm^{-1}) 3473 (N-H), 3327 (O-H), 1581 and 1501 (C=N and C=C). ^1H NMR (400 MHz, DMSO-d_6) δ ppm 2.72-2.79 (m, 1H, H-7), 3.03-3.13 (m, 1H, CH_2), 3.20-3.27 (m, 1H, CH_2), 3.29-3.69 (m, 13H, CH_2, OCH_3), 3.74 (s, 3H, OCH_3), 3.86 (dd, J = 12.0, 4.0 Hz, 1H, H-7), 4.60 (bs, 1H, OH), 4.95-5.05 (m, 1H, H-8), 5.65 (bs, 2H, NH_2), 6.17 (bs, 2H, NH_2), 6.66 (d, J = 8.0 Hz, 1H, Ar-H), 6.82 (d, J = 7.9 Hz, 2H, Ar-H), 6.86-6.91 (m, 1H, Ar-H), 6.96-

7.03 (m, 2H, NH), 7.17-7.26 (m, 1H, Ar-H), 7.67 (d, *J* = 7.9 Hz, 2H, Ar-H). ^{13}C NMR (100 MHz, DMSO-d_6) δ ppm 38.5 (CH_2), 43.1 (CH_2), 43.3 (CH_2), 55.2 (CH_3), 55.6 (CH_3), 56.9 (CH), 59.5 (CH_2), 65.9 (CH_2), 101.5 (C), 110.9 (CH), 113.3 (CH), 118.0 (CH), 122.4 (CH), 127.7 (CH), 133.8 (C), 139.5 (C), 142.0 (C), 150.8 (C), 154.0 (C), 154.6 (C), 159.5 (C), 160.3 (C), 163.6 (C), 165.7 (C), 166.7 (C), 170.1 (C). MS (70 eV) *m/z* (%): 629 (5), 406 (98), 391 (48), 256 (100), 241 (30), 178 (25). Anal. Calcd. for $C_{30}H_{35}N_{11}O_5$: C, 57.22; H, 5.60; N, 24.47; Found: C, 57.25; H, 5.58; N, 24.51.

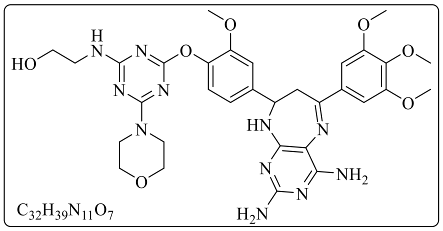

*2-((4-(4-(2,4-diamino-6-(3,4,5-trimethoxyphenyl)-8,9-dihydro-7H-pyrimido[4,5-b][1,4]diazepine-8-yl)-2-methoxyphenoxy)-6-morpholino-1,3,5-triazin-2-yl)amino)ethanol-1-ol (**28d**). Yellow solid*. 54% yield; mp 154-157 °C. FT-IR (ATR): ν (cm^{-1}) 3485 (N-H), 3359 (O-H), 1578 and 1501 (C=N and C=C). ^{1}H NMR (400 MHz, DMSO-d_6) δ ppm 2.76 (d, *J* = 14.0 Hz, 1H, H-7), 3.05-3.13 (m, 1H, CH_2), 3.20-3.28 (m, 1H, CH_2), 3.29-3.45 (m, 5H, OCH_3, CH_2), 3.51-3.66 (m, 11H, CH_2, OCH_3), 3.74 (s, 6H, OCH_3), 3.83 (dd, *J* = 14.0, 5.1 Hz, 1H, H-7), 4.59 (bs, 1H, OH), 5.02-5.11 (m, 1H, H-8), 5.69 (bs, 2H, NH_2), 6.18 (bs, 2H, NH_2), 6.68 (t, *J* = 8.4 Hz, 1H, Ar-H), 6.79 (s, 2H, Ar-H), 6.93 (t, *J* = 8.4 Hz, 1H, Ar-H), 7.00-7.08 (m, 2H, Ar-H, NH), 7.13 (bs, 1H, NH). ^{13}C NMR (100 MHz, DMSO-d_6) δ ppm 38.9 (CH_2), 43.1 (CH_2), 43.3 (CH_2), 55.5 (CH_3), 55.8 (CH_3), 57.9 (CH), 59.5 (CH_3), 60.1 (CH_2), 65.9 (CH_2), 101.4 (C), 103.8 (CH), 111.0 (CH), 118.1 (CH), 122.6 (CH), 137.1 (C), 137.9 (C), 139.5 (C), 142.5 (C), 150.8 (C), 152.3 (C), 154.8 (C), 160.4 (C), 163.6 (C), 165.4 (C), 165.8 (C), 166.7 (C), 170.2 (C). MS (70 eV) *m/z* (%): 689 (34), 466 (100), 451 (48), 316 (97), 301 (56), 124 (49). Anal. Calcd. for $C_{32}H_{39}N_{11}O_7$: C, 55.72; H, 5.70; N, 22.34; Found: C, 55.69; H, 5.71; N, 22.30.

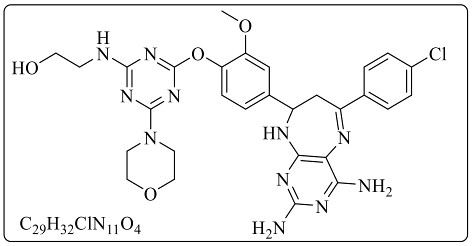

*2-((4-(4-(2,4-diamino-6-(4-chlorophenyl)-8,9-dihydro-7H-pyrimido[4,5-b][1,4]diazepin-8-yl)-2-methoxyphenoxy)-6-morpholino-1,3,5-triazin-2-yl)amino)ethanol-1-ol (**28e**). Yellow solid*. 70% yield; mp 130-131 °C. FT-IR (ATR): ν (cm^{-1}) 3489 (N-H), 3347 (O-H), 1581 and 1501 (C=N and C=C). ^{1}H NMR (400 MHz, DMSO-d_6) δ ppm 2.75 (d, *J* = 14.6 Hz, 1H, H-7), 3.04-3.12 (m, 1H, CH_2), 3.20-3.28 (m, 1H, CH_2), 3.38-3.74 (m, 13H, CH_2, OCH_3), 3.89 (dd, *J* = 14.6, 6.4 Hz, 1H, H-7), 4.58 (bs, 1H, OH), 4.95-5.07 (m, 1H, H-8), 5.72 (bs, 2H, NH_2), 6.23 (bs, 2H, NH_2), 6.64 (d, *J* = 8.1 Hz, 1H, Ar-H), 6.88 (d, *J* = 8.1 Hz, 1H, Ar-H), 6.92-6.99 (m, 1H, Ar-H), 7.08-7.21 (m, 2H, NH), 7.30 (d, *J* = 7.9 Hz, 2H, Ar-H), 7.72 (d, *J* = 7.9 Hz, 2H, Ar-H). ^{13}C NMR (100 MHz, DMSO-d_6) δ ppm 39.0 (CH_2), 43.5 (CH_2), 43.7 (CH_2), 56.1 (CH_3), 57.1 (CH), 59.9 (CH_2), 66.3 (CH_2), 101.9 (C), 111.3 (CH), 118.4 (CH), 122.9 (CH), 128.3 (CH), 128.4 (CH), 133.2 (C), 140.0 (C), 140.3 (C), 142.3 (C), 151.3 (C), 152.9 (C), 155.3 (C), 161.1 (C), 164.3 (C), 166.2 (C), 167.2 (C), 170.6 (C). MS (70 eV) *m/z* (%): 633: 635 [M^+]:[M + 2]$^+$ (25:10), 410 (41), 361 (20), 299 (7), 273 (20), 260 (100). Anal. Calcd. for $C_{29}H_{32}ClN_{11}O_4$: C, 54.93; H, 5.09; N, 24.30; Found: C, 54.89; H, 5.11; N, 24.27.

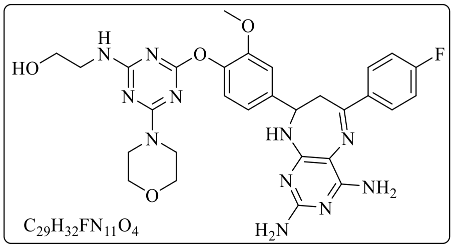

2-((4-(4-(2,4-diamino-6-(4-fluorophenyl)-8,9-dihydro-7H-pyrimido[4,5-b][1,4]diazepin-8-yl)-2-methoxyphenoxy)-6-morpholino-1,3,5-triazin-2-yl)amino)ethanol-1-ol ***(28f)****. Yellow solid*. 73% yield; mp 183-185 °C. FT-IR (ATR): ν (cm^{-1}) 3483 (N-H), 3335 (O-H), 1580 and 1501 (C=N and C=C). ^{1}H NMR (400 MHz, DMSO-d_6) δ ppm 2.69-2.80 (m, 1H, H-7), 3.03-3.11 (m, 1H, CH_2), 3.19-3.27 (m, 1H, CH_2), 3.46-3.76 (m, 13H, CH_2, OCH_3), 3.90 (dd, J = 14.1, 6.5 Hz, 1H, H-7), 4.61 (bs, 1H, OH), 4.93-5.07 (m, 1H, H-8), 5.70 (bs, 2H, NH_2), 6.20 (bs, 2H, NH_2), 6.64 (d, J = 7.9 Hz, 1H, Ar-H), 6.88 (d, J = 7.9 Hz, 1H, Ar-H), 6.96 (bs, 1H, NH), 7.02-7.12 (m, 3H, Ar-H, NH), 7.20 (t, J = 4.8 Hz, 1H, Ar-H), 7.68-7.80 (m, 2H, Ar-H). ^{13}C NMR (100 MHz, DMSO-d_6) δ ppm 38.6 (CH_2), 43.0 (CH_2), 43.3 (CH_2), 55.6 (CH_3), 59.5 (CH), 65.9 (CH_2), 66.4 (CH_2), 101.4 (C), 110.9 (CH), 114.5 (d, $^{2}J_{CF}$ = 22.0 Hz, CH), 117.9 (CH), 122.5 (CH), 128.1 (d, $^{3}J_{CF}$ = 8.4 Hz, CH), 129.1 (C), 133.6 (d, $^{4}J_{CF}$ = 4.5 Hz, C), 137.7 (C), 139.5 (C), 141.6 (C), 141.9 (C), 149.6 (C), 150.8 (C), 161.0 (C), 164.7 (d, $^{1}J_{CF}$ = 202.1 Hz, C), 166.7 (C), 170.1 (C). MS (70 eV) *m/z* (%): 617 (6), 394 (56), 379 (3), 299 (5), 244 (100). Anal. Calcd. for $C_{29}H_{32}FN_{11}O_4$: C, 56.39; H, 5.22; N, 24.95; Found: C, 56.42; H, 5.17; N, 24.89.

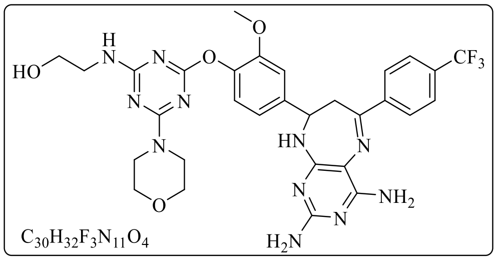

2-((4-(4-(2,4-diamino-6-(4-(trifluoromethyl)phenyl)-8,9-dihydro-7H-pyrimido[4,5-b][1,4]diazepine-8-yl)-2-methoxyphenoxy)-6-morpholino-1,3,5-triazin-2-yl)amino)ethane-1-ol ***(28g)****. Yellow solid*. 68% yield; mp 201-203 °C. FT-IR (ATR): ν (cm^{-1}) Not observed (N-H), 3368 (O-H), 1582 and 1502 (C=N and C=C). ^{1}H NMR (400 MHz, DMSO-d_6) δ ppm 2.79 (d, J = 14.3 Hz, 1H,H-7), 3.00-3.11 (m, 1H, CH_2), 3.20-3.27 (m, 1H, CH_2), 3.21-3.72 (m, 13H, CH_2, CH_3), 3.91-4.03 (m, 1H, H-7), 4.57 (bs, 1H, OH), 4.97-5.09 (m, 1H, H-8), 5.77 (bs, 2H, NH_2), 6.27 (bs, 2H, NH_2), 6.64 (d, J = 7.8 Hz, 1H, Ar-H), 6.89 (d, J = 7.8 Hz, 1H, Ar-H), 6.95 (bs, 1H, NH), 7.12-7.26 (m, 2H, Ar-H, NH), 7.59 (d, J = 7.2 Hz, 2H, Ar-H), 7.89 (d, J = 7.2 Hz, 2H, Ar-H). ^{13}C NMR (100 MHz, DMSO-d_6) δ ppm 38.7 (CH_2), 43.1 (CH_2), 43.2 (CH_2), 55.5 (CH_3), 56.3 (CH), 59.4 (CH_2), 65.9 (CH_2), 101.6 (C), 110.8 (CH), 117.8 (CH), 122.5 (CH), 123.5 (q, $^{1}J_{CF}$ = 250.0 Hz, CF_3), 124.8 (d, $^{4}J_{CF}$ = 4.8 Hz, CH), 125.7 (C), 126.7 (d, $^{3}J_{CF}$ = 1.6 Hz, CH) , 139.5 (C), 141.8 (C), 144.8 (C), 150.8 (C), 151.4 (d, $^{2}J_{CF}$ = 45.5 Hz, C), 155.0 (C), 160.8 (C), 164.0 (C), 165.7 (C), 166.7 (C), 170.1 (C). MS (70 eV) *m/z* (%): 667 (13), 444 (43), 429 (20), 361 (14), 294 (100), 166 (14). Anal. Calcd. for $C_{30}H_{32}F_3N_{11}O_4$: C, 53.97; H, 4.83; N, 23.08; Found: C, 54.01; H, 4.85; N, 23.12.

Characterization of triazinyloxy-diazepines (29a-g)

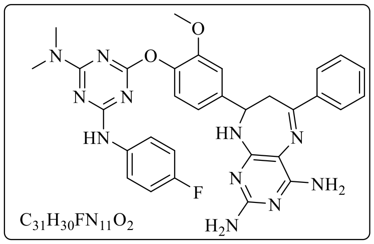

8-(4-((4-(dimethylamino)-6-((4-fluorophenyl)amino)-1,3,5-triazin-2-yl)oxy)-3-methoxyphenyl)-6-phenyl-8,9-dihydro-7H-pyrimido[4,5-b][1,4]diazepine-2,4-diamine ***(29a)****.* Yellow solid. 65% yield; mp 177-178 °C. FT-IR (ATR): ν (cm^{-1}) 3479 (N-H), 3106 (=C-H), 1537 and 1500 (C=N and C=C). ^{1}H NMR (400 MHz, DMSO-d_6) δ ppm 2.81 (d, J = 15.0 Hz, 1H, H-7), 2.86 (s, 3H, CH_3), 3.07 (s, 3H, CH_3), 3.54 (s, 3H, OCH_3), 3.93 (dd, J = 14.5, 5.8 Hz, 1H, H-7), 5.01-5.10 (m, 1H, H-8), 5.72 (bs, 2H, NH_2), 6.24 (bs, 2H, NH_2), 6.72 (d, J = 7.9 Hz, 1H, Ar-H), 6.96 (d, J = 8.1 Hz, 1H, Ar-

H), 7.03 (bs, 3H, Ar-H, NH), 7.12 (d, *J* = 5.0 Hz, 1H, Ar-H), 7.21-7.32 (m, 3H, Ar-H), 7.60 (bs, 2H, Ar-H), 7.71 (d, *J* = 6.4 Hz, 2H, Ar-H), 9.50 (bs, 1H, NH). ^{13}C NMR (100 MHz, DMSO-d_6) δ ppm 35.6 (CH_3), 36.0 (CH_3), 38.6 (CH_2), 55.6 (CH_3), 56.7 (CH), 101.5 (C), 110.9 (CH), 114.8 (d, $^2J_{CF}$ = 23.6 Hz, CH), 118.0 (CH), 121.3 (d, $^3J_{CF}$ = 7.3 Hz, CH), 122.3 (CH), 126.1 (CH), 127.9 (CH), 128.1 (CH), 136.0 (d, $^4J_{CF}$ = 2.2 Hz, C), 139.5 (C), 141.1 (C), 142.1(C), 150.8 (C), 154.7 (C), 155.08 (d, $^1J_{CF}$ = 234.7 Hz, C), 158.6 (C), 160.4 (C), 163.8 (C), 164.8 (C), 166.1 (C), 170.0 (C). MS (70 eV) m/z (%): 607 (2), 577 (21), 503 (42), 393 (70), 313 (30), 110 (21). Anal. Calcd. for $C_{31}H_{30}FN_{11}O_2$: C, 61.28; H, 4.98; N, 25.36; Found: C, 61.32; H, 4.95; N, 25.33.

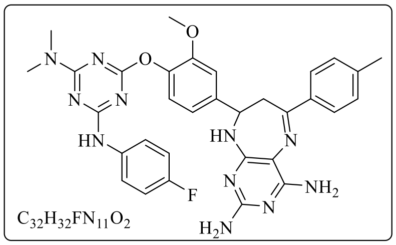

8-(4-((4-(dimethylamino)-6-((4-fluorophenyl)amino)-1,3,5-triazin-2-yl)oxy)-3-methoxyphenyl)-6-(p-tolyl))-8,9-dihydro-7H-pyrimido[4,5-b][1,4]diazepine-2,4-diamine (***29b***). Yellow solid. 68% yield; mp 180-182 °C. FT-IR (ATR): ν (cm^{-1}) 3381 (N-H), 3108 (=C-H), 1541 and 1500 (C=N and C=C). ^{1}H NMR (400 MHz, DMSO-d_6) δ ppm 2.26 (s, 3H, CH_3), 2.78 (d, *J* = 14.4 Hz, 1H, H-7), 2.86 (s, 3H, CH_3), 3.07 (s, 3H, CH_3), 3.57 (s, 3H, OCH_3), 3.89 (dd, *J* = 14.4, 6.1 Hz, 1H, H-7), 5.00-5.08 (m, 1H, H-8), 5.67 (bs, 2H, NH_2), 6.18 (bs, 2H, NH_2), 6.71 (d, *J* = 8.1 Hz, 1H, Ar-H), 6.95 (d, *J* = 8.1 Hz, 1H, Ar-H), 6.98-7.11 (m, 6H, Ar-H, NH), 7.57 (bs, 2H, Ar-H), 7.61 (d, *J* = 8.2 Hz, 2H, Ar-H), 9.50 (bs, 1H, NH). ^{13}C NMR (100 MHz, DMSO-d_6) δ ppm 21.2 (CH_3), 36.1 (CH_3), 36.5 (CH_3), 39.1 (CH_2), 56.0 (CH_3), 57.3 (CH), 101.9 (C), 111.4 (CH), 115.3 (d, $^2J_{CF}$ = 24.4 Hz, CH), 118.5 (CH), 121.8 (d, $^3J_{CF}$ = 7.5 Hz, CH), 122.8 (CH), 126.6 (CH), 127.5 (C), 129.1 (CH), 136.5 (d, $^4J_{CF}$ = 2.2 Hz, C), 138.0 (C), 138.9 (C), 140.0 (C), 142.7 (C), 151.2 (C), 154.5 (C), 155.2 (C), 160.8 (C), 165.2 (C), 165.4 (d, $^1J_{CF}$ = 250.3 Hz, C), 167.1 (C), 170.5 (C). MS (70 eV) m/z (%): 621 (63), 606 (10), 311 (9), 375 (9), 390 (14), 240 (100). Anal. Calcd. for $C_{32}H_{32}FN_{11}O_2$: C, 61.82; H, 5.19; N, 24.78; Found: C, 61.79; H, 5.22; N, 24.80.

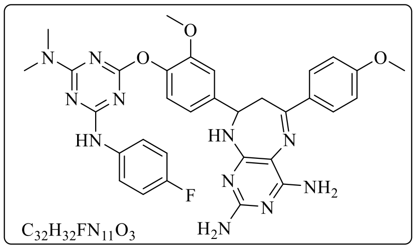

8-(4-((4-(dimethylamino)-6-((4-fluorophenyl)amino)-1,3,5-triazin-2-yl)oxy)-3-methoxyphenyl)-6-(4-methoxyphenyl))-8,9-dihydro-7H-pyrimido[4,5-b][1,4]diazepine-2,4-diamine (***29c***). Yellow solid. 69% yield; mp 190-192 °C. FT-IR (ATR): ν (cm^{-1}) 3472 (N-H), 3105 (=C-H), 1542 and 1501 (C=N and C=C). ^{1}H NMR (400 MHz, DMSO-d_6) δ ppm 2.78 (d, *J* = 14.3 Hz, 1H, H-7), 2.87 (s, 3H, CH_3), 3.07 (s, 3H, CH_3), 3.57 (s, 3H, OCH_3), 3.74 (s, 3H. OCH_3), 3.87 (dd, *J* = 14.6, 6.2 Hz, 1H, H-7), 5.00-5.08 (m, 1H, H-8), 5.66 (bs, 2H, NH_2), 6.17 (bs, 2H, NH_2), 6.72 (d, *J* = 8.0 Hz, 1H, Ar-H), 6.81 (d, *J* = 8.8 Hz, 2H, Ar-H), 6.96 (d, *J* = 8.0 Hz, 1H, Ar-H), 6.98-7.09 (m, 4H, Ar-H, NH), 7.60 (bs, 2H, Ar-H), 7.68 (d, *J* = 8.8 Hz, 2H, Ar-H), 9.50 (bs, 1H, NH). ^{13}C NMR (100 MHz, DMSO-d_6) δ ppm 35.6 (CH_3), 36.0 (CH_3), 38.5 (CH_2), 55.1 (CH_3), 55.6 (CH_3), 57.0 (CH), 101.4 (C), 110.8 (CH), 113.3 (CH), 114.8 (d, $^2J_{CF}$ = 27.5 Hz, CH), 118.0 (CH), 121.3 (d, $^3J_{CF}$ = 6.1 Hz, CH), 122.2 (C), 122.3 (CH), 127.6 (CH), 133.7 (C), 136.0 (d, $^4J_{CF}$ = 2.5 Hz, C), 139.5 (C), 142.1 (C), 150.7 (C), 154.1 (C), 154.4 (C), 158.6 (C), 159.5 (C), 160.0 (C), 164.7 (C), 164.8 (d, $^1J_{CF}$ = 272.3 Hz, C), 170.0 (C). MS (70 eV) m/z (%): 637 (93), 622 (16), 406 (12), 305 (11), 381 (7), 256 (100). Anal. Calcd. for $C_{32}H_{32}FN_{11}O_3$: C, 60.27; H, 5.06; N, 24.16; Found: C, 60.33; H, 5.02; N, 24.10.

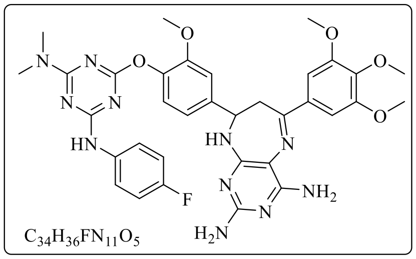

*8-(4-((4-(dimethylamino)-6-((4-fluorophenyl)amino)-1,3,5-triazin-2-yl)oxy)-3-methoxyphenyl)-6-(3,4),5-trimethoxyphenyl)-8,9-dihydro-7H-pyrimido[4,5-b][1,4]diazepine-2,4-diamine (**29d**)*. Yellow solid. 62% yield; mp 188-189 °C. FT-IR (ATR): ν (cm^{-1}) 3474 (N-H), 3108 (=C-H), 1542 and 1500 (C=N and C=C). ^{1}H NMR (400 MHz, DMSO-d_6) δ ppm 2.78 (d, J = 14.0 Hz, 1H, H-7), 2.87 (s, 3H, CH_3), 3.07 (s, 3H, CH_3), 3.56 (s, 3H, OCH_3), 3.63 (s, 3H, OCH_3), 3.74 (s, 6H, OCH_3), 3.88 (dd, J = 14.0, 5.4 Hz, 1H, H-7), 5.07-5.14 (m, 1H, H-8), 5.72 (bs, 2H, NH_2), 6.20 (bs, 2H, NH_2), 6.72 (d, J = 7.9 Hz, 1H, Ar-H), 6.83 (s, 2H, Ar-H), 6.99 (d, J = 8.2 Hz, 1H, Ar-H), 7.01 (bs, 2H, Ar-H), 7.08 (bs, 1H, NH), 7.12 (d, J = 5.3 Hz, 1H, Ar-H), 7.60 (bs, 2H, Ar-H), 9.46 (bs, 1H, NH). ^{13}C NMR (100 MHz, DMSO-d_6) δ ppm 35.58 (CH_3), 36.1 (CH_3), 38.9 (CH_2), 55.5 (OCH_3), 55.7 (OCH_3), 57.7 (OCH_3), 60.0 (CH), 101.3 (C), 103.8 (CH), 110.9 (CH), 111.0 (CH), 114.8 (d, $^2J_{CF}$ = 23.9 Hz, CH), 118.0 (CH), 121.3 (d, $^3J_{CF}$ = 10.1 Hz, CH), 122.4 (CH), 136.0 (d, $^4J_{CF}$ = 2.2 Hz, C), 137.0 (C), 138.0 (C), 139.6 (C), 142.5 (C), 150.8 (C), 152.3 (C), 154.6 (C), 154.7 (C), 156.2 (C), 160.1 (C), 164.7 (C), 164.8 (d, $^1J_{CF}$ = 277.1 Hz, C), 170.0 (C). MS (70 eV) m/z (%): 697 (35), 682 (6), 466 (93), 451 (45), 381 (12), 316 (76). Anal. Calcd. for $C_{34}H_{36}FN_{11}O_5$: C, 58.53; H, 5.20; N, 22.08; Found: C, 58.49; H, 5.17; N, 22.05.

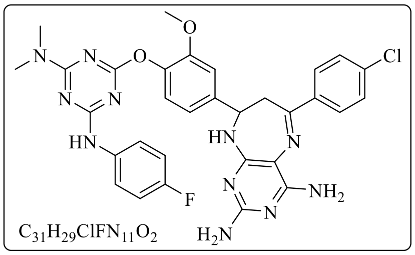

*6-(4-chlorophenyl)-8-(4-((4-(dimethylamino)-6-((4-fluorophenyl)amino)-1,3,5-triazin-2-yl)oxy)-3-methoxyphenyl)-8,9-dihydro-7H-pyrimido[4,5-b][1,4]diazepine-2,4-diamine (**29e**)*. Yellow solid. 70% yield; mp 168-170 °C. FT-IR (ATR): ν (cm^{-1}) 3516 (N-H), 3140 (=C-H), 1543 and 1501 (C=N and C=C). ^{1}H NMR (400 MHz, DMSO-d_6) δ ppm 2.76 (d, J = 14.5 Hz, 1H, H-7), 2.84 (s, 3H, CH_3), 3.07 (s, 3H, CH_3), 3.57 (s, 3H, OCH_3), 3.93 (dd, J = 14.5, 6.0 Hz, 1H, H-7), 5.00-5.09 (m, 1H, H-8), 5.70 (bs, 2H, NH_2), 6.21 (bs, 2H, NH_2), 6.68 (d, J = 8.1 Hz, 1H, Ar-H), 6.96 (d, J = 8.1 Hz, 1H, Ar-H), 7.02 (bs, 3H, Ar-H, NH), 7.14 (d, J = 5.7 Hz, 1H, Ar-H), 7.28 (d, J = 8.5 Hz, 2H, Ar-H), 7.61 (bs, 2H, Ar-H), 7.73 (d, J = 8.5 Hz, 2H, Ar-H), 9.50 (bs, 1H, NH). NMR ^{13}C (100 MHz, DMSO-d_6) δ ppm 35.5 (CH_3), 36.0 (CH_3), 38.4 (CH_2), 55.6 (CH_3), 56.7 (CH), 101.4 (C), 110.9 (C), 114.8 (d, $^2J_{CF}$ = 22.8 Hz, CH), 117.9 (CH), 121.3 (d, $^3J_{CF}$ = 15.4 Hz, CH), 122.3 (CH), 127.8 (CH), 127.9 (CH), 132.7 (C), 136.0 (C), 139.5 (C), 139.8 (C), 142.0 (C), 150.8 (C), 152.4 (C), 154.9 (C), 156.2 (C), 158.6 (C), 160.6 (C), 164.7 (C), 165.0 (d, $^1J_{CF}$ = 231.2 Hz, C), 170.0 (C). MS (70 eV) m/z (%): 641: 643 $[M^+]:[M + 2]^+$ (59/23), 410 (11), 382 (19), 393 (3), 260 (100), 233 (17). Anal. Calcd. for $C_{31}H_{29}ClFN_{11}O_2$: C, 57.99; H, 4.55; N, 24.00; Found: C, 58.02; H, 4.50; N, 24.04.

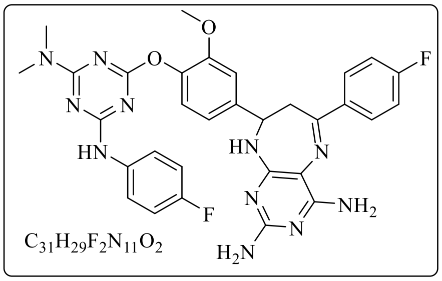

*8-(4-((4-(dimethylamino)-6-((4-fluorophenyl)amino)-1,3,5-triazin-2-yl)oxy)-3-methoxyphenyl)-6-(4-fluorophenyl))-8,9-dihydro-7H-pyrimido[4,5-b][1,4]diazepine-2,4-diamine (**29f**)*. Yellow solid. 71% yield; mp 151-153 °C. FT-IR (ATR): ν (cm^{-1}) 3258 (N-H), 3119 (=C-H), 1556 and 1501 (C=N and C=C).1H NMR (400 MHz, DMSO-d_6) δ ppm 2.79 (d, *J* = 14.5 Hz, 1H, H-7), 2.86 (s, 3H, CH_3), 3.07 (s, 3H, CH_3), 3.56 (s, 3H, OCH_3), 3.92 (dd, *J* = 14.5, 5.5 Hz, 1H, H-7), 5.00-5.10 (m, 1H, H-8), 5.73 (bs, 2H, NH_2), 6.25 (bs, 2H, NH_2), 6.70 (d, *J* = 7.7 Hz, 1H, Ar-H), 6.96 (d, *J* = 8.1 Hz, 1H, Ar-H), 6.99-7.17 (m, 6H, Ar-H, NH), 7.61 (bs, 2H, Ar-H), 7.71-7.82 (m, 2H, Ar-H), 9.51 (bs, 1H, NH). ^{13}C NMR (100 MHz, DMSO-d_6) δ ppm 35.6 (CH_3), 36.0 (CH_3), 38.1 (CH_2), 55.6 (CH_3), 56.8 (CH), 101.4 (C), 110.8 (CH), 114.6 (d, $^2J_{CF}$ = 20.3 Hz, CH), 114.8 (d, $^2J_{CF}$ = 21.7 Hz, CH), 117.9 (CH), 121.3 (d, $^3J_{CF}$ = 10.2 Hz, CH), 122.3 (CH), 128.3 (d, $^3J_{CF}$ = 8.9 Hz, CH) , 131.7 (C), 136 (d, $^4J_{CF}$ = 2.3 Hz, C), 137.6 (d, $^4J_{CF}$ = 2.9 Hz, C), 139.5 (C), 142.0 (C), 150.8 (C), 154.7 (C), 157.4 (d, $^1J_{CF}$ = 238.5 Hz, C), 160.3 (C), 162.2 (d, $^1J_{CF}$ = 245.9 Hz, C), 163.7 (C), 164.8 (C), 166.1 (C), 170.0 (C). MS (70 eV) m/z (%): 625 (77), 577 (29), 382 (18), 313 (32), 264 (27), 244 (100). Anal. Calcd. for $C_{31}H_{29}F_2N_{11}O_2$: C, 59.51; H, 4.67; N, 24.63; Found: C, 59.48; H, 4.60; N, 24.59.

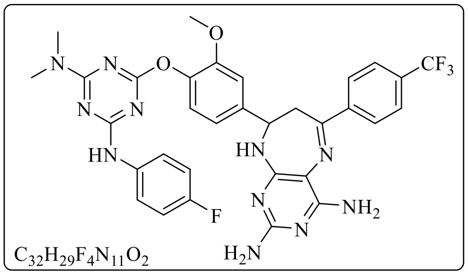

*8-(4-((4-(dimethylamino)-6-((4-fluorophenyl)amino)-1,3,5-triazin-2-yl)oxy)-3-methoxyphenyl)-6-(4-(trifluoromethyl)phenyl)-8,9-dihydro-7H-pyrimido[4,5-b][1,4]diazepine-2,4-diamine (**29g**)*. Yellow solid. 70% yield; mp 179-181 °C. FT-IR (ATR): ν (cm^{-1}) 3387 (N-H), 3065 (=C-H), 1541 and 1500 (C=N and C=C). ^{1}H NMR (400 MHz, DMSO-d_6) δ ppm 2.75-2.80 (m, 3H, H-7, CH_3), 3.06 (s, 3H, CH_3), 3.55 (s, 3H, OCH_3), 4.00 (dd, *J* = 14.7, 5.8 Hz, 1H, H-7), 5.03-5.12 (m, 1H, H-8), 5.75 (bs, 2H, NH_2), 6.26 (bs, 2H, NH_2), 6.68 (d, *J* = 7.7 Hz, 1H, Ar-H), 6.95 (d, *J* = 8.2 Hz, 1H, Ar-H), 7.03 (bs, 3H, Ar-H, NH), 7.25 (d, *J* = 5.2 Hz, 1H, Ar-H), 7.43-7.73 (m, 4H, Ar-H), 7.90 (d, *J* = 8.1 Hz, 2H, Ar-H), 9.50 (bs, 1H, NH). ^{13}C NMR (100 MHz, DMSO-d_6) δ ppm 35.5 (CH_3), 35.9 (CH_3), 38.7 (CH_2), 55.6 (CH_3), 56.4 (CH), 101.6 (C), 110.8 (CH), 114.8 (d, $^2J_{CF}$ = 21.6 Hz, CH), 117.8 (CH), 121.3 (d, $^3J_{CF}$ = 10.5 Hz, CH), 122.4 (CH), 124.4 (d, $^1J_{CF}$ = 270.9 Hz, CF_3), 124.8 (d, $^3J_{CF}$ = 6.3 Hz, CH), 126.7 (CH), 127.8 (d, $^2J_{CF}$ = 32.0 Hz, C) , 136.0 (d, $^4J_{CF}$ = 2.6 Hz, C), 139.5 (C), 142.0 (C), 144.8 (C), 150.8 (C), 151.6 (C), 155.0 (C), 156.2 (C), 160.8 (C), 164.8 (C), 165.1 (d, $^1J_{CF}$ = 210.3 Hz, C), 170.0 (C). MS (70 eV) m/z (%): 675 (73), 444 (9), 382 (38), 338 (14), 294 (100), 233 (19). Anal. Calcd. for $C_{32}H_{29}F_4N_{11}O_2$: C, 56.89; H, 4.33; N, 22.80; Found: C, 56.93; H, 4.29; N, 22.78.

Characterization of triazinylamino-diazepines (30a-g)

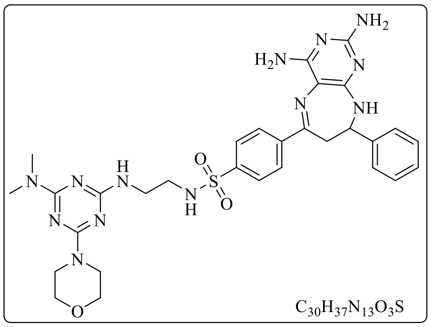

*4-(2,4-diamino-8-phenyl-8,9-dihydro-7H-pyrimido[4,5-b][1,4]diazepin-6-yl)-N-(2-((4-(dimethylamino)-6-morpholino-1,3,5-triazin-2-yl)amino)ethyl)benzenesulfonamide (**30a**).* Yellow solid. 74% yield; mp 186-188 °C. FT-IR (ATR): ν (cm^{-1}) 3485 and 3366 (N-H), 2970 (C-H), 1562 and 1529 (C=N and C=C), 1153 (S=O). ^1H NMR (400 MHz, DMSO-d_6) δ ppm 2.79 (d, J = 14.2 Hz, 1H, H-7), 2.81-2.90 (m, 2H, CH_2), 2.97 (s, 6H, CH_3), 3.20 (bs, 2H, CH_2), 3.31-3.66 (m, 8H, CH_2), 3.81 (dd, J = 14.2, 5.8 Hz, 1H, H-7), 5.02-5.10 (m, 1H, H-8), 5.78 (bs, 2H, NH_2), 6.29 (bs, 2H, NH_2), 6.62 (bs, 1H, NH), 7.05 (t, J = 6.4 Hz, 1H, Ar-H), 7.10-7.20 (m, 5H, Ar-H, NH), 7.57 (d, J = 8.3 Hz, 2H, Ar-H), 7.66 (bs, 1H, NH), 7.75 (d, J = 8.3 Hz, 2H, Ar-H). ^{13}C NMR (100 MHz, DMSO-d_6) δ ppm 35.5 (CH_3), 38.9 (CH_2), 39.9 (CH_2), 42.0 (CH_2), 43.2 (CH_2), 57.1 (CH), 66.1 (CH_2), 101.6 (C), 125.7 (CH), 126.0 (CH), 126.5 (CH), 126.7 (CH), 128.0 (CH), 134.0 (C), 139.1 (C), 143.9 (C), 144.3 (C), 151.9 (C), 155.0 (C), 157.3 (C), 160.7 (C), 163.9 (C), 165.5 (C). MS (70 eV) m/z (%): 659 (79), 556 (19), 328 (82), 266 (93), 237 (100), 104 (98). Anal. Calcd. for $C_{30}H_{37}N_{13}O_3S$: C, 54.61; H, 5.65; N, 27.60; S, 4.86; Found: C, 54.59; H, 5.60; N, 27.57; S, 4.83.

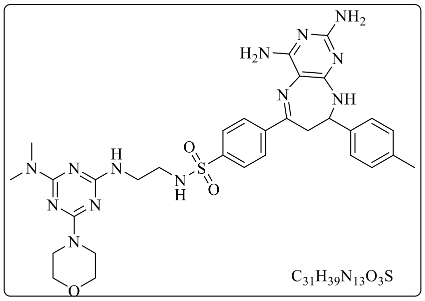

*4-(2,4-diamino-8-(p-tolyl)-8,9-dihydro-7H-pyrimido[4,5-b][1,4]diazepin-6-yl)-N-(2-((4-(dimethylamino)-6-morpholino-1,3,5-triazin-2-yl)amino)ethyl)benzenesulfonamide (**30b**).* Yellow solid. 72% yield; mp >300 °C. FT-IR (ATR): ν (cm^{-1}) 3508 and 3392 (N-H), 2972 (C-H), 1589 and 1527 (C=N and C=C), 1151 (S=O). ^1H NMR (400 MHz, DMSO-d_6) δ ppm 2.13 (s, 3H, CH_3), 2.78 (d, J = 14.3 Hz, 1H, H-7), 2.86 (bs, 2H, CH_2), 2.97 (s, 6H, CH_3), 3.21 (bs, 2H, CH_3), 3.50-3.65 (m, 8H, CH_2), 3.80 (dd, J = 14.3, 5.1 Hz, 1H, H-7), 5.03-5.11 (m, 1H, H-8), 5.78 (bs, 2H, NH_2), 6.29 (bs, 2H, NH_2), 6.62 (bs, 1H, NH), 6.97 (d, J = 7.4 Hz, 2H, Ar-H), 7.03 (d, J = 7.4 Hz, 2H, Ar-H), 7.11 (bs, 1H, NH), 7.59 (d, J = 7.8 Hz, 2H, Ar-H), 7.67 (bs, 1H, NH), 7.79 (d, J = 7.8 Hz, 2H, Ar-H). ^{13}C NMR (100 MHz, DMSO-d_6) δ ppm 20.5 (CH_3), 35.5 (CH_3), 39.1 (CH_2), 39.9 (CH_2), 42.0 (CH_2), 43.2 (CH_2), 56.5 (CH), 66.0 (CH_2), 101.5 (C), 125.7 (CH), 126.1 (CH), 126.5 (CH), 128.6 (CH), 135.6 (C), 139.1 (C), 140.9 (C), 144.4 (C), 151.7 (C), 155.0 (C), 160.7 (C), 163.9 (C), 164.6 (C), 165.2 (C), 165.5 (C). MS (70 eV) m/z (%): 673 (62), 627 (14), 342 (86), 266 (94), 237 (100), 207 (31). Anal. Calcd. for $C_{31}H_{39}N_{13}O_3S$: C, 55.26; H, 5.83; N, 27.02; S, 4.76; Found: C, 55.29; H, 5.80; N, 27.00; S, 4.77.

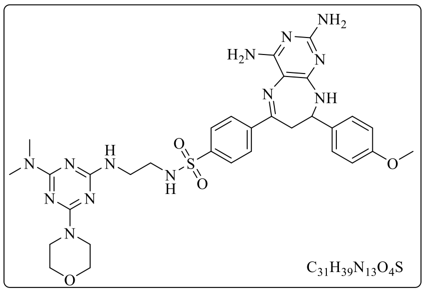

*4-(2,4-diamino-8-(4-methoxyphenyl)-8,9-dihydro-7H-pyrimido[4,5-b][1,4]diazepin-6-yl)-N-(2-((4-(dimethylamino)-6-morpholino-1,3,5-triazin-2-yl)amino)ethyl)benzenesulfonamide (**30c**).* Yellow solid. 78% yield; mp 169-171 °C. FT-IR (ATR): ν (cm^{-1}) 3471 and 3370 (N-H), 2930 (C-H), 1589 and 1523 (C=N and C=C), 1154 (S=O). ^{1}H NMR (400 MHz, DMSO-d_6) δ ppm 2.79 (d, *J* = 14.0 Hz, 1H, H-7), 2.82-2.90 (m, 2H, CH_2), 2.97 (s, 6H, CH_3), 3.17-3.26 (m, 2H, CH_2), 3.51-3.64 (m, 11H, CH_2, OCH_3), 3.75 (dd, *J* = 14.0, 5.4 Hz, 1H, H-7), 4.93-5.00 (m, 1H, H-8), 5.78 (bs, 2H, NH_2), 6.30 (bs, 2H, NH_2), 6.62 (bs, 1H, NH), 6.74 (d, *J* = 8.6 Hz, 2H, Ar-H), 7.07 (d, *J* = 8.6 Hz, 3H, Ar-H, NH), 7.60 (d, *J* = 8.4 Hz, 2H, Ar-H), 7.67 (bs, 1H, NH), 7.79 (d, *J* = 8.4 Hz, 2H, Ar-H). ^{13}C NMR (100 MHz, $CDCl_3$) δ ppm 36.3 (CH_3), 40.4 (CH_2), 41.8 (CH_2), 43.8 (CH_2), 45.5 (CH_2), 55.4 (CH_3), 57.7 (CH), 66.9 (CH_2), 102.6 (C), 114.4 (CH), 126.8 (CH), 127.0 (CH), 127.3 (CH), 134.9 (C), 139.5 (C), 144.5 (C), 154.2 (C), 154.8 (C), 159.4 (C), 160.4 (C), 164.2 (C), 164.8 (C), 165.3 (C), 166.8 (C). MS (70 eV) m/z (%): 689 (28), 627 (13), 556 (33), 358 (56), 266 (90), 237 (100). Anal. Calcd. for $C_{31}H_{39}N_{13}O_4S$: C, 53.98; H, 5.70; N, 26.40; S, 4.65; Found: C, 53.95; H, 5.67; N, 26.37; S, 4.69.

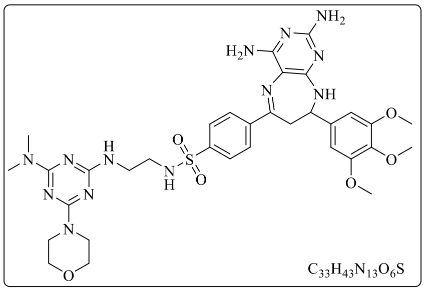

*4-(2,4-diamino-8-(3,4,5-trimethoxyphenyl)-8,9-dihydro-7H-pyrimido[4,5-b][1,4]diazepin-6-yl)-N -(2-((4-(dimethylamino)-6-morpholino-1,3,5-triazin-2-yl)amino)ethyl)benzenesulfonamide (**30d**).* Yellow solid. 65% yield; mp 118-120 °C. FT-IR (ATR): ν (cm^{-1}) 3508 and 3392 (N-H), 2972 (C-H), 1589 and 1527 (C=N and C=C), 1151 (S=O). ^{1}H NMR (400 MHz, DMSO-d_6) δ ppm 2.79 (d, *J* = 14.3 Hz, 1H, H-7), 2.84 (bs, 2H, CH_2), 2.97 (s, 6H, CH_3), 3.22 (bs, 2H, CH_2), 3.48 (s, 3H, OCH_3), 3.56 (bs, 14H, CH_2, OCH_3), 3.81 (dd, *J* = 14.3, 6.1 Hz, 1H, H-7), 4.90-4.99 (m, 1H, H-8), 5.77 (bs, 2H, NH_2), 6.27 (bs, 2H, NH_2), 6.46 (s, 2H, Ar-H), 6.63 (bs, 1H, NH), 7.03 (bs, 1H, NH), 7.62 (d, *J* = 8.4 Hz, 2H, Ar-H), 7.68 (bs, 1H, NH), 7.85 (d, *J* = 8.4 Hz, 2H, Ar-H). ^{13}C NMR (100 MHz, DMSO-d_6) δ ppm 35.4 (CH_3), 38.8 (CH_2), 39.9 (CH_2), 42.0 (CH_2), 43.2 (CH_2), 55.7 (CH_3), 57.3 (CH), 59.9 (CH_2), 66.0 (CH_2), 101.7 (C), 103.7 (CH), 126.1 (CH), 126.6 (CH), 136.3 (C), 139.2 (C), 139.5 (C), 144.2 (C), 152.3 (C), 152.5 (C), 154.7 (C), 160.7 (C), 163.9 (C), 164.6 (C), 165.2 (C), 165.5 (C). MS (70 eV) m/z (%): 749 (8), 556 (29), 418 (17), 266 (51), 237 (72), 194 (100). Anal. Calcd. for $C_{33}H_{43}N_{13}O_6S$: C, 52.86; H, 5.78; N, 24.28; S, 4.28; Found: C, 52.80; H, 5.80; N, 24.31; S, 4.30.

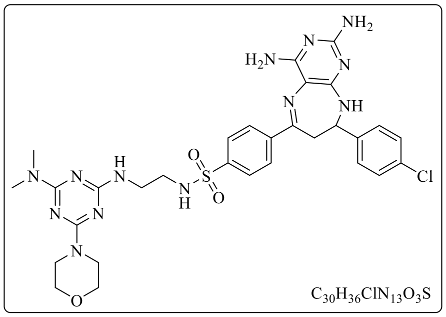

*4-(2,4-diamino-8-(4-chlorophenyl)-8,9-dihydro-7H-pyrimido[4,5-b][1,4]diazepin-6-yl)-N-(2-((4-(dimethylamino)-6-morpholino-1,3,5-triazin-2-yl)amino)ethyl)benzenesulfonamide (**30e**)*. Yellow solid. 77% yield; mp >300 °C. FT-IR (ATR): ν (cm^{-1}) 3504 and 3388 (N-H), 2951 (C-H), 1590 and 1527 (C=N and C=C), 1150 (S=O). ^{1}H NMR (400 MHz, DMSO-d_6) δ ppm 2.76 (d, *J* = 14.2 Hz, 1H, H-7), 2.85 (bs, 2H, CH_2), 2.96 (s, 6H, CH_3), 3.17-3.26 (m, 2H, CH_2), 3.51-3.63 (m, 8H, CH_2), 3.86 (dd, *J* = 14.2, 5.8 Hz, 1H, H-7), 5.03-5.08 (m, 1H, H-8), 5.76 (bs, 2H, NH_2), 6.26 (bs, 2H, NH_2), 6.62 (bs, 1H, NH), 7.15 (d, *J* = 8.5 Hz, 2H, Ar-H), 7.18 (bs, 1H, NH), 7.22 (d, *J* = 8.5 Hz, 2H, Ar-H), 7.59 (d, *J* = 8.4 Hz, 2H, Ar-H), 7.72 (bs, 1H, NH), 7.78 (d, *J* = 8.4 Hz, 2H, Ar-H). ^{13}C NMR (100 MHz, DMSO-d_6) δ ppm 35.5 (CH_3), 38.6 (CH_2), 39.7 (CH_2), 42.0 (CH_2), 43.2 (CH_2), 56.4 (CH), 66.0 (CH_2), 101.6 (C), 126.1 (CH), 126.5 (CH), 127.7 (CH), 127.9 (CH), 131.1 (C), 139.2 (C), 142.9 (C), 144.2 (C), 151.5 (C), 155.0 (C), 160.9 (C), 164.0 (C), 164.6 (C), 165.2 (C), 165.5 (C). MS (70 eV) m/z (%): 693:695 [M^{+}]:[M + 2]$^{+}$ (16/5), 556 (12), 362 (17), 266 (77), 237 (100), 225 (37), 138 (47). Anal. Calcd. for $C_{30}H_{36}ClN_{13}O_3S$: C, 51.90; H, 5.23; N, 26.23; S, 4.62; Found: C, 51.88; H, 5.19; N, 26.28; S, 4.58.

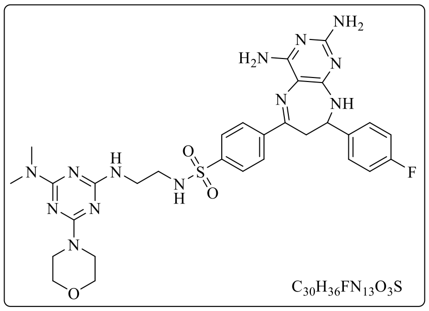

*4-(2,4-diamino-8-(4-fluorophenyl)-8,9-dihydro-7H-pyrimido[4,5-b][1,4]diazepin-6-yl)-N-(2-((4-(dimethylamino)-6-morpholino-1,3,5-triazin-2-yl)amino)ethyl)benzenesulfonamide (**30f**)*. Yellow solid. 70% yield; mp >300 °C. FT-IR (ATR): ν (cm^{-1}) 3485 and 3384 (N-H), 2956 (C-H), 1589 and 1524 (C=N and C=C), 1153 (S=O). ^{1}H NMR (400 MHz, DMSO-d_6) δ ppm 2.75 (d, *J* = 14.1 Hz, 1H, H-7), 2.81-2.90 (m, 2H, CH_2), 2.97 (s, 6H, CH_3), 3.22 (q, *J* = 6.0 Hz, 2H, CH_2), 3.51-3.63 (m, 8H, CH_2), 3.82 (dd, *J* = 14.7, 5.6 Hz, 1H, H-7), 5.07 (t, *J* = 5.6 Hz, 1H, H-8), 5.80 (bs, 2H, NH_2), 6.31 (bs, 2H, NH_2), 6.62 (bs, 1H, NH), 6.99 (t, *J* = 8.8 Hz, 2H, Ar-H), 7.13-7.21 (m, 3H, Ar-H, NH), 7.59 (d, *J* = 8.4 Hz, 2H, Ar-H), 7.67 (bs, 1H, NH), 7.77 (d, *J* = 8.4 Hz, 2H, Ar-H). ^{13}C NMR (100 MHz, DMSO-d_6) δ ppm 35.4 (CH_3), 39.1 (CH_2), 40.1 (CH_2), 42.0 (CH_2), 43.2 (CH_2), 56.5 (CH), 66.0 (CH_2), 101.6 (C), 114.7 (d, $^{2}J_{CF}$ = 19.7 Hz, CH), 126.1 (CH), 126.5 (CH), 127.7 (d, $^{3}J_{CF}$ = 8.3 Hz, CH), 139.2 (C), 140.2 (C), 144.2 (C), 151.8 (C), 154.9 (C), 159.7 (C), 160.7 (C), 163.9 (C), 163.3 (d, $^{1}J_{CF}$ = 252.0 Hz, C), 164. 7 (C), 165.5 (C). MS (70 eV) m/z (%): 677 (48), 556 (20), 346 (38), 266 (81), 237 (100), 122 (95). Anal. Calcd. for $C_{30}H_{36}FN_{13}O_3S$: C, 53.16; H, 5.35; N, 26.87; S, 4.73; Found: C, 53.13; H, 5.30; N, 26.91; S, 4.76.

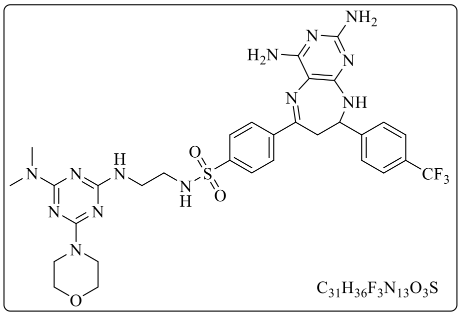

*4-(2,4-diamino-8-(4-(trifluoromethyl)phenyl)-8,9-dihydro-7H-pyrimido[4,5-b][1,4]diazepin-6-yl)-N-(2-((4-(dimethylamino)-6-morpholino-1,3,5-triazin-2-yl)amino)ethyl)benzenesulfonamide (**30g**)*. Yellow solid. 73% yield; mp >300 °C. FT-IR (ATR): ν (cm^{-1}) 3505 and 3387 (N-H), 2953 (C-H), 1589 and 1523 (C=N and C=C), 1151 (S=O). ^1H NMR (400 MHz, DMSO-d_6) δ ppm 2.81 (d, J = 14.1 Hz, 3H, H-7, CH_2), 2.96 (s, 6H, CH_3), 3.21 (q, J = 5.9 Hz, 2H, CH_2), 3.50-3.63 (m, 8H, CH_2), 3.91 (dd, J = 14.1, 5.9 Hz, 1H, H-7), 5.13-5.20 (m, 1H, H-8), 5.79 (bs, 2H, NH_2), 6.28 (bs, 2H, NH_2), 7.25 (bs, 1H, NH), 7.36 (d, J = 8.1 Hz, 2H, Ar-H), 7.57 (d, J = 8.1 Hz, 2H, Ar-H), 7.58 (d, J = 8.5 Hz, 3H, Ar-H, NH), 7.76 (d, J = 8.6 Hz, 3H, Ar-H, NH). ^{13}C NMR (100 MHz, DMSO-d_6) δ ppm 35.5 (CH_3), 38.5 (CH_2), 39.9 (CH_2), 42.0 (CH_2), 43.2 (CH_2), 56.7 (CH), 66.0 (CH_2), 101.7 (C), 124.9 (d, $^3J_{CF}$ = 16.0 Hz, CH), 126.1 (CH), 126.2 (q, $^1J_{CF}$ = 261.2 Hz, CF_3), 126.4 (CH), 126.6 (CH), 127.5 (C), 129.0 (d, $^2J_{CF}$ = 58.0 Hz, C) 139.3 (C), 144.1 (C), 148.4 (C), 151.4 (C), 155.0 (C), 160.9 (C), 164.0 (C), 164.7 (C), 165.5 (C). MS (70 eV) m/z (%): 727 (23), 659 (18), 328 (21), 266 (66), 237 (91), 193 (33). Anal. Calcd. for $C_{31}H_{36}F_3N_{13}O_3S$: C, 51.16; H, 4.99; N, 25.02; S, 4.41; Found: C, 51.18; H, 4.98; N, 25.07; S, 4.43.

Characterization of triazinylamino-diazepines (31a-g)

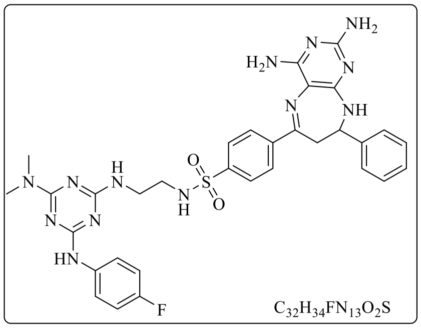

*4-(2,4-diamino-8-phenyl-8,9-dihydro-7H-pyrimido[4,5-b][1,4]diazepin-6-yl)-N-(2-((4-(dimethylamino)-6-((4-fluorophenyl)amino)-1,3,5-triazin-2-yl)amino)ethyl)benzenesulfonamide (**31a**)*. Yellow solid. 65% yield; mp 172-174 °C. FT-IR (ATR): ν (cm^{-1}) 3369 (N-H), 2919 (C-H), 1584 and 1497 (C=N and C=C), 1153 (S=O). ^1H NMR (400 MHz, DMSO-d_6) δ ppm 2.77 (d, J = 14.4 Hz, 1H, H-7), 2.88 (bs, 2H, CH_2), 3.02 (s, 6H, CH_3), 3.26 (bs, 2H, CH_2), 3.80 (dd, J = 14.4, 4.7 Hz, 1H, H-7), 5.01-5.08 (m, 1H, H-8), 5.76 (bs, 2H, NH_2), 6.26 (bs, 2H, NH_2), 6.75 (bs, 1H, NH), 7.00-7.08 (m, 3H, Ar-H), 7.10-7.18 (m, 5H, Ar-H, NH), 7.58 (d, J = 8.3 Hz, 2H, Ar-H), 7.69 (bs, 1H, NH), 7.71-7.80 (m, 4H, Ar-H), 8.91 (bs, 1H, NH). ^{13}C NMR (100 MHz, DMSO-d_6) δ ppm 35.7 (CH_3), 38.9 (CH_2), 39.9 (CH_2), 42.2 (CH_2), 56.0 (CH), 101.6 (C), 114.68 (d, $^2J_{CF}$ = 24.2 Hz, CH), 120.8 (d, $^3J_{CF}$ = 7.5 Hz, CH), 125.7 (CH), 126.1 (CH), 126.5 (CH), 126.6 (CH), 128.0 (CH), 137.04 (d, $^4J_{CF}$ = 2.2 Hz, C), 139.1 (C), 144.0 (C), 144.3 (C), 151.8 (C), 155.1 (C), 155.8 (C), 158.1 (C), 162.29 (d, $^1J_{CF}$ = 294.0 Hz, C), 164.0 (C), 165.1 (C), 165.3 (C). MS (70 eV) m/z (%): 683 (9), 580 (8), 328 (29), 290 (23), 261 (56), 104 (100). Anal. Calcd. for $C_{32}H_{34}FN_{13}O_2S$: C, 56.21; H, 5.01; N, 26.63; S, 4.69; Found: C, 56.19; H, 4.99; N, 26.69; S, 4.73.

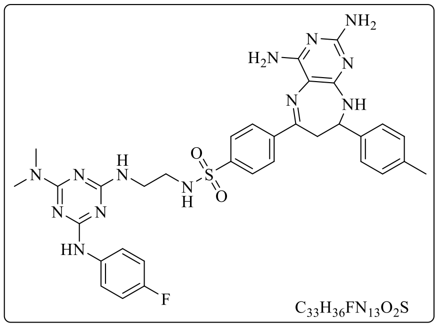

*4-(2,4-diamino-8-(p-tolyl)-8,9-dihydro-7H-pyrimido[4,5-b][1,4]diazepin-6-yl)-N-(2-((4-(dimethylamino)-6-((4-fluorophenyl)amino)-1,3,5-triazin-2-yl)amino)ethyl)benzenesulfonamide (**31b**)*. Yellow solid. 63% yield; mp 162-164 °C. FT-IR (ATR): ν (cm^{-1}) 3370 (N-H), 2922 (C-H), 1583 and 1497 (C=N and C=C), 1153 (S=O). ^{1}H NMR (400 MHz, DMSO-d_6) δ ppm 2.12 (s, 3H, CH_3), 2.76 (d, J = 14.1 Hz, 1H, H-7), 2.88 (bs, 2H, CH_2), 3.02 (s, 6H, CH_3), 3.27 (bs, 2H, CH_2), 3.77 (d, J = 14.1 Hz, 1H, H-7), 4.93-5.00 (m, 1H, H-8), 5.75 (bs, 2H, NH_2), 6.26 (bs., 2H, NH_2), 6.76 (bs, 1H, NH), 6.96 (d, J = 7.8 Hz, 2H, Ar-H), 7.01 (d, J = 7.8 Hz, 2H, Ar-H), 7.03-7.11 (m, 3H, Ar-H, NH), 7.60 (d, J = 8.1 Hz, 2H, Ar-H), 7.63-7.80 (m, 5H, Ar-H, NH), 8.91 (bs, 1H, NH). ^{13}C NMR (100 MHz, DMSO-d_6) δ ppm 20.4 (CH_3), 35.6 (CH_3), 38.9 (CH_2), 39.1 (CH_2), 41.9 (CH_2), 56.5 (CH), 101.6 (C), 114.7 (d, $^2J_{CF}$ = 23.6 Hz, CH), 120.8 (d, $^3J_{CF}$ = 7.4 Hz, CH), 125.6 (CH), 126.1 (CH), 126.5 (CH), 128.6 (CH), 133.9 (C), 135.6 (C), 137.0 (C), 137.1 (d, $^4J_{CF}$ = 2.3 Hz, C), 141.0 (C), 144.4 (C), 151.6 (C), 155.1 (C), 155.8 (C), 162.3 (d, $^1J_{CF}$ = 295.5 Hz, C), 164.0 (C), 165.1 (C), 166.3 (C). MS (70 eV) m/z (%): 697 (5), 579 (11), 342 (16), 290 (35), 261 (71), 117 (100). Anal. Calcd. for $C_{33}H_{36}FN_{13}O_2S$: C, 56.80; H, 5.20; N, 26.10; S, 4.59; Found: C, 56.78; H, 5.17; N, 26.12; S, 4.64.

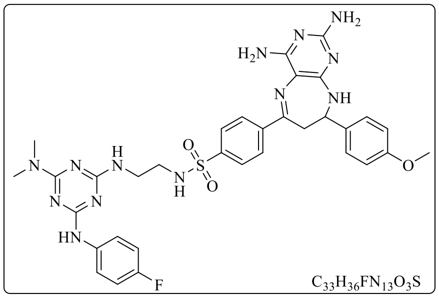

*4-(2,4-diamino-8-(4-methoxyphenyl)-8,9-dihydro-7H-pyrimido[4,5-b][1,4]diazepin-6-yl)-N-(2-((4-(dimethylamino)-6-((4-fluorophenyl)amino)-1,3,5-triazin-2-yl)amino)ethyl)benzenesulfonamide (**31c**)*. Yellow solid. 70% yield; mp 186-187 °C. FT-IR (ATR): ν (cm^{-1}) 3369 (N-H), 2930 (C-H), 1585 and 1501 (C=N and C=C), 1153 (S=O). ^{1}H NMR (400 MHz, DMSO-d_6) δ ppm 2.77 (d, J = 14.4 Hz, 1H, H-7), 2.90 (bs, 2H, CH_2), 3.02 (s, 6H, CH_3), 3.28 (bs, 2H, CH_2), 3.60 (s, 3H, OCH_3), 3.65-3.79 (m, 1H, H-7), 4.91-5.00 (m, 1H, H-8), 5.76 (bs, 2H, NH_2), 6.27 (bs, 2H, NH_2), 6.73 (d, J = 8.5 Hz, 2H, Ar-H), 6.76 (bs, 1H NH), 6.99-7.10 (m, 5H, Ar-H, NH), 7.61 (d, J = 8.4 Hz, 2H, Ar-H), 7.65-7.83 (m, 5H, Ar-H, NH), 8.91 (bs, 1H, NH). ^{13}C NMR (100 MHz, DMSO-d_6) δ ppm 35.6 (CH_3), 38.9 (CH_2), 39.9 (CH_2), 41.9 (CH_2), 54.9 (OCH_3), 56.3 (CH), 101.5 (C), 113.4 (CH), 114.7 (d, $^2J_{CF}$ = 22.4 Hz, CH), 120.8 (d, $^3J_{CF}$ = 7.1 Hz, CH), 126.1 (CH), 126.5 (CH), 126.9 (CH), 136.1 (C), 137.1 (d, $^4J_{CF}$ = 2.1 Hz, C), 139.1 (C), 144.4 (C), 151.7 (C), 155.0 (C), 155.8 (C), 158.1 (C), 159.3 (d, $^1J_{CF}$ = 287.7 Hz, C), 163.7 (C), 164.0 (C), 165.1 (C), 165.3 (C). MS (70 eV) m/z (%): 713 (2), 580 (8), 358 (12), 290 (15), 261 (42), 134 (100). Anal. Calcd. for $C_{33}H_{36}FN_{13}O_3S$: C, 55.53; H, 5.08; N, 25.51; S, 4.49; Found: C, 55.48; H, 5.09; N, 25.56; S, 4.47.

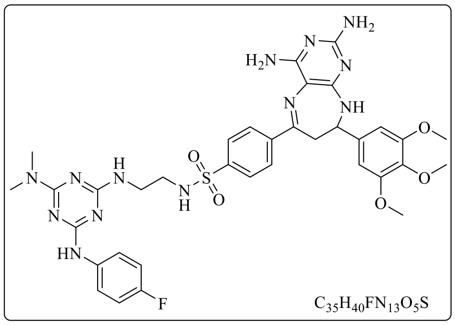

*(2,4-diamino-8-(3,4,5-trimethoxyphenyl)-8,9-dihydro-7H-pyrimido[4,5-b][1,4]diazepin-6-yl)-N-(2-((4-(dimethylamino)-6-((4-fluorophenyl)amino)-1,3,5-triazin-2-yl)amino)ethyl)benzenesulfonamide (**31d**)*. Yellow solid. 55% yield; mp 180-183 °C. FT-IR (ATR): ν (cm^{-1}) 3370 (N-H), 2968 (C-H), 1587 and 1502 (C=N and C=C), 1154 (S=O). ^{1}H NMR (400 MHz, DMSO-d_6) δ ppm 2.77 (d, *J* = 14.0 Hz, 1H, H-7), 2.88 (bs, 2H, CH_2), 3.02 (s, 6H, CH_3), 3.27 (bs, 2H, CH_2), 3.49 (s, 3H, OCH_3), 3.56 (s, 6H, OCH_3), 3.79 (d, *J* = 14.0 Hz, 1H, H-7), 4.90-4.97 (m, 1H, H-8), 5.75 (bs, 2H, NH_2), 6.25 (bs, 2H, NH_2), 6.45 (s, 2H, Ar-H), 6.76 (bs, 1H, NH), 6.99-7.10 (m, 3H, Ar-H, NH), 7.63 (d, *J* = 7.9 Hz, 2H, Ar-H), 7.67-7.77 (m, 3H, Ar-H, NH), 7.84 (d, *J* = 7.9 Hz, 2H, Ar-H), 8.90 (bs, 1H, NH). ^{13}C NMR (100 MHz, DMSO-d_6) δ ppm 35.7 (CH_3), 38.7 (CH_2), 39.8 (CH_2), 55.7 (CH_3), 57.3 (CH), 59.9 (CH_3), 101.7 (C), 103.6 (CH), 114.7 (d, $^{2}J_{CF}$ = 24.0 Hz, CH), 120.8 (d, $^{3}J_{CF}$ = 7.6 Hz, CH), 122.5 (C), 126.1 (CH), 126.6 (CH), 136.3 (C), 137.0 (d, $^{4}J_{CF}$ = 1.3 Hz, C), 139.5 (C), 144.3 (C), 147.7 (C), 152.5 (C), 153.5 (d, $^{1}J_{CF}$ = 255.2 Hz C), 155.8 (C), 160.8 (C), 162.3 (C), 162.4 (C), 163.8 (C), 163.9 (C), 165.1 (C). MS (70 eV) m/z (%): 773 (2), 621 (6), 580 (15), 290 (32), 261 (100), 140 (49). Anal. Calcd. for $C_{35}H_{40}FN_{13}O_5S$: C, 54.32; H, 5.21; N, 23.53; S, 4.14; Found: C, 54.29; H, 5.19; N, 23.55; S, 4.16.

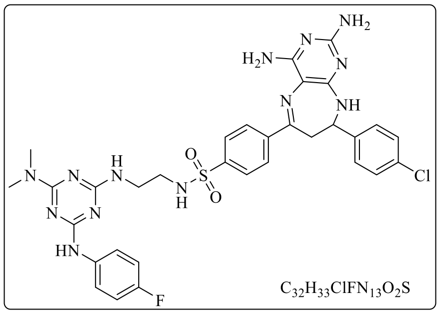

*4-(2,4-diamino-8-(4-chlorophenyl)-8,9-dihydro-7H-pyrimido[4,5-b][1,4]diazepin-6-yl)-N-(2-((4-(dimethylamino)-6-((4-fluorophenyl)amino)-1,3,5-triazin-2-yl)amino)ethyl)benzenesulfonamide (**31e**)*. Yellow solid. 67% yield; mp 148-150 °C. FT-IR (ATR): ν (cm^{-1}) 3370 (N-H), 2970 (C-H), 1583 and 1501 (C=N and C=C), 1153 (S=O). ^{1}H NMR (400 MHz, DMSO-d_6) δ ppm 2.75 (d, *J* = 14.5 Hz, 1H, H-7), 2.89 (bs, 2H, CH_2), 3.02 (s, 6H, CH_3), 3.28 (bs, 2H, CH_2), 3.78-3.88 (m, 1H, H-7), 5.00-5.09 (m, 1H, H-8), 5.77 (bs, 2H, NH_2), 6.27 (bs, 2H, NH_2), 6.75 (bs, 1H, NH), 7.00-7.09 (m, 2H, Ar-H), 7.13 (d, *J* = 8.2 Hz, 2H, Ar-H), 7.16-7.24 (m, 3H, Ar-H, NH), 7.61 (d, *J* = 8.2 Hz, 2H, Ar-H), 7.64-7.80 (m, 5H, Ar-H, NH), 8.90 (bs, 1H, NH). ^{13}C NMR (100 MHz, DMSO-d_6) δ ppm 35.7 (CH_3), 38.6 (CH_2), 39.7 (CH_2), 41.9 (CH_2), 56.4 (CH), 101.6 (C), 114.7 (d, $^{2}J_{CF}$ = 21.5 Hz, CH), 120.8 (d, $^{3}J_{CF}$ = 7.4 Hz, CH), 126.1 (CH), 126.5 (CH), 127.7 (CH), 127.9 (CH), 131.2 (C), 137.1 (d, $^{4}J_{CF}$ = 3.8 Hz, C), 142.9 (C), 144.2 (C), 151.5 (C), 155.0 (C), 155.8 (C), 158.1 (C), 162.3 (d, $^{1}J_{CF}$ = 291.3 Hz, C), 164.0 (C), 165.1 (C), 165.3 (C), 165.4 (C). MS (70 eV) m/z (%): 717:719 $[M^+]:[M + 2]^+$ (3/1), 580 (6), 362 (11), 290 (28), 262 (68), 138 (100), 103 (87). Anal. Calcd. for $C_{32}H_{33}ClFN_{13}O_2S$: C, 53.52; H, 4.63; N, 25.35; S, 4.46; Found: C, 53.51; H, 4.66; N, 25.31; S, 4.43.

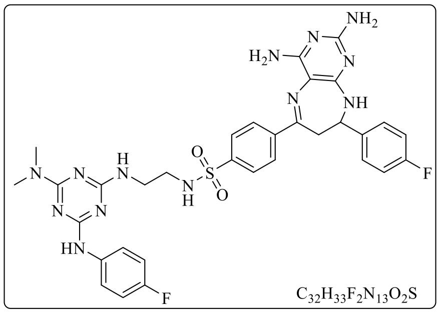

*4-(2,4-diamino-8-(4-fluorophenyl)-8,9-dihydro-7H-pyrimido[4,5-b][1,4]diazepin-6-yl)-N-(2-((4-(dimethylamino)-6-((4-fluorophenyl)amino)-1,3,5-triazin-2-yl)amino)ethyl)benzenesulfonamide (**31f**).* Yellow solid. 65% yield; mp 169-171 °C. FT-IR (ATR): ν (cm^{-1}) 3370 (N-H), 2971 (C-H), 1584 and 1502 (C=N and C=C), 1154 (S=O). ^1H NMR (400 MHz, DMSO-d_6) δ ppm 2.75 (d, J = 14.4 Hz, 1H, H-7), 2.89 (bs, 2H, CH_2), 3.02 (s, 6H, CH_3), 3.27 (bs, 2H, CH_2), 3.79 (dd, J = 14.4, 4.0 Hz, 1H, H-7), 4.99-5.09 (m, 1H, H-8), 5.75 (bs, 2H, NH_2), 6.25 (bs, 2H, NH_2), 6.74 (bs, 1H, NH), 6.98 (t, J = 8.5 Hz, 2H, Ar-H), 7.05 (t, J = 8.5 Hz, 2H, Ar-H), 7.11-7.18 (m, 3H, Ar-H, NH), 7.60 (d, J = 8.0 Hz, 2H, Ar-H), 7.63- 7.79 (m, 5H, Ar-H, NH), 8.90 (bs, 1H, NH). ^{13}C NMR (100 MHz, DMSO-d_6) δ ppm 35.6 (CH_3), 38.8 (CH_2), 39.9 (CH_2), 42.2 (CH_2), 56.5 (CH), 101.6 (C), 114.7 (d, $^2J_{CF}$ = 21.7 Hz, CH), 120.8 (d, $^3J_{CF}$ = 7.6 Hz, CH), 126.1 (CH), 126.5 (CH), 127.7 (d, $^2J_{CF}$ = 24.5 Hz, CH), 127.8 (d, $^3J_{CF}$ = 8.0 Hz, CH), 137.0 (d, $^4J_{CF}$ = 2.8 Hz, C), 140.20 (d, $^4J_{CF}$ = 2.7 Hz, C), 142.9 (C), 144.3 (C), 151.7 (C), 154.4 (C), 154.9 (C), 160.85 (d, $^1J_{CF}$ = 241.3 Hz, C), 162.30 (d, $^1J_{CF}$ = 290.5 Hz, C), 164.0 (C), 165.1 (C), 166.3 (C), 168. 8 (C). MS (70 eV) m/z (%): 701 (10), 580 (8), 346 (32), 290 (19), 261 (57), 122 (100). Anal. Calcd. for $C_{32}H_{33}F_2N_{13}O_2S$: C, 54.77; H, 4.74; N, 25.95; S, 4.57; Found: C, 54.73; H, 4.79; N, 25.99; S, 4.61.

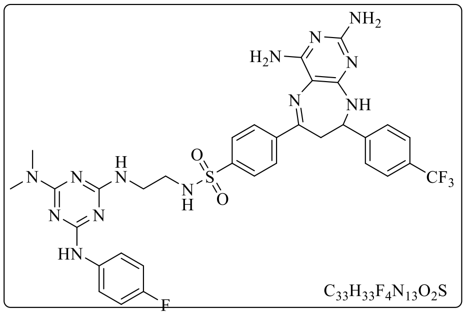

*4-(2,4-diamino-8-(4-(trifluoromethyl)phenyl)-8,9-dihydro-7H-pyrimido[4,5-b][1,4]diazepin-6-yl)-N-(2-((4-(dimethylamino)-6-((4-fluorophenyl)amino)-1,3,5-triazin-2-yl)amino)ethyl)benzenesulfonamide (**31g**).* Yellow solid. 63% yield; mp 180-183 °C. FT-IR (ATR): ν (cm^{-1}) 3370 (N-H), 2972 (C-H), 1585 and 1503 (C=N and C=C), 1154 (S=O). ^1H NMR (400 MHz, DMSO-d_6) δ ppm 2.80 (d, J = 14.0 Hz, 1H, H-7), 2.87 (bs, 2H, CH_2), 3.02 (s, 6H, CH_3), 3.27 (bs, 2H, CH_2), 3.88 (d, J = 14.0 Hz, 1H, H-7), 5.09-5.20 (m, 1H, H-8), 5.80 (bs, 2H, NH_2), 6.28 (bs, 2H, NH_2), 6.74 (bs, 1H, NH), 7.00-7.10 (m, 2H, Ar-H), 7.25 (bs, 1H, NH), 7.34 (d, J = 8.0 Hz, 2H, Ar-H), 7.53 (d, J = 8.0 Hz, 2H, Ar-H), 7.59 (d, J = 8.3 Hz, 2H, Ar-H), 7.70 (bs, 1H, NH), 7.71-7.80 (m, 4H, Ar-H), 8.91 (bs, 1H, NH). ^{13}C NMR (100 MHz, DMSO-d_6) δ ppm 35.6 (CH_3), 38.5 (CH_2), 39.7 (CH_2), 41.9 (CH_2), 56.7 (CH), 101.7 (C), 114.6 (d, $^2J_{CF}$ = 21.2 Hz, CH), 120.8 (d, $^3J_{CF}$ = 6.0 Hz, CH), 126.1 (CH), 124.2 (q, $^1J_{CF}$ = 272.1 Hz, CF_3), 126.1 (d, $^3J_{CF}$ = 3.0 Hz, CH), 126.4 (CH), 126.6 (CH), 127.3 (d, $^2J_{CF}$ = 31.5 Hz, C), 131.9 (C), 137.1 (d, $^4J_{CF}$ = 5.8 Hz, C), 144.1 (C), 148.4 (C), 151.4 (C), 155.0 (C), 155.8 (C), 158.1 (C), 162.3 (d, $^1J_{CF}$ = 287.4 Hz, C), 164.0 (C), 165.1 (C), 165.3 (C). MS (70 eV) m/z (%): 751 (19), 580 (14), 396 (33), 290 (38), 261 (100), 172 (36). Anal. Calcd. for $C_{33}H_{33}F_4N_{13}O_2S$: C, 52.72; H, 4.42; N, 24.22; S, 4.26; Found: C, 52.70; H, 4.39; N, 24.18; S, 4.29.

Characterization of triazinylamino-diazepines (32a-g)

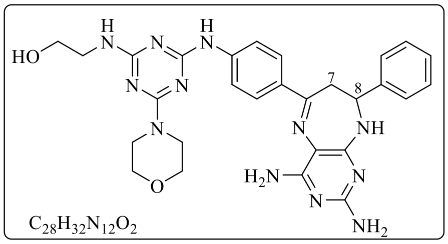

2-((4-((4-(2,4-diamino-8-phenyl-8,9-dihydro-7H-pyrimido[4,5-b][1,4]diazepin-6-yl)phenyl)amino)-6-morpholino-1,3,5-triazin-2-yl)amino)ethanol-1-ol ***(32a)***. Yellow solid. 75% yield; mp 200-203 °C. FT-IR (ATR): ν (cm^{-1}) 3465 (N-H), 3346 (O-H), 1585 and 1497 (C=N and C=C). ^{1}H NMR (400 MHz, DMSO-d_6) δ ppm 2.81 (d, *J* = 14.2 Hz, 1H, H-7), 3.46-3.54 (m, 4H,CH_2), 3.57-3.76 (m, 9H, CH_2, H-7), 4.66 (bs, 1H, OH), 4.99-5.08 (m, 1H, H-8), 5.92 (bs, 2H, NH_2), 6.38 (bs, 2H, NH_2), 6.81 (bs, 1H, NH), 7.05-7.15 (m, 2H, Ar-H, NH), 7.15-7.25 (m, 4H, Ar-H), 7.55 (d, *J* = 7.7 Hz, 2H, Ar-H), 7.61 (d, *J* = 8.5 Hz, 2H, Ar-H), 9.06 (bs, 1H, NH). ^{13}C NMR (100 MHz, DMSO-d_6) δ ppm 38.6 (CH_2), 42.9 (CH_2), 43.3 (CH_2), 57.7 (CH), 60.0 (CH_2), 66.0 (CH_2), 101.1 (C), 118.5 (CH), 125.9 (CH), 126.5 (CH), 126.7 (CH), 128.0 (CH), 133.7 (C), 140.9 (C), 143.9 (C), 153.8 (C), 155.2 (C), 158.6 (C), 162.6 (C), 164.0 (C), 164.8 (C), 165.8 (C). MS (70 eV) *m/z* (%): 568(26), 524 (15), 465 (11), 140 (27), 104 (85). Anal. Calcd. for $C_{28}H_{32}N_{12}O_2$: C, 59.14; H, 5.67; N, 29.56; Found: C, 59.09; H, 5.70; N, 29.59.

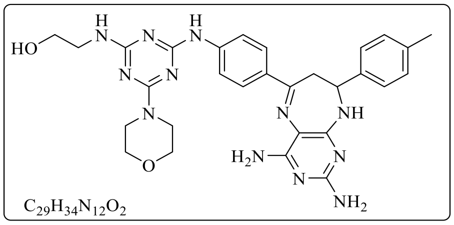

2-((4-((4-(2,4-diamino-8-(p-tolyl)-8,9-dihydro-7H-pyrimido[4,5-b][1,4]diazepin-6-yl)phenyl)amino)-6-morpholino-1,3,5-triazin-2-yl)amino)ethan-1-ol ***(32b)***. Yellow solid. 73% yield; mp 199-201 °C. FT-IR (ATR): ν (cm^{-1}) 3477 (N-H), 3336 (O-H), 1548 and 1488 (C=N and C=C). ^{1}H NMR (400 MHz, DMSO-d_6) δ ppm 2.18 (s, 3H, CH_3), 2.78 (d, *J* = 14.1 Hz, 1H, H-7), 3.45-3.57 (m, 4H, CH_2), 3.58-3.73 (m, 9H, CH_2, H-7), 4.66 (bs, 1H, OH), 4.91-4.99 (m, 1H, H-8), 5.72 (bs, 2H, NH_2), 6.23 (bs, 2H, NH_2), 6.81 (bs, 1H, NH), 6.89 (bs, 1H, NH), 7.01 (d, *J* = 8.0 Hz, 2H, Ar-H), 7.08 (d, *J* = 8.0 Hz, 2H, Ar-H), 7.52-7.60 (m, 2H, Ar-H), 7.63 (d, *J* = 8.7 Hz, 2H, Ar-H), 9.06 (bs, 1H, NH). ^{13}C NMR (100 MHz, DMSO-d_6) δ ppm 20.6 (CH_3), 38.7 (CH_2), 42.9 (CH_2), 43.3 (CH_2), 57.1 (CH), 59.9 (CH_2), 66.0 (CH_2), 101.3 (C), 118.5 (CH), 125.8 (CH), 126.5 (CH), 128.6 (CH), 130.9 (C), 133.9 (C), 135.6 (C), 140.8 (C), 141.2 (C), 154.4 (C), 159.6 (C), 163.2 (C), 163.9 (C), 164.7 (C), 165.6 (C). MS (70 eV) *m/z* (%): 582(55), 567 (19), 464 (9), 344 (28), 329 (41). Anal. Calcd. for $C_{29}H_{34}N_{12}O_2$: C, 59.78; H, 5.88; N, 28.85; Found: C, 59.80; H, 5.83; N, 28.90.

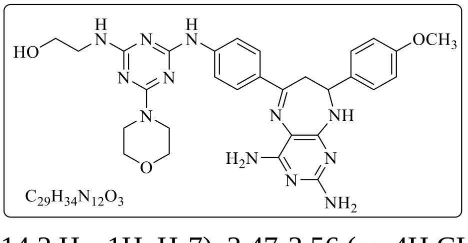

2-((4-((4-(2,4-diamino-8-(4-methoxyphenyl)-8,9-dihydro-7H-pyrimido[4,5-b][1,4]diazepin-6-yl)phenyl)amino)-6-morpholino-1,3,5-triazin-2-yl)amino)ethanol-1-ol ***(32c)***. Yellow solid. 64% yield; mp 217-220 °C. FT-IR (ATR): ν (cm^{-1}) 3482 (N-H), 3368 (O-H), 1560 and 1487 (C=N and C=C). ^{1}H NMR (400 MHz, DMSO-d_6) δ ppm 2.79 (d, *J* = 14.2 Hz, 1H, H-7), 3.47-3.56 (m, 4H,CH_2), 3.57-3.74 (m, 12H, CH_2, OCH_3, H-7), 4.67 (bs, 1H, OH), 4.90-4.97 (m, 1H, H-8), 5.73 (bs, 2H, NH_2), 6.24 (bs, 2H, NH_2), 6.76 (d, *J* = 8.4 Hz, 2H, Ar-H), 6.81 (bs, 1H, NH), 6.88 (bs, 1H, NH), 7.11 (d, *J* = 8.4 Hz, 2H, Ar-H), 7.49-7.61 (m, 2H, Ar-H), 7.63 (d, *J* = 8.6 Hz, 2H, Ar-H), 9.07 (bs, 1H, NH). ^{13}C NMR (100 MHz, DMSO-d_6) δ ppm 42.9 (CH_2), 43.3 (CH_2), 54.9 (CH_3), 57.0 (CH), 59.9 (CH_2), 66.0 (CH_2), 69.8 (CH_2), 101.3 (C), 113.4 (CH), 118.5

(CH), 126.5 (CH), 127.1 (CH), 133.9 (C), 136.3 (C), 140.8 (C), 154.2 (C), 154.6 (C), 157.9 (C), 159.6 (C), 163.2 (C), 163.8 (C), 164.7 (C), 165.6 (C). MS (70 eV) *m/z* (%): 598(5), 464 (6), 329 (7), 135 (12), 91 (21). Anal. Calcd. for $C_{29}H_{34}N_{12}O_3$: C, 58.18; H, 5.72; N, 28.08; Found: C, 58.21; H, 5.71; N, 28.11.

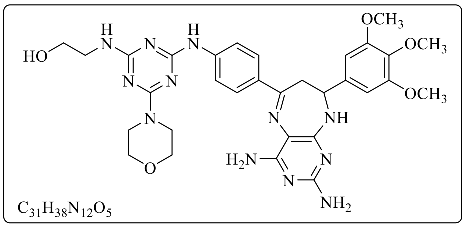

*2-((4-((4-(2,4-diamino-8-(3,4,5-trimethoxyphenyl)-8,9-dihydro-7H-pyrimido[4,5-b][1,4]diazepin-6-yl)phenyl)amino)-6-morpholino-1,3,5-triazin-2-yl)amino)ethanol-1-ol (**32d**).* Yellow solid. 62% yield; mp 200-207 °C. FT-IR (ATR): ν (cm^{-1}) 3490 (N-H), 3355 (O-H), 1591 and 1497 (C=N and C=C). ^{1}H NMR (400 MHz, DMSO-d_6) δ ppm 2.80 (d, *J* = 14.1 Hz, 1H, H-7), 3.20-3.84 (m, 22H, CH_2,OCH_3, H-7), 4.66 (bs, 1H, OH), 4.82-4.99 (m, 1H, H-8), 5.66 (bs, 2H, NH_2), 6.17 (bs, 2H, NH_2), 6.52 (s, 2H, Ar-H), 6.78 (bs, 2H, NH), 7.54-7.74 (m, 4H, Ar-H), 7.11 (d, *J* = 8.4 Hz, 2H, Ar-H), 7.49-7.61 (m, 2H, Ar-H), 7.63 (d, *J* = 8.6 Hz, 2H, Ar-H), 9.08 (bs, 1H, NH). ^{13}C NMR (100 MHz, DMSO-d_6) δ ppm 39.3 (CH_2), 43.4 (CH_2), 43.8 (CH_2), 56.3 (CH_3), 58.5 (CH), 60.3 (CH_3), 60.4 (CH_2), 66.5 (CH_2), 102.1 (C), 104.3 (CH), 118.9 (CH), 127.0 (CH), 134.4 (C), 134.4 (C), 136.8 (C), 140.2 (C), 141.3 (C), 153.0 (C), 154.7 (C), 155.4 (C), 160.0 (C), 160.5 (C), 163.8 (C), 166.4 (C). MS (70 eV) *m/z* (%): 658 (9), 465 (10), 344 (18), 329 (28), 314 (8). Anal. Calcd. for $C_{31}H_{38}N_{12}O_5$: C, 56.52; H, 5.81; N, 25.52; Found: C, 56.49; H, 5.83; N, 25.50.

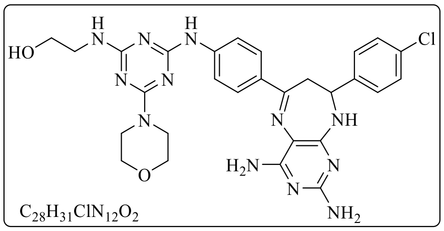

*2-((4-((4-(2,4-diamino-8-(4-chlorophenyl)-8,9-dihydro-7H-pyrimido[4,5-b][1,4]diazepin-6-yl)phenyl)amino)-6-morpholino-1,3,5-triazin-2-yl)amino)ethan-1-ol (**32e**).* Yellow solid. 78% yield; mp 220-223 °C. FT-IR (ATR): ν (cm^{-1}) 3485 (N-H), 3336 (O-H), 1568 and 1485 (C=N and C=C). ^{1}H NMR (400 MHz, DMSO-d_6) δ ppm 2.75 (d, *J* = 14.2 Hz, 1H, H-7), 3.44-3.57 (m, 4H,CH_2), 3.56-3.72 (m, 8H, CH_2), 3.77 (dd, *J* = 14.2, 6.0 Hz, 1H, H-7), 4.66 (bs, 1H, OH), 5.00-5.08 (m, 1H, H-8), 5.71 (bs, 2H, NH_2), 6.23 (bs, 2H, NH_2), 6.81 (bs, 1H, NH), 7.00 (bs, 1H, NH), 7.19 (d, *J* = 8.4 Hz, 2H, Ar-H), 7.25 (d, *J* = 8.4 Hz, 2H, Ar-H), 7.47-7.58 (m, 2H, Ar-H), 7.62 (d, *J* = 8.4 Hz, 2H, Ar-H), 9.06 (bs, 1H, NH). ^{13}C NMR (100 MHz, DMSO-d_6) δ ppm 38.2 (CH_2), 42.9 (CH_2), 43.3 (CH_2), 57.0 (CH), 60.0 (CH_2), 66.0 (CH_2), 101.4 (C), 118.5 (CH), 126.4 (CH), 127.8 (CH), 127.9 (CH), 131.1 (C), 133.7 (C), 140.8 (C), 143.1 (C), 154.3 (C), 154.4 (C), 159.9 (C), 163.3 (C), 164.0 (C), 164.6 (C), 165.6 (C). MS (70 eV) *m/z* (%): 602: 604 [M^+]:[M + 2]$^+$ (18/6), 558 (19), 464 (11), 138 (70), 103 (63), 43 (100). Anal. Calcd. for $C_{28}H_{31}ClN_{12}O_2$: C, 55.76; H, 5.18; N, 27.87; Found: C, 55.71; H, 5.15; N, 27.83.

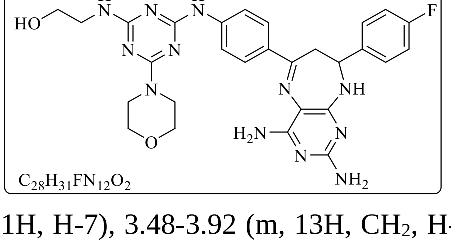

*2-((4-((4-(2,4-diamino-8-(4-fluorophenyl)-8,9-dihydro-7H-pyrimido[4,5-b][1,4]diazepin-6-yl)phenyl)amino)-6-morpholino-1,3,5-triazin-2-yl)amino)ethan-1-ol (**32f**).* Yellow solid. 73% yield; mp 227-229 °C. FT-IR (ATR): ν (cm^{-1}) 3492 (N-H), 3350 (O-H), 1590 and 1492 (C=N and C=C). ^{1}H NMR (400 MHz, DMSO-d_6) δ ppm 2.67-2.82 (m, 1H, H-7), 3.48-3.92 (m, 13H, CH_2, H-7), 4.67 (bs, 1H, OH), 4.93-5.17 (m, 1H, H-8), 5.75 (bs, 2H, NH_2), 6.24 (bs, 2H, NH_2), 6.81 (bs, 1H, NH), 6.91-7.11 (m, 3H, Ar-H, NH), 7.11-7.33 (m, 2H, Ar-

H), 7.39-7.79 (m, 4H, Ar-H), 9.06 (bs, 1H, NH). ^{13}C NMR (100 MHz, DMSO-d_6) δ ppm 38.5 (CH_2), 42.9 (CH_2), 43.3 (CH_2), 57.2 (CH), 59.9 (CH_2), 66.0 (CH_2), 101.4 (C), 114.6 (d, $^2J_{CF}$ = 20.2 Hz, CH), 118.5 (CH), 126.3 (d, $^4J_{CF}$ = 2.2 Hz, C), 126.4 (CH), 127.87 (d, $^3J_{CF}$ = 8.6 Hz, CH), 133.7 (C), 140.4 (C), 140.8 (C), 154.3 (C), 154.6 (C), 159.7 (C), 163.2 (C), 163.53 (d, $^1J_{CF}$ = 244.3 Hz, C), 165.7 (C), 165.9 (C). MS (70 eV) *m/z* (%): 586(29), 572 (11), 542 (2), 464 (7), 271 (7), 245 (7), 122 (32). Anal. Calcd. for $C_{28}H_{31}FN_{12}O_2$: C, 57.33; H, 5.33; N, 28.65; Found: C, 57.29; H, 5.35; N, 28.68.

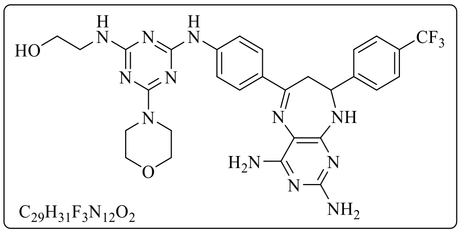

*2-((4-((4-(2,4-diamino-8-(4-(trifluoromethyl)phenyl)-8,9-dihydro-7H-pyrimido[4,5-b][1,4]diazepin-6-yl)phenyl)amino)-6-morpholino-1,3,5-triazin-2-yl)amino)ethanol-1-ol (**32g**)*. Yellow solid. 70% yield; mp 225-228 °C. FT-IR (ATR): ν (cm^{-1}) 3442 (N-H), 3323 (O-H), 1541 and 1486 (C=N and C=C). ^1H NMR (400 MHz, DMSO-d_6) δ ppm 2.69-2.93 (m, 1H, H-7), 3.44-3.57 (m, 4H, CH_2), 3.57-3.77 (m, 8H, CH_2), 3.77-3.89 (m, 1H, H-7), 4.66 (bs, 1H, OH), 5.02-5.23 (m, 1H, H-8), 5.71 (bs, 2H, NH_2), 6.21 (bs, 2H, NH_2), 6.80 (bs, 1H, NH), 7.07 (bs, 1H, NH), 7.25-7.82 (m, 8H, Ar-H), 9.06 (bs, 1H, NH). ^{13}C NMR (100 MHz, DMSO-d_6) δ ppm 38.2 (CH_2), 42.9 (CH_2), 43.3 (CH_2), 57.3 (CH), 60.0 (CH_2), 66.0 (CH_2), 101.6 (C), 111.6 (q, $^1J_{CF}$ = 268.43 Hz, CF_3), 118.5 (CH), 122.9 (C), 124.9 (d, $^3J_{CF}$ = 6.27 Hz, CH), 126.4 (d, $^4J_{CF}$ = 3.18 Hz, CH), 126.8 (CH), 126.3 (d, $^2J_{CF}$ = 147.95 Hz, C), 133.7 (C), 140.9 (C), 148.7 (C), 154.2 (C), 154.5 (C), 160.0 (C), 163.4 (C), 164.0 (C), 164.7 (C). MS (70 eV) *m/z* (%): 636(81), 621 (18), 477 (9), 464 (10), 341 (13), 295 (15), 266 (14). Anal. Calcd. for $C_{29}H_{31}F_3N_{12}O_2$: C, 54.71; H, 4.91; N, 26.40; Found: C, 54.69; H, 4.93; N, 26.46.

Characterization of triazinylamino-diazepines (33a-g)

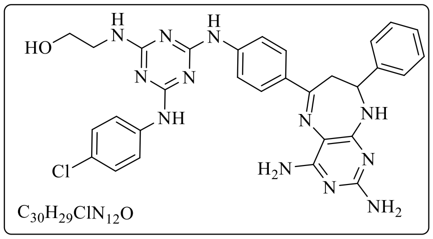

*2-((4-((4-chlorophenyl)amino)-6-((4-(2,4-diamino-8-phenyl-8,9-dihydro-7H-pyrimido[4,5-b][1,4]diazepin-6-yl)phenyl)amino)-1,3,5-triazin-2-yl)amino)ethanol-1-ol (**33a**)*. Yellow solid. 62% yield; mp 194-196 °C. FT-IR (ATR): ν (cm^{-1}) 3486 (N-H), 3371 (O-H), 1556 and 1489 (C=N and C=C). ^1H NMR (400 MHz, DMSO-d_6) δ ppm 2.79 (d, *J* = 14.0 Hz, 1H, H-7), 3.35-3.43 (m, 2H, CH_2), 3.51-3.59 (m, 2H, CH_2), 3.73 (dd, *J* = 14.0, 5.9 Hz, 1H, H-7), 4.70 (bs, 1H, OH), 5.00-5.08 (m, 1H, H-8), 5.64 (bs, 2H, NH_2), 6.15 (bs, 2H, NH_2), 6.90 (bs, 1H, NH), 7.00 (bs, 1H, NH), 7.10 (s, 1H, Ar-H), 7.15-7.24 (m, 4H, Ar-H), 7.28 (d, *J* = 7.3 Hz, 2H, Ar-H), 7.48-7.58 (m, 2H, Ar-H), 7.66 (d, *J* = 8.2 Hz, 2H, Ar-H), 7.83 (d, *J* = 7.3 Hz, 2H, Ar-H), 9.12 (bs, 1H, NH), 9.23 (bs, 1H, NH). ^{13}C NMR (100 MHz, DMSO-d_6) δ ppm 38.7 (CH_2), 42.9 (CH_2), 57.7 (CH), 59.9 (CH_2), 101.5 (C), 118.9 (CH), 121.2 (CH), 125.9 (CH), 126.3 (CH), 126.6 (CH), 128.0 (CH), 128.1 (CH), 134.3 (C), 139.4 (C), 140.4 (C), 144.3 (C), 154.3 (C), 154.6 (C), 160.2 (C), 163.5 (C), 163.8 (C), 164.0 (C), 164.2 (C), 165.7 (C). MS (70 eV) *m/z* (%): 608:610 [M^+]:[M + 2]$^+$ (6/3), 466 (7), 345 (11), 140 (22), 104 (100). Anal. Calcd. for $C_{30}H_{29}ClN_{12}O$: C, 59.16; H, 4.80; N, 27.60; Found: C, 59.13; H, 4.84; N, 27.65.

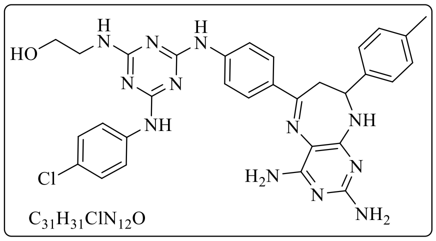

*2-((4-((4-chlorophenyl)amino)-6-((4-(2,4-diamino-8-(p-tolyl)-8,9-dihydro-7H-pyrimido[4,5-b][1,4]diazepin-6-yl)phenyl)amino)-1,3,5-triazin-2-yl)amino)ethanol-1-ol (**33b**)*. Yellow solid. 64% yield; mp 193-195 °C. FT-IR (ATR): ν (cm^{-1}) 3495 (N-H), 3377 (O-H), 1562 and 1484 (C=N and C=C). ^{1}H NMR (400 MHz, DMSO-d_6) δ ppm 2.17 (s, 3H, CH_3), 2.79 (d, *J* = 14.5 Hz, 1H, H-7), 3.37-3.44 (m, 2H, CH_2), 3.51-3.59 (m, 2H, CH_2), 3.71 (dd, *J* = 14.5, 6.6 Hz, 1H, H-7), 4.71 (bs, 1H, OH), 4.93-5.01 (m, 1H, H-8), 5.65 (bs, 2H, NH_2), 6.17 (bs, 2H, NH_2), 6.86 (bs, 1H, NH), 7.01 (d, *J* = 7.9 Hz, 3H, Ar-H, NH), 7.08 (d, *J* = 7.9 Hz, 2H, Ar-H), 7.29 (d, *J* = 6.9 Hz, 2H, Ar-H), 7.54-7.62 (m, 2H, Ar-H), 7.68 (d, *J* = 8.4 Hz, 2H, Ar-H), 7.84 (d, *J* = 6.9 Hz, 2H, Ar-H), 9.14 (bs, 1H, NH), 9.24 (bs, 1H, NH). ^{13}C NMR (100 MHz, DMSO-d_6) δ ppm 20.6 (CH_3), 38.7 (CH_2), 42.9 (CH_2), 57.1 (CH), 59.9 (CH_2), 101.5 (C), 119.0 (CH), 121.2 (CH), 125.1 (C), 125.8 (CH), 126.4 (CH), 128.1 (CH), 128.6 (CH), 134.4 (C), 135.5 (C), 139.4 (C), 140.4 (C), 141.3 (C), 154.1 (C), 154.6 (C), 160.2 (C), 163.5 (C), 163.8 (C), 163.9 (C), 165.7 (C). MS (70 eV) *m/z* (%): 622:624 [M$^+$]:[M + 2]$^+$ (1.25/0.43), 588 (1), 505 (1), 486 (3), 359 (5), 241 (8), 118 (100). Anal. Calcd. for $C_{31}H_{31}ClN_{12}O$: C, 59.75; H, 5.01; N, 26.97; Found: C, 59.70; H, 5.05; N, 27.00.

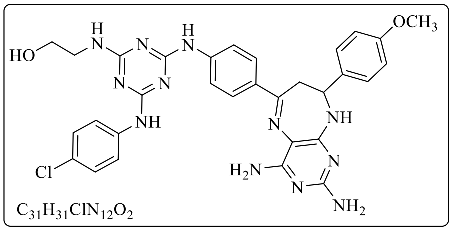

*2-((4-((4-chlorophenyl)amino)-6-((4-(2,4-diamino-8-(4-methoxyphenyl)-8,9-dihydro-7H-pyrimido[4,5-b][1,4]diazepin-6-yl)phenyl)amino)-1,3,5-triazin-2-yl)amino)ethanol-1-ol (**33c**)*. Yellow solid. 62% yield; mp 200-203 °C. FT-IR (ATR): ν (cm^{-1}) 3468 (N-H), 3377 (O-H), 1568 and 1507 (C=N and C=C). ^{1}H NMR (400 MHz, DMSO-d_6) δ ppm 2.79 (d, *J* = 14.2 Hz, 1H, H-7), 3.37-3.43 (m, 2H, CH_2), 3.51-3.60 (m, 2H, CH_2), 3.60-3.74 (m, 4H, OCH_3, H-7), 4.71 (bs, 1H, OH), 4.91-5.00 (m, 1H, H-8), 5.64 (bs, 2H, NH_2), 6.16 (bs, 2H, NH_2), 6.77 (d, *J* = 8.2 Hz, 2H, Ar-H), 6.83 (bs, 1H, NH), 7.02 (bs, 1H, NH), 7.11 (d, *J* = 8.2 Hz, 2H, Ar-H), 7.29 (d, *J* = 7.5 Hz, 2H, Ar-H), 7.52-7.62 (m, 2H, Ar-H), 7.69 (d, *J* = 7.8 Hz, 2H, Ar-H), 7.84 (d, *J* = 7.5 Hz, 2H, Ar-H), 9.12 (bs, 1H, NH), 9.24 (bs, 1H, NH). ^{13}C NMR (100 MHz, DMSO-d_6) δ ppm 38.9 (CH_2), 42.9 (CH_2), 54.9 (CH), 56.9 (CH_3), 59.9 (CH_2), 101.5 (C), 113.4 (CH), 119.0 (CH), 121.2 (CH), 125.1 (C), 126.4 (CH), 127.0 (CH), 128.1 (CH), 134.4 (C), 136.4 (C), 139.3 (C), 140.4 (C), 154.2 (C), 154.5 (C), 157.9 (C), 160.2 (C), 163.5 (C), 163.8 (C), 163.9 (C), 165.7 (C). MS (70 eV) *m/z* (%): 638:640 [M$^+$]:[M + 2]$^+$ (3/1), 505 (11), 460 (3), 375 (8), 241 (10), 134 (100). Anal. Calcd. for $C_{31}H_{31}ClN_{12}O_2$: C, 58.26; H, 4.89; N, 26.30; Found: C, 58.22; H, 4.90; N, 26.28.

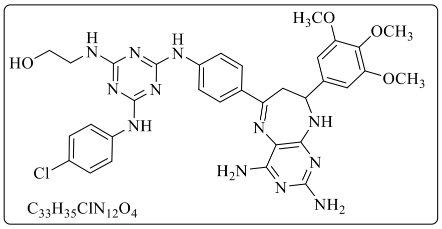

*2-((4-((4-chlorophenyl)amino)-6-((4-(2,4-diamino-8-(3,4,5-trimethoxyphenyl)-8,9-dihydro-7H-pyrimido[4,5-b][1,4]diazepin-6-yl)phenyl)amino)-1,3,5-triazin-2-yl)amino)ethan-1-ol (**33d**)*. Yellow solid. 56% yield; mp 197-199 °C. FT-IR (ATR): ν (cm^{-1}) 3479 (N-H), 3366 (O-H), 1568 and 1487 (C=N and C=C). ^{1}H NMR (400 MHz, DMSO-d_6) δ ppm 2.82 (d, *J* = 13.9 Hz, 1H, H-7), 3.37-3.42 (m, 2H, CH_2), 3.54 (s, 3H, OCH_3), 3.53-3.58 (m, 2H, CH_2), 3.61 (s, 6H, OCH_3), 3.67-4.74 (m, 1H, H-7), 4.71 (bs, 1H, OH), 4.89-4.96 (m, 1H, H-8),

5.66 (bs, 2H, NH_2), 6.17 (bs, 2H, NH_2), 6.53 (s, 2H, Ar-H), 6.81 (bs, 1H, NH), 7.02 (bs, 1H, NH), 7.25-7.32 (m, 2H, Ar-H), 7.63 (t, J = 7.8 Hz, 2H, Ar-H), 7.72 (d, J = 8.5 Hz, 2H), 7.84 (d, J = 7.8 Hz, 2H), 9.14 (bs, 1H, NH), 9.25 (bs, 1H, NH). ^{13}C NMR (100 MHz, DMSO-d_6) δ ppm 38.6 (CH_2), 43.0 (CH_2), 55.8 (CH_3), 57.8 (CH), 59.9 (CH_2), 59.9 (CH_3), 101.7 (C), 103.8 (CH), 118.8 (CH), 121.2 (CH), 125.0 (C), 126.4 (CH), 128.1 (CH), 134.3 (C), 136.3 (C), 139.4 (C), 139.7 (C), 140.5 (C), 152.5 (C), 154.3 (C), 154.8 (C), 160.1 (C), 163.4 (C), 163.8 (C), 163.9 (C), 165.7 (C).MS (70 eV) *m/z* (%): [M^+ - 34] 664 (4), 603 (10), 577 (20), 313 (22), 194 (36). Anal. Calcd. for $C_{33}H_{35}ClN_{12}O_4$: C, 56.69; H, 5.05; N, 24.04; Found: C, 56.65; H, 5.11; N, 24.01.

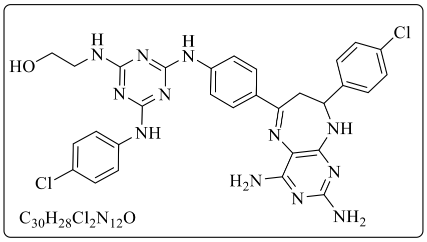

*2-((4-((4-chlorophenyl)amino)-6-((4-(2,4-diamino-8-(4-chlorophenyl)-8,9-dihydro-7H-pyrimido[4,5-b][1,4]diazepin-6-yl)phenyl)amino)-1,3,5-triazin-2-yl)amino)ethanol-1-ol (**33e**)*. Yellow solid. 88% yield; mp 203-205 °C. FT-IR (ATR): ν (cm^{-1}) 3486 (N-H), 3365 (O-H), 1574 and 1487 (C=N and C=C). ^{1}H NMR (400 MHz, DMSO-d_6) δ ppm 2.75 (d, J = 14.2 Hz, 1H, H-7), 3.36-3.43 (m, 2H, CH_2), 3.50-3.64 (m, 2H, CH_2), 3.79 (dd, J = 14.2, 5.4 Hz, 1H, H-7), 4.70 (bs, 1H, OH), 5.02-5.09 (m, 1H, H-8), 5.65 (bs, 2H, NH_2), 6.17 (bs, 2H, NH_2), 6.94-7.07 (m, 2H, NH), 7.20 (d, J = 8.2 Hz, 2H, Ar-H), 7.23-7.33 (m, 4H, Ar-H), 7.50-7.60 (m, 2H, Ar-H), 7.68 (d, J = 8.2 Hz, 2H, Ar-H), 7.84 (d, J = 7.2 Hz, 2H, Ar-H), 9.14 (bs, 1H, NH), 9.24 (bs, 1H, NH). ^{13}C NMR (100 MHz, DMSO-d_6) δ ppm 38.3 (CH_2), 42.9 (CH_2), 57.0 (CH), 59.9 (CH_2), 101.6 (C), 118.9 (CH), 121.2 (CH), 125.1 (C), 126.3 (CH), 127.8 (CH), 127.9 (CH), 128.1 (CH), 131.0 (C), 134.2 (C), 139.4 (C), 140.5 (C), 143.2 (C), 154.0 (C), 154.6 (C), 160.3 (C), 163.6 (C), 163.8 (C), 163.9 (C), 165.7 (C). MS (70 eV) *m/z* (%): 642:644:646 [M^+]:[M + 2]$^+$:[M + 4]$^+$ (6/3/1), 627 (1), 379 (8), 262 (10), 241 (8), 138 (100). Anal. Calcd. for $C_{30}H_{28}Cl_2N_{12}O$: C, 55.99; H, 4.39; N, 26.12; Found: C, 56.03; H, 4.41; N, 26.13.

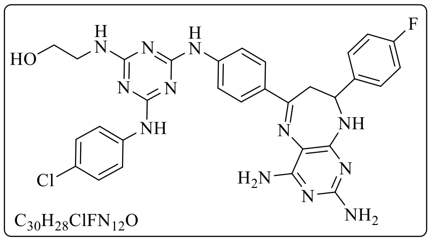

*2-((4-((4-chlorophenyl)amino)-6-((4-(2,4-diamino-8-(4-fluorophenyl)-8,9-dihydro-7H-pyrimido[4,5-b][1,4]diazepin-6-yl)phenyl)amino)-1,3,5-triazin-2-yl)amino)ethanol-1-ol (**33f**)*. Yellow solid. 70% yield; mp 240-243 °C. FT-IR (ATR): ν (cm^{-1}) 3480 (N-H), 3385 (O-H), 1558 and 1488 (C=N and C=C). ^{1}H NMR (400 MHz, DMSO-d_6) δ ppm 2.76 (d, J = 14.1 Hz, 1H, H-7), 3.36-3.46 (m, 2H, CH_2), 3.51-3.60 (m, 2H, CH_2), 3.75 (dd, J = 14.1, 6.1 Hz, 1H, H-7), 4.71 (bs, 1H, OH), 5.01-5.10 (m, 1H, H-8), 5.67 (bs, 2H, NH_2), 6.19 (bs, 2H, NH_2), 6.92-7.06 (m, 4H, NH, Ar-H), 7.21 (dd, J = 8.0, 5.8 Hz, 2H, Ar-H), 7.29 (d, J = 7.0 Hz, 2H, Ar-H), 7.50-7.59 (m, 2H, Ar-H), 7.68 (d, J = 8.2 Hz, 2H, Ar-H), 7.84 (d, J = 7.0 Hz, 2H, Ar-H), 9.13 (bs, 1H, NH), 9.24 (bs, 1H, NH).NMR ^{13}C (100 MHz, DMSO-d_6) δ ppm 39.1 (CH_2), 43.4 (CH_2), 57.6 (CH), 60.4 (CH_2), 102.0 (C), 115.1 (d, $^{2}J_{CF}$ = 21.2 Hz, CH), 119.4 (CH), 121.7 (CH), 124.3 (C), 125.6 (d, $^{4}J_{CF}$ = 4.9 Hz, C), 126.8 (CH), 128.3 (d, $^{3}J_{CF}$ = 8.0 Hz, CH), 128.6 (CH), 134.7 (C), 139.8 (C), 141.0 (C), 154.8 (C), 155.0 (C), 160.6 (C), 161.4 (d, $^{1}J_{CF}$ = 241.7 Hz, C), 163.9 (C), 164.2 (C), 164.4 (C), 166.2 (C). MS (70 eV) *m/z* (%): 626:628 [M^+]:[M + 2]$^+$ (2/1), 540 (2), 363 (15), 262 (40), 241 (41), 122 (100). Anal. Calcd. for $C_{30}H_{28}ClFN_{12}O$: C, 57.46; H, 4.50; N, 26.80; Found: C, 57.42; H, 4.47; N, 26.77.

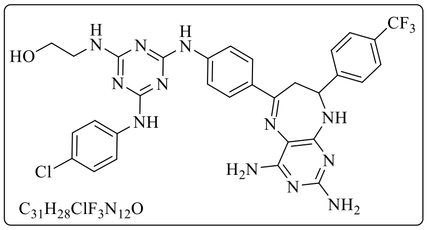

*2-((4-((4-chlorophenyl)amino)-6-((4-(2,4-diamino-8-(4-(trifluoromethyl)phenyl)-8,9-dihydro-7H-pyrimido[4,5-b][1,4]diazepin-6-yl)phenyl)amino)-1,3,5-triazin-2-yl)amino)ethanol-1-ol (**33g**)*. Yellow solid. 68% yield; mp 235-236 °C. FT-IR (ATR): ν (cm^{-1}) 3493 (N-H), 3366 (O-H), 1558 and 1488 (C=N and C=C). ^{1}H NMR (400 MHz, DMSO-*d*$_6$) δ ppm 2.80 (d, *J* = 14.2 Hz, 1H, H-7), 3.37-3.44 (m, 2H, CH_2), 3.52-3.59 (m, 2H, CH_2), 3.86 (dd, *J* = 14.2, 5.8 Hz, 1H, H-7), 4.71 (bs, 1H, OH), 5.12-5.20 (m, 1H, H-8), 5.69 (bs, 2H, NH_2), 6.19 (bs, 2H, NH_2), 7.01 (bs, 1H, NH), 7.06 (bs, 1H, NH), 7.29 (d, *J* = 6.7 Hz, 2H, Ar-H), 7.41 (d, *J* = 8.1 Hz, 2H, Ar-H), 7.49-7.60 (m, 4H, Ar-H), 7.67 (d, *J* = 8.1 Hz, 2H, Ar-H), 7.83 (d, *J* = 6.7 Hz, 2H, Ar-H), 9.13 (bs, 1H, NH), 9.24 (bs, 1H, NH). ^{13}C NMR (100 MHz, DMSO-*d*$_6$) δ ppm 38.2 (CH_2), 42.9 (CH_2), 57.2 (CH), 59.9 (CH_2), 101.6 (C), 118.9 (CH), 121.2 (CH), 124.0 (q, $^1J_{CF}$ = 220.6 Hz, CF_3), 124.9 (d, $^3J_{CF}$ = 4.0 Hz, CH), 126.3 (d, $^4J_{CF}$ = 3.0 Hz, CH), 126.3 (d, $^2J_{CF}$ = 148.7 Hz, C), 126.8 (CH), 127.4 (C), 128.1 (CH), 134.1 (C), 139.3 (C), 140.5 (C), 148.7 (C), 153.9 (C), 154.6 (C), 160.2 (C), 163.6 (C), 163.7 (C), 163.9 (C), 165.7 (C). MS (70 eV) *m/z* (%): 676:678 [M^+]:$[M + 2]^+$ (5/2), 413 (22), 398 (8), 262 (26), 172 (73), 140 (67). Anal. Calcd. for $C_{31}H_{28}ClF_3N_{12}O$: C, 54.99; H, 4.17; N, 24.82; Found: C, 55.02; H, 4.13; N, 24.79.

NMR spectra of synthetized compounds

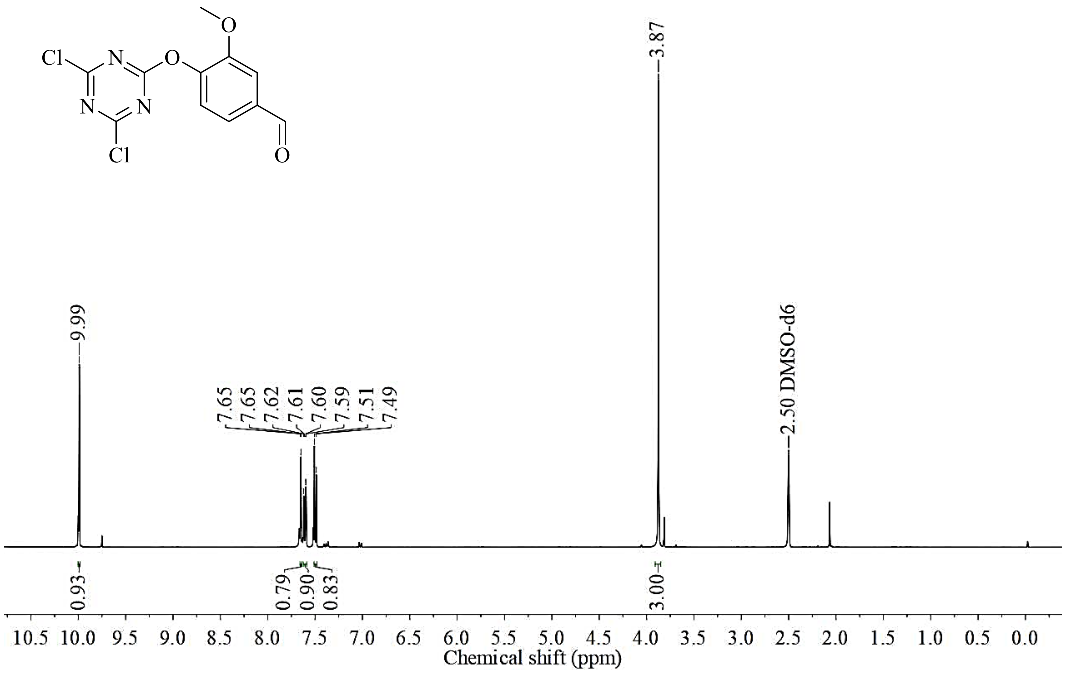

^{1}H NMR (400 MHz, DMSO-d_6) spectra of **7**.

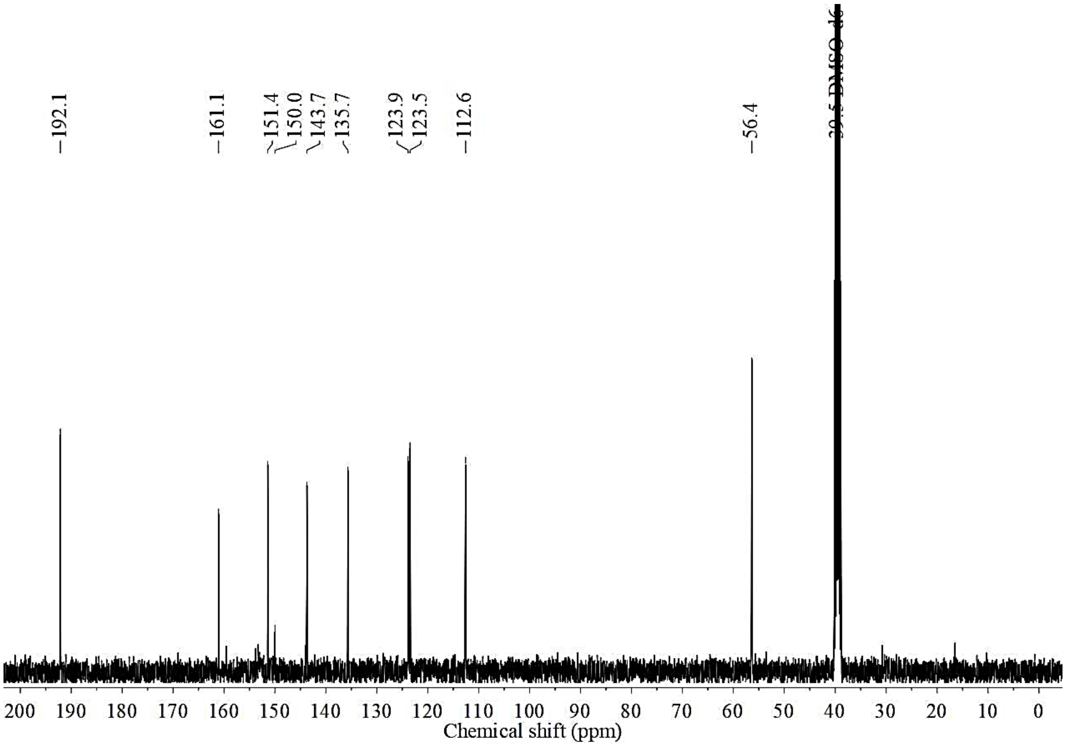

^{13}C NMR (100 MHz, DMSO-d_6) spectra of **7**.

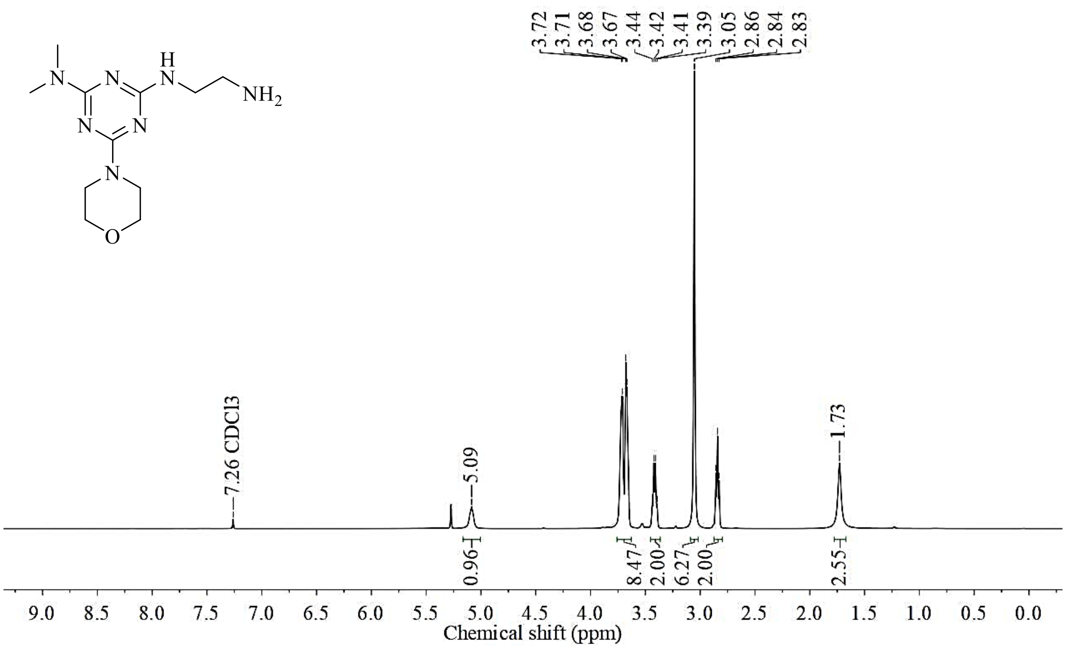

^{1}H NMR (400 MHz, $CDCl_3$) spectra of **12**.

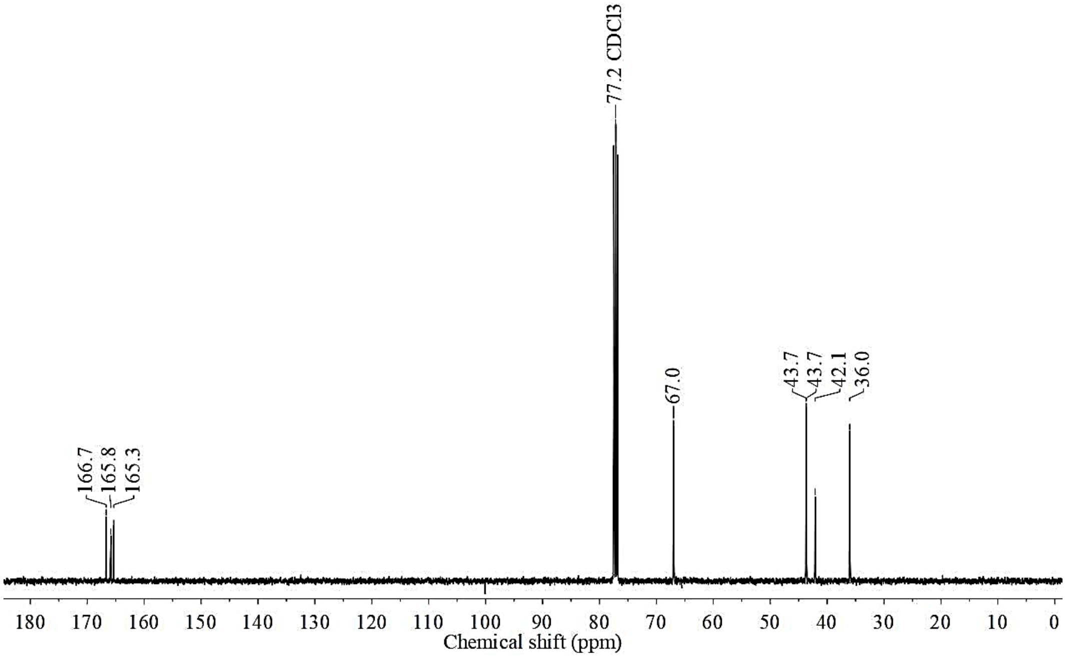

^{13}C NMR (100 MHz, 400 MHz, $CDCl_3$) spectra of **12**.

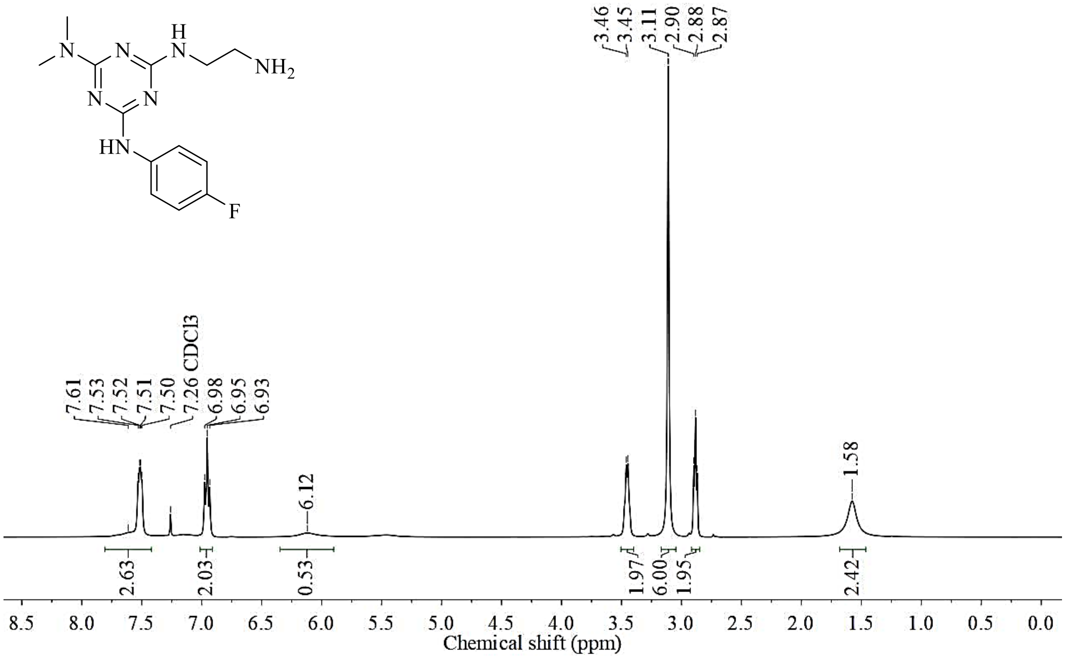

^{1}H NMR (400 MHz, CDCl3) spectra of **13**.

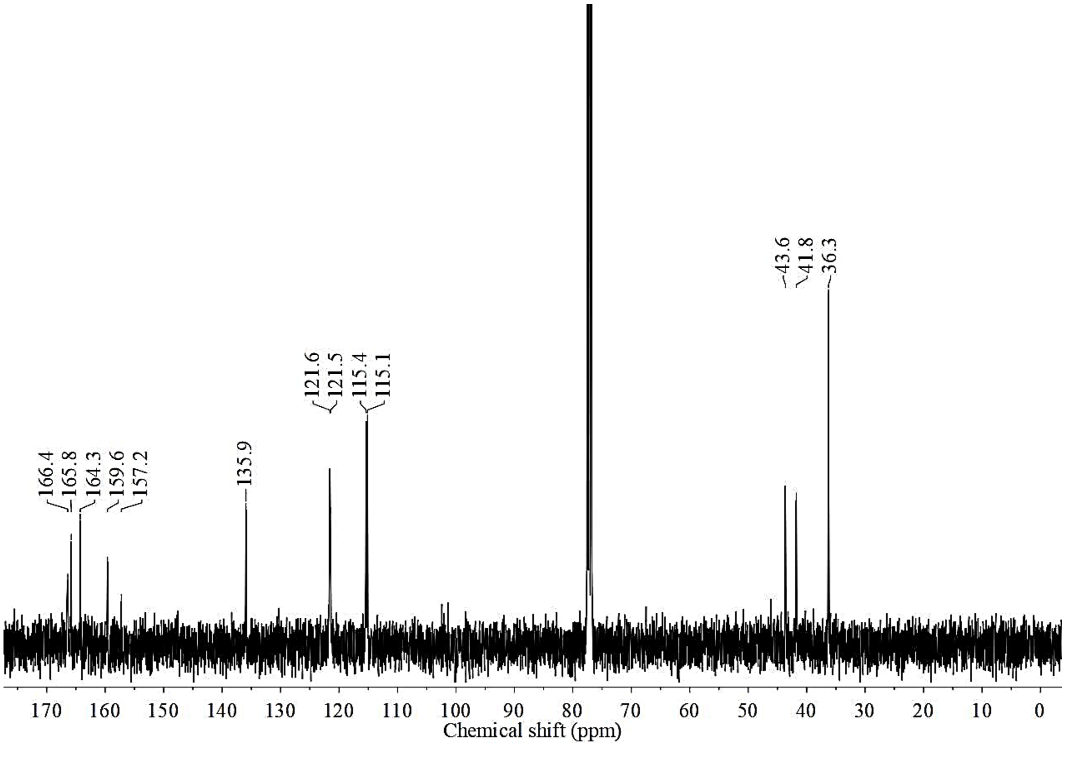

^{13}C NMR (100 MHz, CDCl3) spectra of **13**.

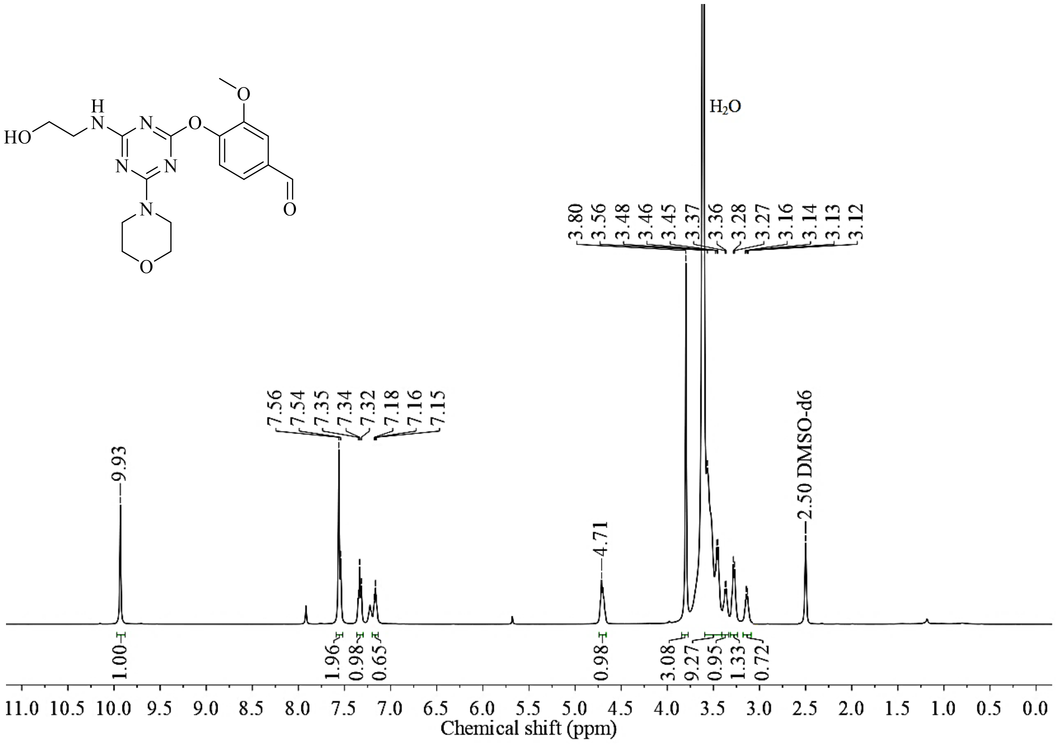

^{1}H NMR (400 MHz, DMSO-d_6) spectra of **14**.

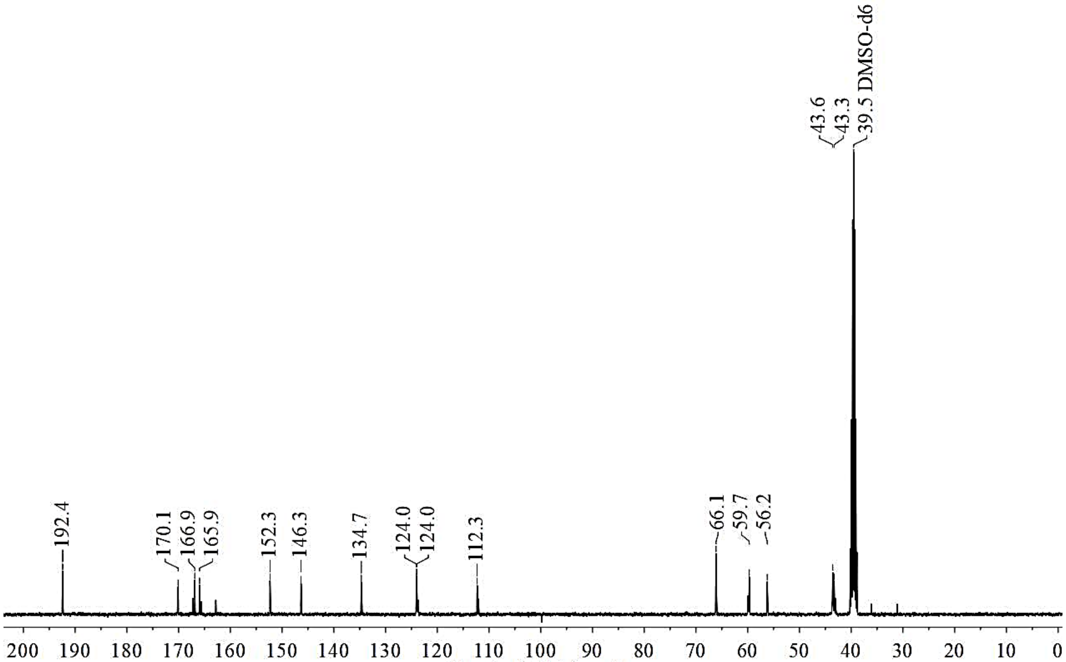

^{13}C NMR (100 MHz, DMSO-d_6) spectra of **14**.

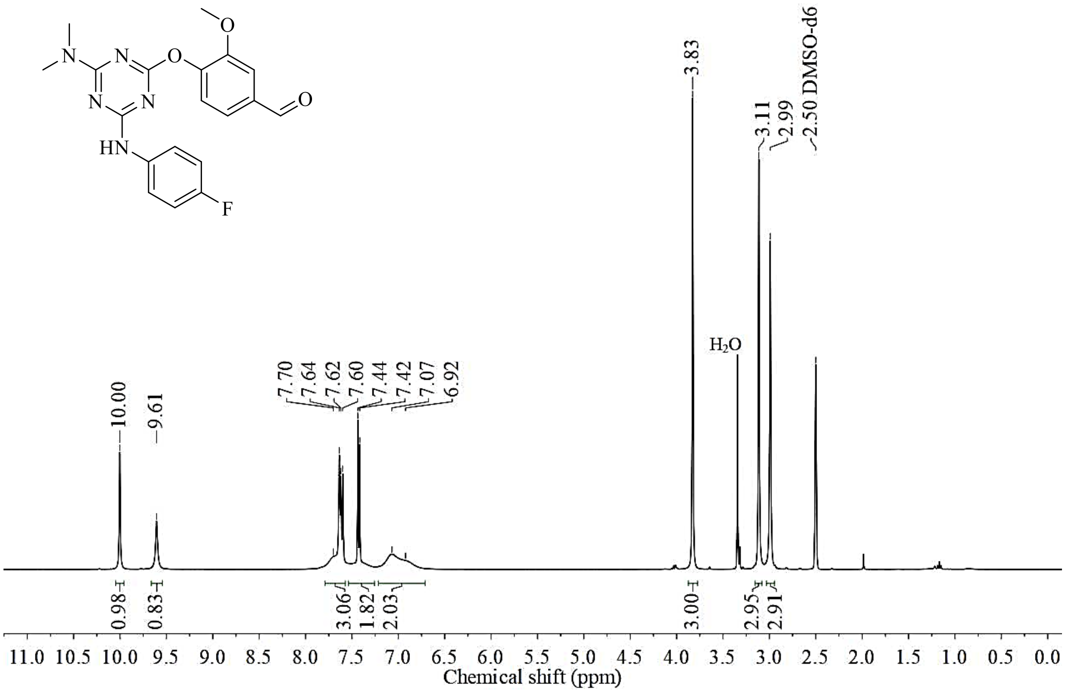

^{1}H NMR (400 MHz, DMSO-d_6) spectra of **15**.

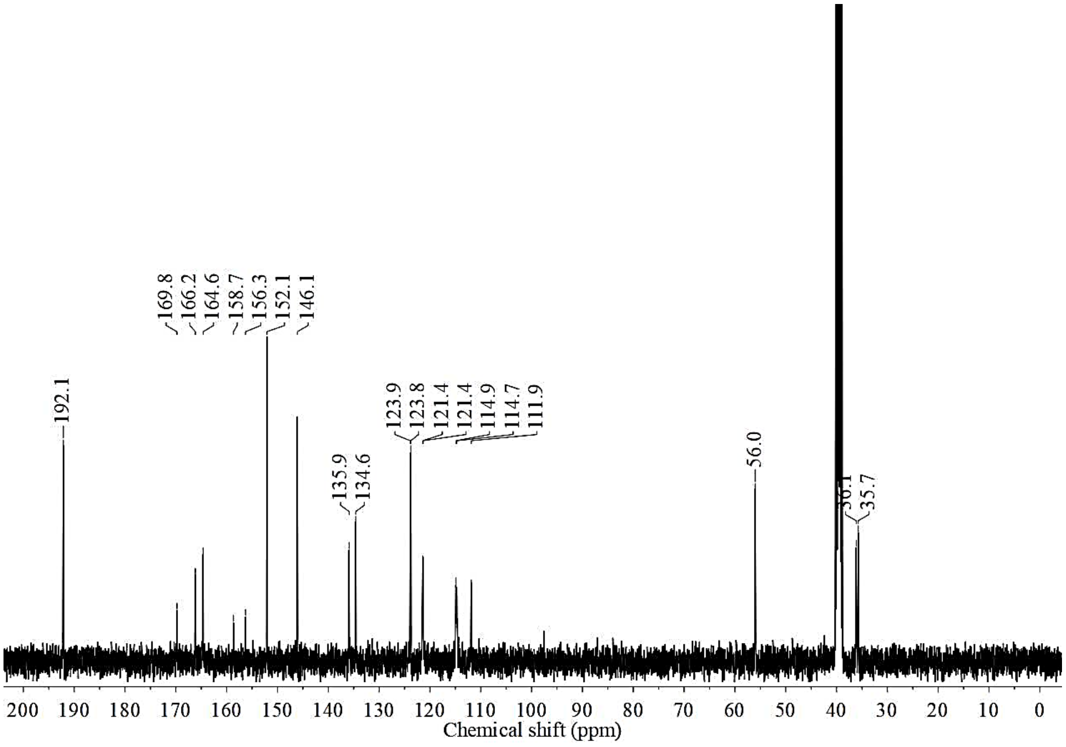

^{13}C NMR (100 MHz, DMSO-d_6) spectra of **15**.

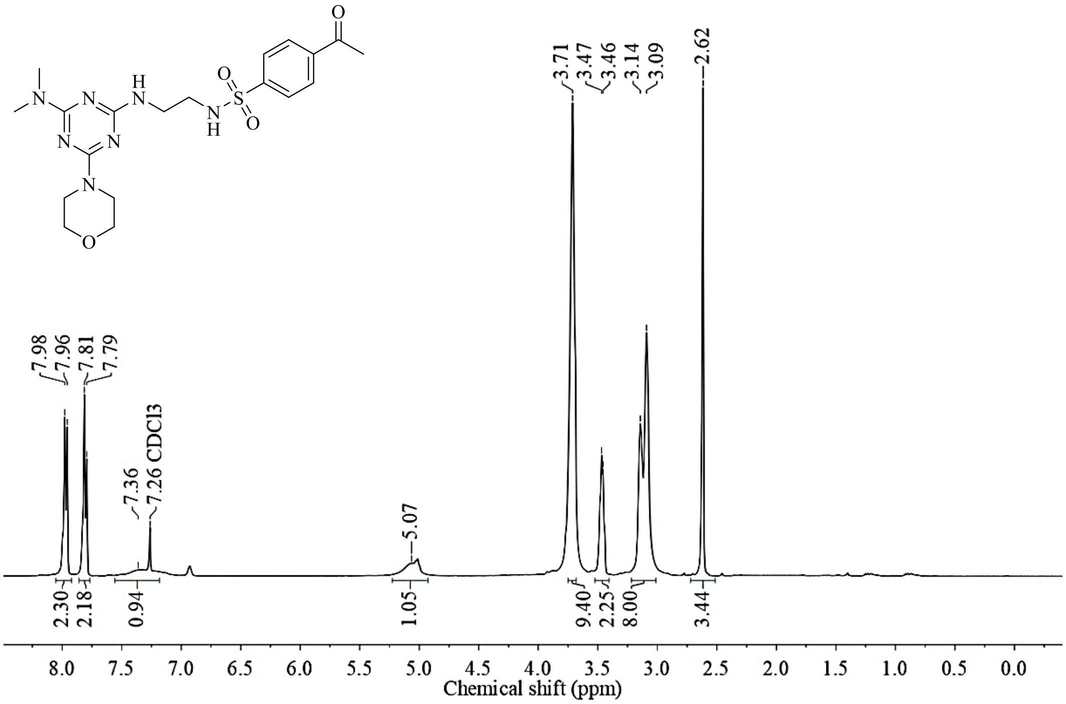

^{1}H NMR (400 MHz, $CDCl_3$) spectra of **17**.

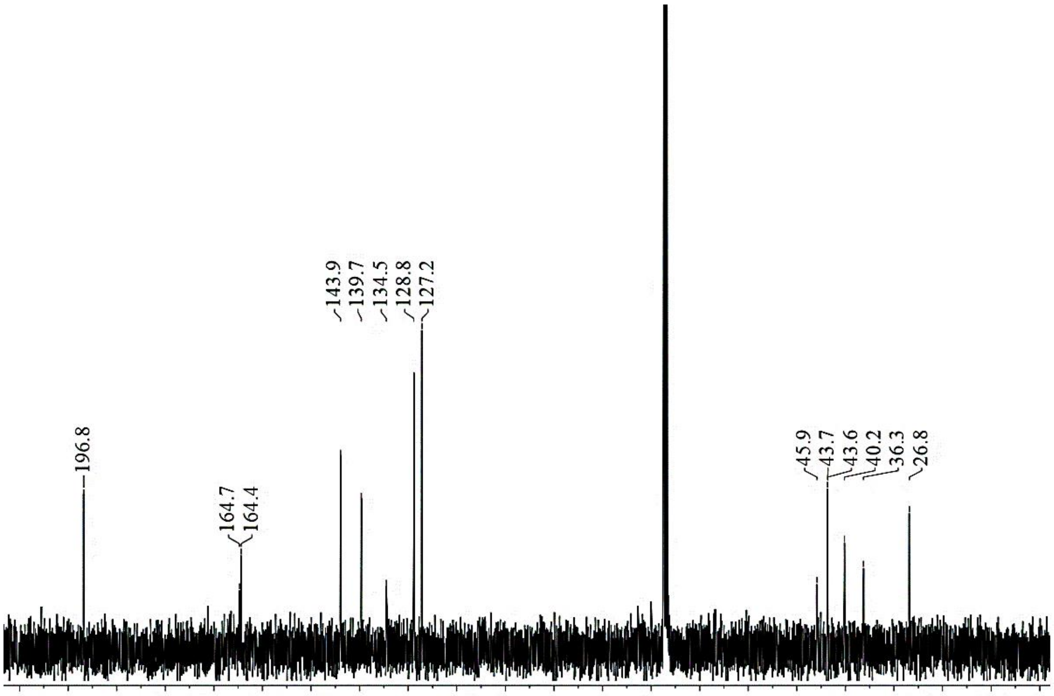

^{13}C NMR (100 MHz, $CDCl_3$) spectra of **17**.

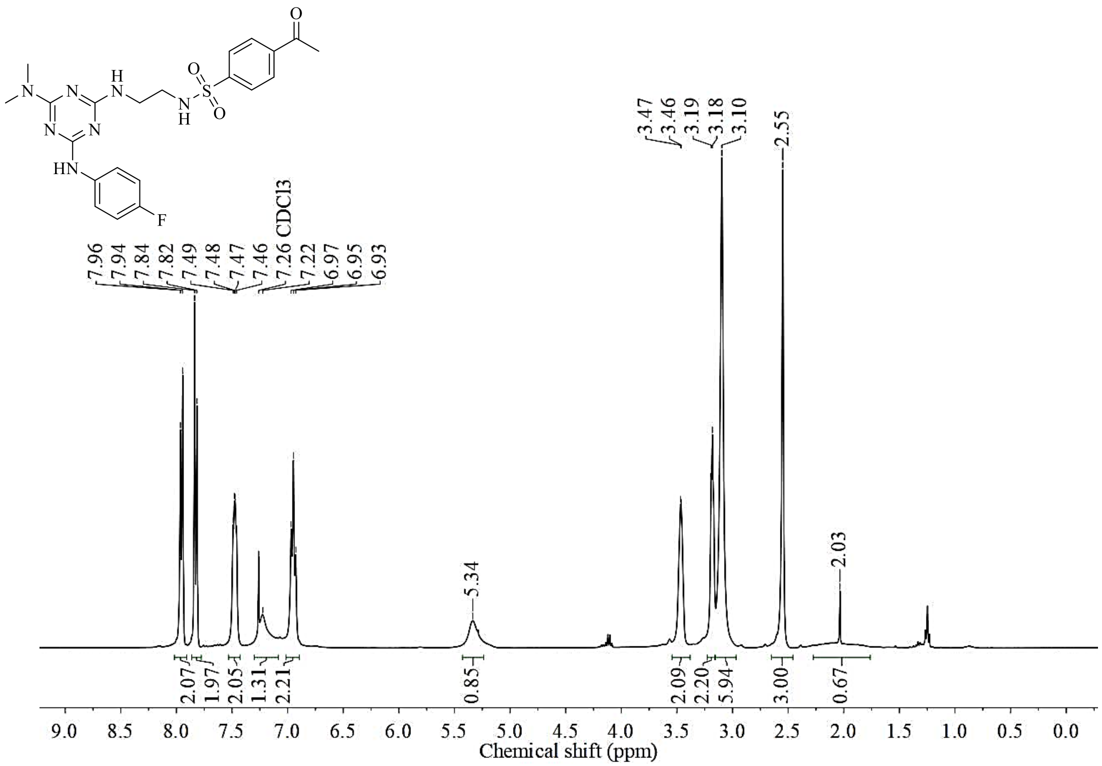

^{1}H NMR (400 MHz, $CDCl_3$) spectra of **18**.

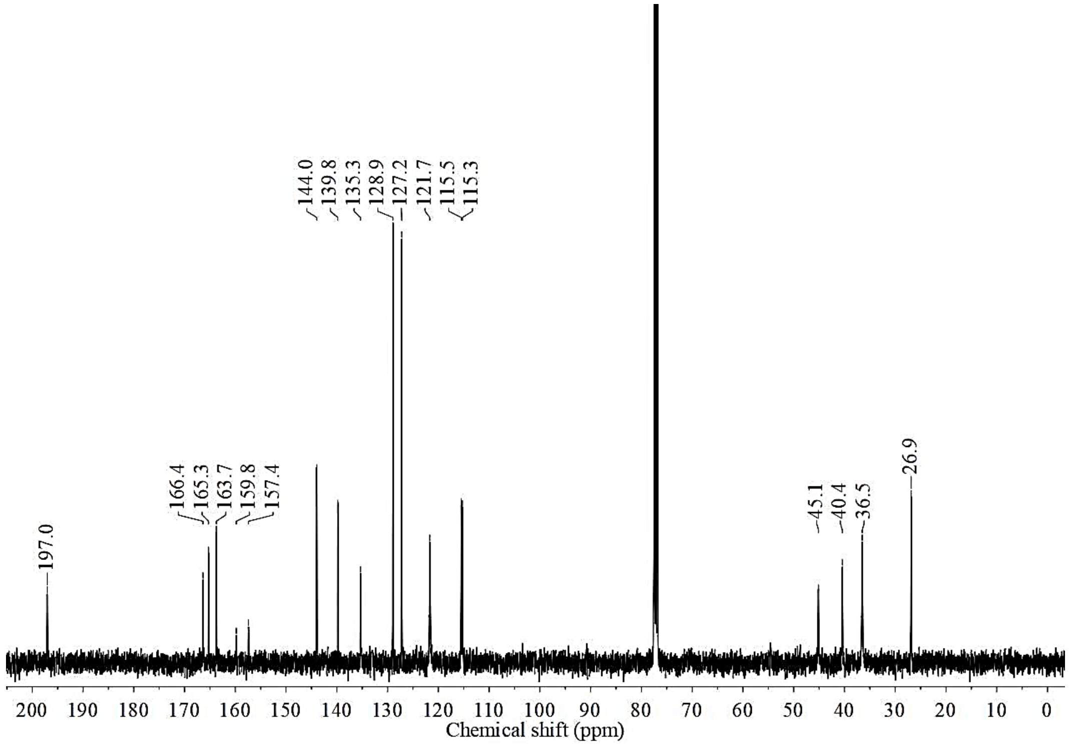

^{13}C NMR (100 MHz, $CDCl_3$) spectra of **18**.

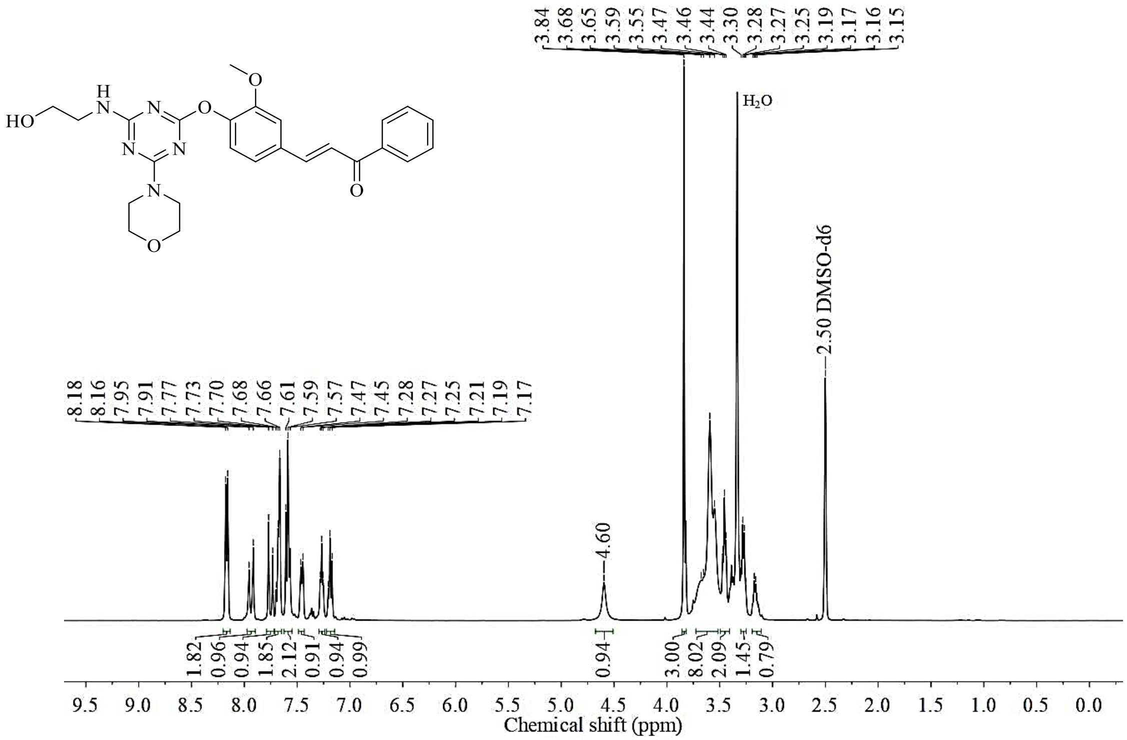

^1H NMR (400 MHz, DMSO-d_6) spectra of **20a**.

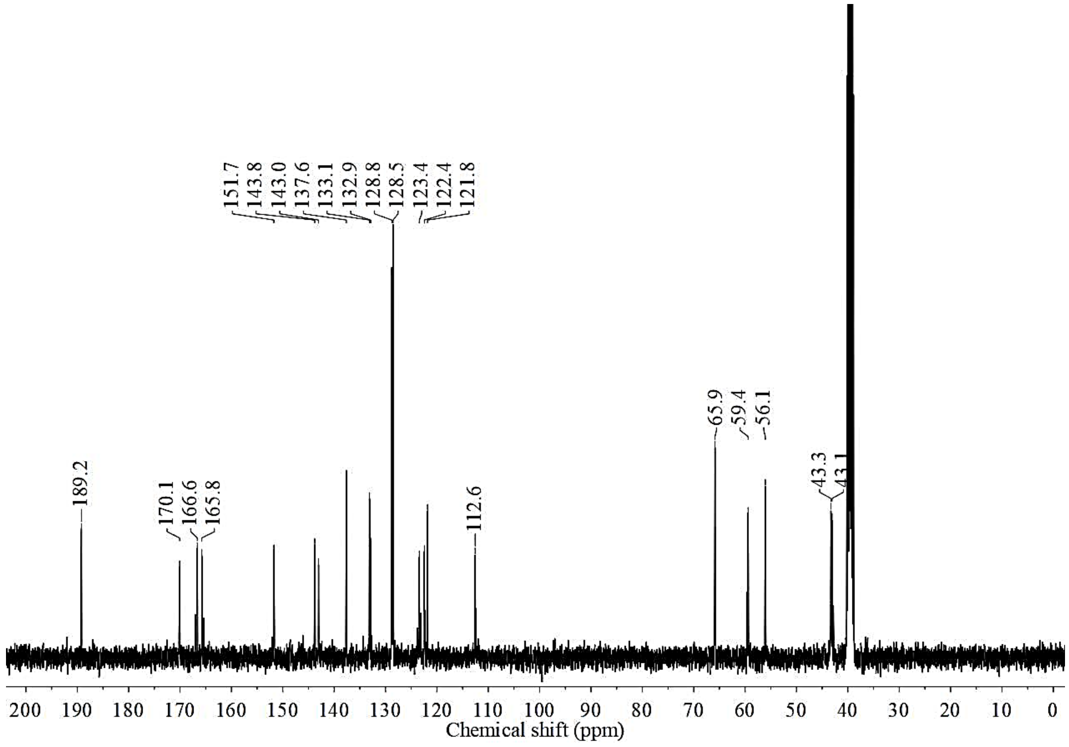

^{13}C NMR (100 MHz, DMSO-d_6) spectra of **20a**.

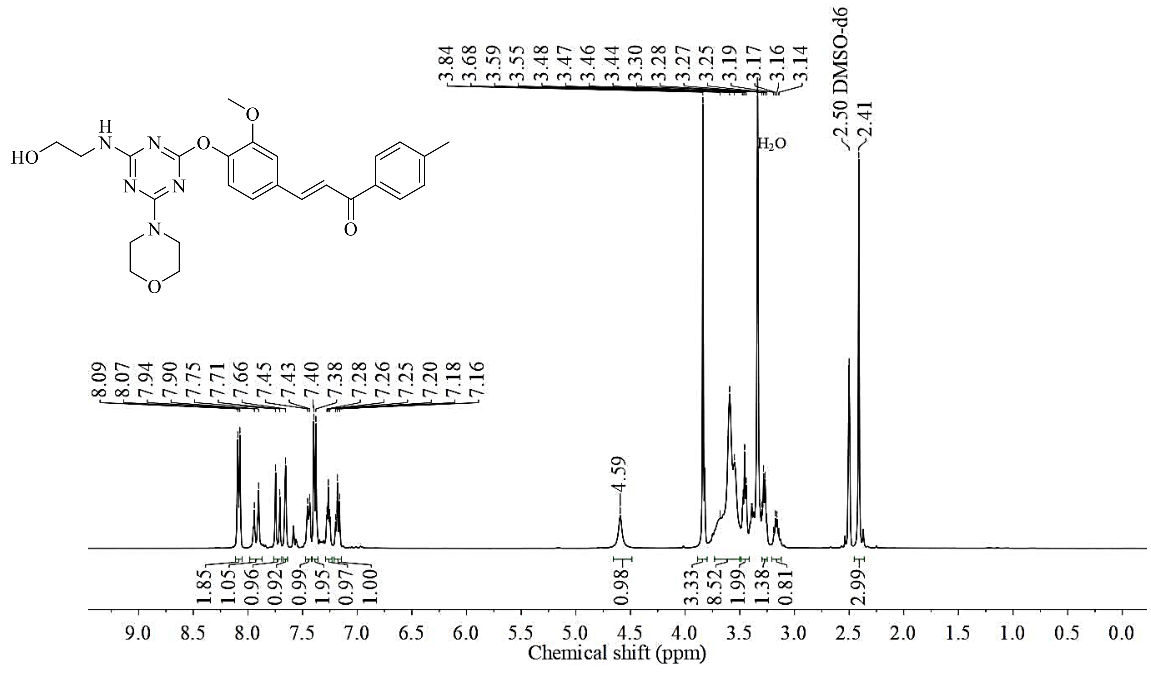

^{1}H NMR (400 MHz, DMSO-d_6) spectra of **20b**.

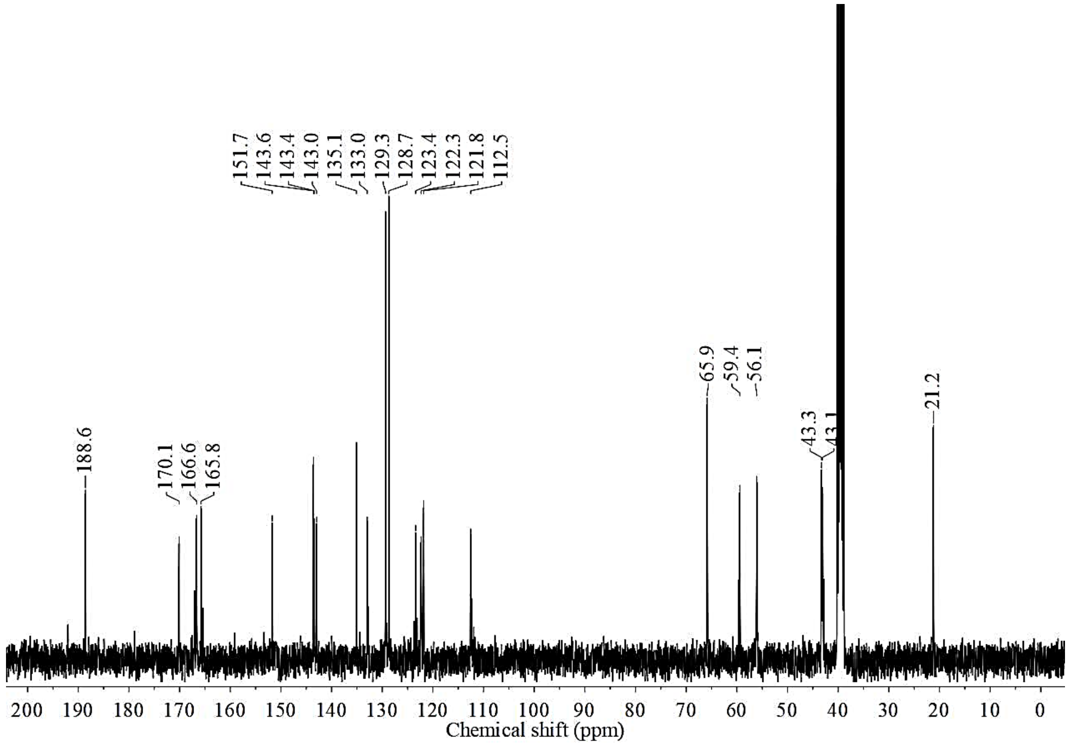

^{13}C NMR (100 MHz, DMSO-d_6) spectra of **20b**.

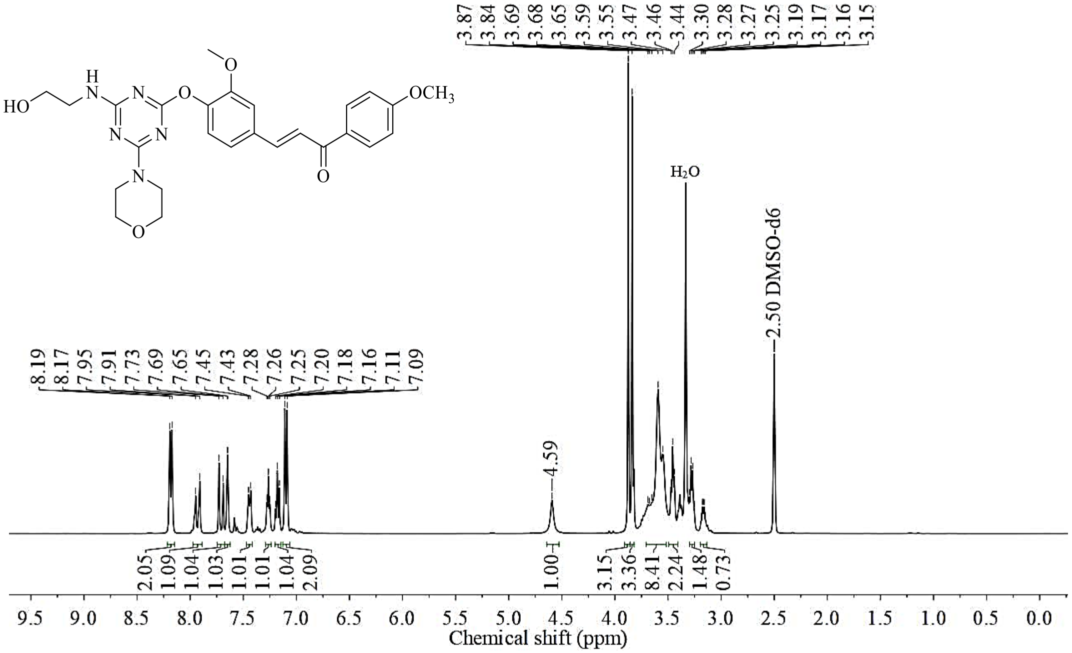

^{1}H NMR (400 MHz, DMSO-d_6) spectra of **20c**.

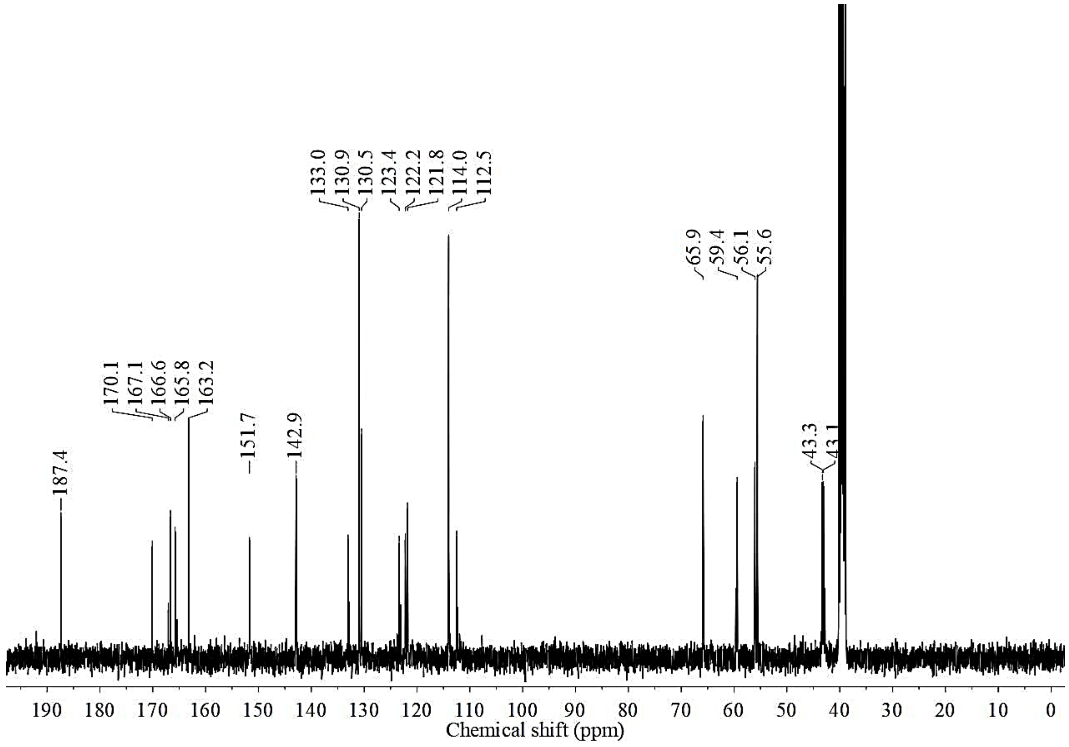

^{13}C NMR (100 MHz, DMSO-d_6) spectra of **20c**.

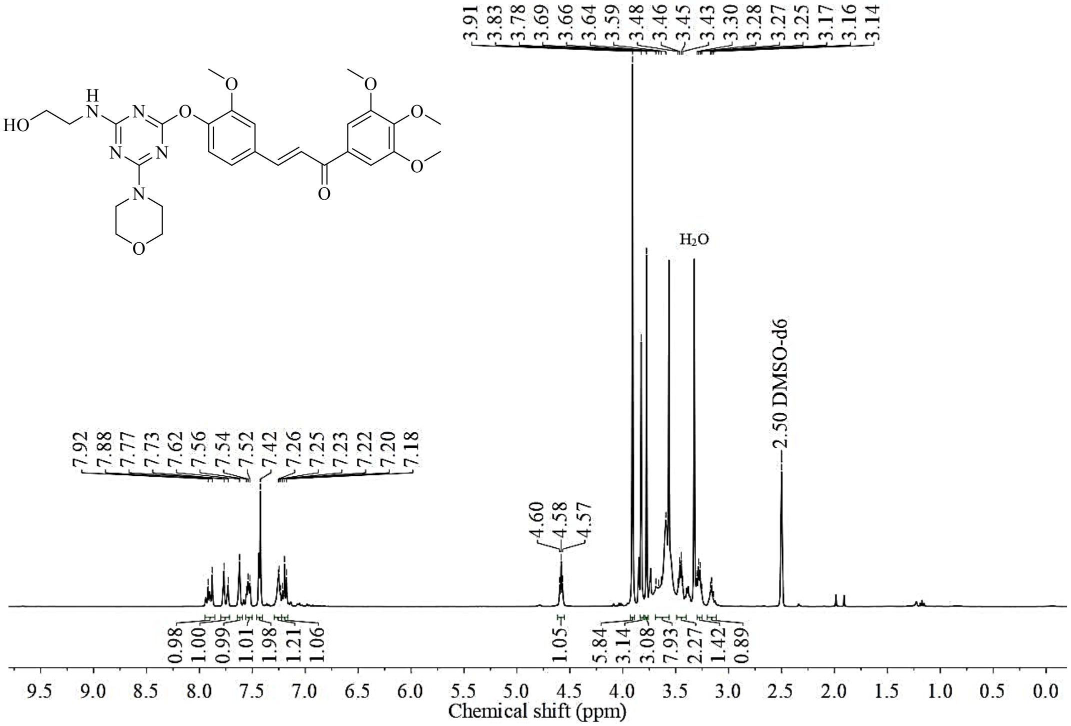

^{1}H NMR (400 MHz, DMSO-*d*6) spectra of **20d**.

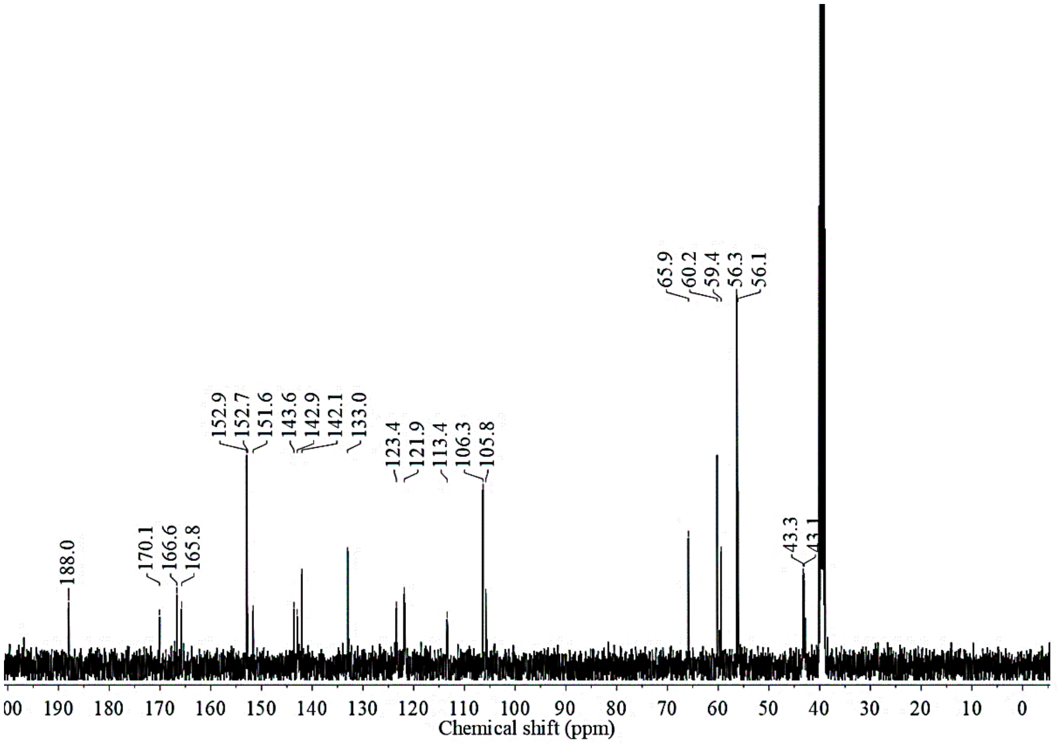

^{13}C NMR (100 MHz, DMSO-*d*6) spectra of **20d**.

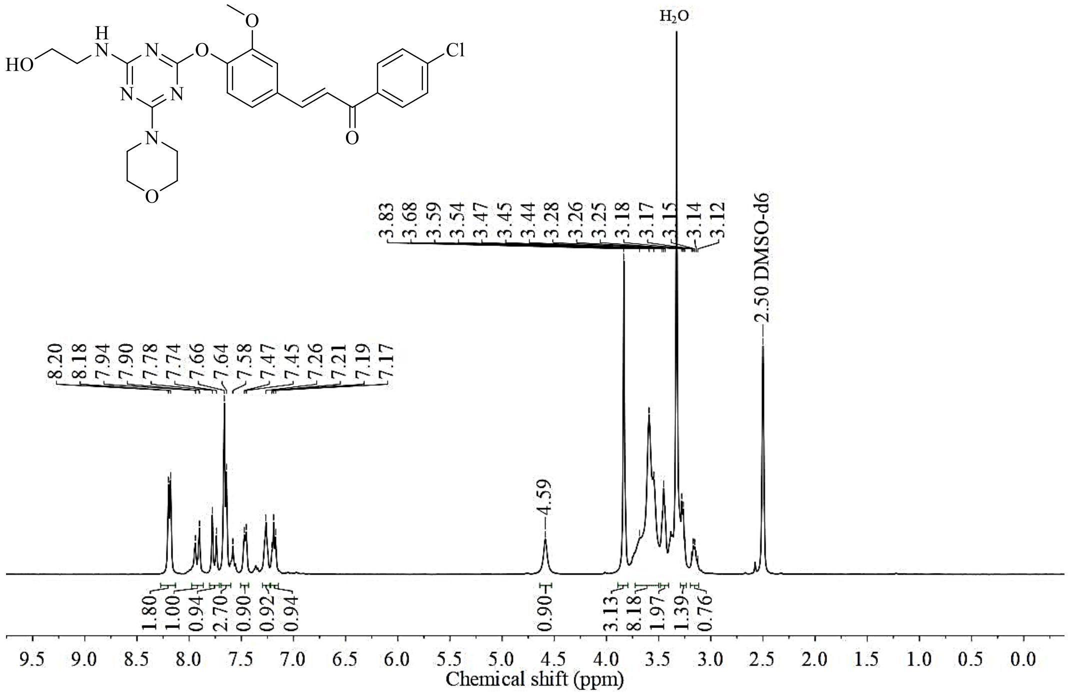

^{1}H NMR (400 MHz, DMSO-*d*$_6$) spectra of **20e**.

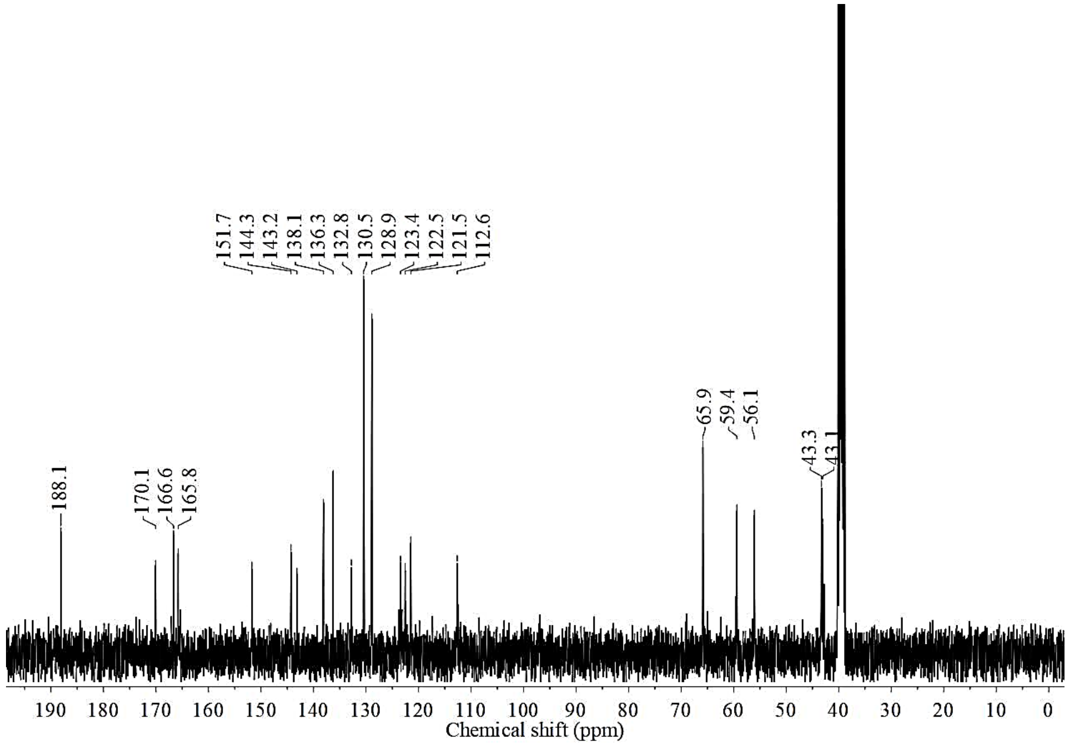

^{13}C NMR (100 MHz, DMSO-*d*$_6$) spectra of **20e**.

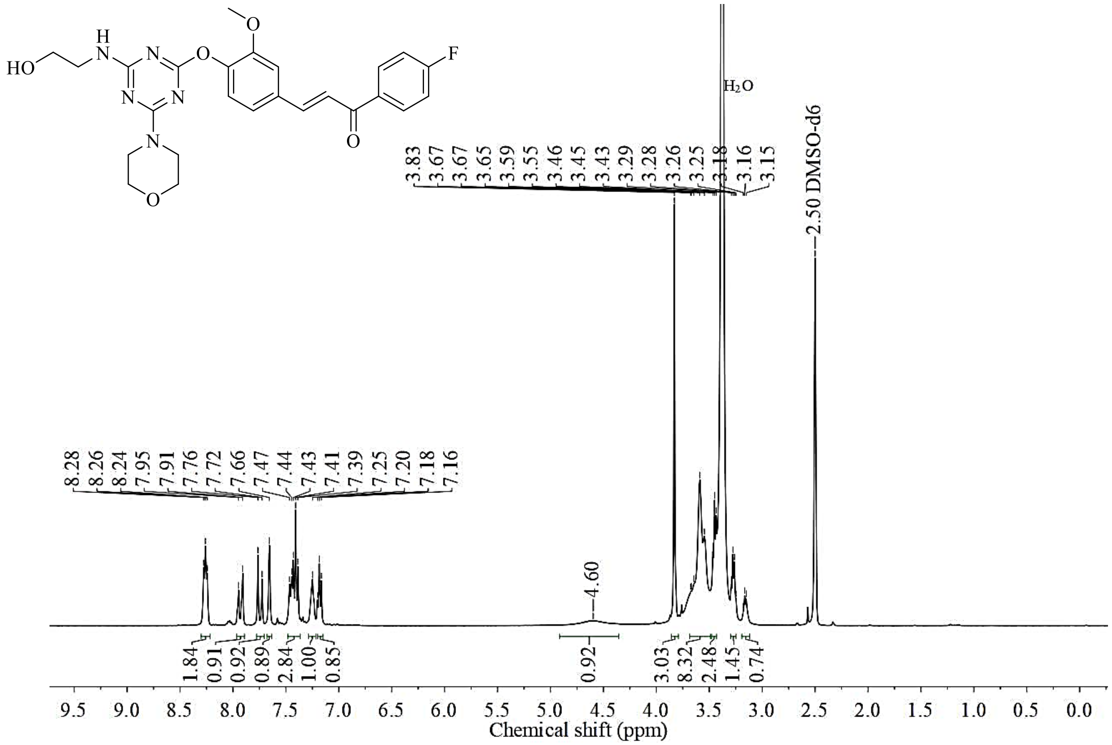

^{1}H NMR (400 MHz, DMSO-d_6) spectra of **20f**.

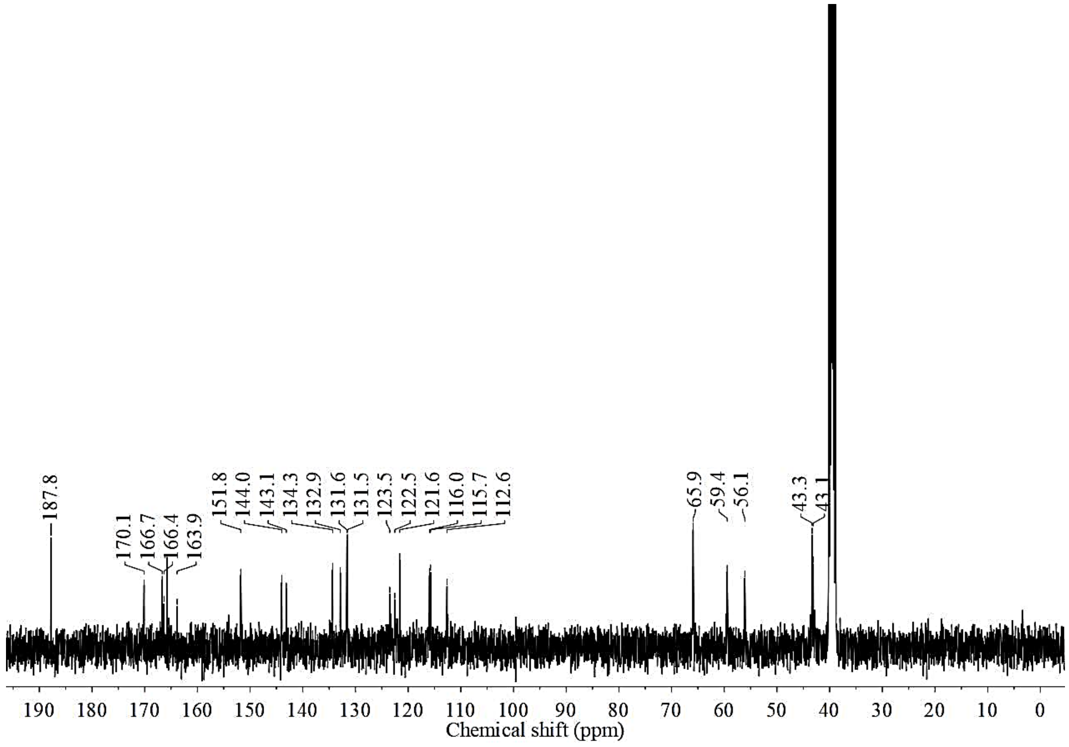

^{13}C NMR (100 MHz, DMSO-d_6) spectra of **20f**.

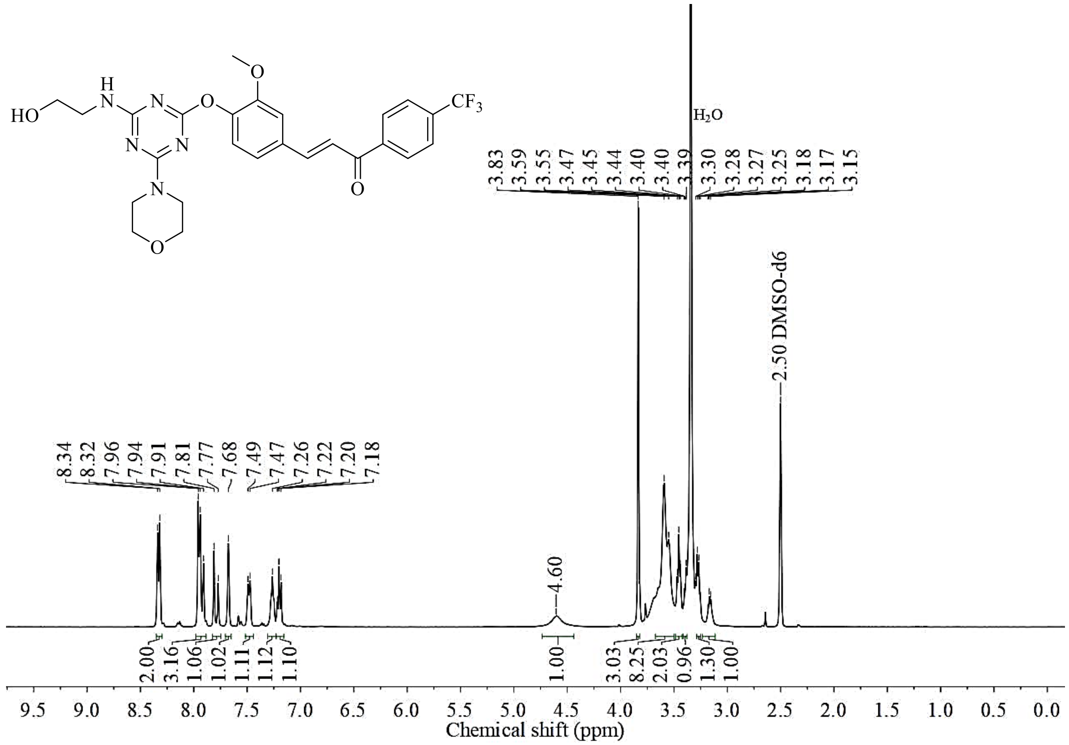

^{1}H NMR (400 MHz, DMSO-d_6) spectra of **20g**.

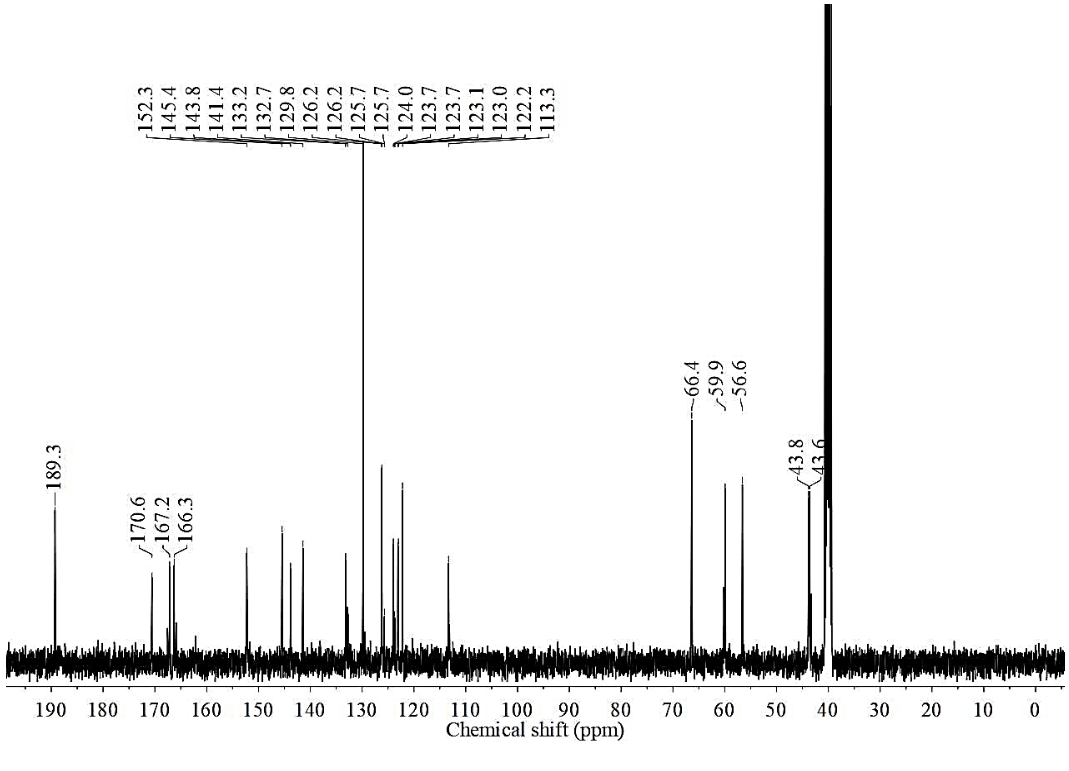

^{13}C NMR (100 MHz, DMSO-d_6) spectra of **20g**.

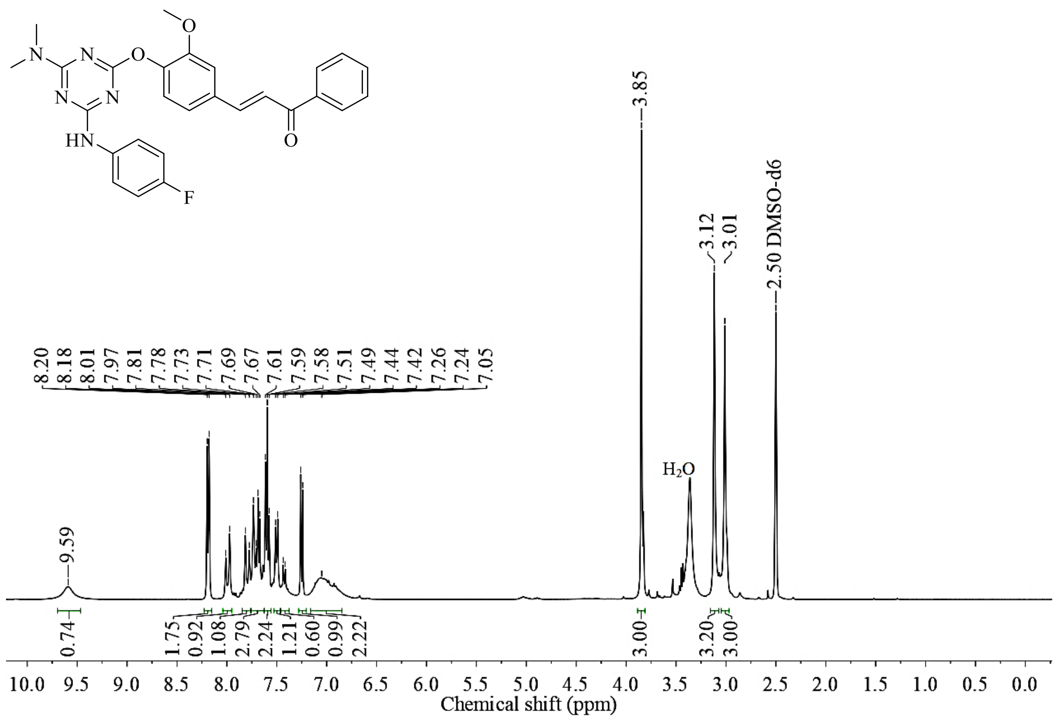

^{1}H NMR (400 MHz, DMSO-d_6) spectra of **21a**.

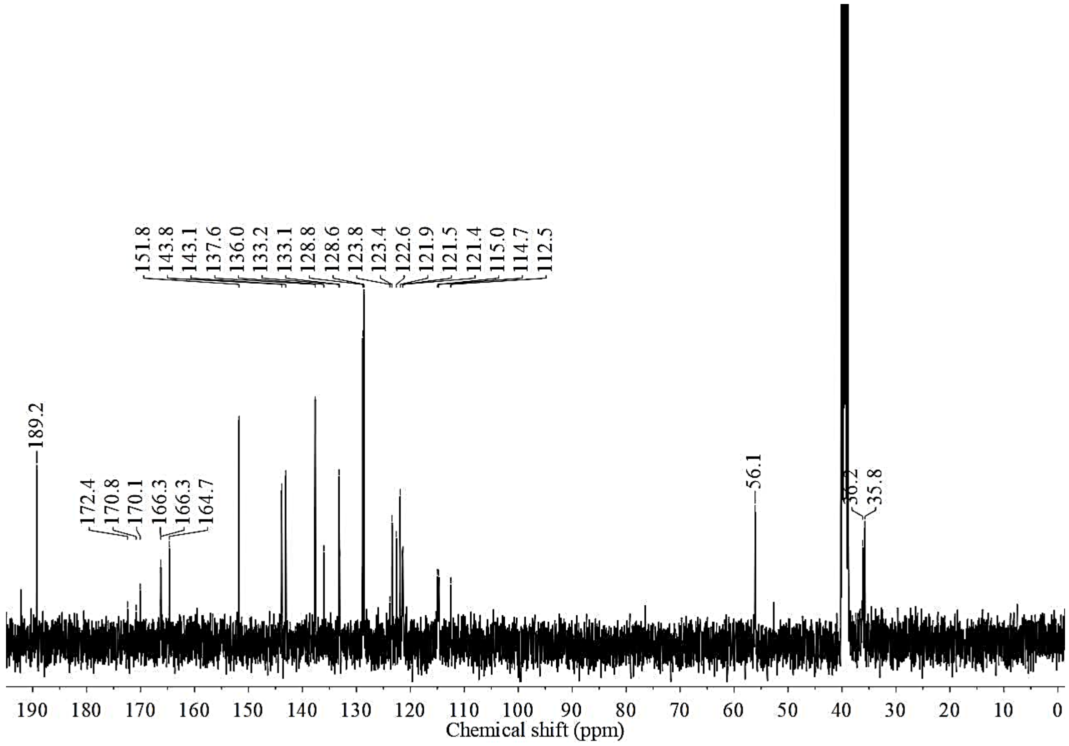

^{13}C NMR (100 MHz, DMSO-d_6) spectra of **21a**.

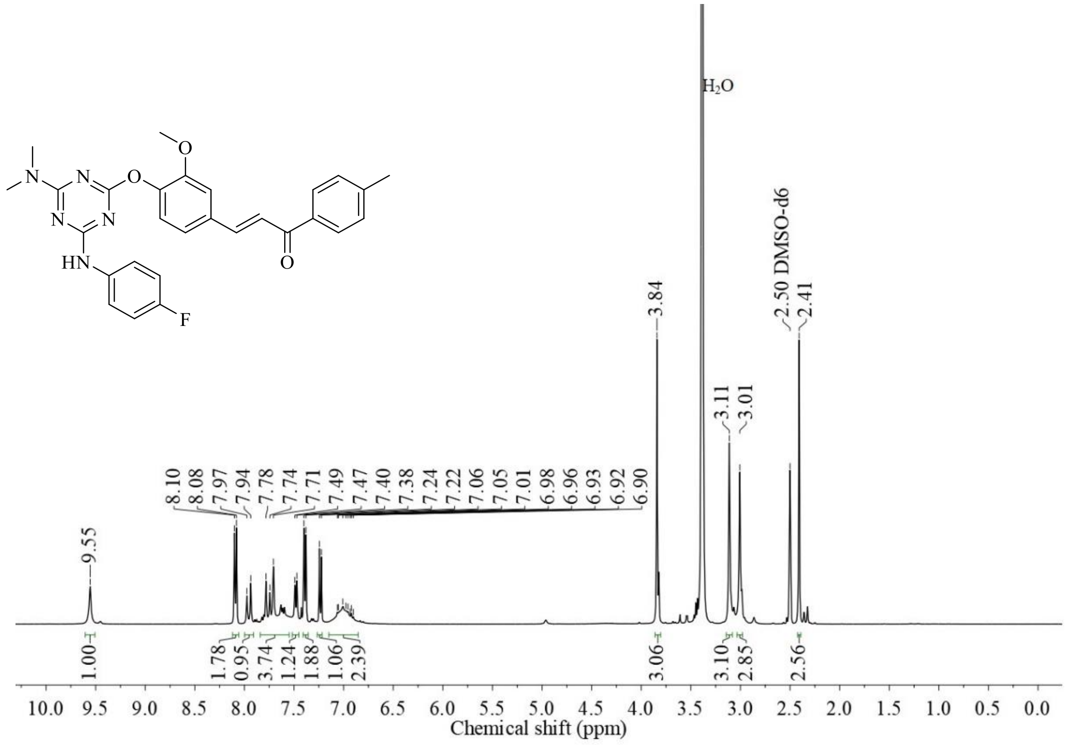

^{1}H NMR (400 MHz, DMSO-d_6) spectra of **21b**.

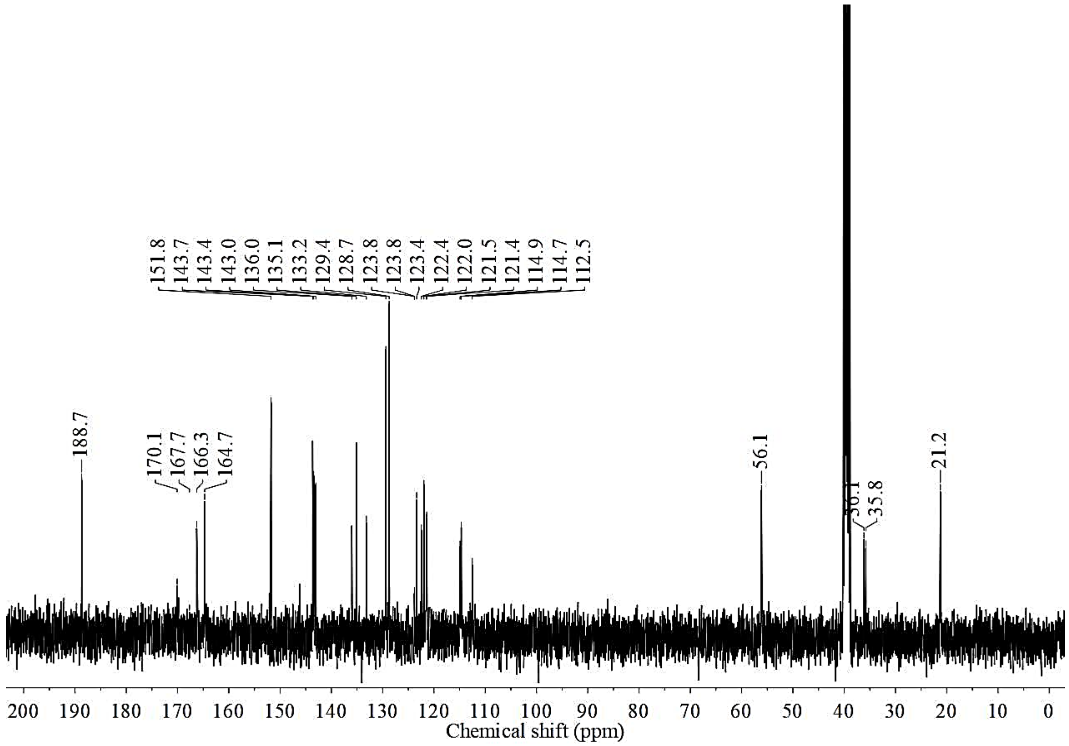

^{13}C NMR (100 MHz, DMSO-d_6) spectra of **21b**.

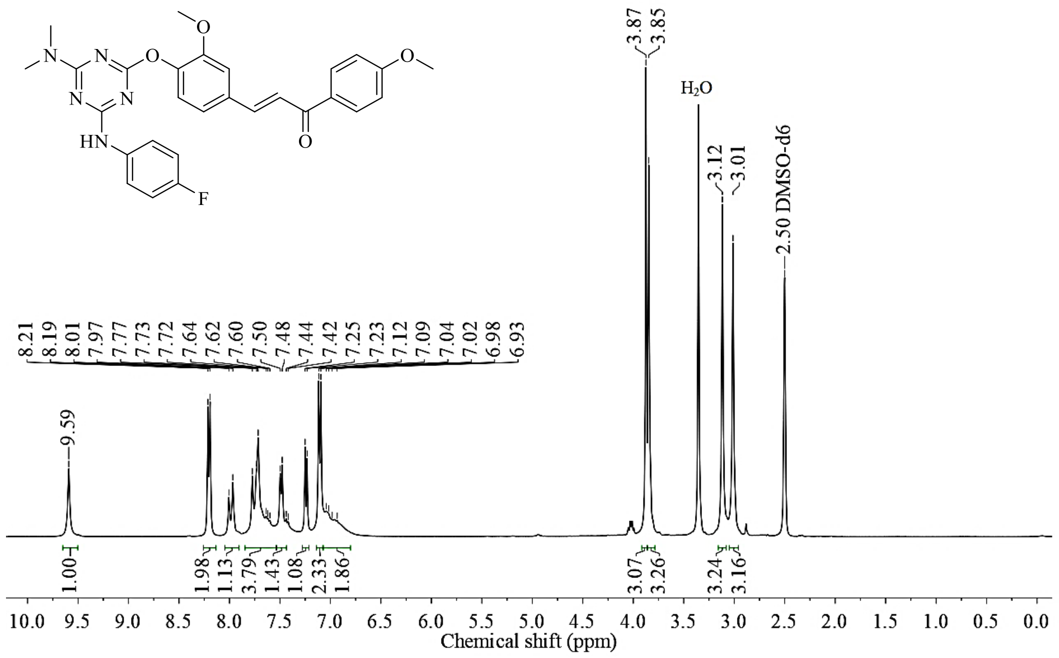

^1H NMR (400 MHz, DMSO-d_6) spectra of **21c**.

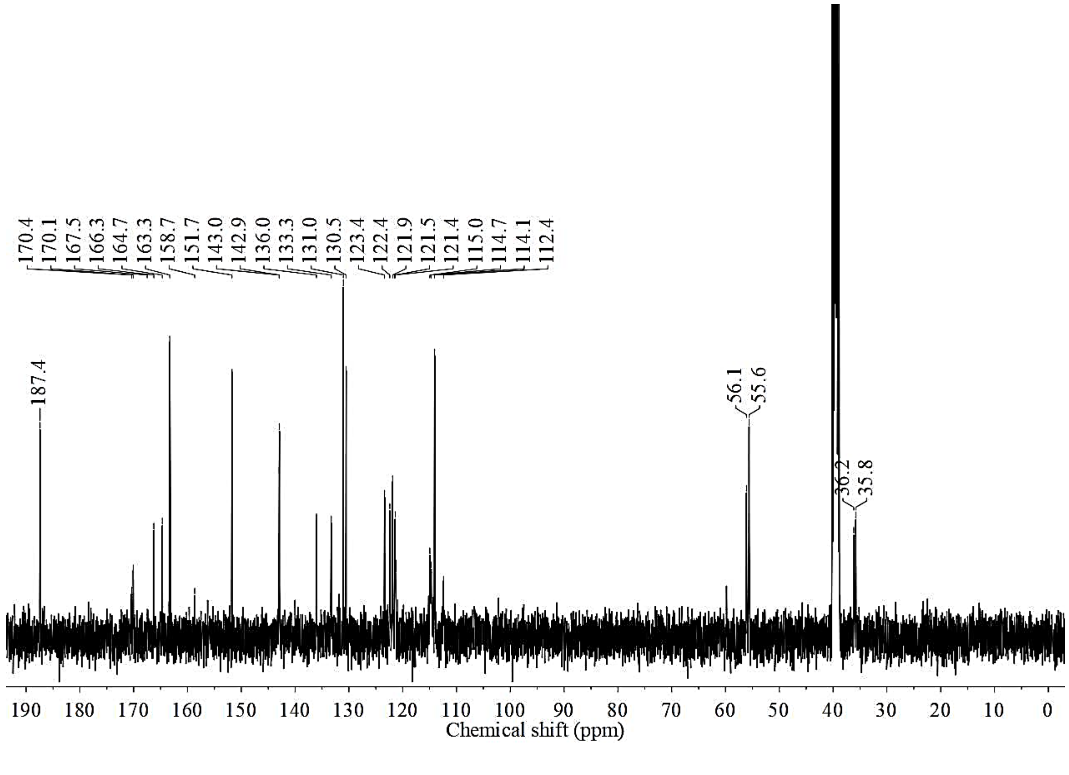

^{13}C NMR (100 MHz, DMSO-d_6) spectra of **21c**.

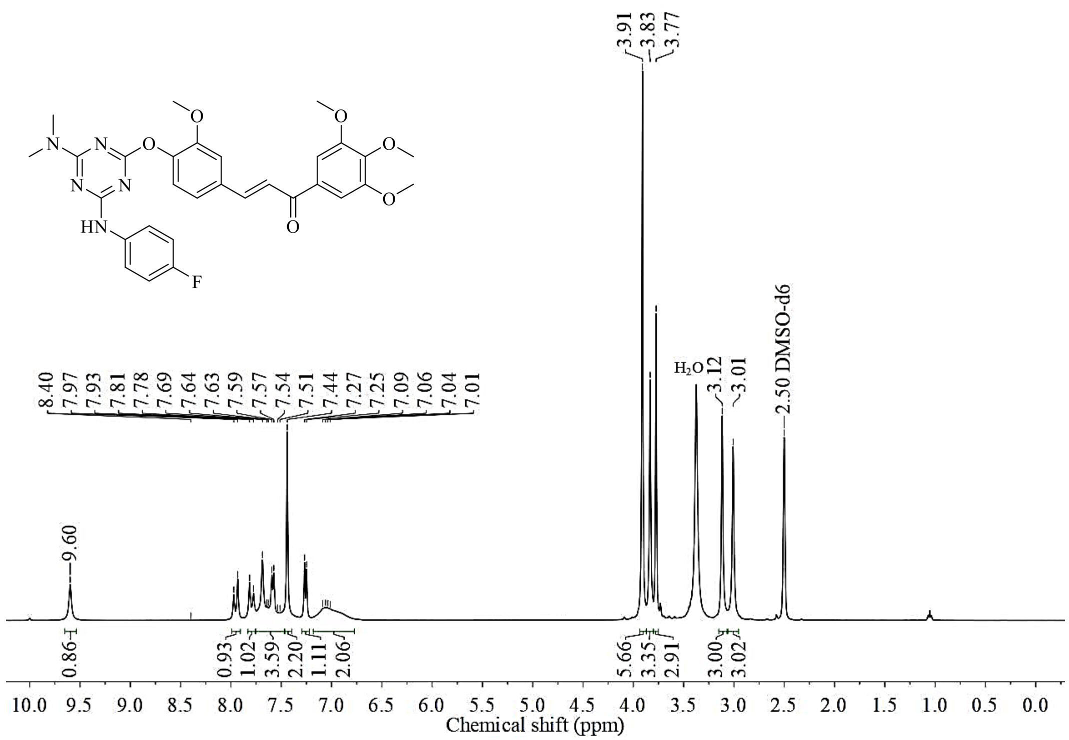

^{1}H NMR (400 MHz, DMSO-d_6) spectra of **21d**.

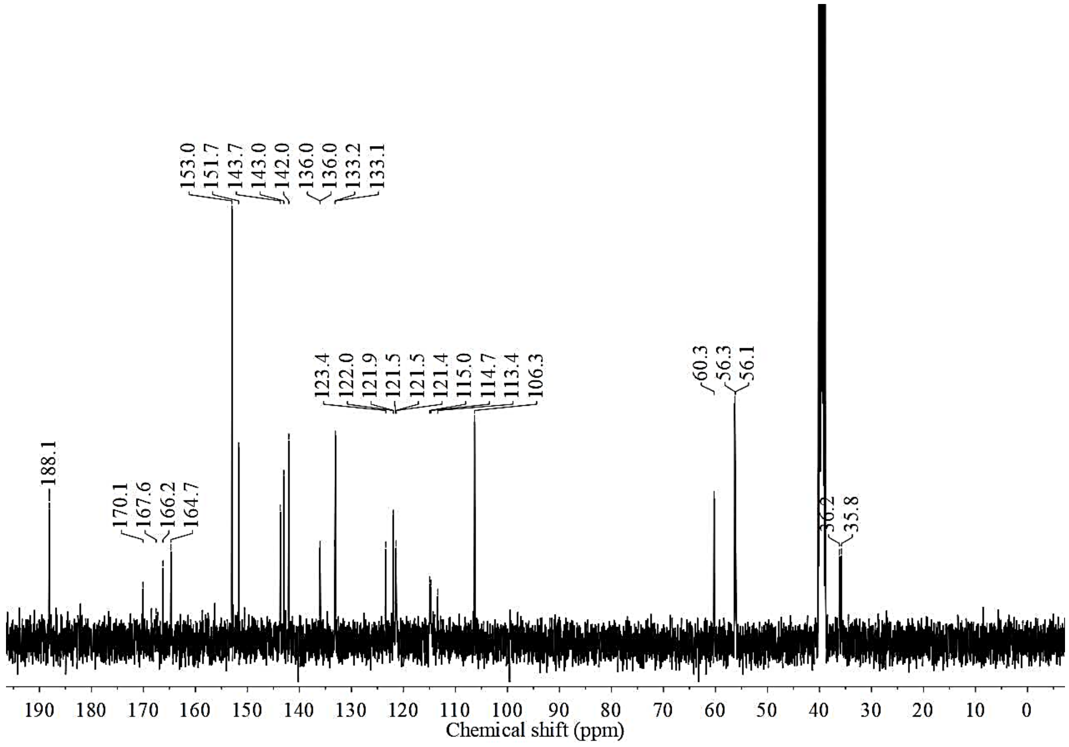

^{13}C NMR (100 MHz, DMSO-d_6) spectra of **21d**.

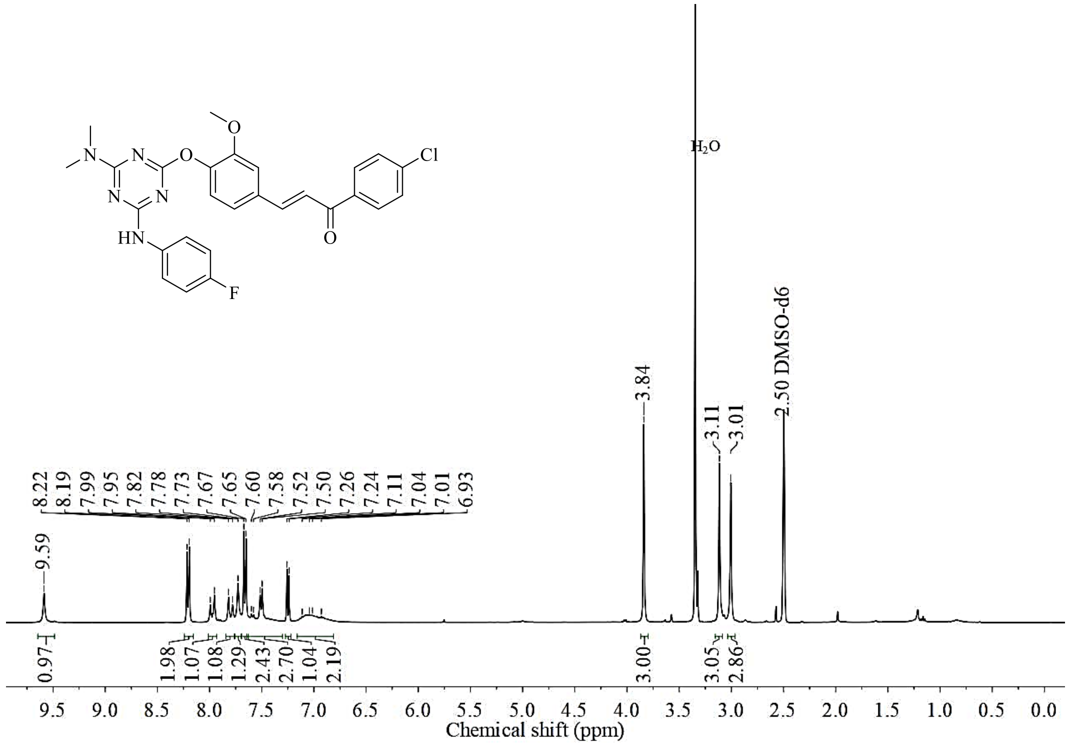

^{1}H NMR (400 MHz, DMSO-d_6) spectra of **21e**.

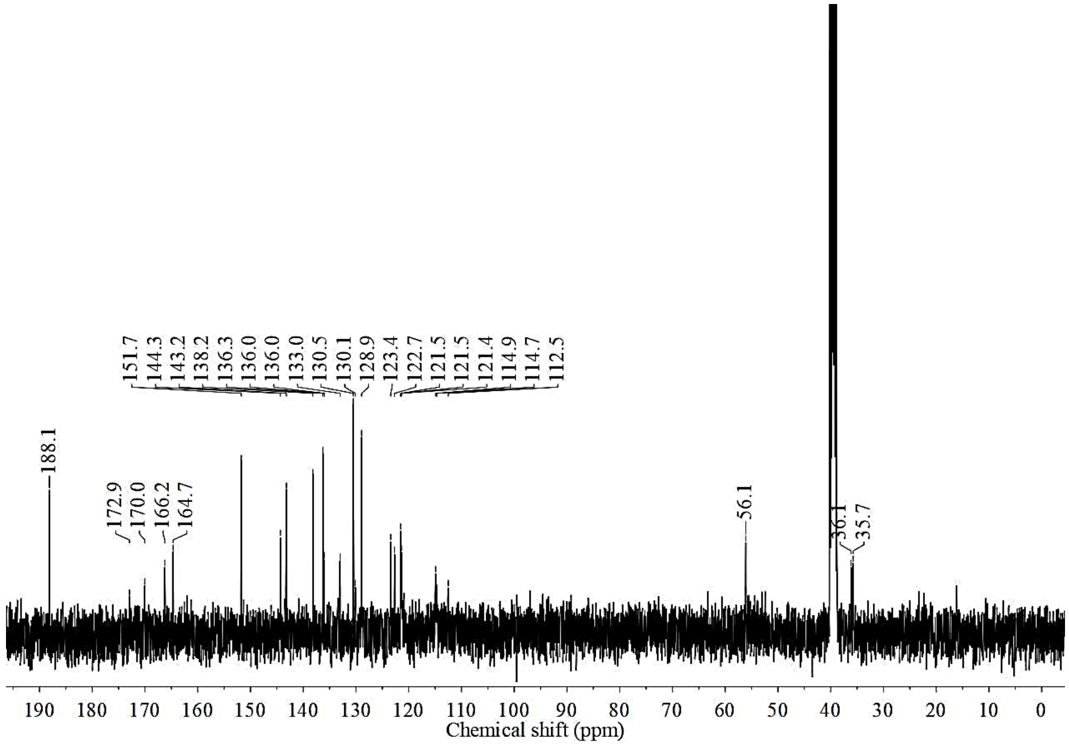

^{13}C NMR (100 MHz, DMSO-d_6) spectra of **21e**.

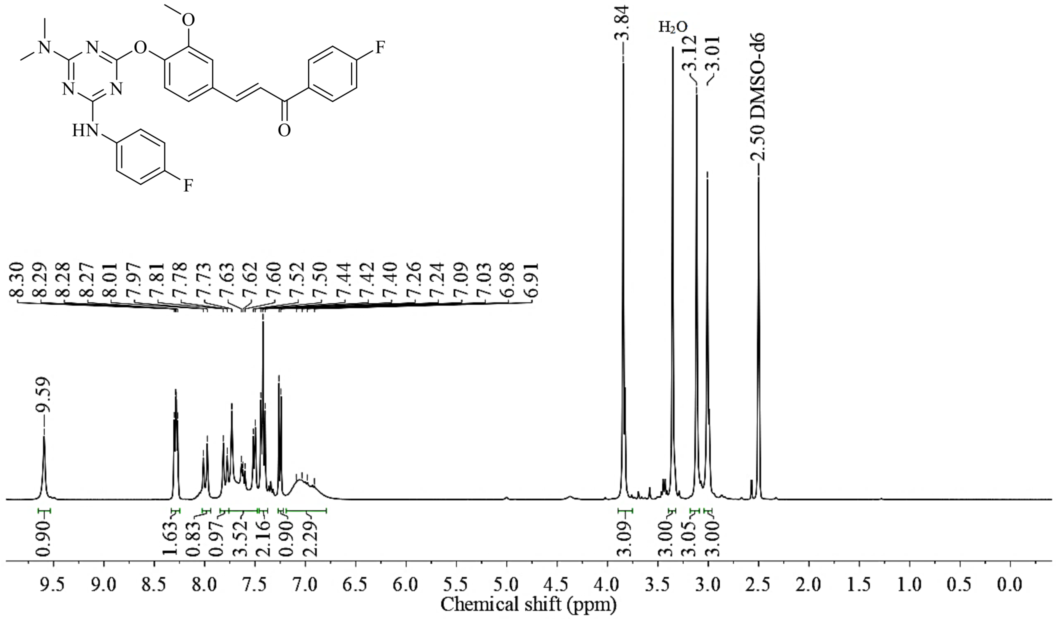

^{1}H NMR (400 MHz, DMSO-d_6) spectra of **21f**.

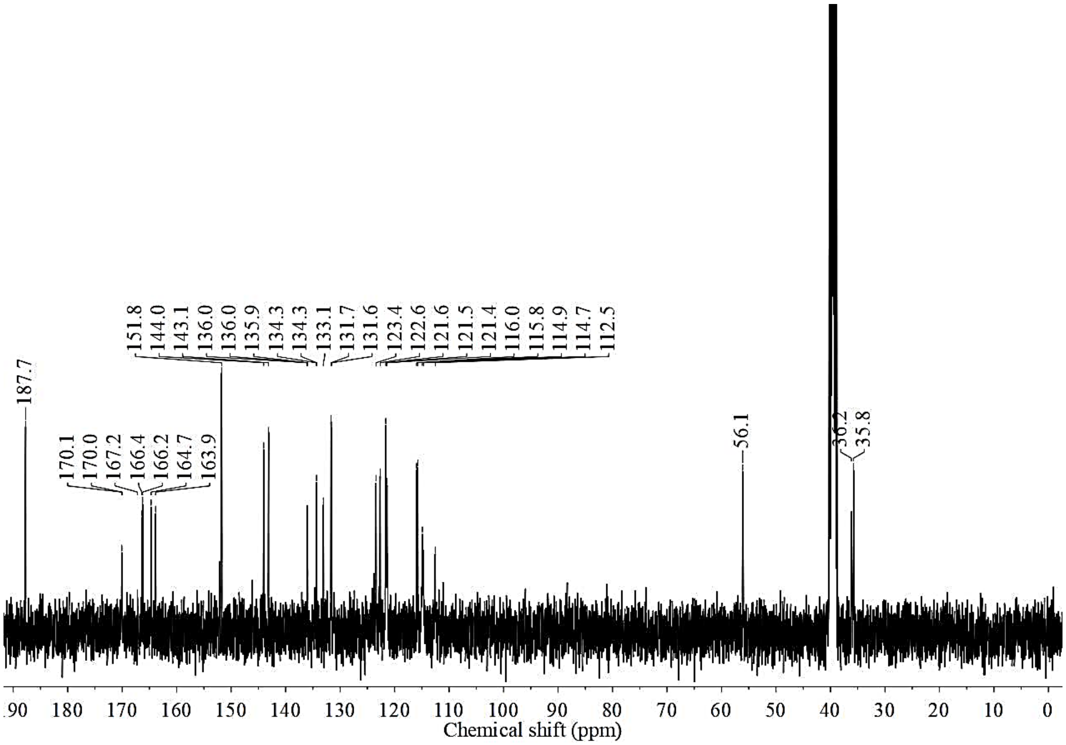

^{13}C NMR (100 MHz, DMSO-d_6) spectra of **21f**.

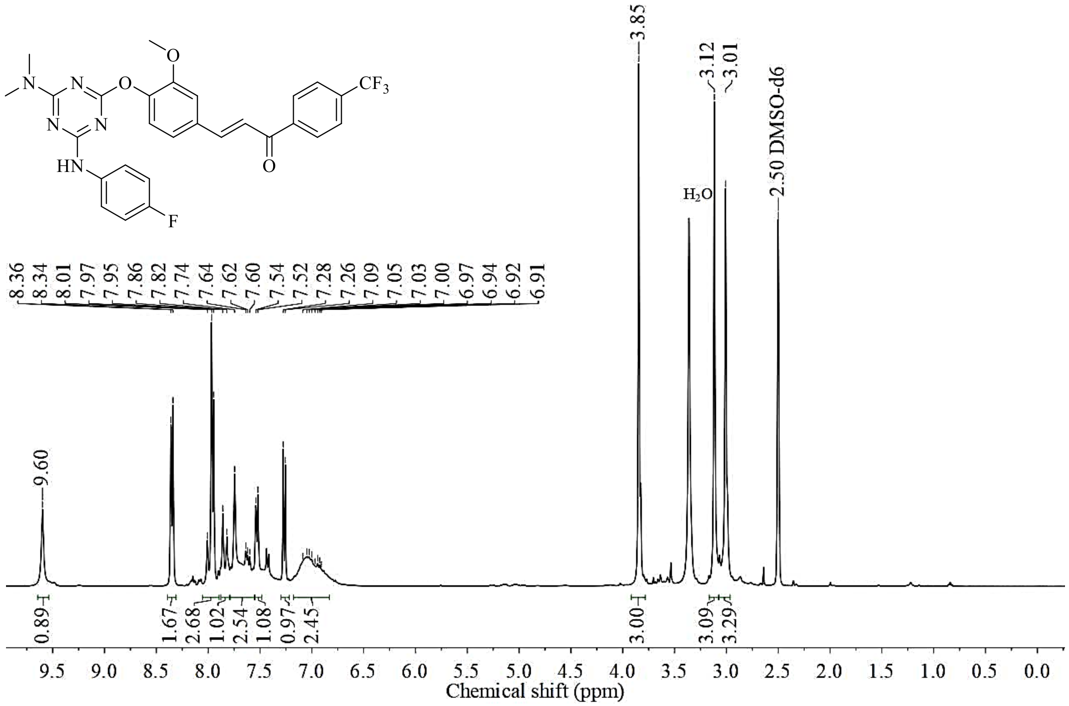

^{1}H NMR (400 MHz, DMSO-d_6) spectra of **21g**.

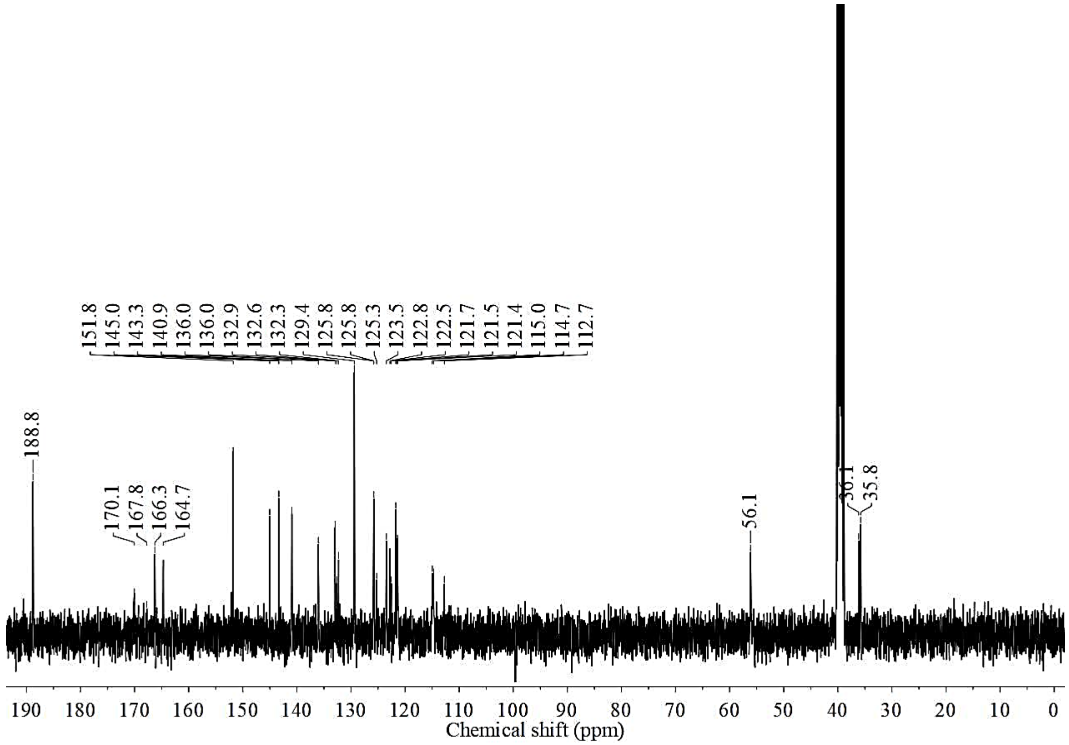

^{13}C NMR (100 MHz, DMSO-d_6) spectra of **21g**.

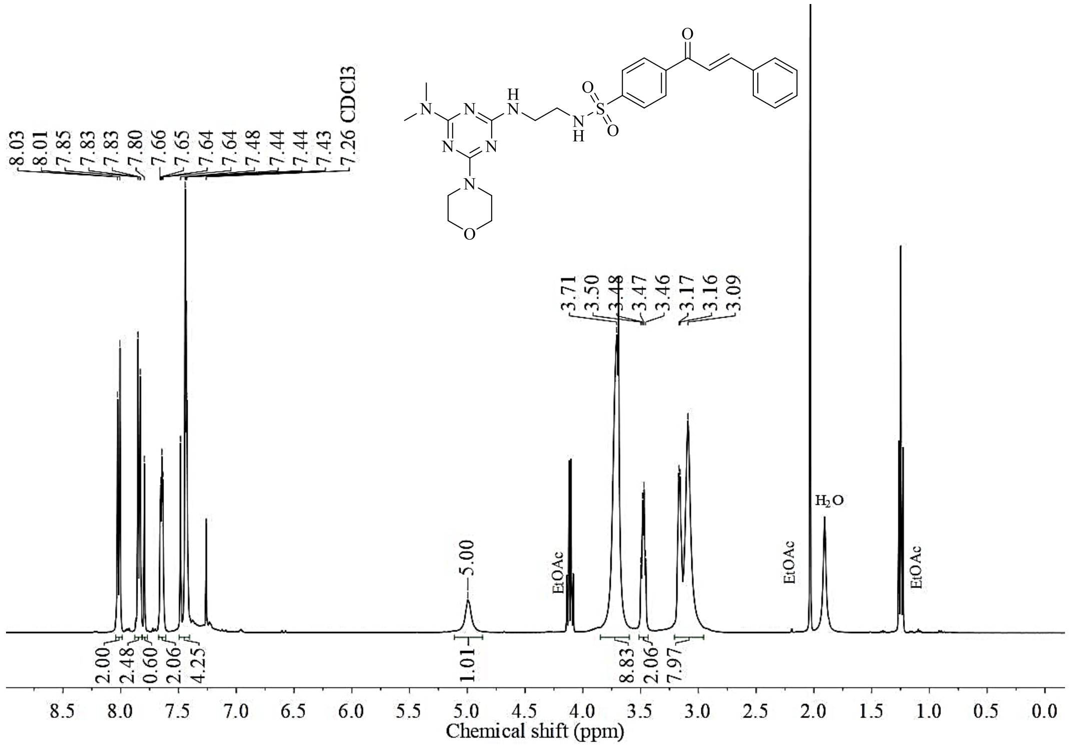

^{1}H NMR (400 MHz, $CDCl_3$) spectra of **23a**.

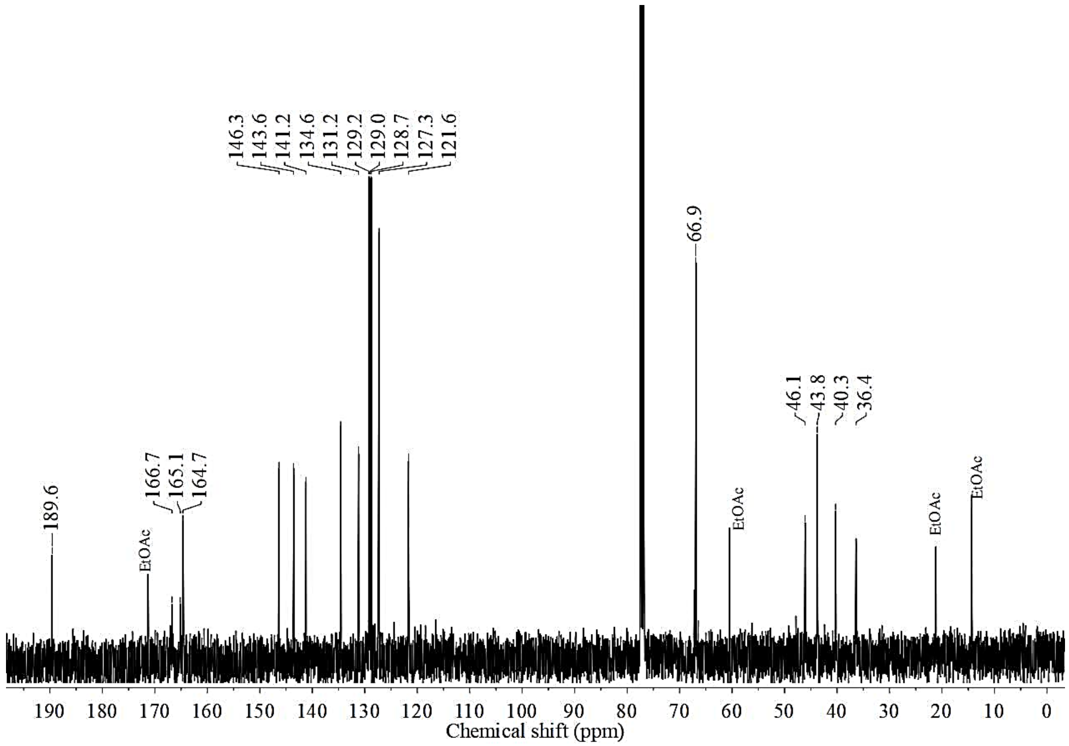

^{13}C NMR (100 MHz, $CDCl_3$) spectra of **23a**.

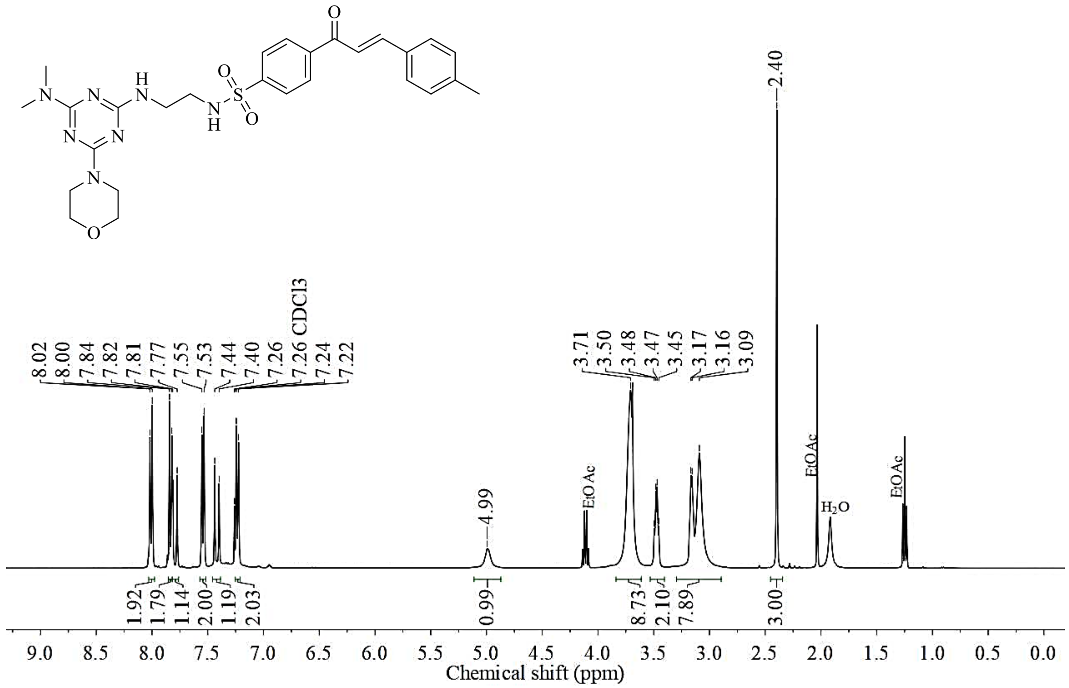

^{1}H NMR (400 MHz, $CDCl_3$) spectra of **23b**.

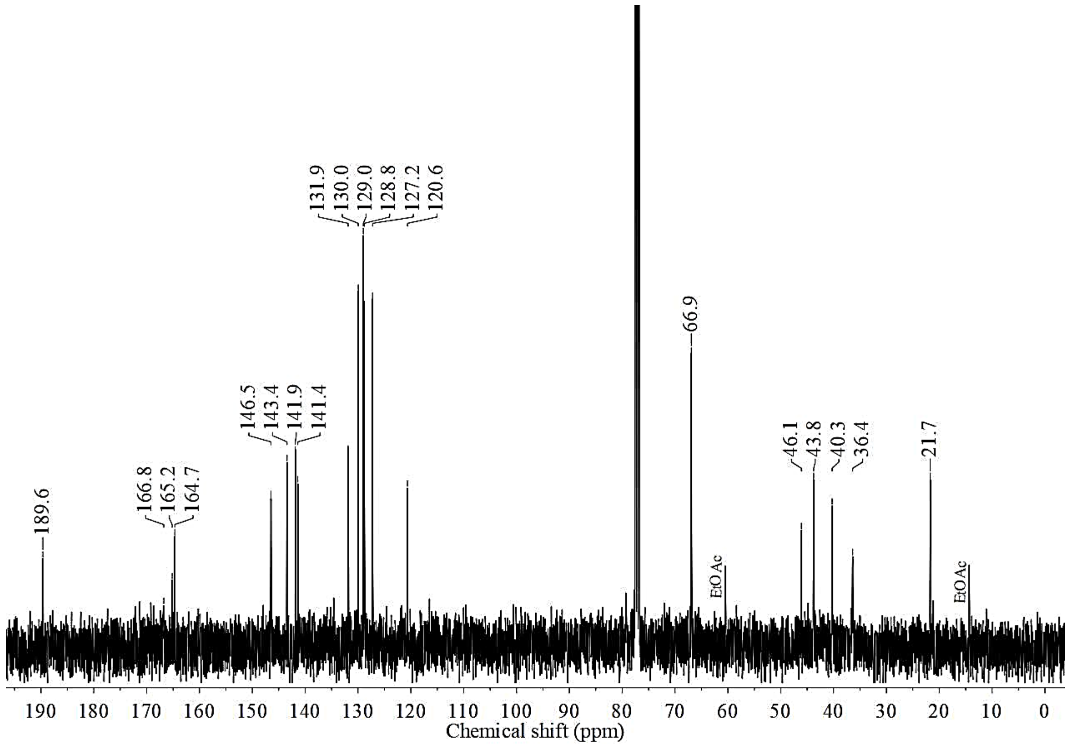

^{13}C NMR (100 MHz, $CDCl_3$) spectra of **23b**.

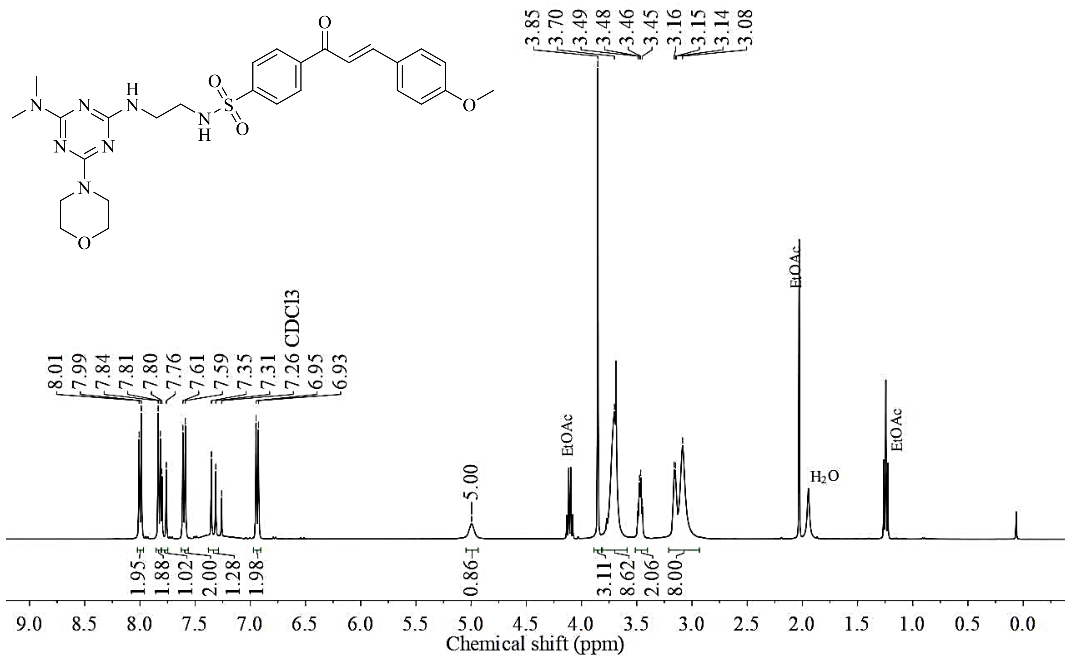

^{1}H NMR (400 MHz, $CDCl_3$) spectra of **23c**.

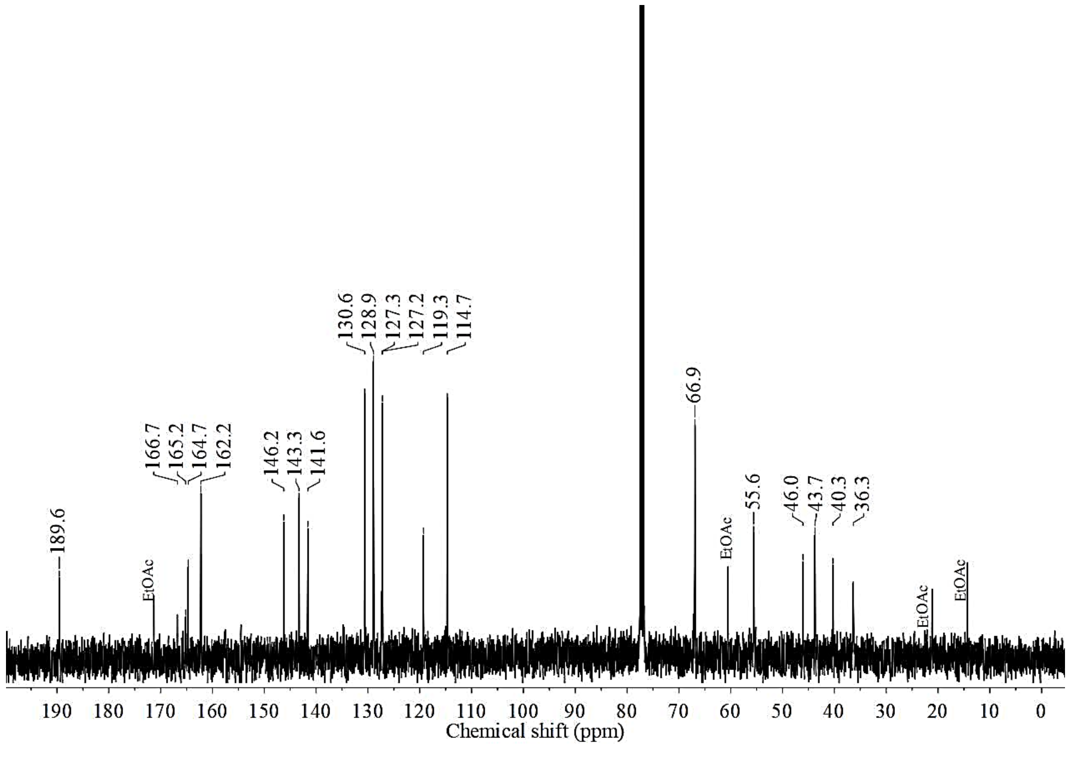

^{13}C NMR (100 MHz, $CDCl_3$) spectra of **23c**.

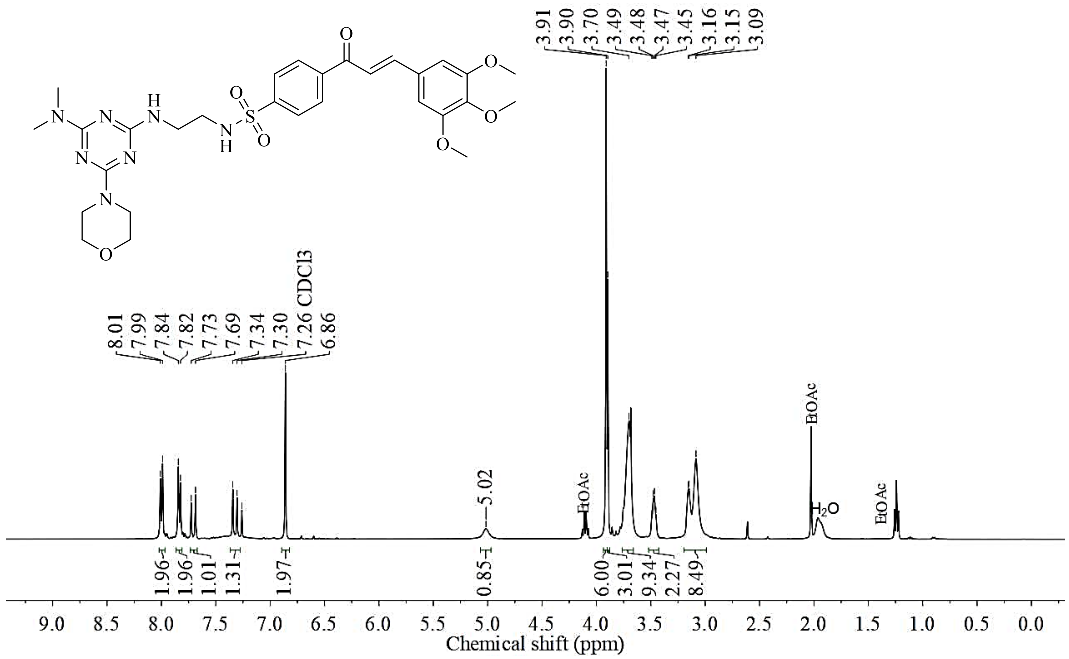

^{1}H NMR (400 MHz, $CDCl_3$) spectra of **23d**.

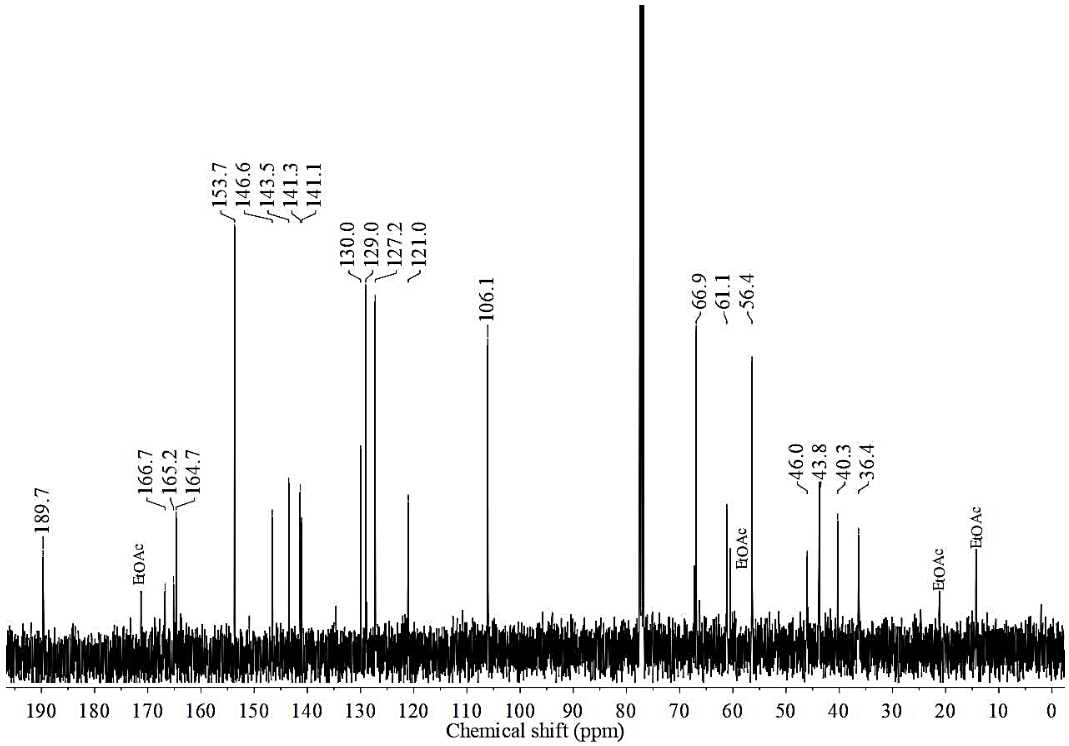

^{13}C NMR (100 MHz, $CDCl_3$) spectra of **23d**.

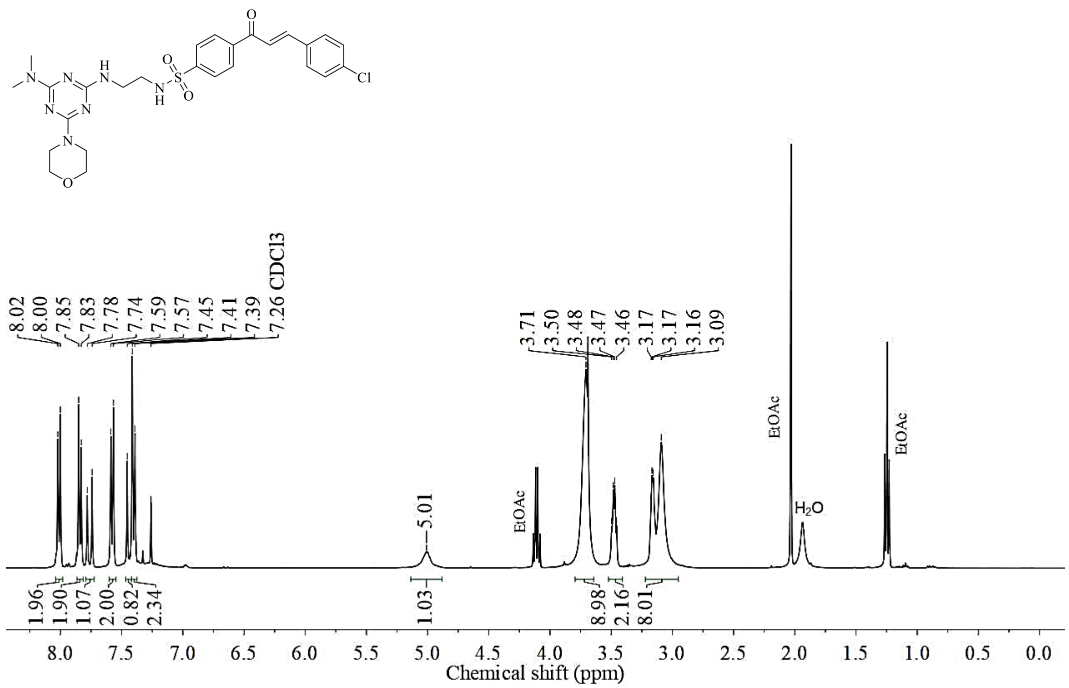

^{1}H NMR (400 MHz, $CDCl_3$) spectra of **23e**.

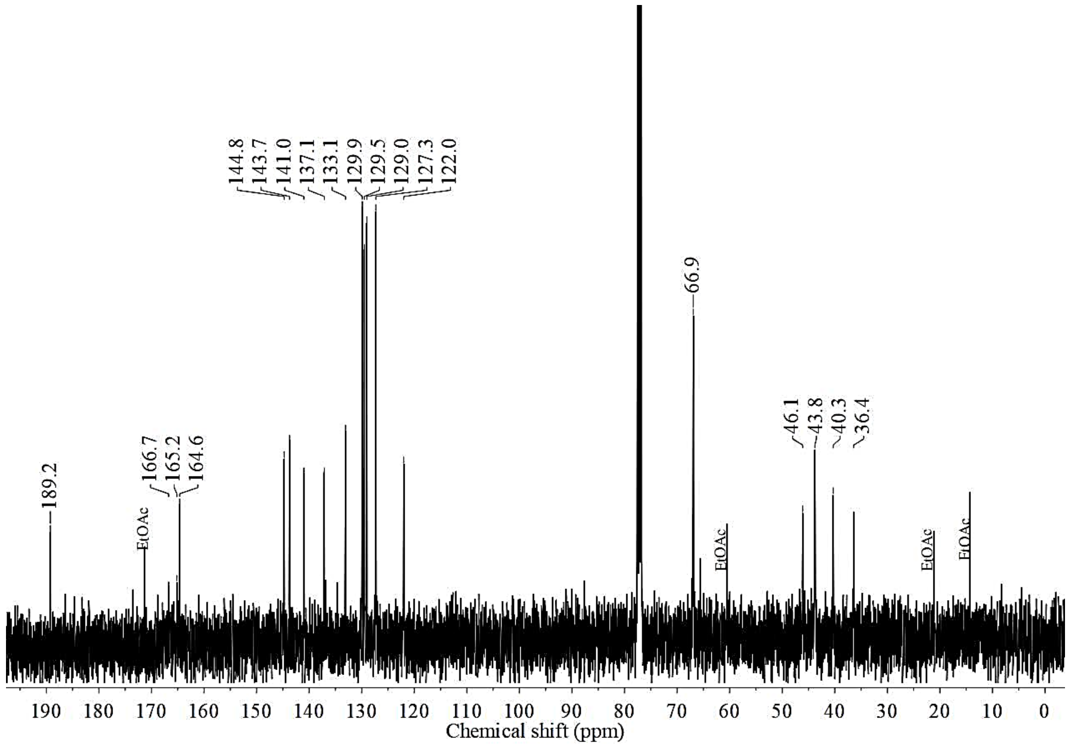

^{13}C NMR (100 MHz, $CDCl_3$) spectra of **23e**.

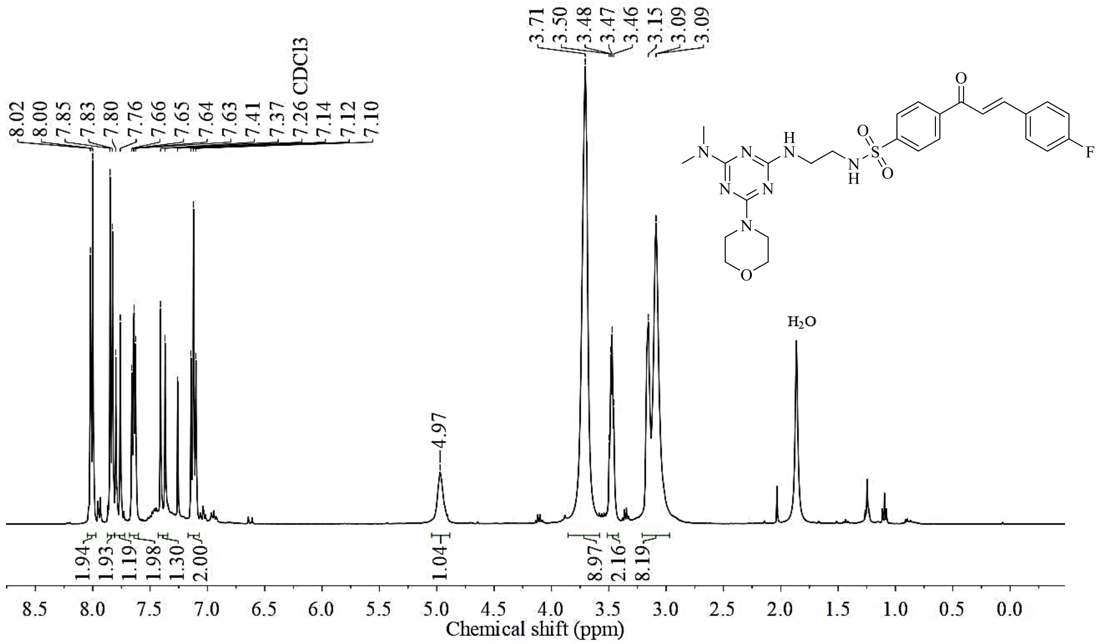

^{1}H NMR (400 MHz, CDCl3) spectra of **23f**.

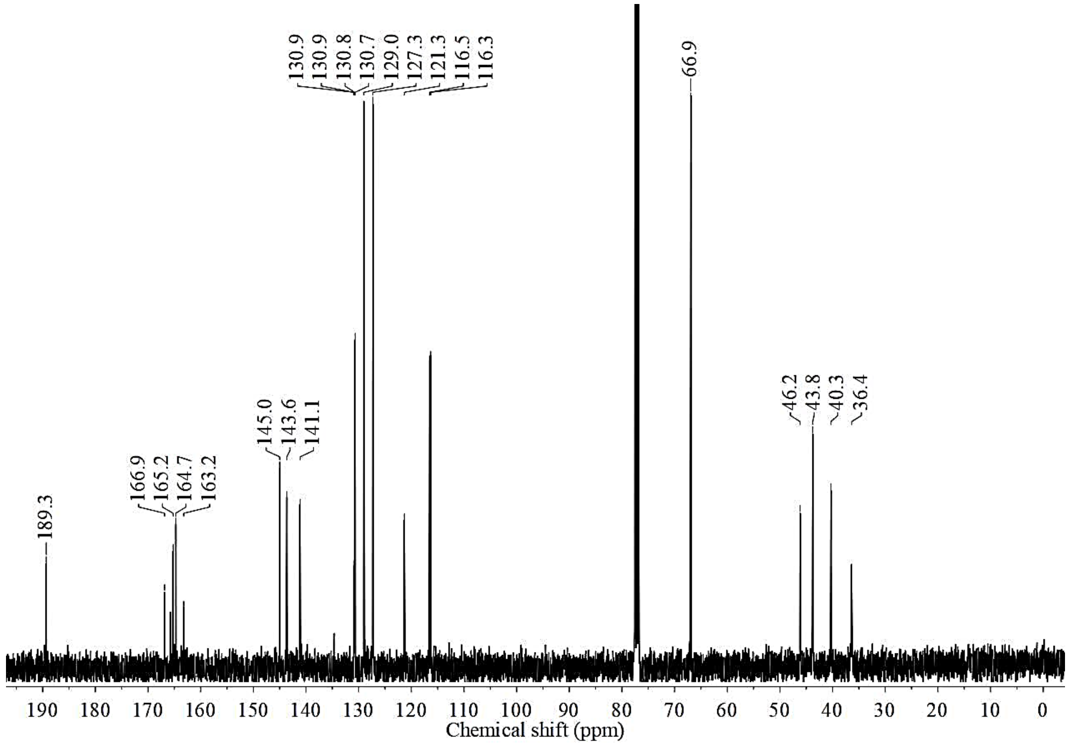

^{13}C NMR (100 MHz, CDCl3) spectra of **23f**.

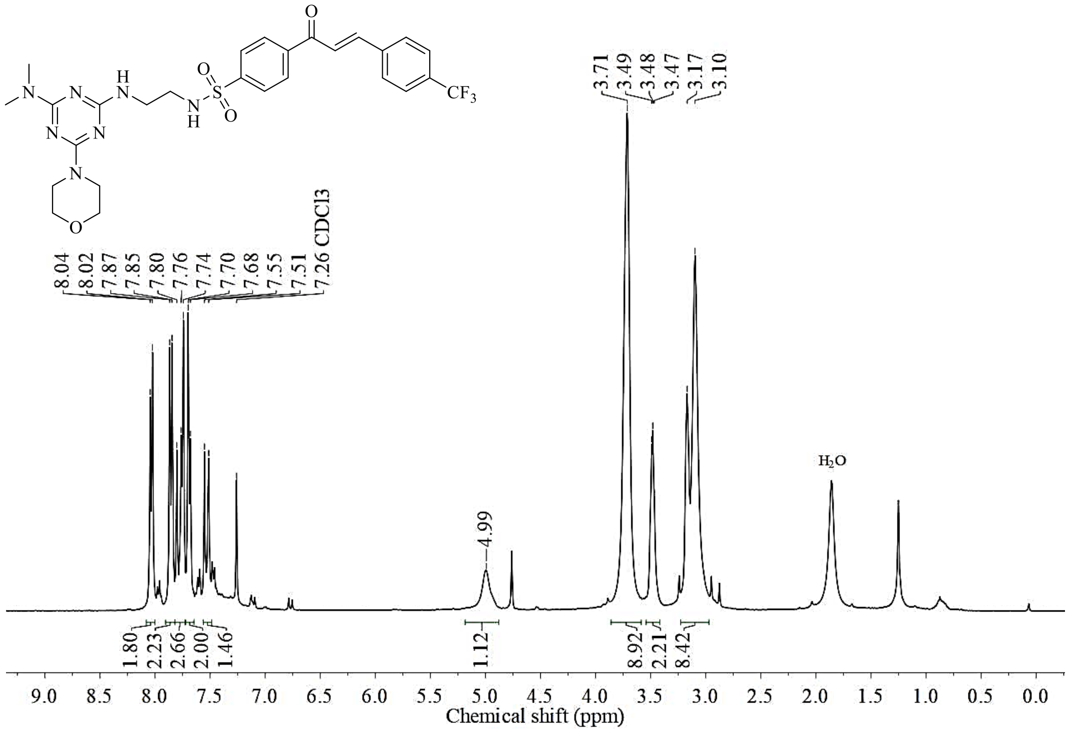

^{1}H NMR (400 MHz, $CDCl_3$) spectra of **23g**.

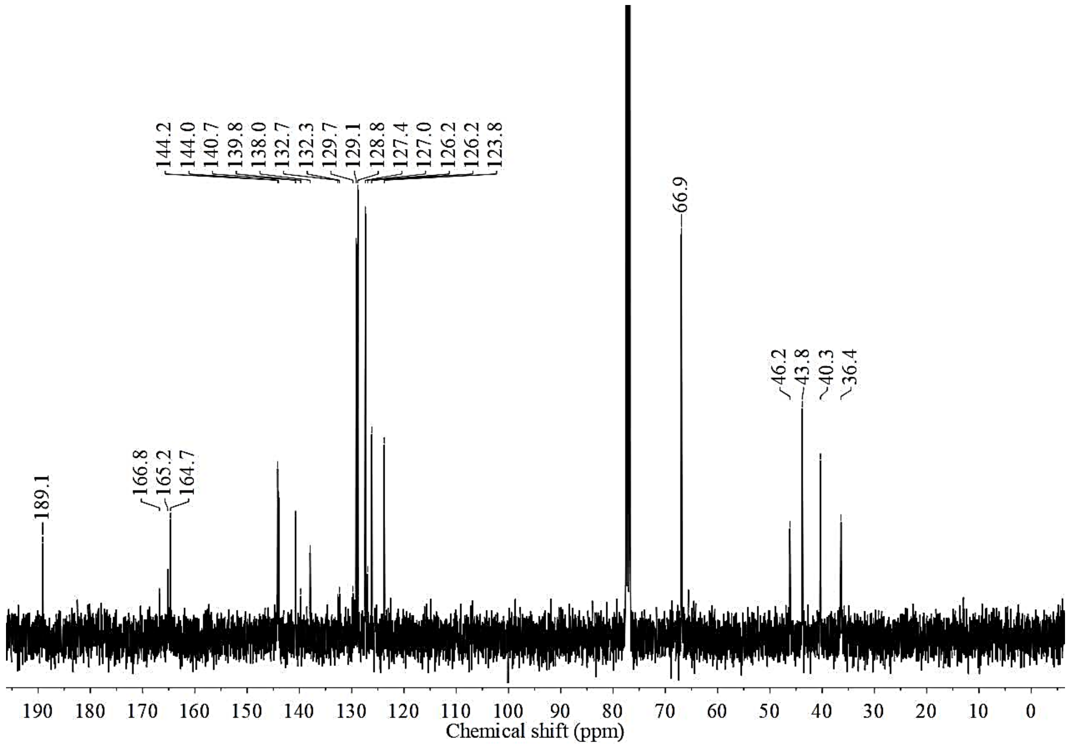

^{13}C NMR (100 MHz, $CDCl_3$) spectra of **23g**.

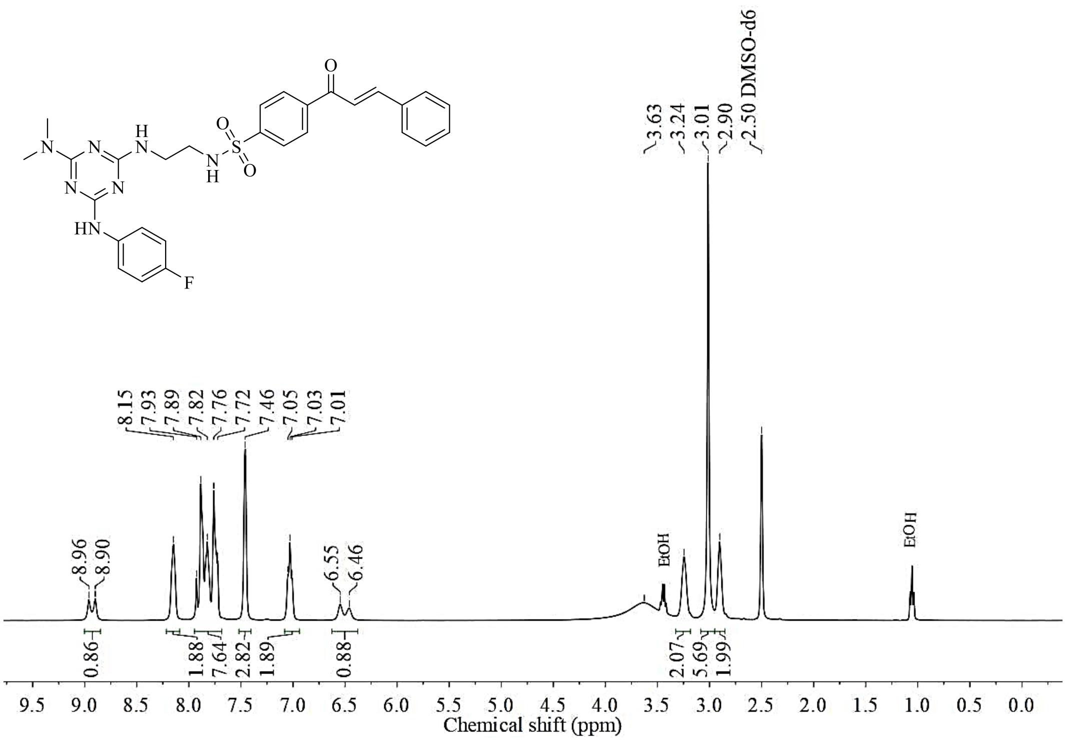

^{1}H NMR (400 MHz, DMSO-d_6) spectra of **24a**.

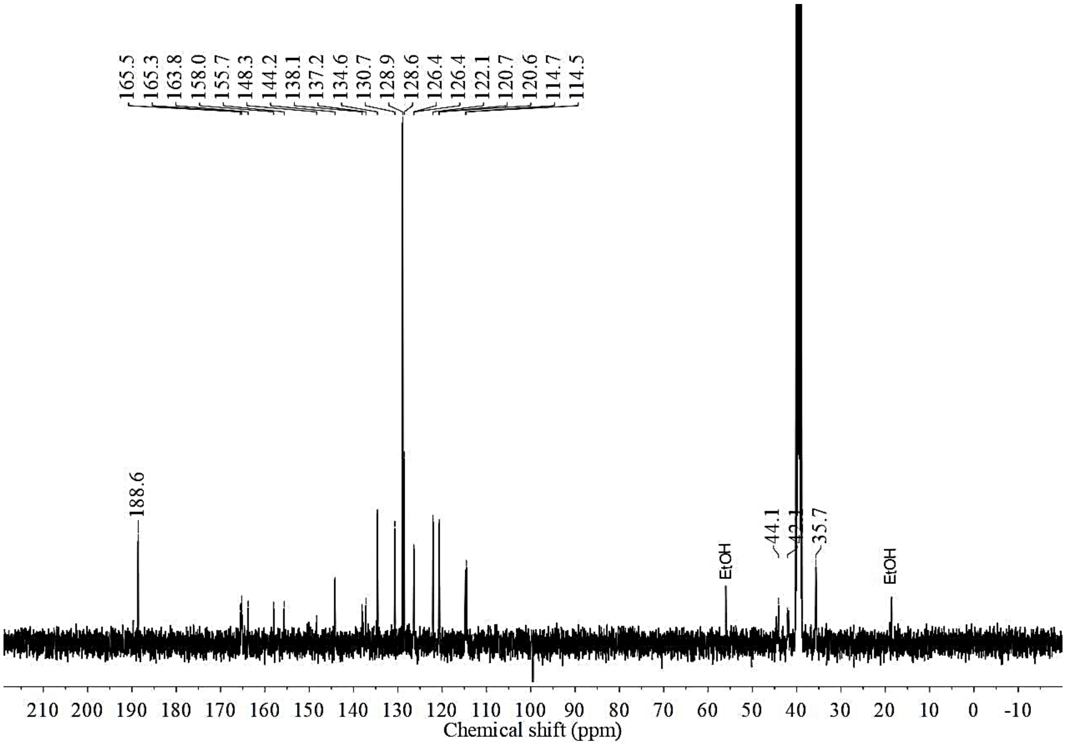

^{13}C NMR (100 MHz, DMSO-d_6) spectra of **24a**.

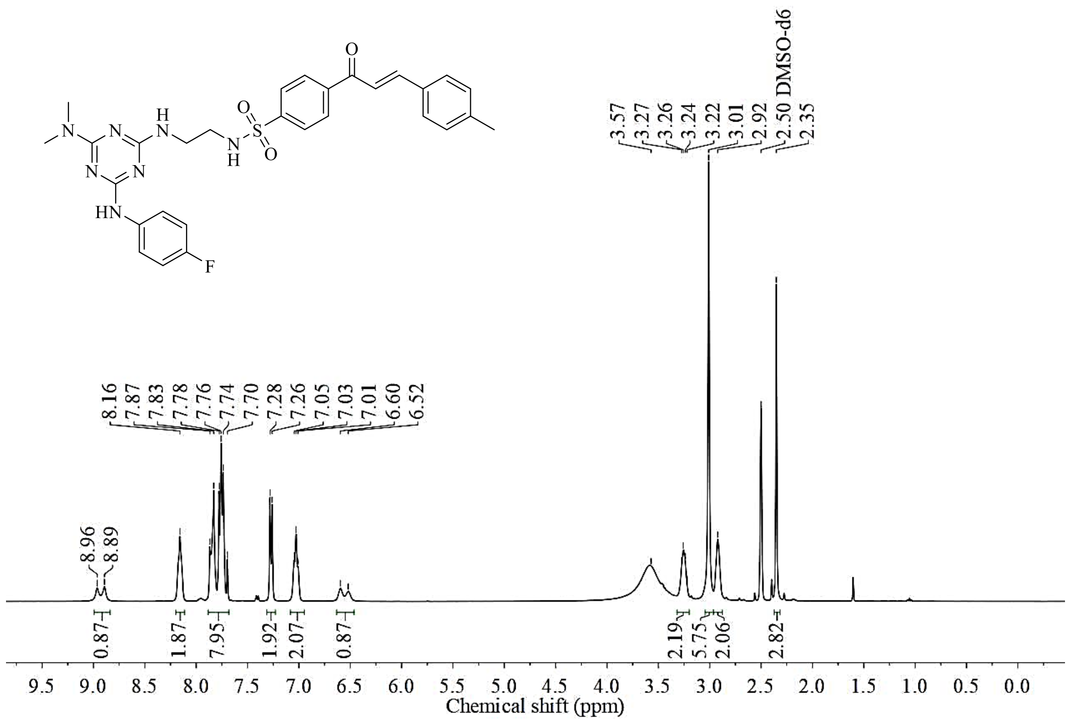

^{1}H NMR (400 MHz, DMSO-d_6) spectra of **24b**.

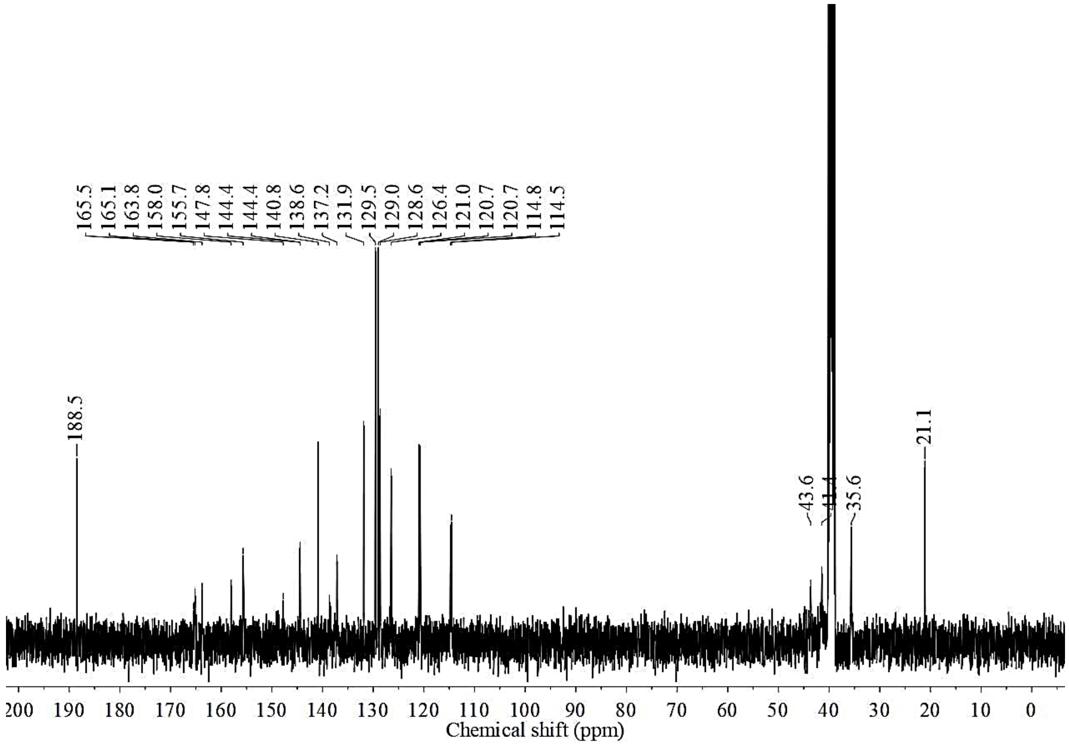

^{13}C NMR (100 MHz, DMSO-d_6) spectra of **24b**.

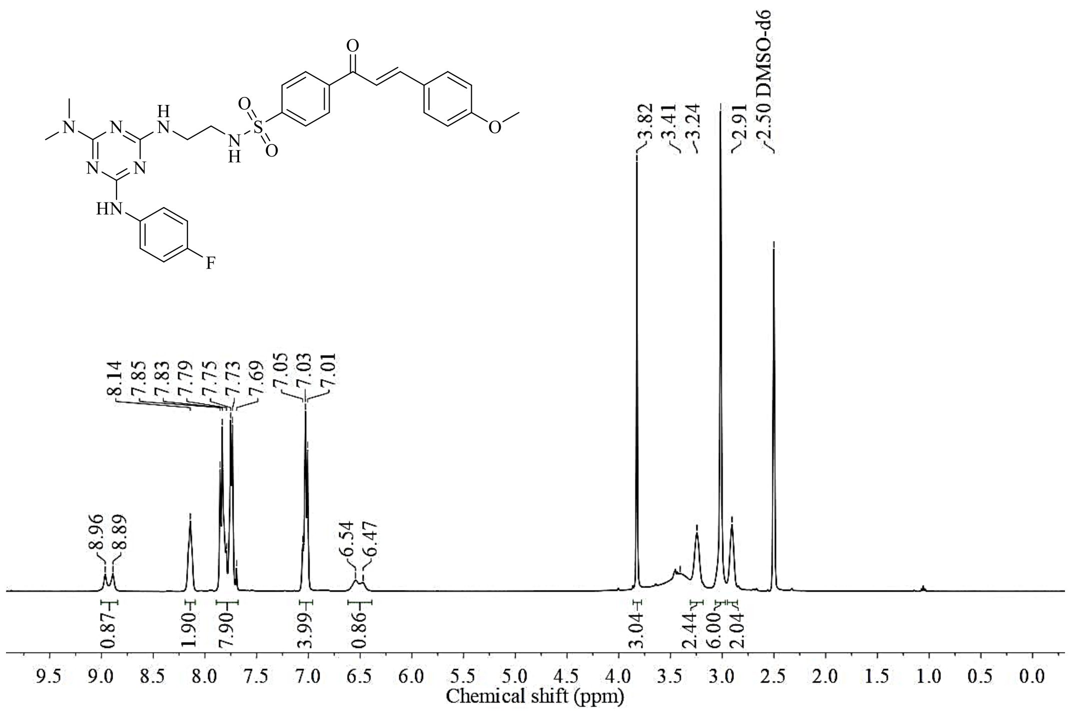

^{1}H NMR (400 MHz, DMSO-d_6) spectra of **24c**.

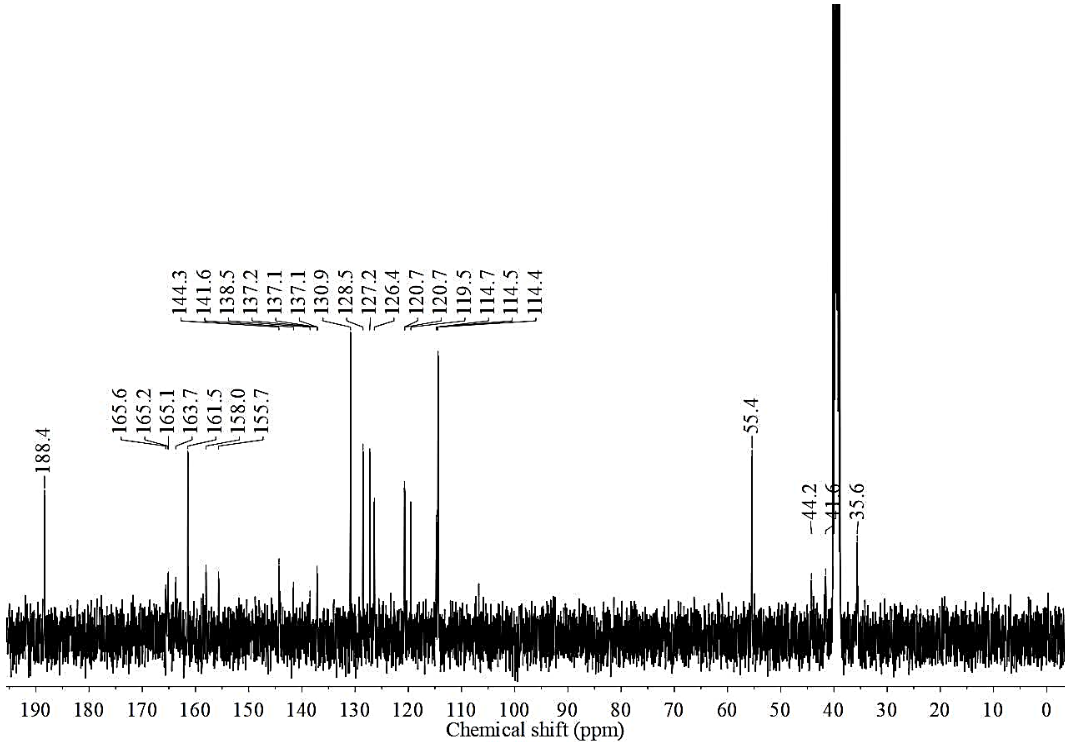

^{13}C NMR (100 MHz, DMSO-d_6) spectra of **24c**.

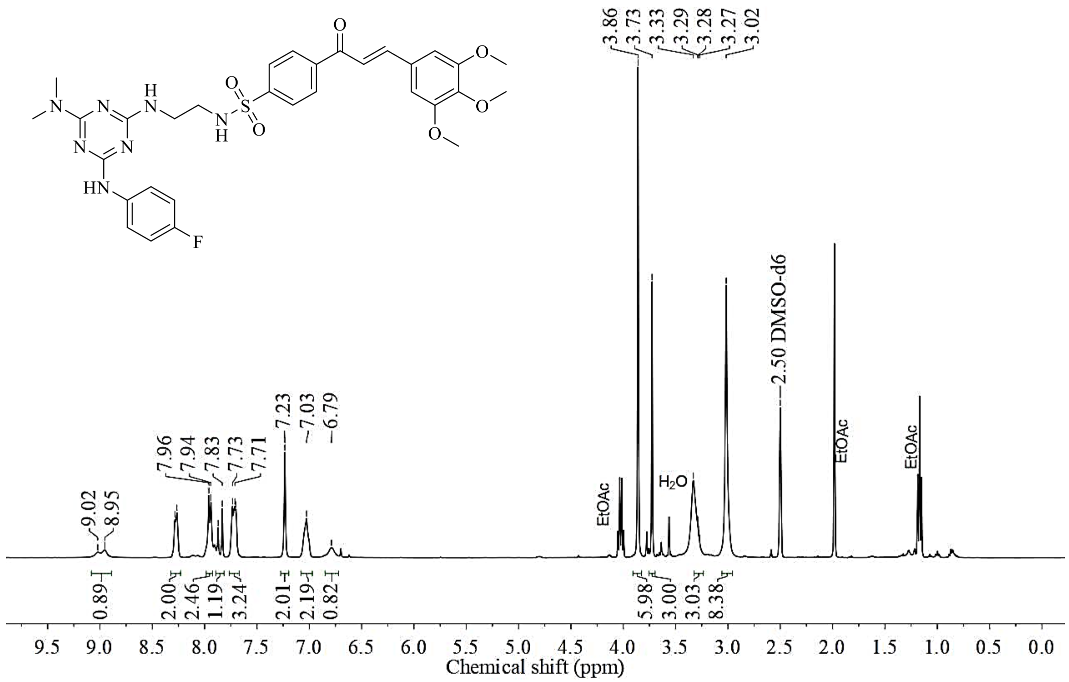

^{1}H NMR (400 MHz, DMSO-d_6) spectra of **24d**.

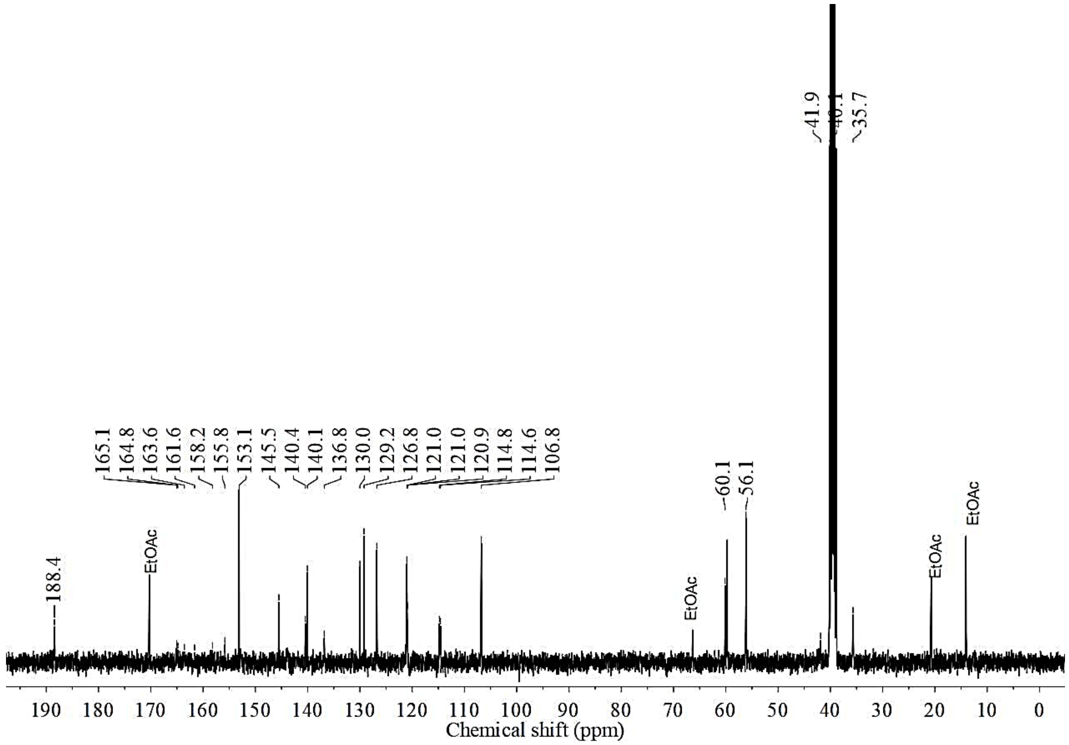

^{13}C NMR (100 MHz, DMSO-d_6) spectra of **24d**.

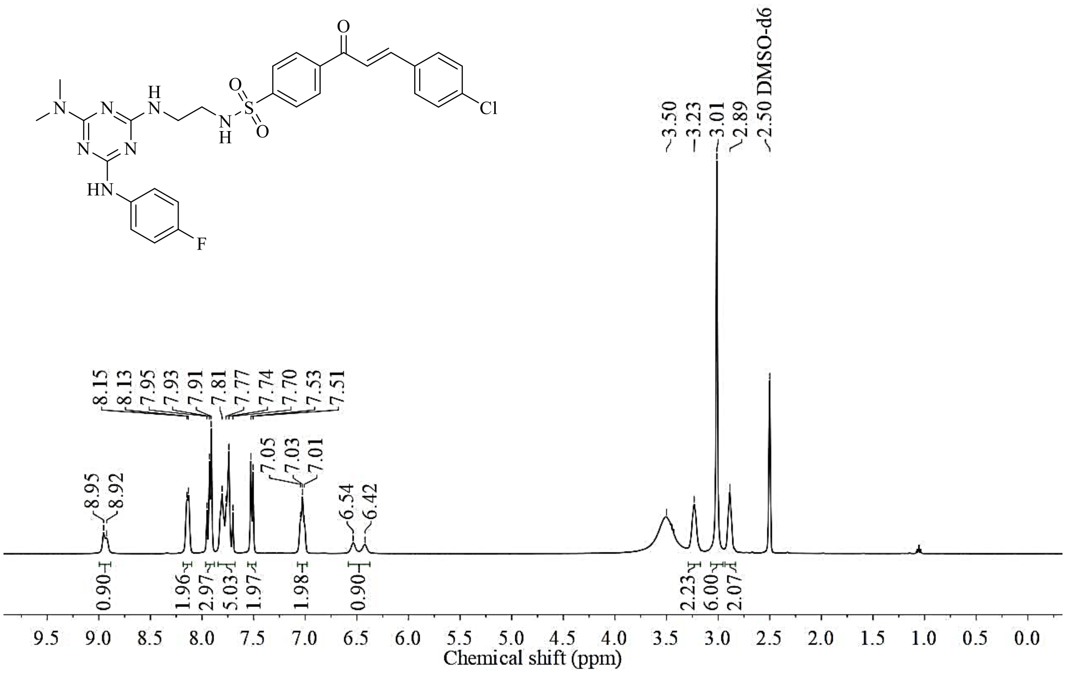

^{1}H NMR (400 MHz, DMSO-d_6) spectra of **24e**.

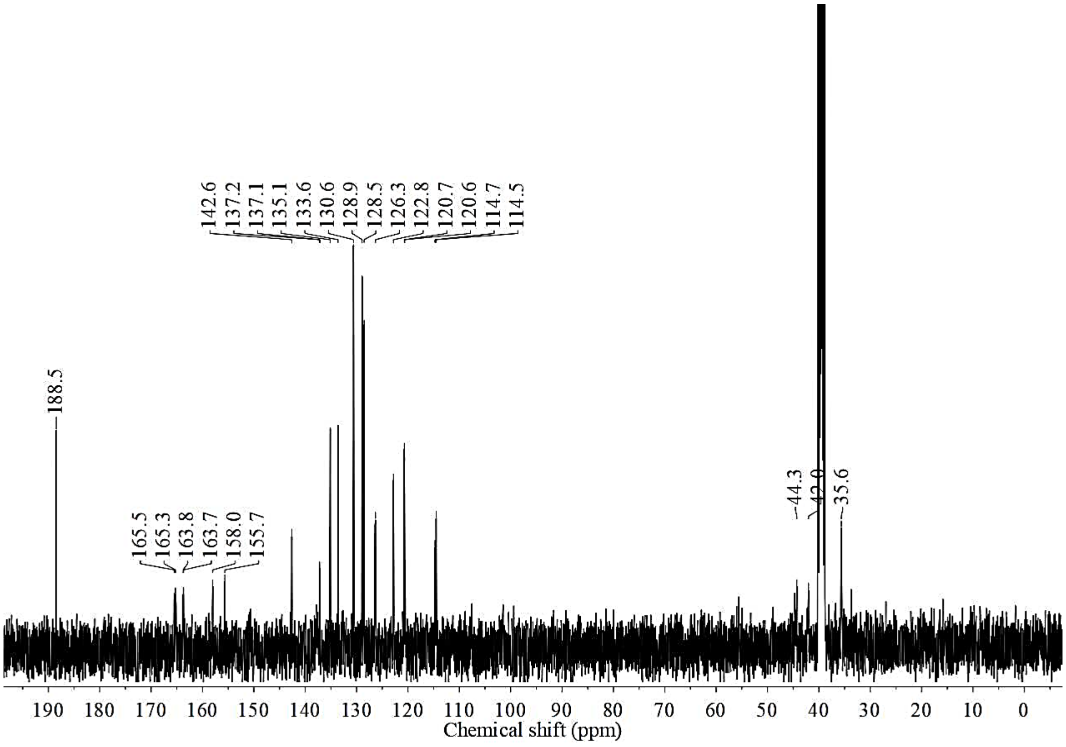

^{13}C NMR (100 MHz, DMSO-d_6) spectra of **24e**.

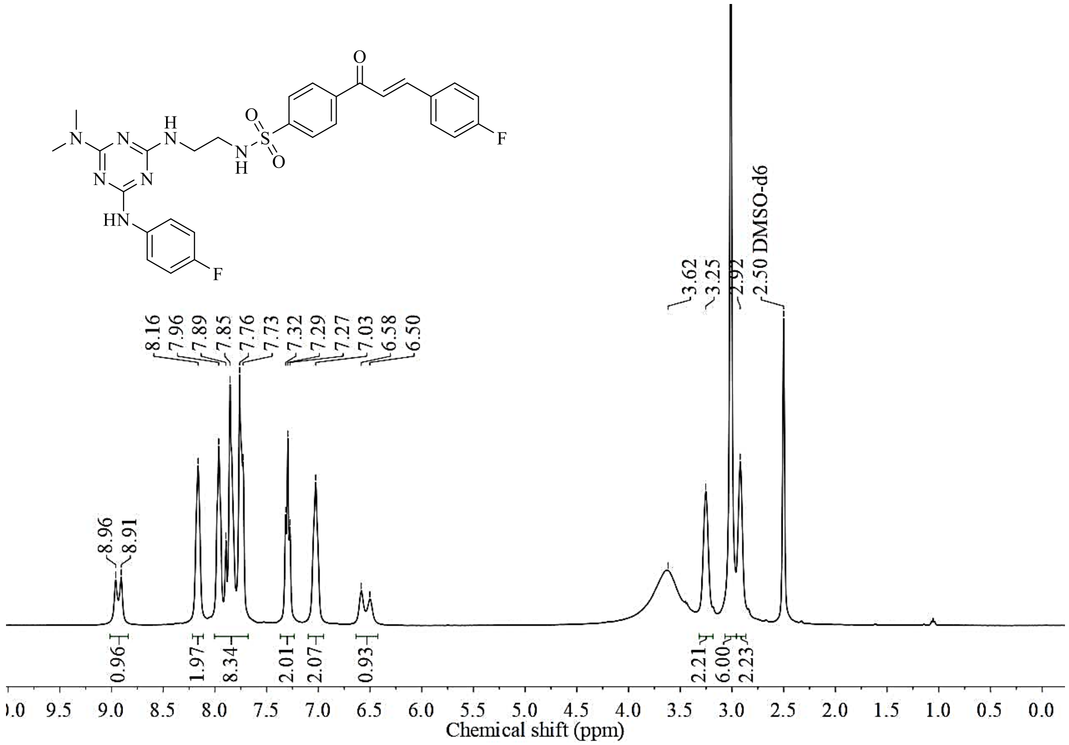

^{1}H NMR (400 MHz, DMSO-d_6) spectra of **24f**.

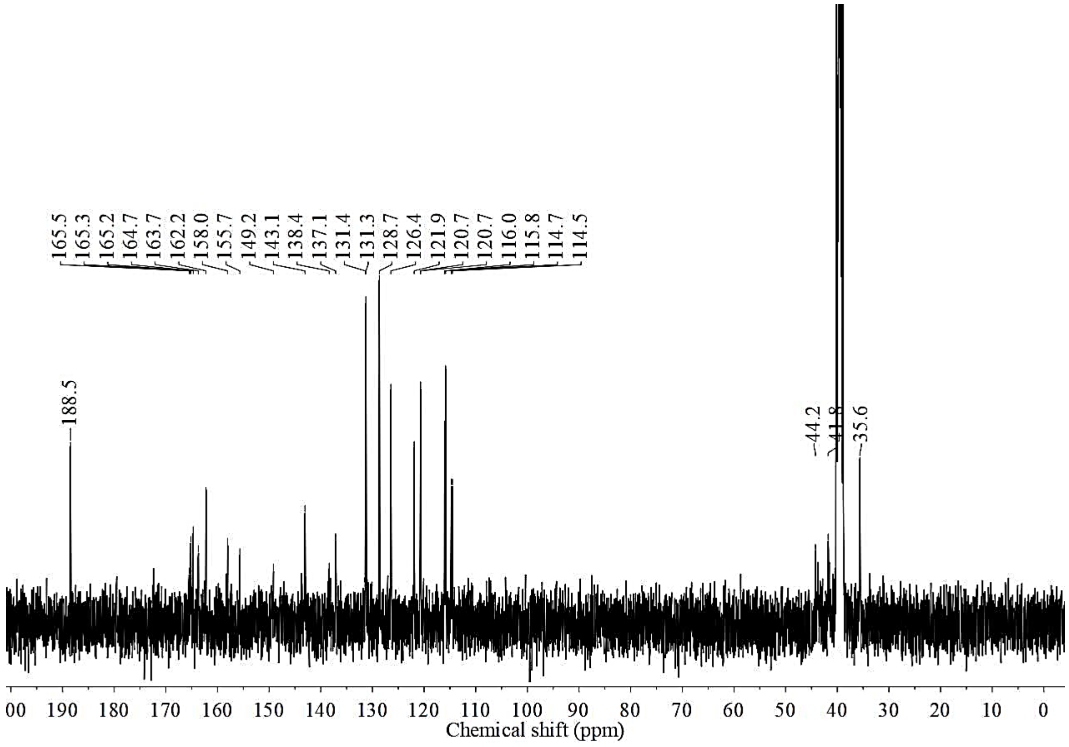

^{13}C NMR (100 MHz, DMSO-d_6) spectra of **24f**.

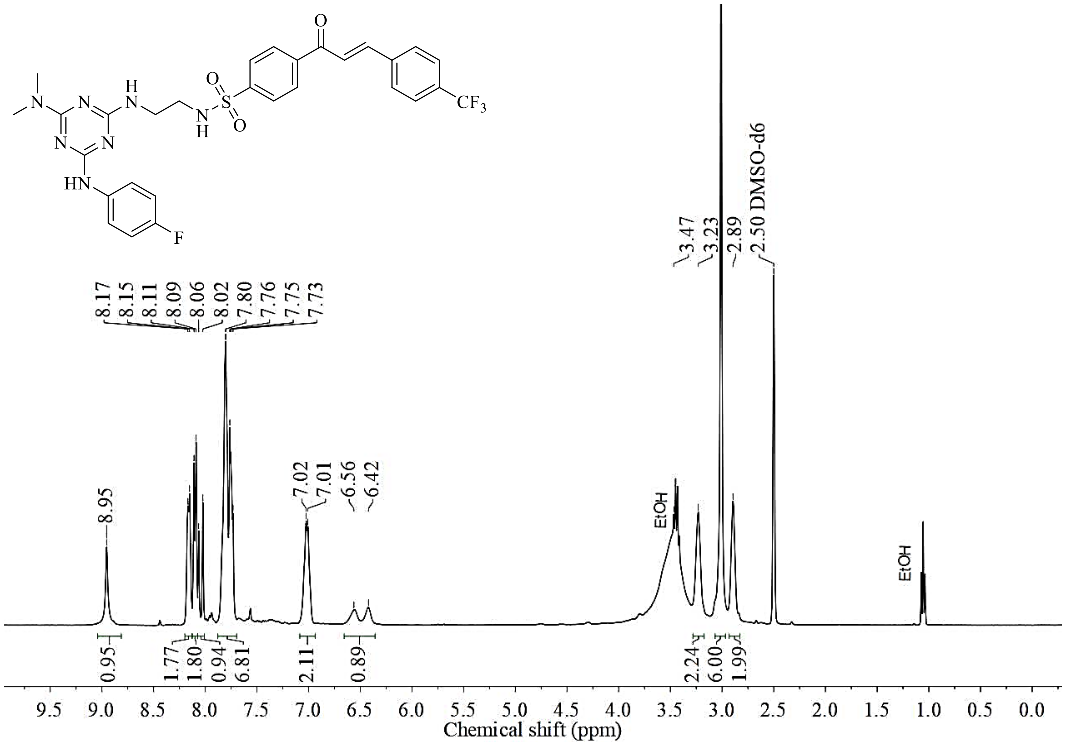

^{1}H NMR (400 MHz, DMSO-d_6) spectra of **24g**.

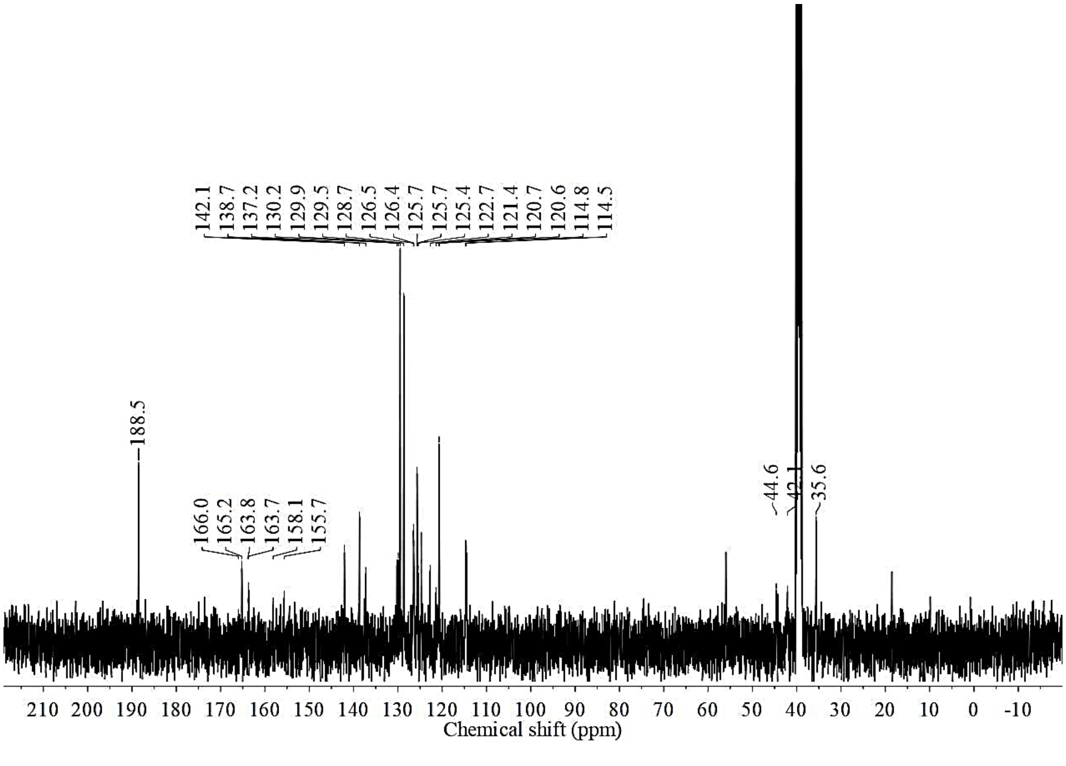

^{13}C NMR (100 MHz, DMSO-d_6) spectra of **24g**.

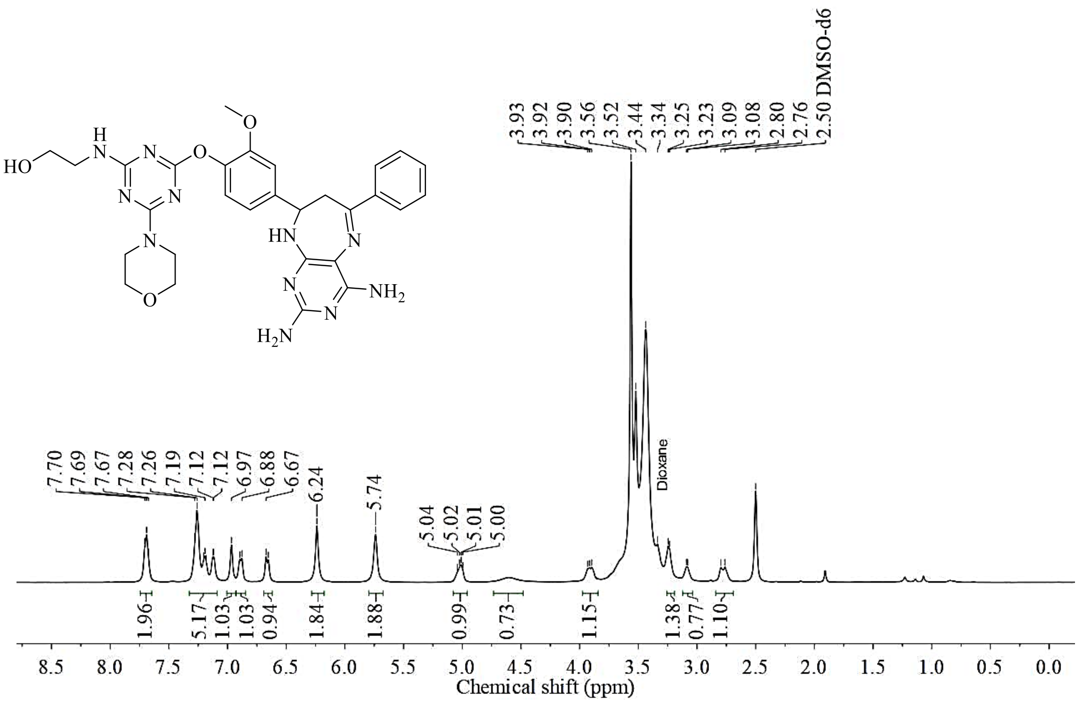

^{1}H NMR (400 MHz, DMSO-d_6) spectra of **28a**.

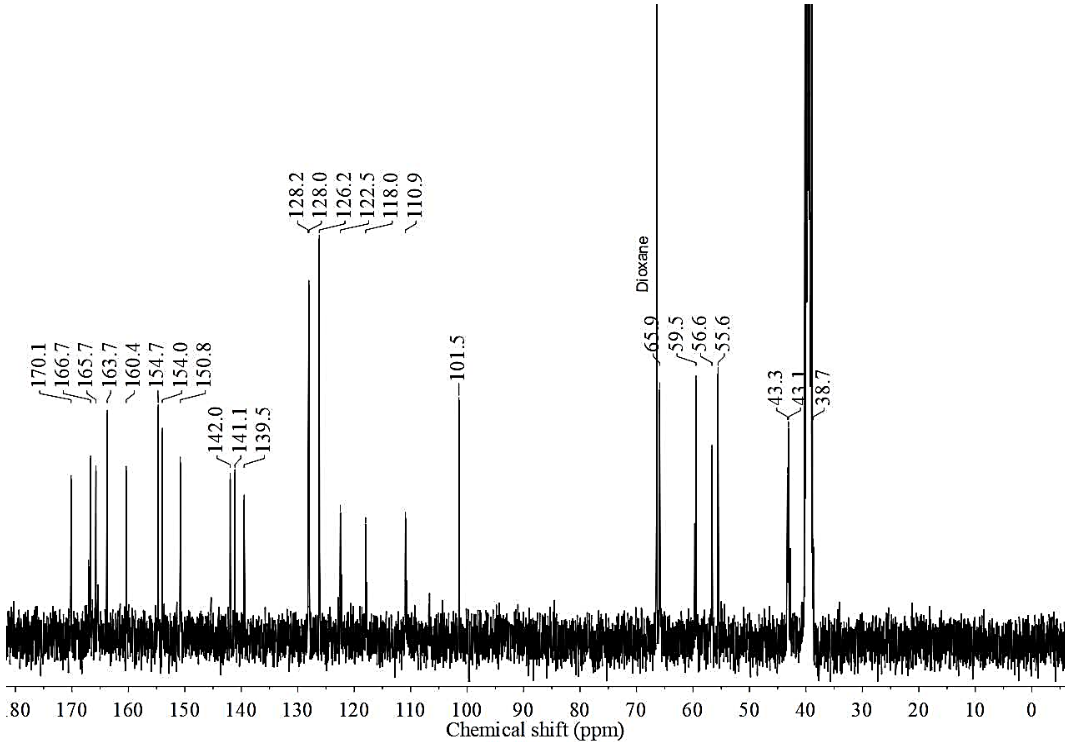

^{13}C NMR (100 MHz, DMSO-d_6) spectra of **28a**.

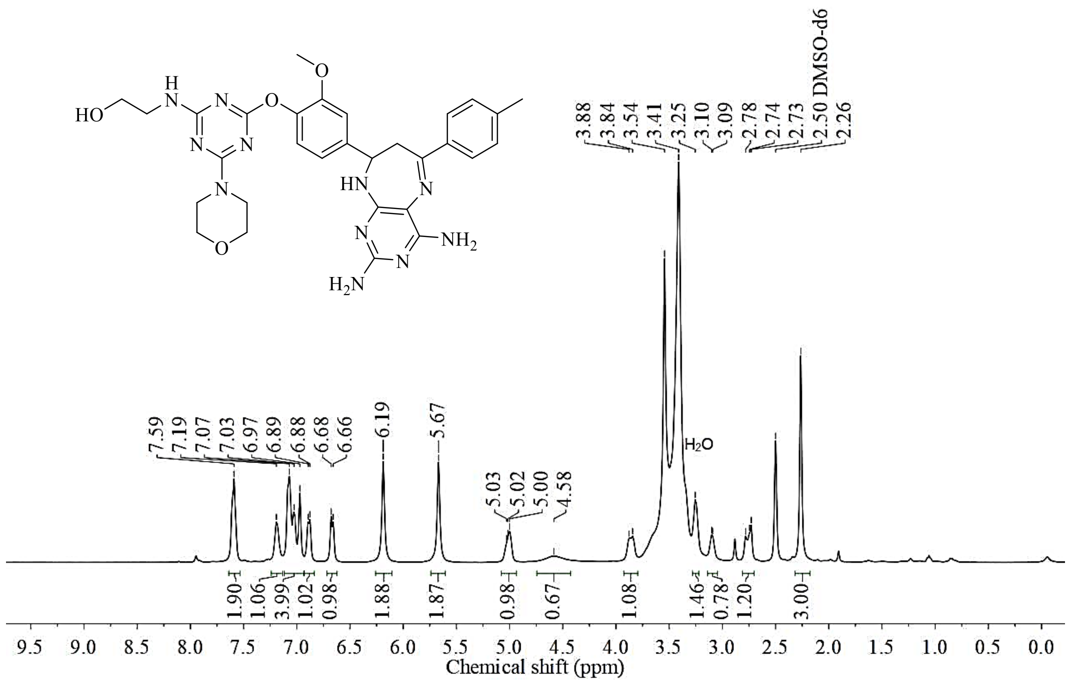

^{1}H NMR (400 MHz, DMSO-d_6) spectra of **28b**.

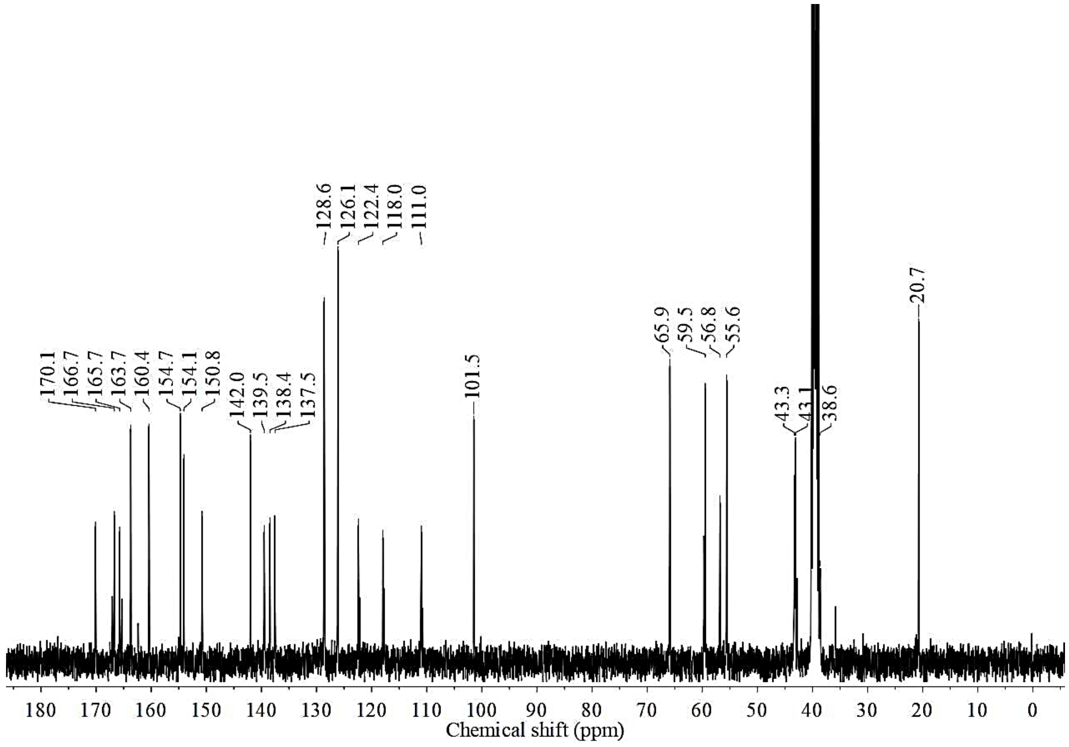

^{13}C NMR (100 MHz, DMSO-d_6) spectra of **28b**.

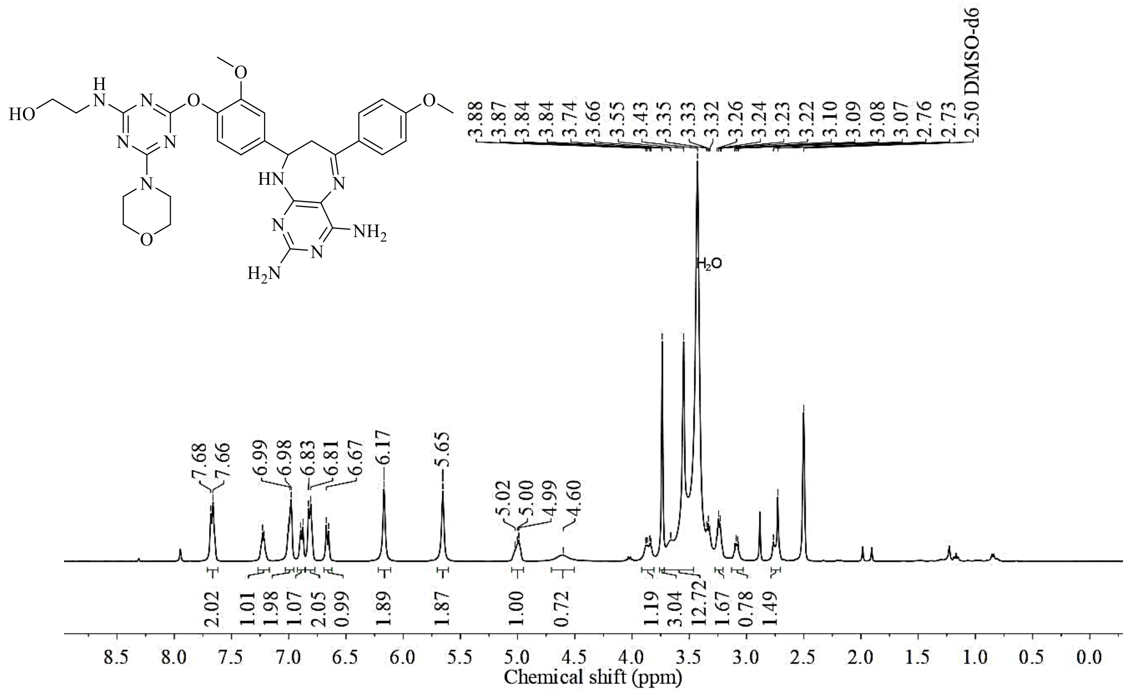

^{1}H NMR (400 MHz, DMSO-d_6) spectra of **28c**.

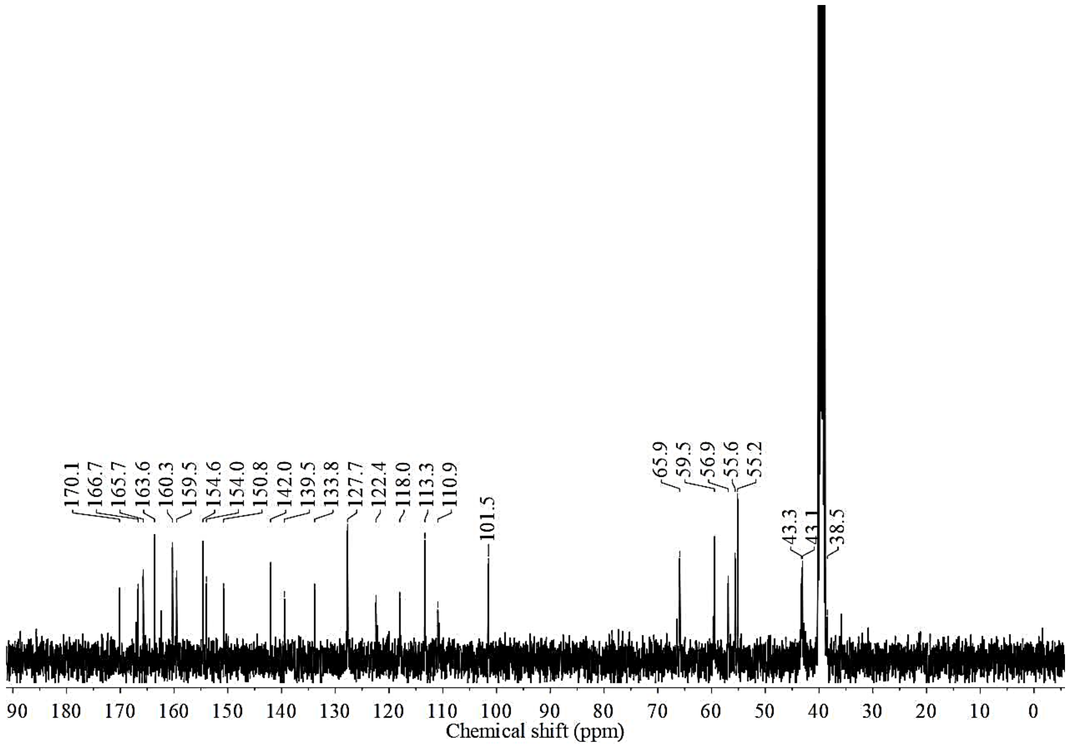

^{13}C NMR (100 MHz, DMSO-d_6) spectra of **28c**.

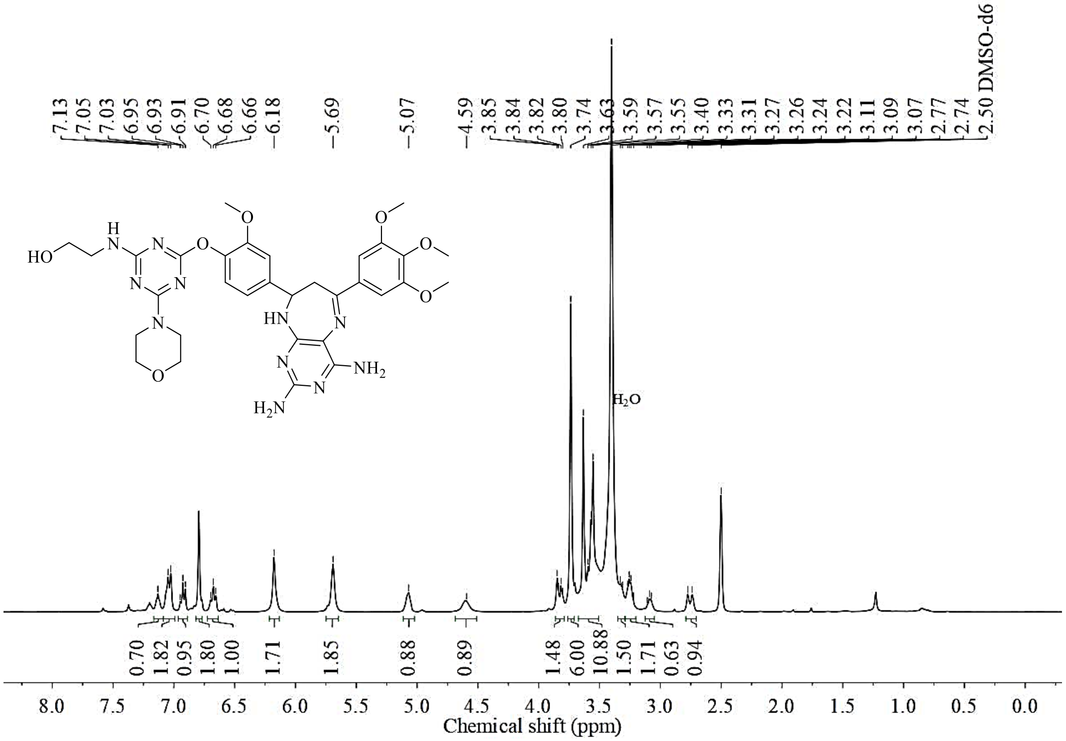

^{1}H NMR (400 MHz, DMSO-d_6) spectra of **28d**.

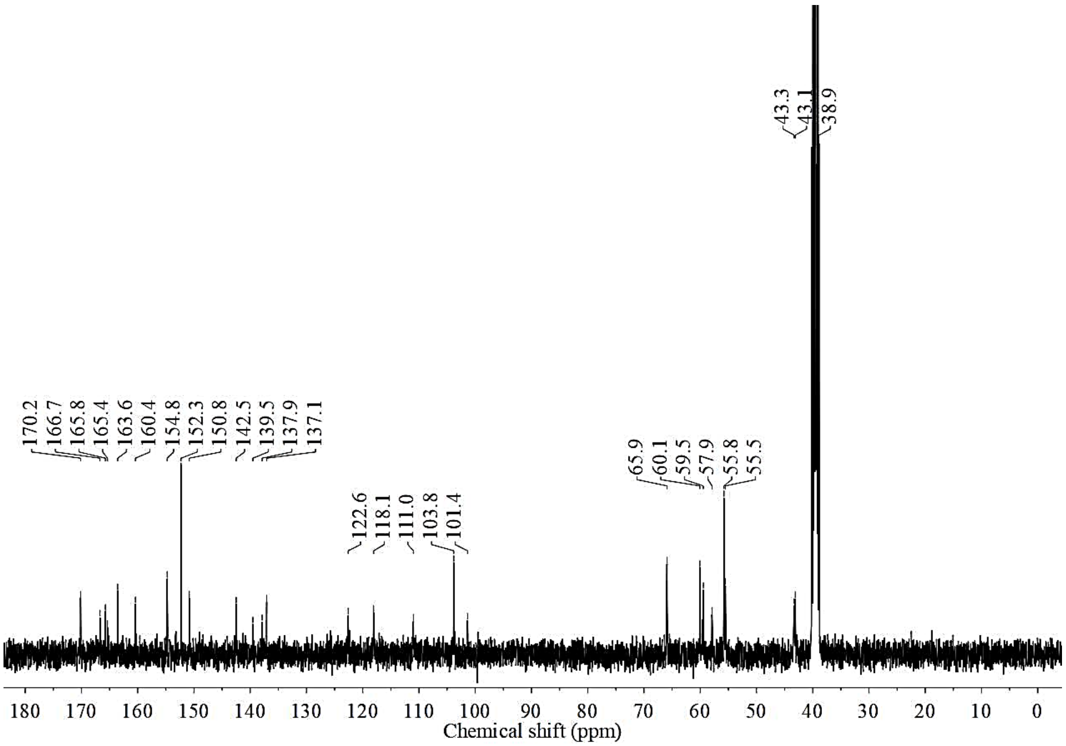

^{13}C NMR (100 MHz, DMSO-d_6) spectra of **28d**.

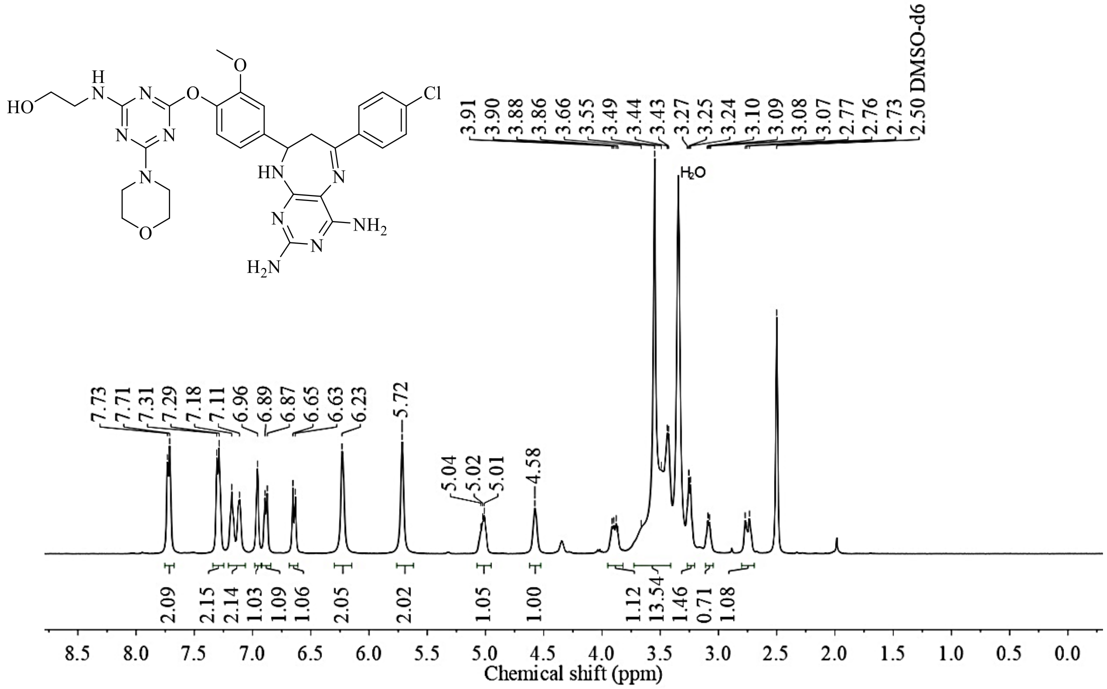

^{1}H NMR (400 MHz, DMSO-d_6) spectra of **28e**.

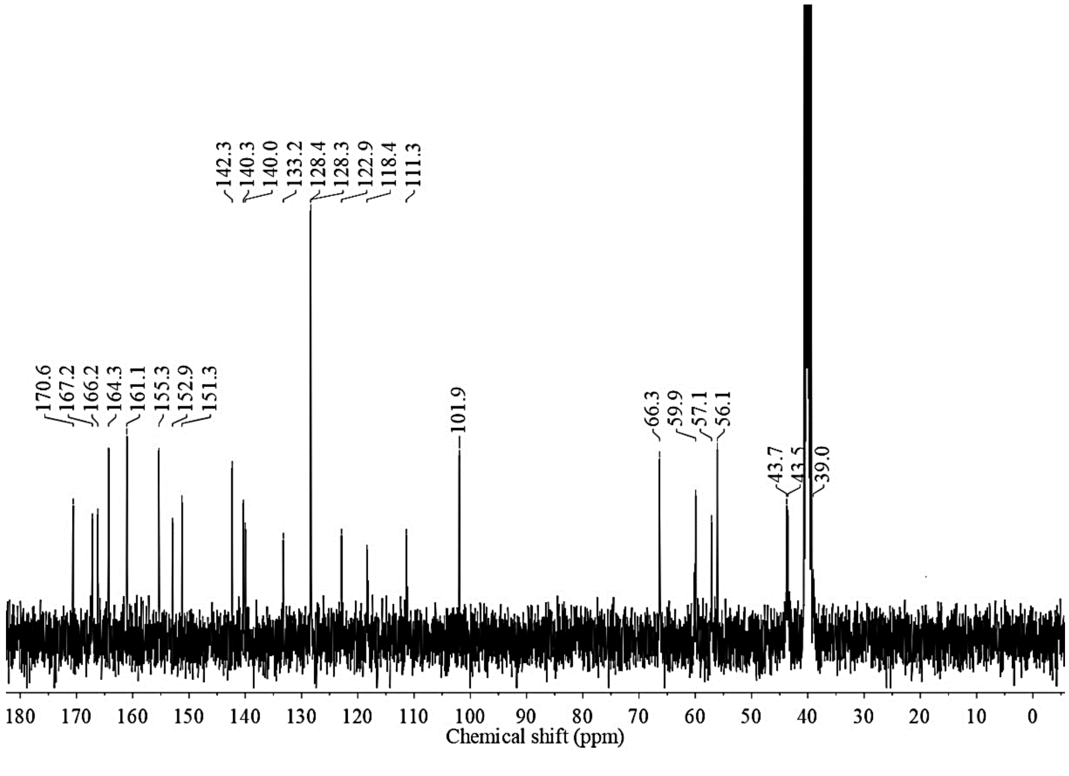

^{13}C NMR (100 MHz, DMSO-d_6) spectra of **28e**.

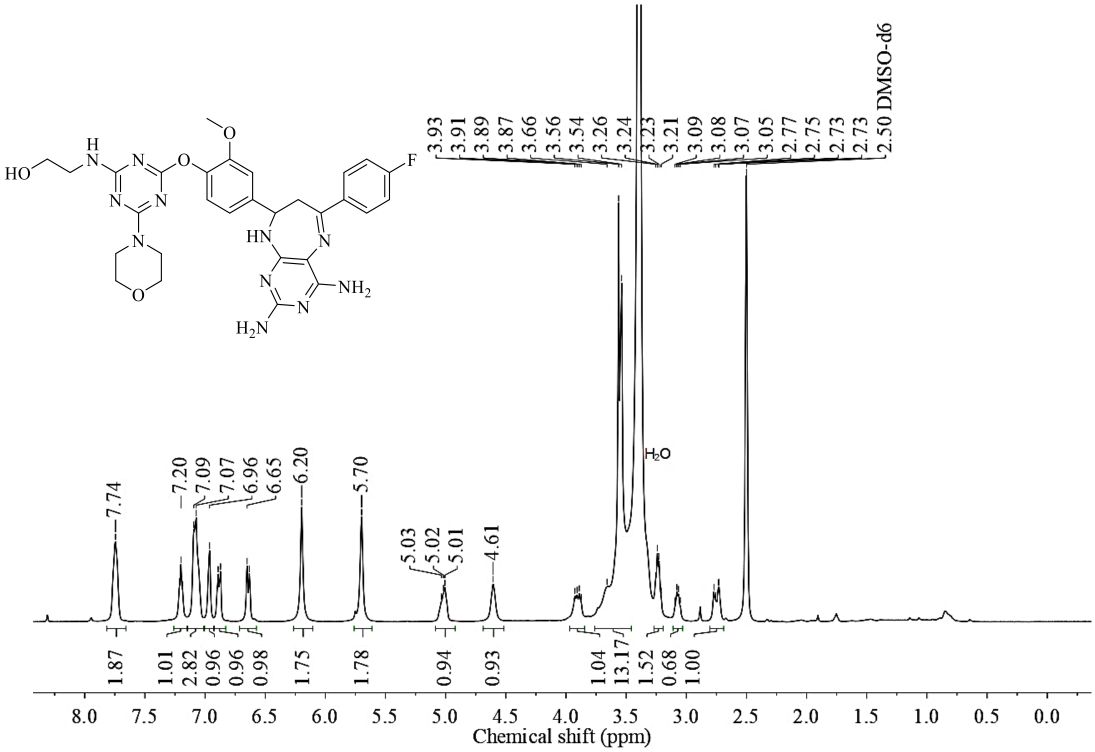

^{1}H NMR (400 MHz, DMSO-d_6) spectra of **28f**.

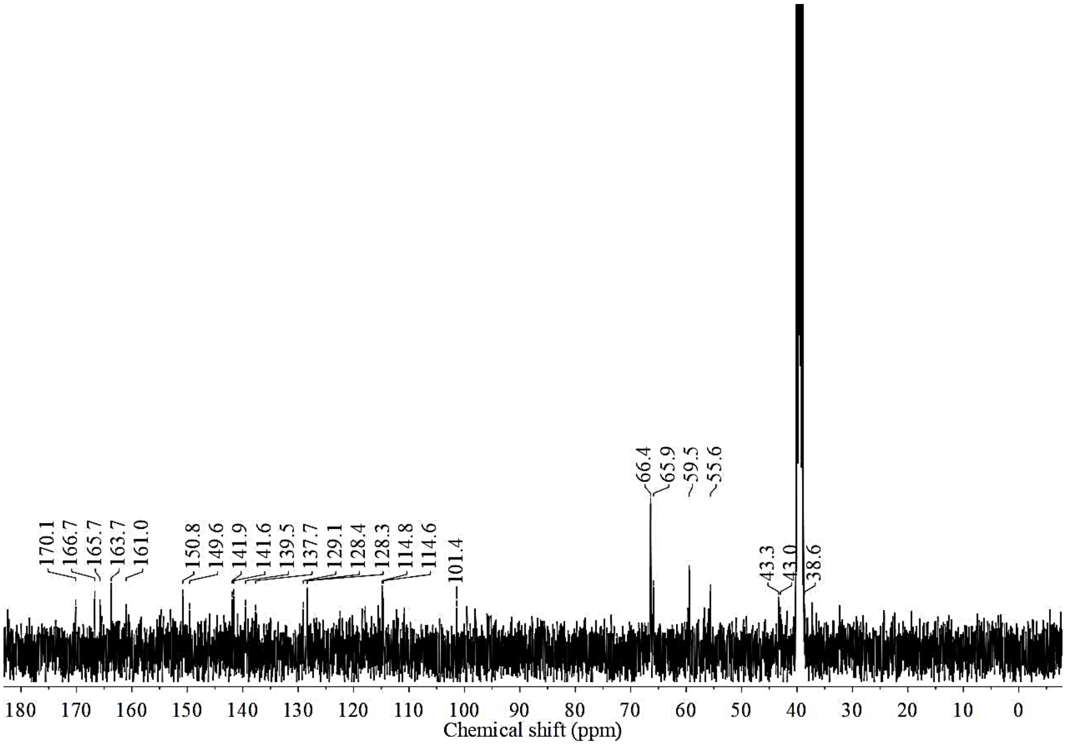

^{13}C NMR (100 MHz, DMSO-d_6) spectra of **28f**.

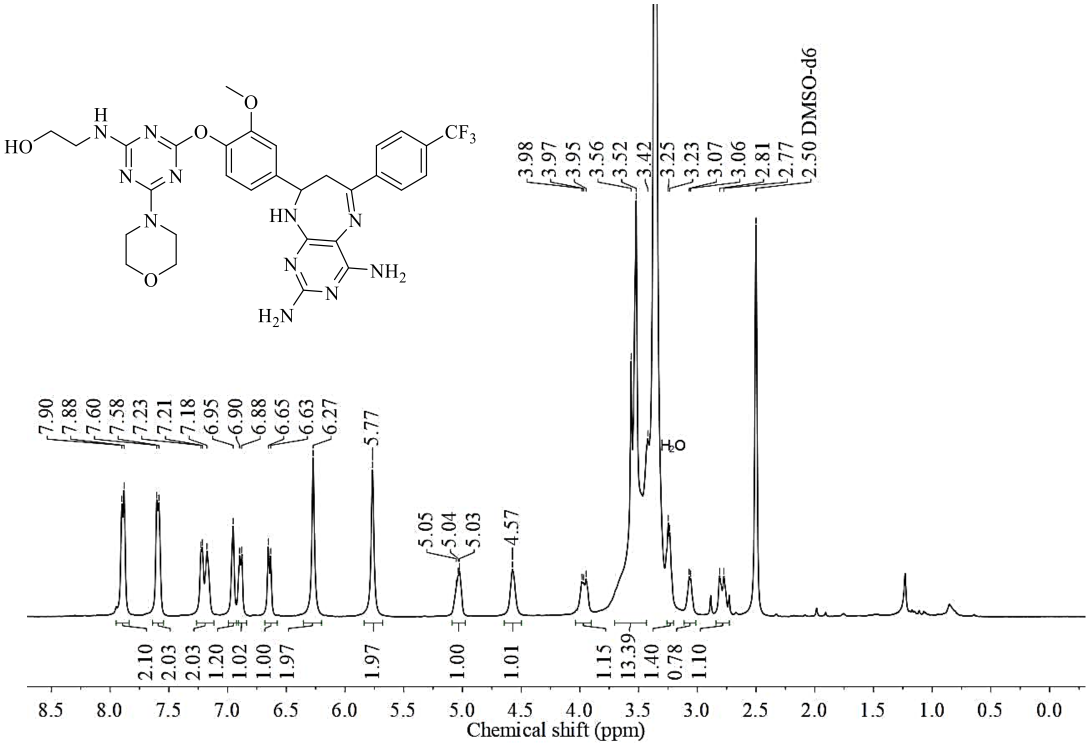

^{1}H NMR (400 MHz, DMSO-d_6) spectra of **28g**.

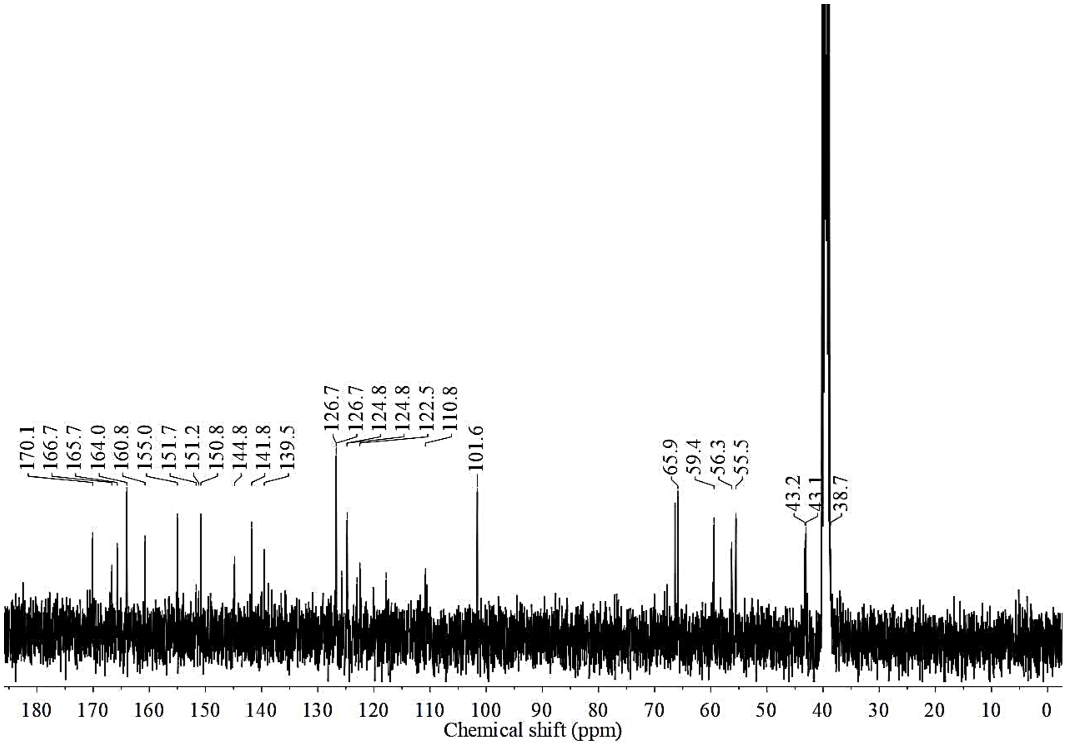

^{13}C NMR (100 MHz, DMSO-d_6) spectra of **28g**.

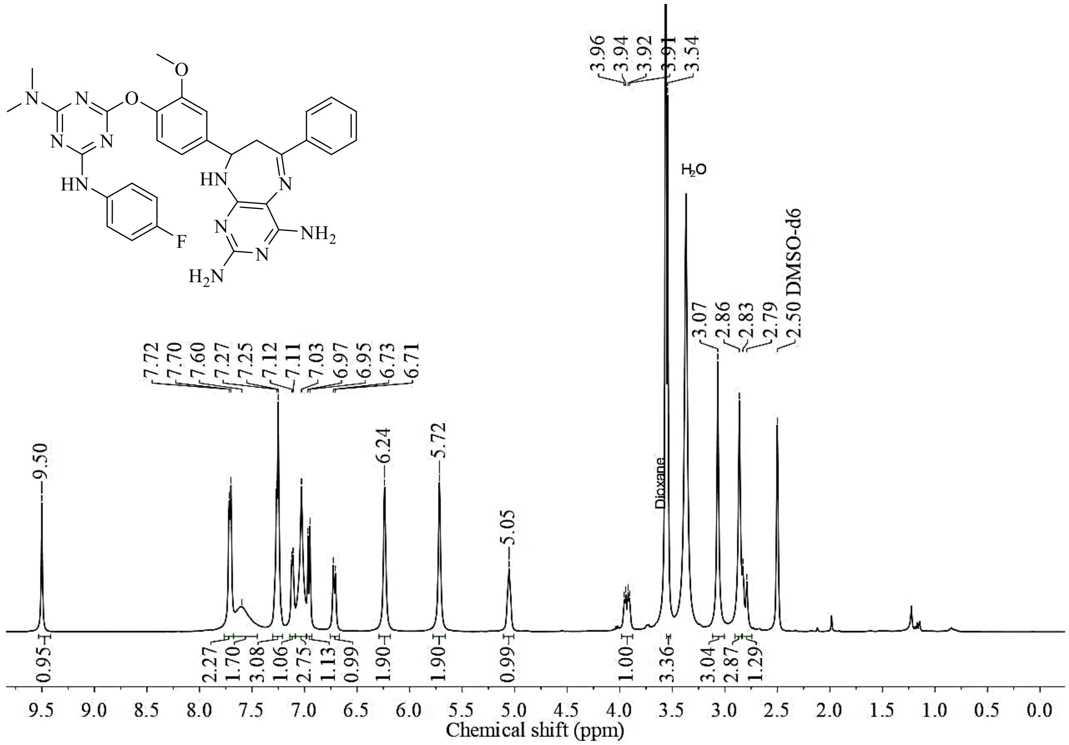

^{1}H NMR (400 MHz, DMSO-d_6) spectra of **29a**.

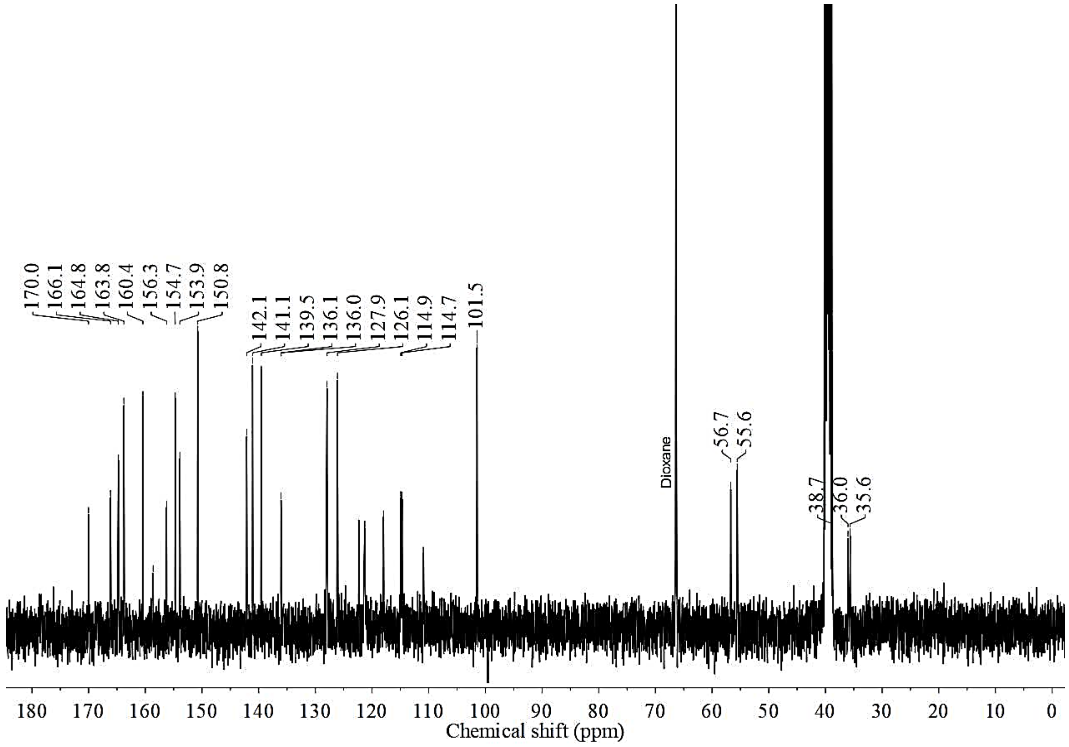

^{13}C NMR (100 MHz, DMSO-d_6) spectra of **29a**.

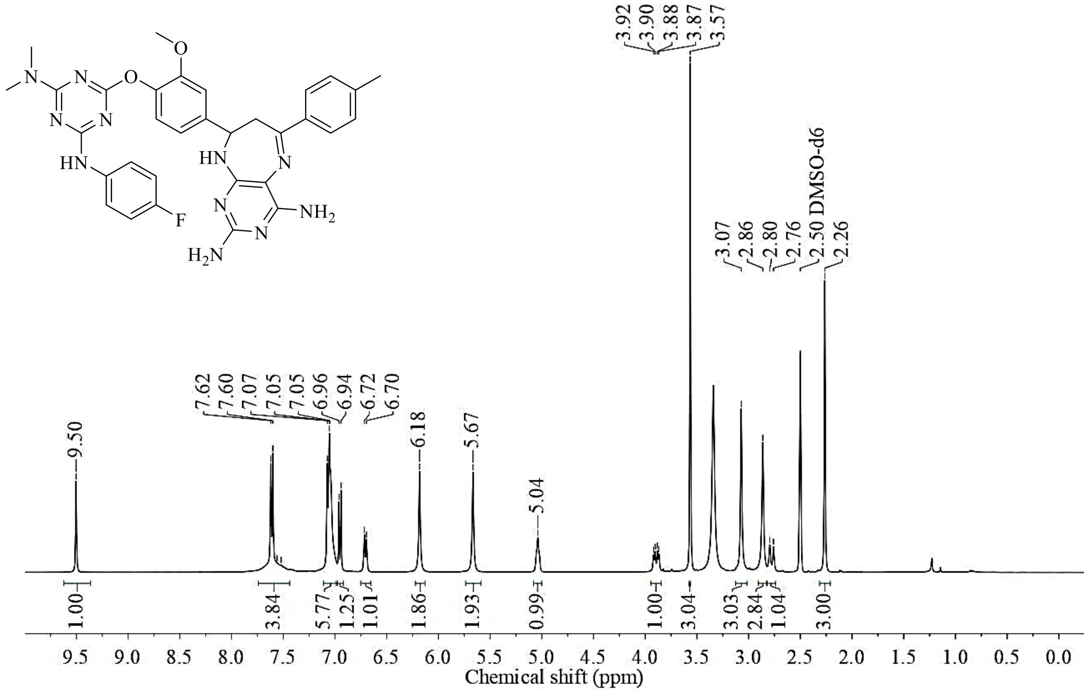

^{1}H NMR (400 MHz, DMSO-d_6) spectra of **29b**.

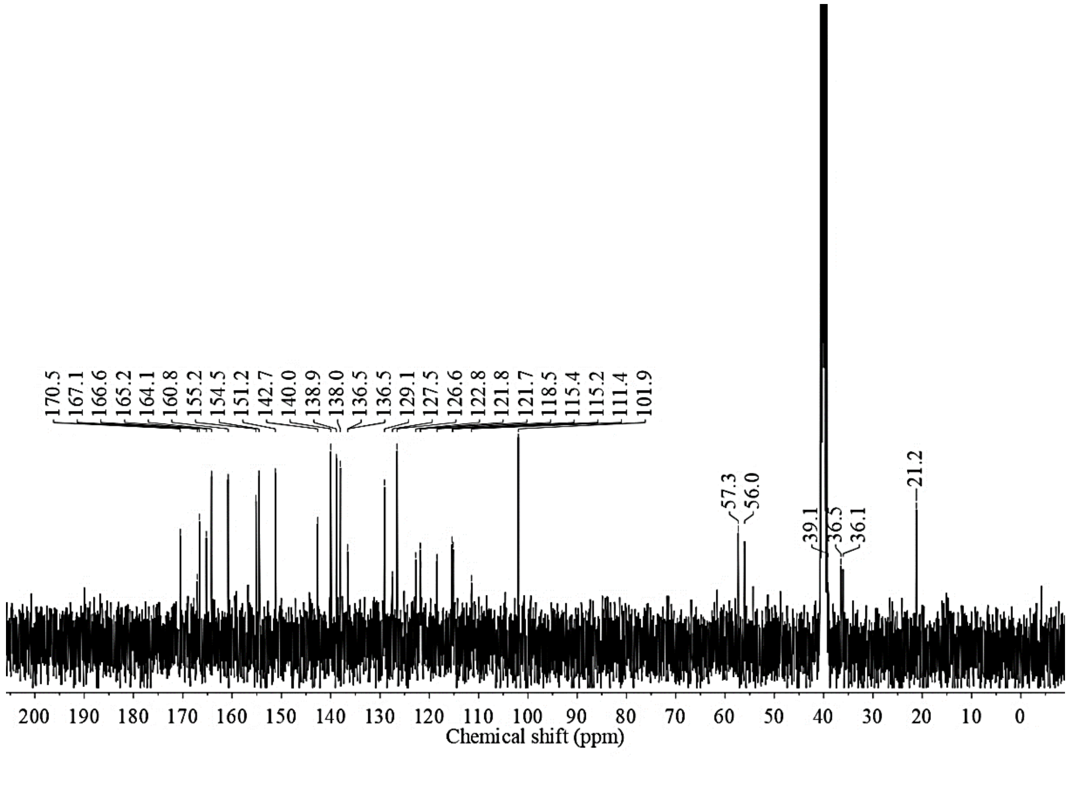

^{13}C NMR (100 MHz, DMSO-d_6) spectra of **29b**.

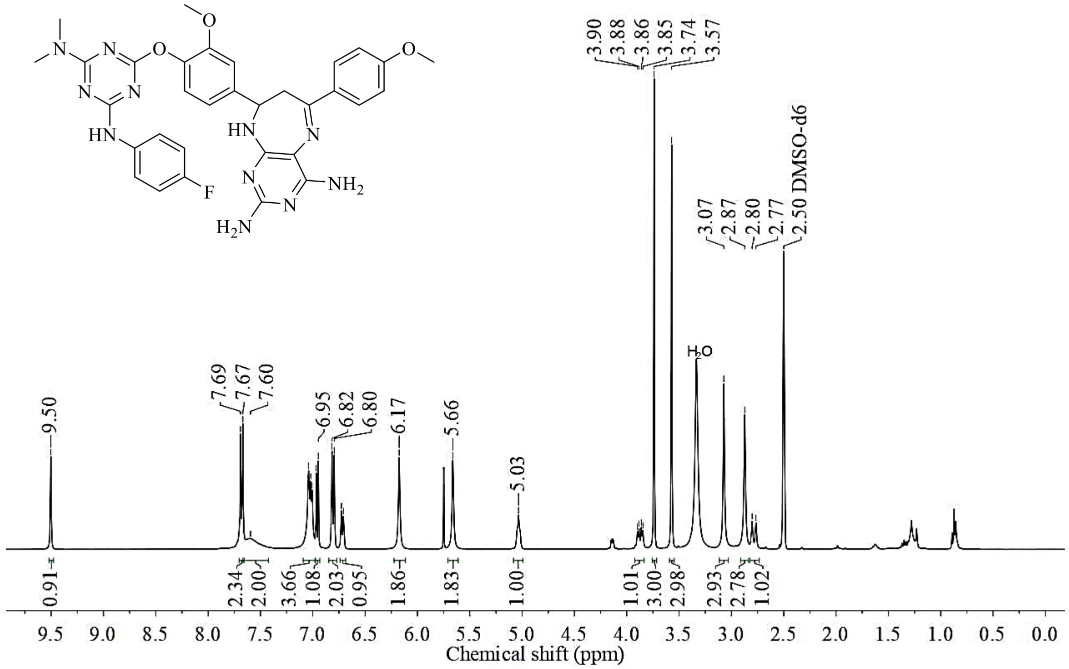

^{1}H NMR (400 MHz, DMSO-d_6) spectra of **29c**.

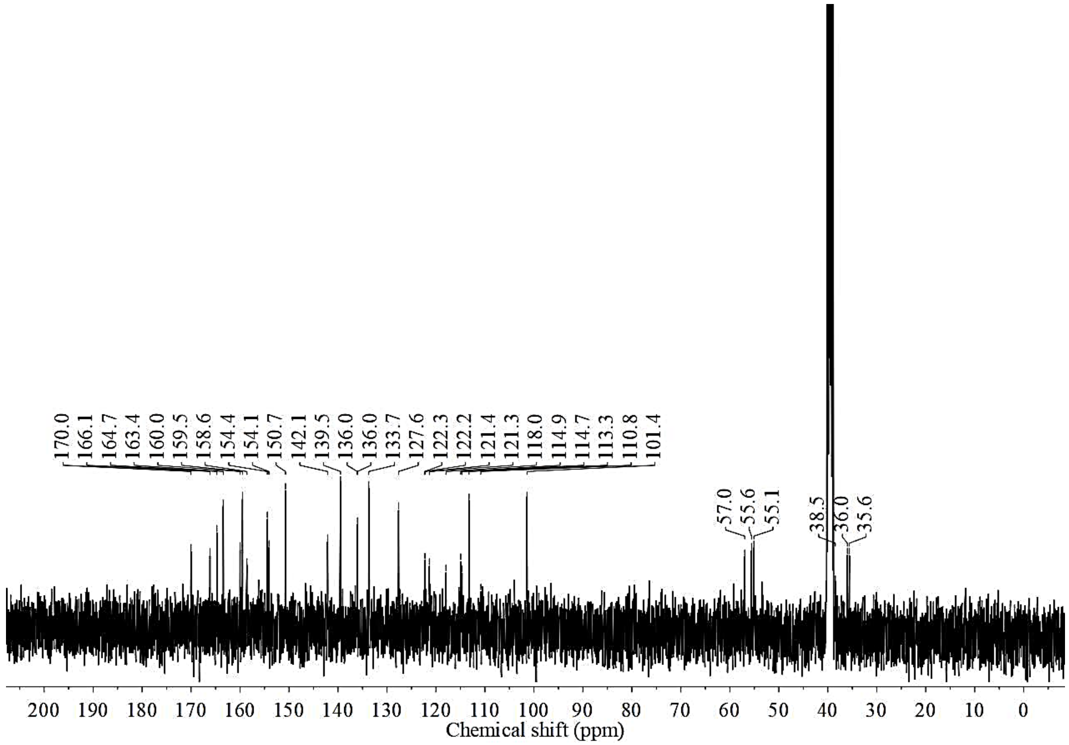

^{13}C NMR (100 MHz, DMSO-d_6) spectra of **29c**.

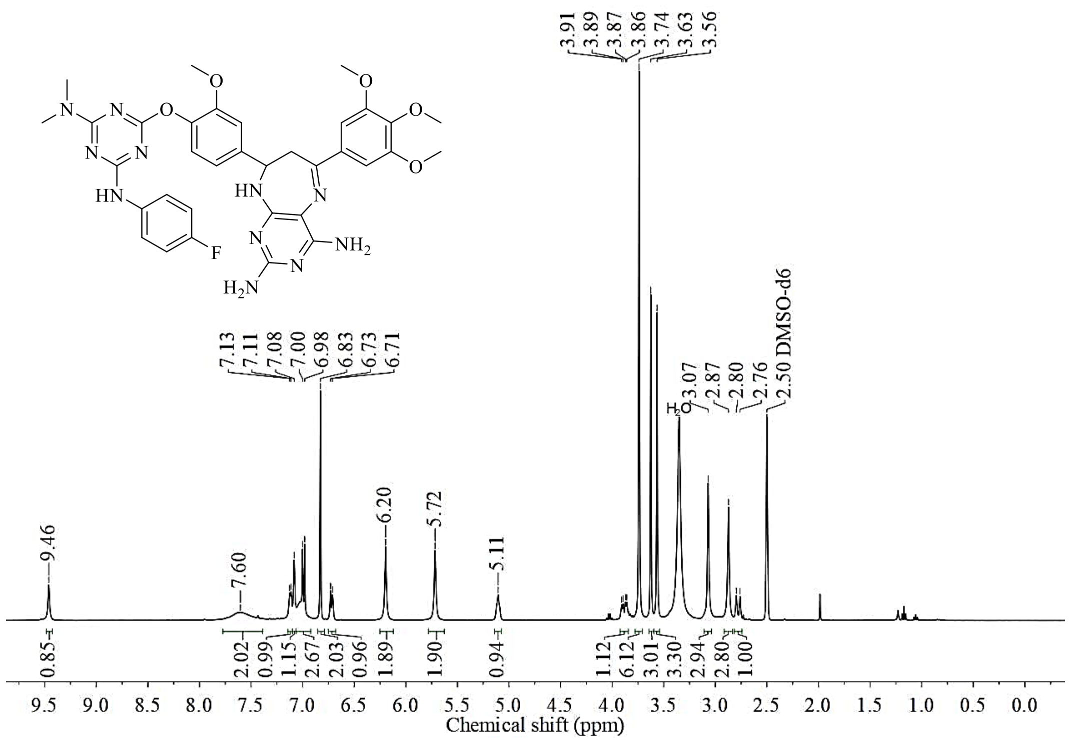

^{1}H NMR (400 MHz, DMSO-d_6) spectra of **29d**.

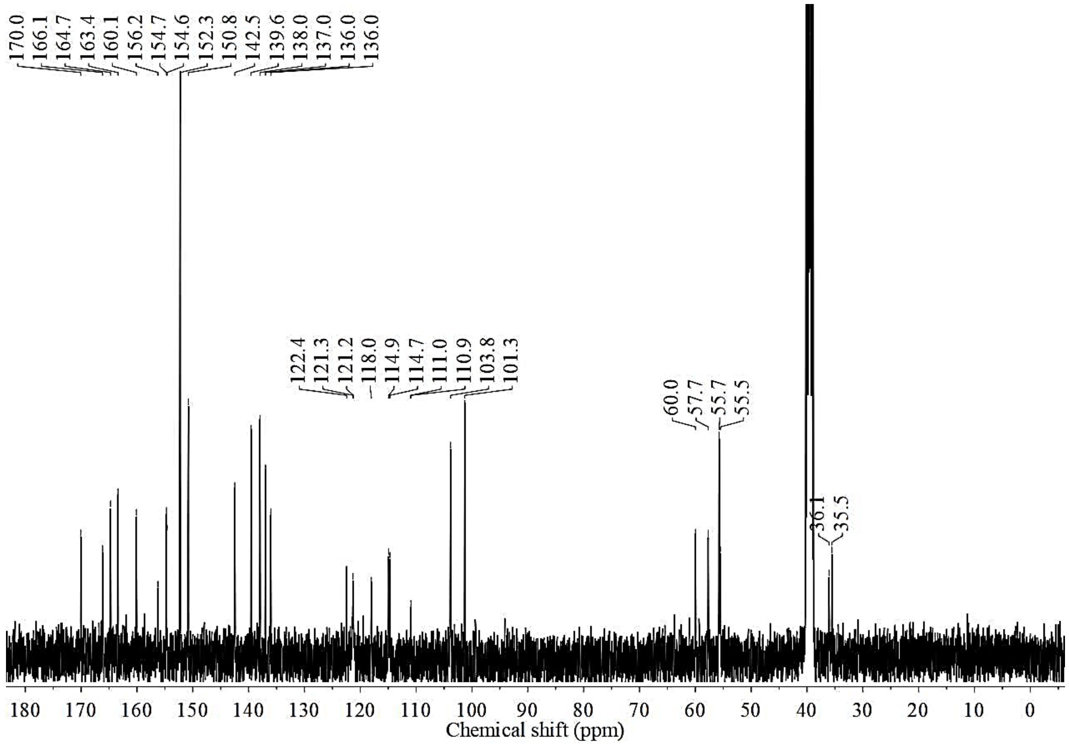

^{13}C NMR (100 MHz, DMSO-d_6) spectra of **29d**.

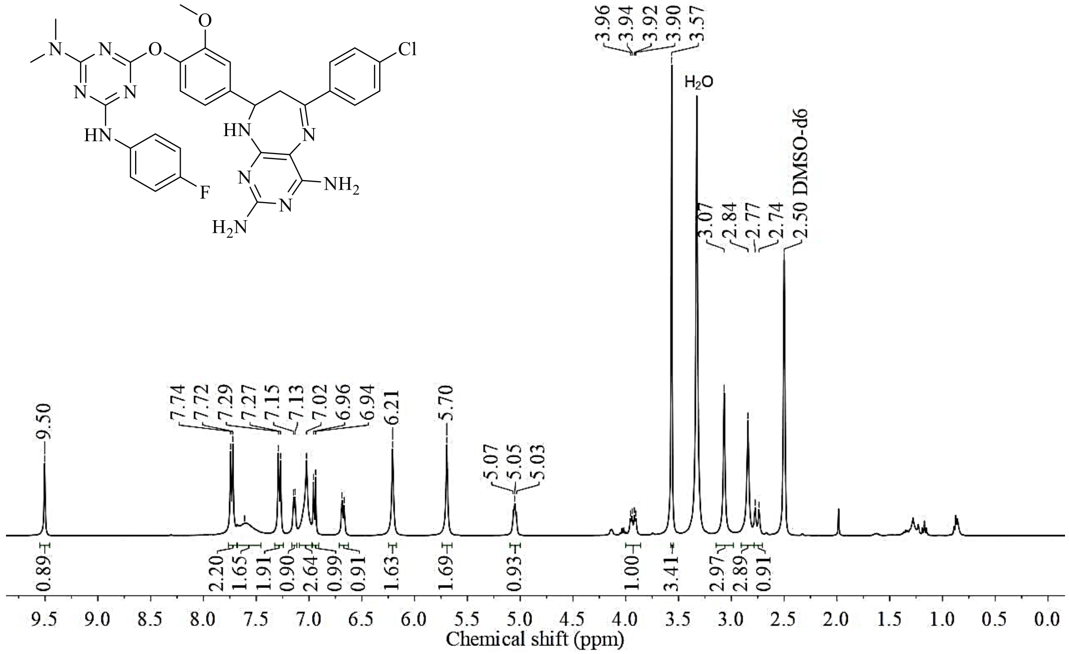

^{1}H NMR (400 MHz, DMSO-d_6) spectra of **29e**.

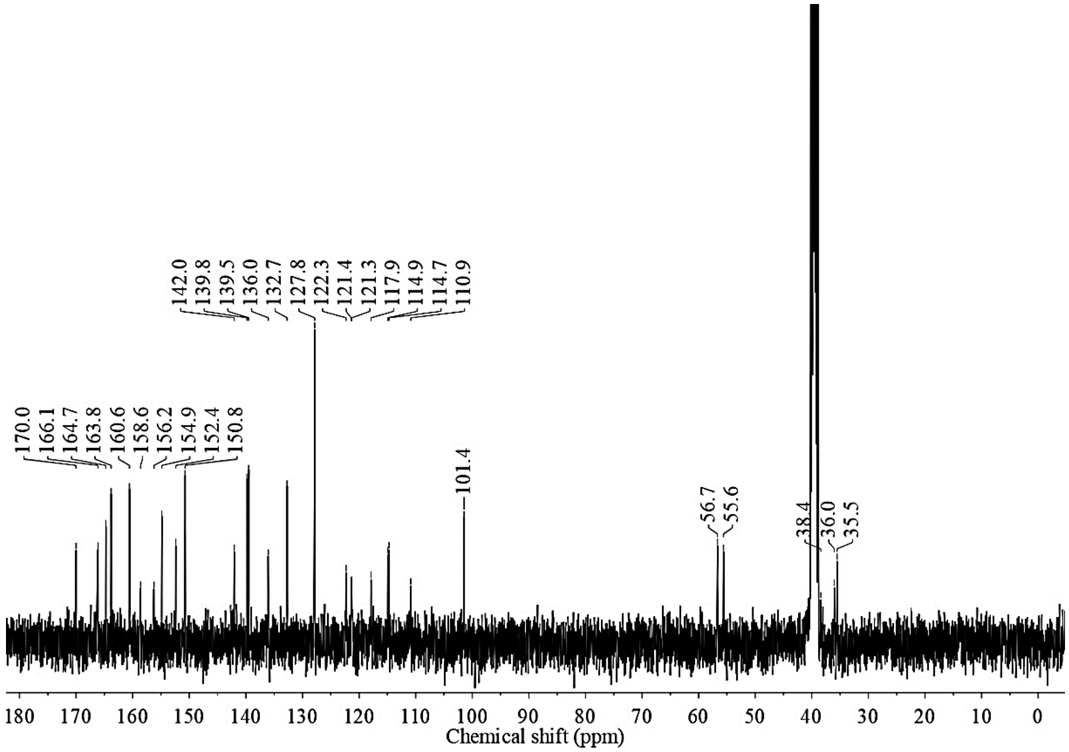

^{13}C NMR (100 MHz, DMSO-d_6) spectra of **29e**.

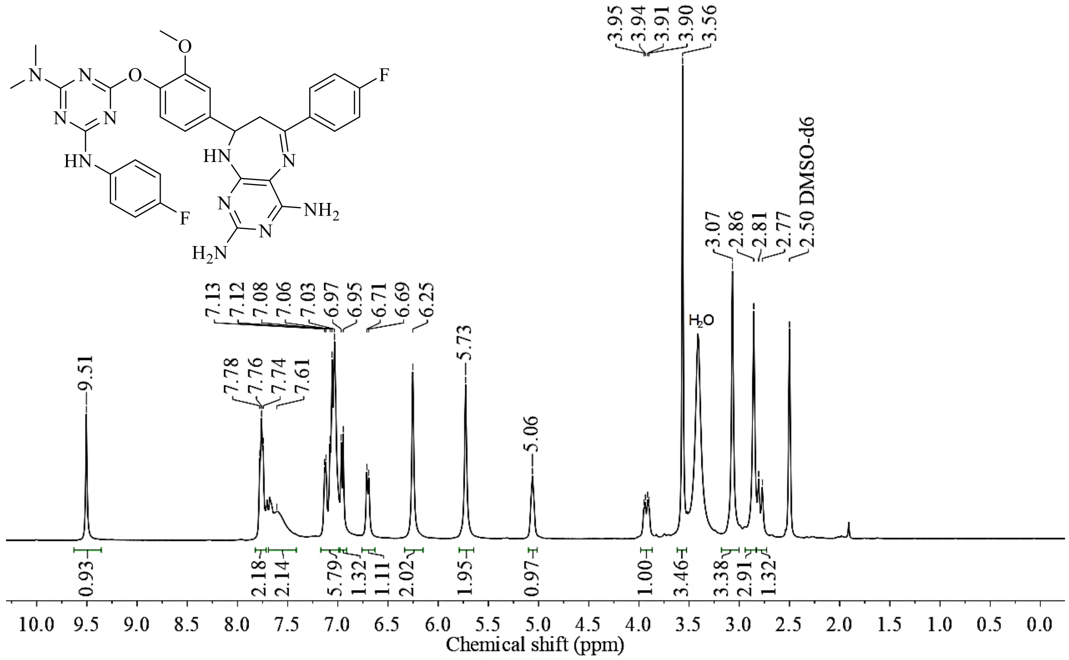

^{1}H NMR (400 MHz, DMSO-d_6) spectra of **29f**.

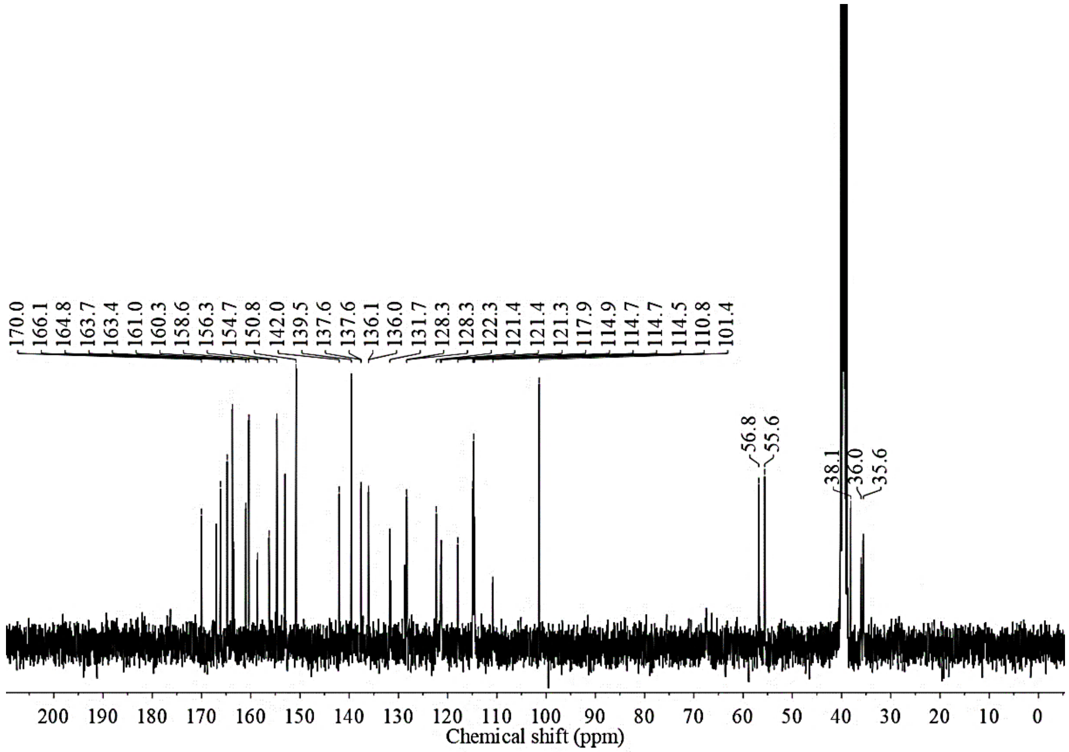

^{13}C NMR (100 MHz, DMSO-d_6) spectra of **29f**.

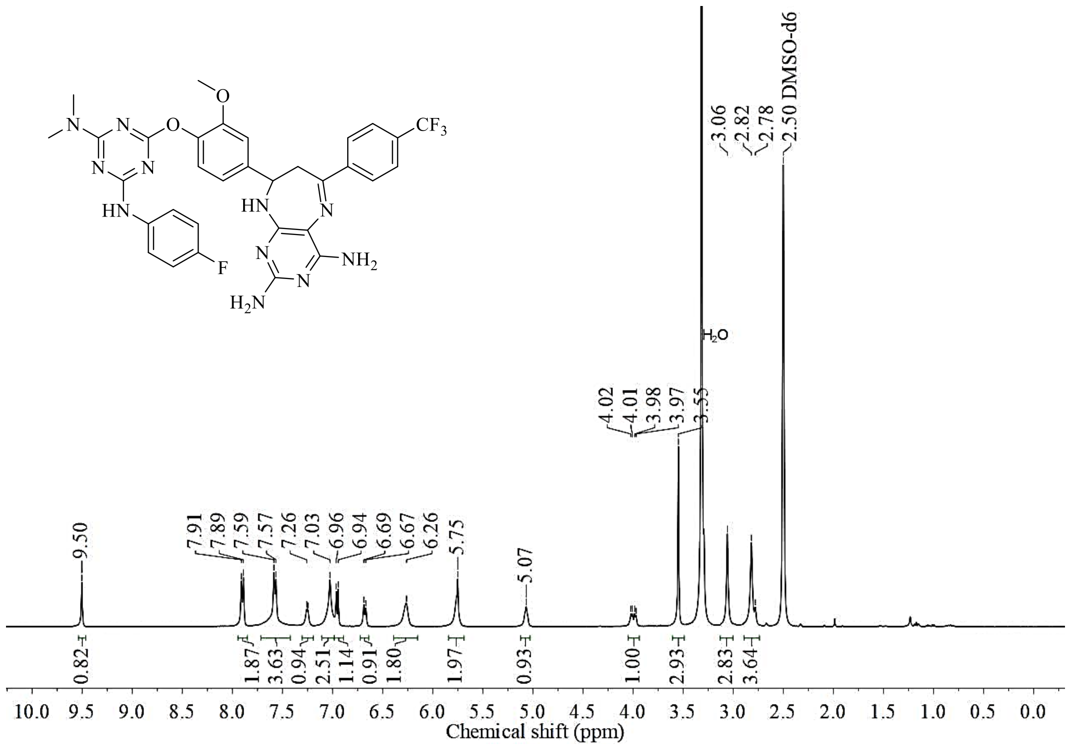

^{1}H NMR (400 MHz, DMSO-d_6) spectra of **29g**.

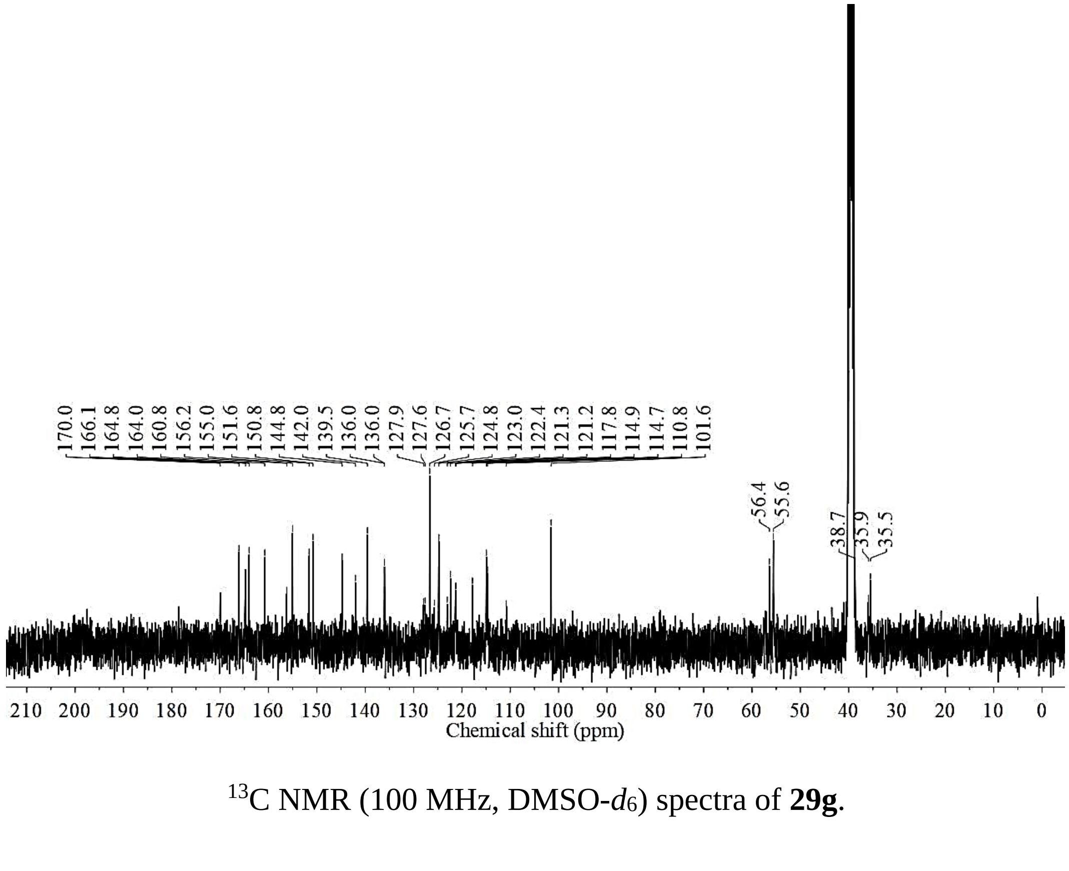

^{13}C NMR (100 MHz, DMSO-d_6) spectra of **29g**.

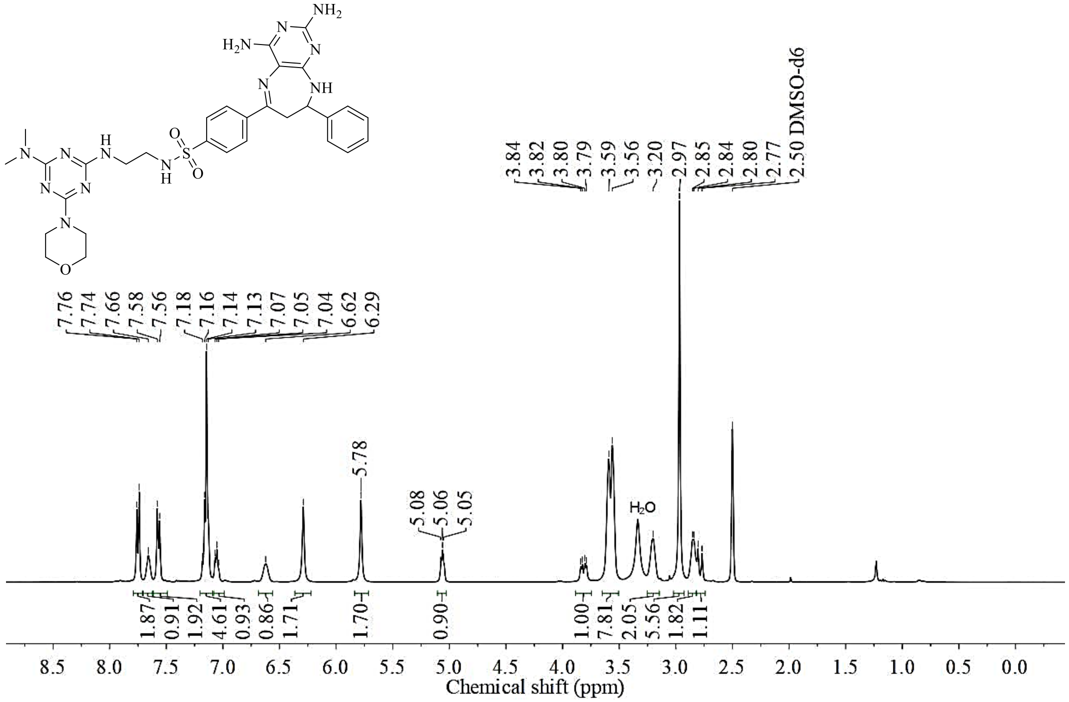

^{1}H NMR (400 MHz, DMSO-d_6) spectra of **30a**.

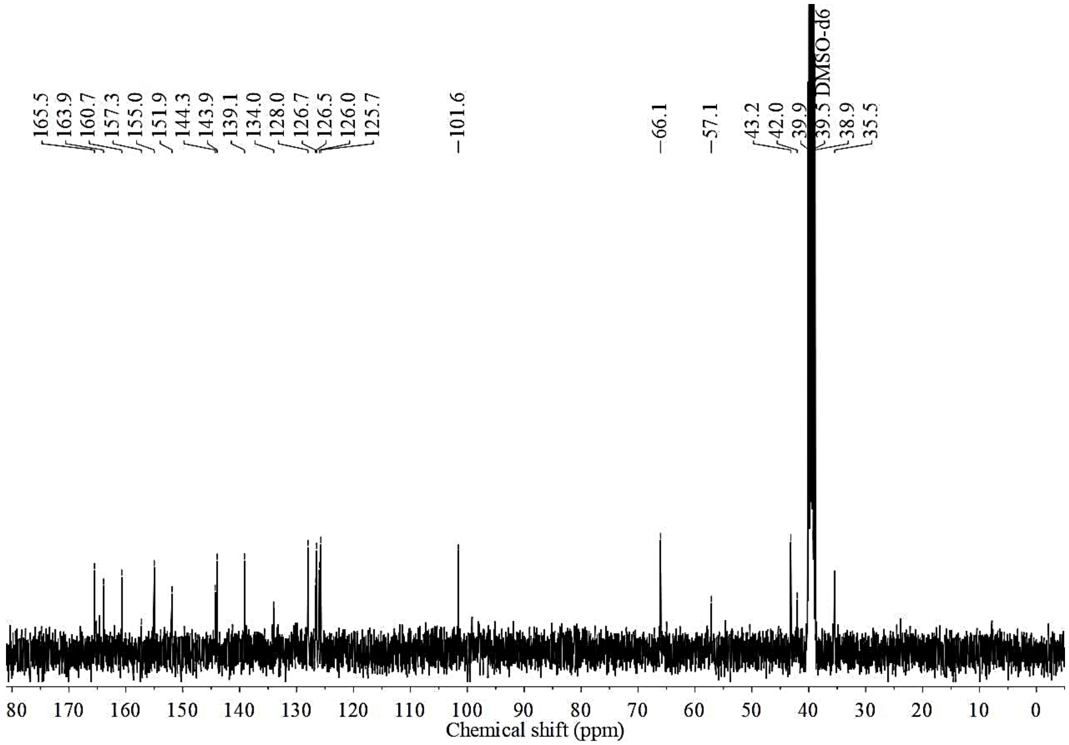

^{13}C NMR (100 MHz, DMSO-d_6) spectra of **30a**.

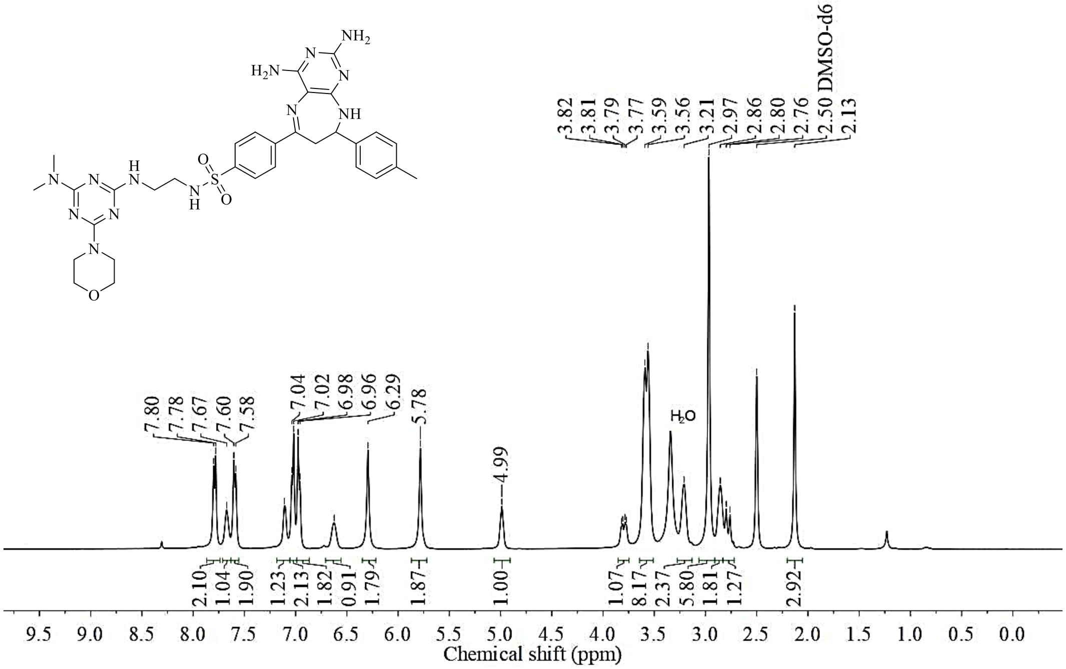

^{1}H NMR (400 MHz, DMSO-d_6) spectra of **30b**.

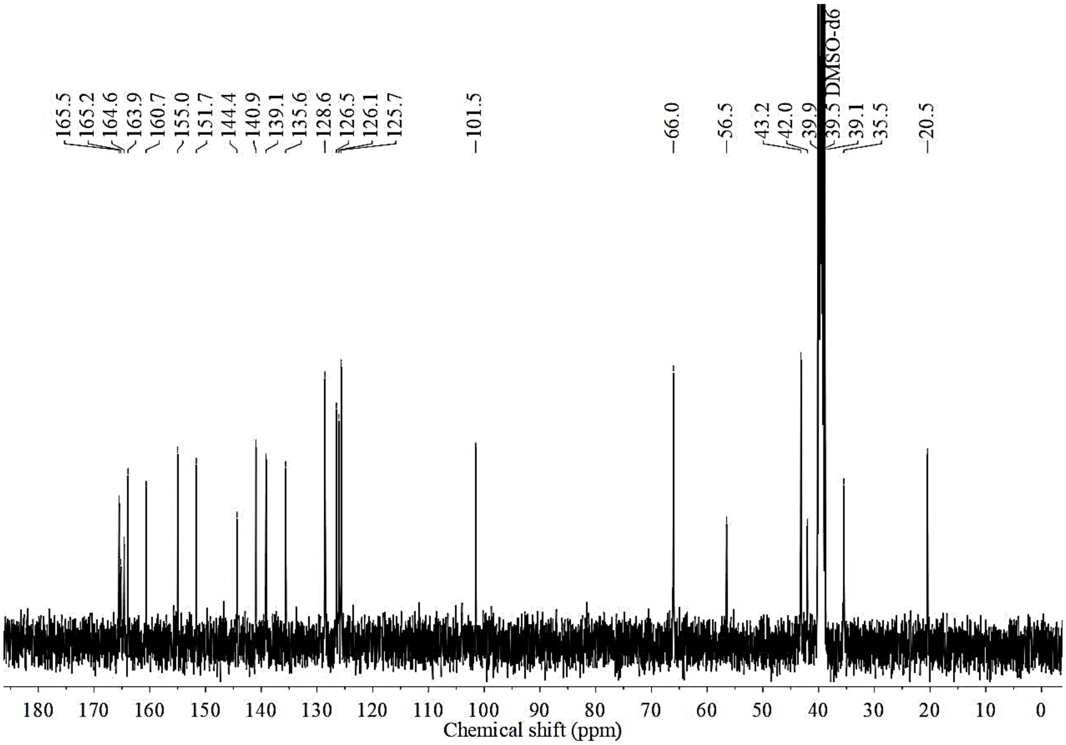

^{13}C NMR (100 MHz, DMSO-d_6) spectra of **30b**.

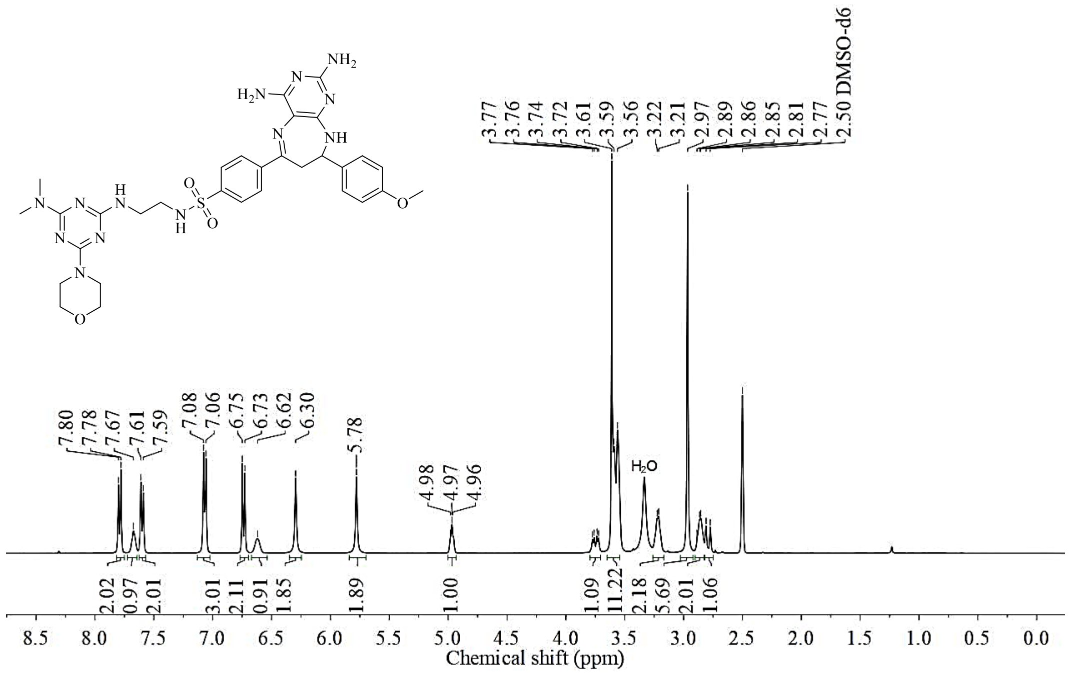

^{1}H NMR (400 MHz, DMSO-d_6) spectra of **30c**.

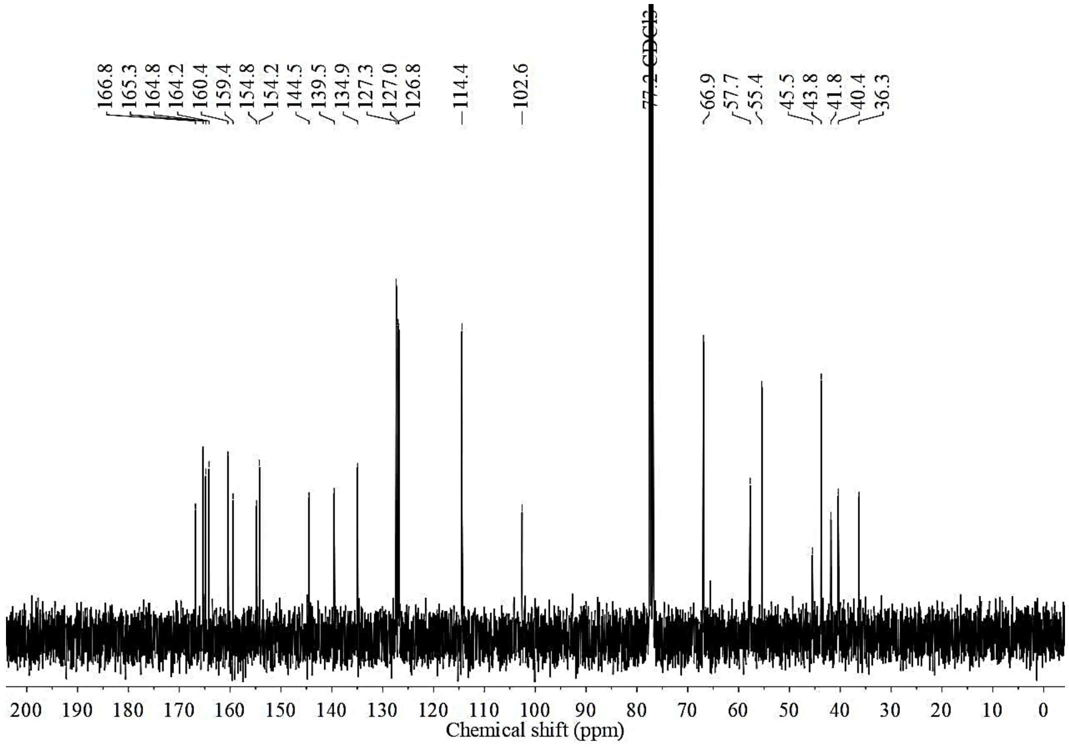

^{13}C NMR (100 MHz, $CDCl_3$) spectra of **30c**.

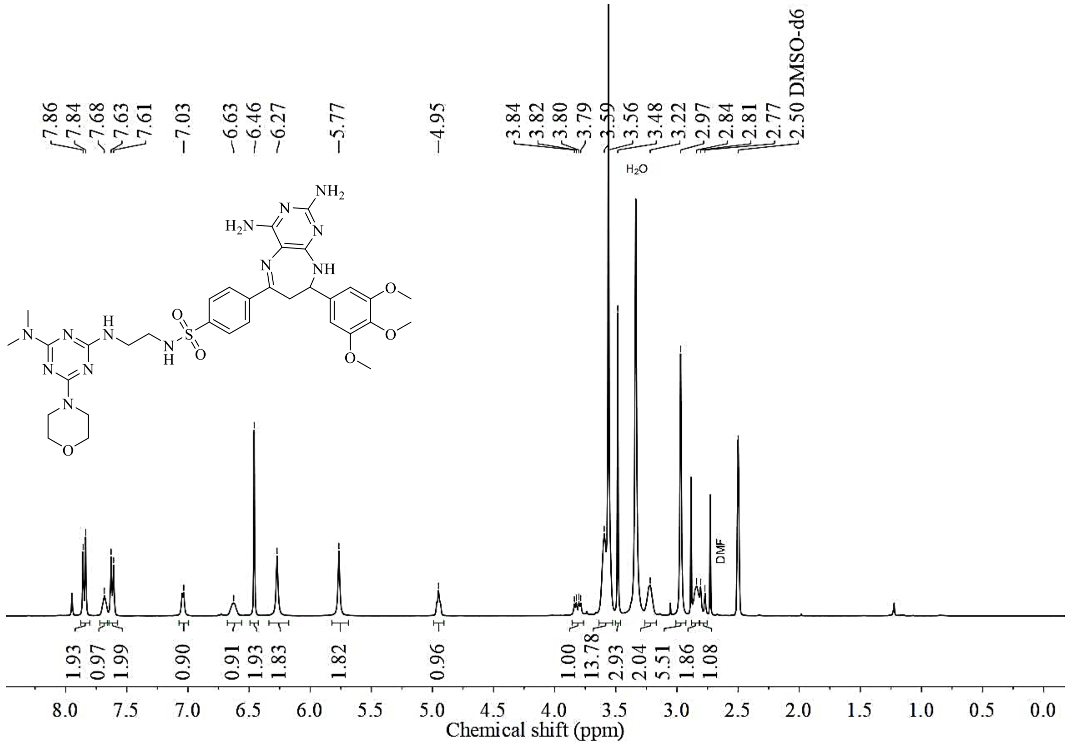

^{1}H NMR (400 MHz, DMSO-d_6) spectra of **30d**.

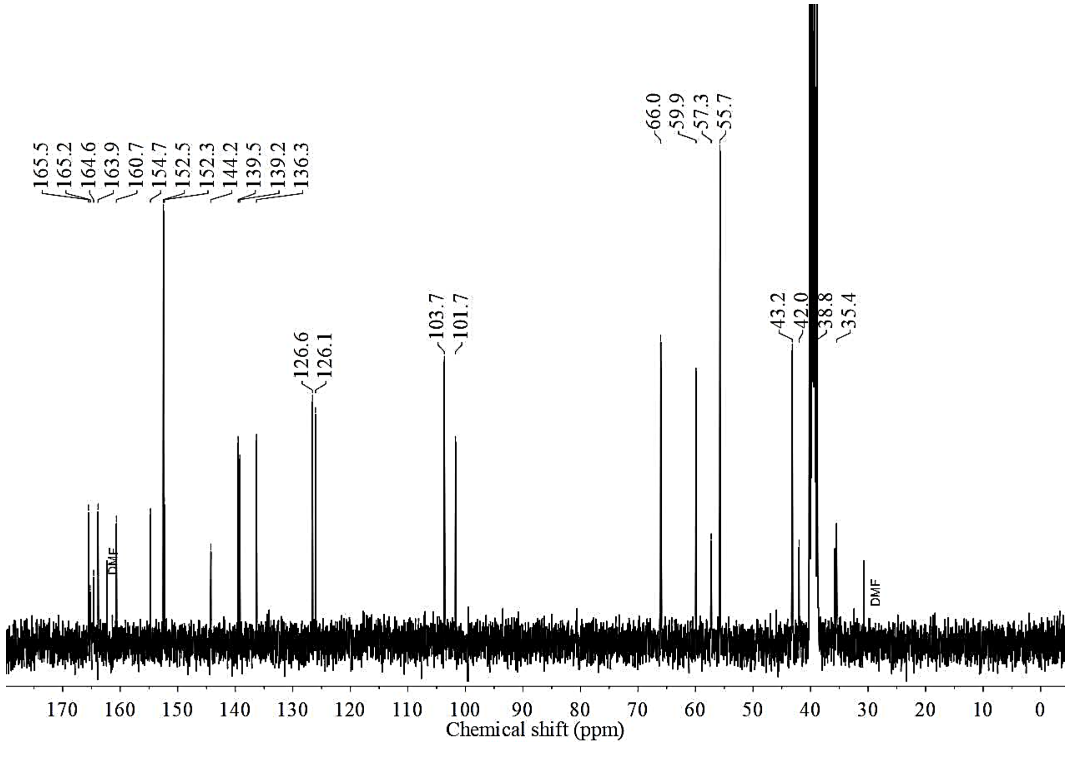

^{13}C NMR (100 MHz, DMSO-d_6) spectra of **30d**.

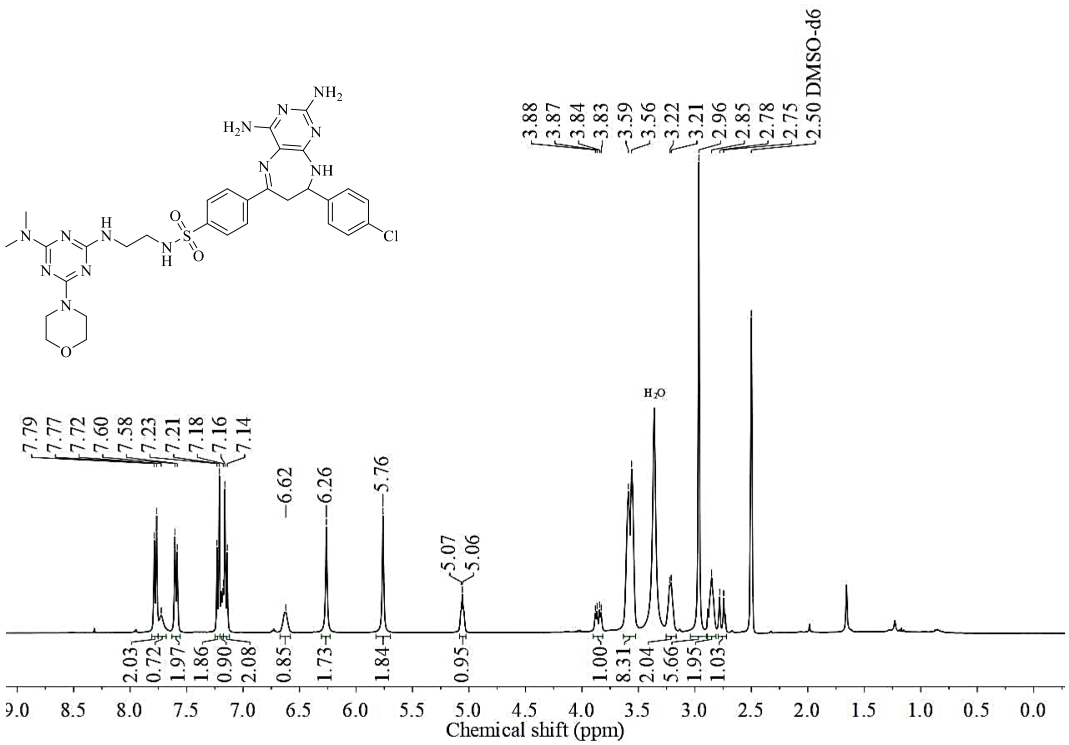

^{1}H NMR (400 MHz, DMSO-d_6) spectra of **30e**.

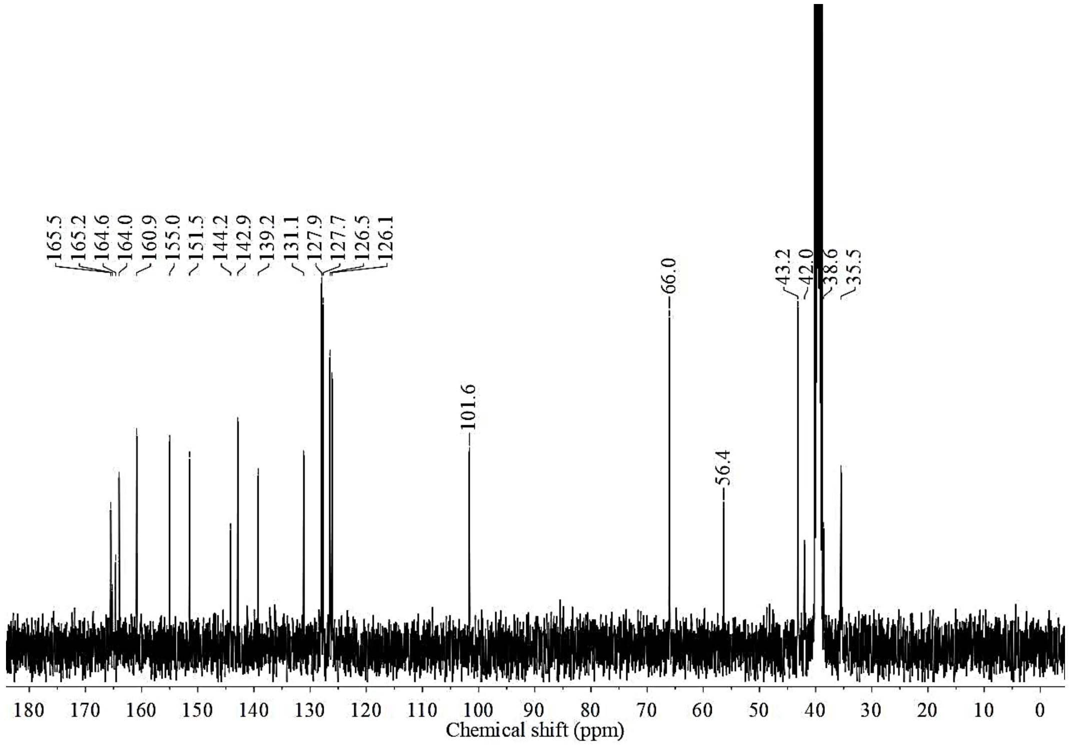

^{13}C NMR (100 MHz, DMSO-d_6) spectra of **30e**.

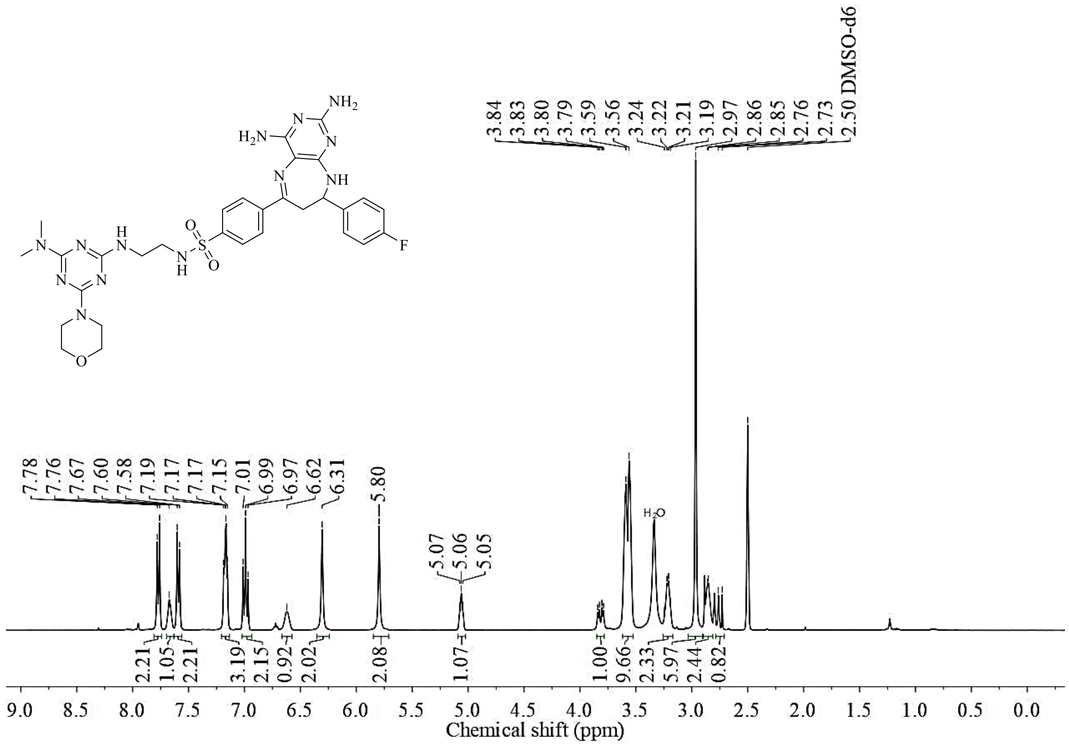

^{1}H NMR (400 MHz, DMSO-*d*6) spectra of **30f**.

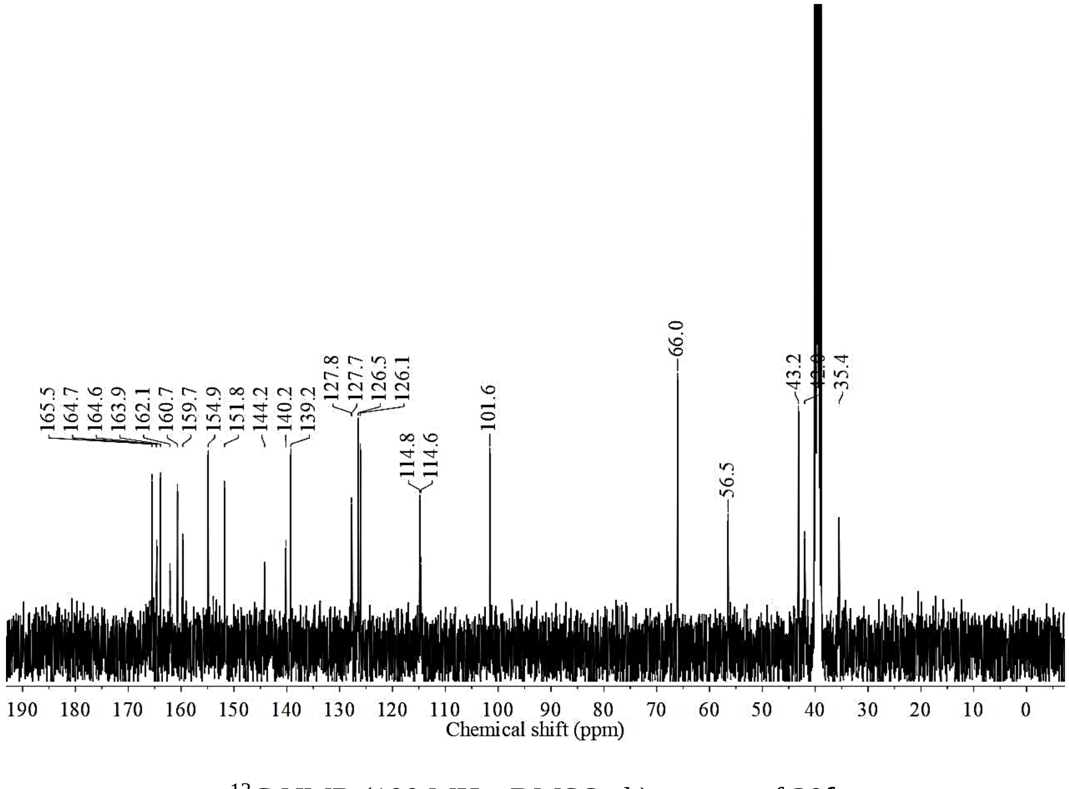

^{13}C NMR (100 MHz, DMSO-*d*6) spectra of **30f**.

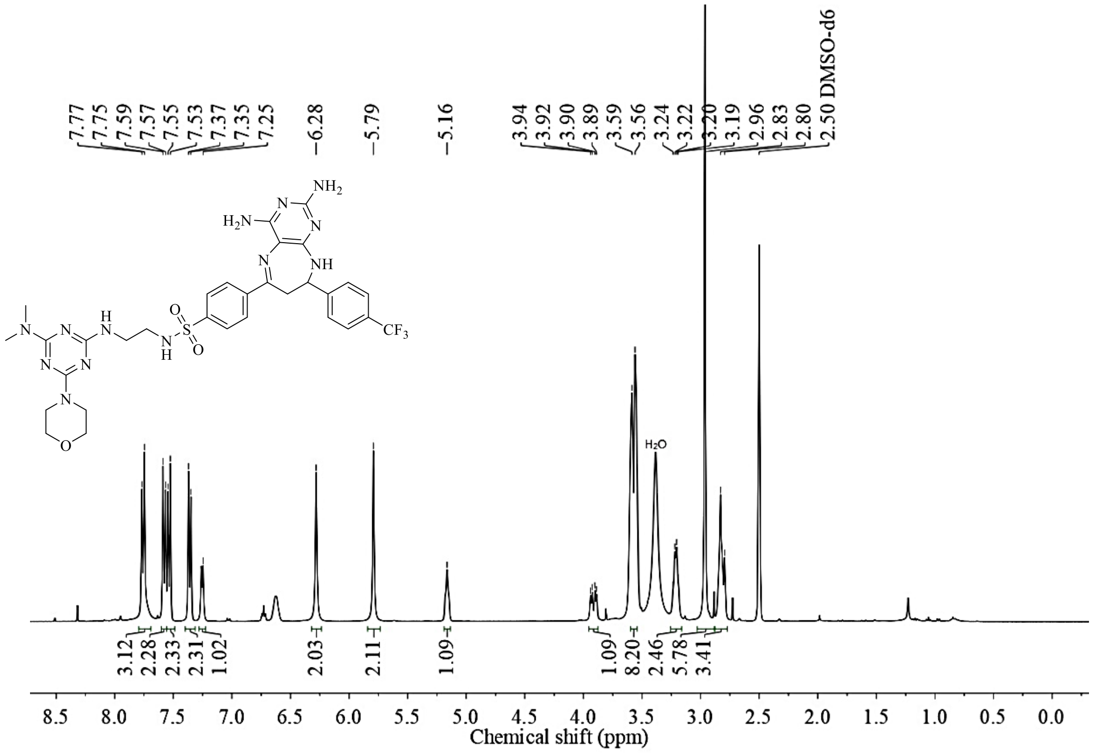

^{1}H NMR (400 MHz, DMSO-d_6) spectra of **30g**.

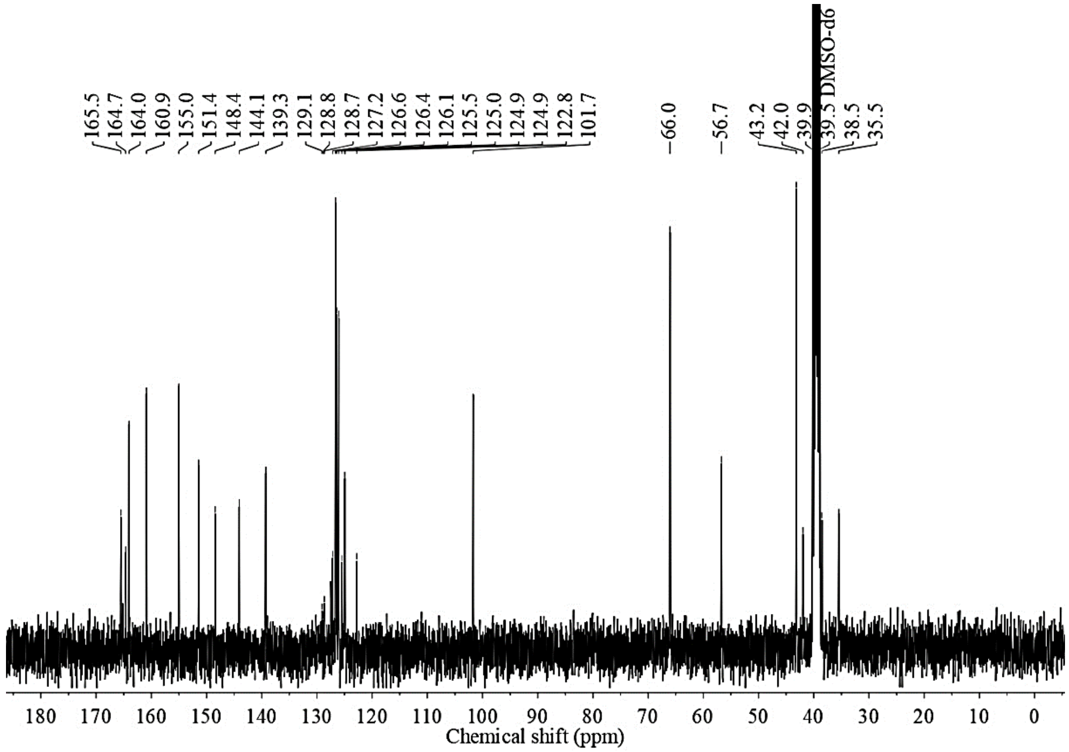

^{13}C NMR (100 MHz, DMSO-d_6) spectra of **30g**.

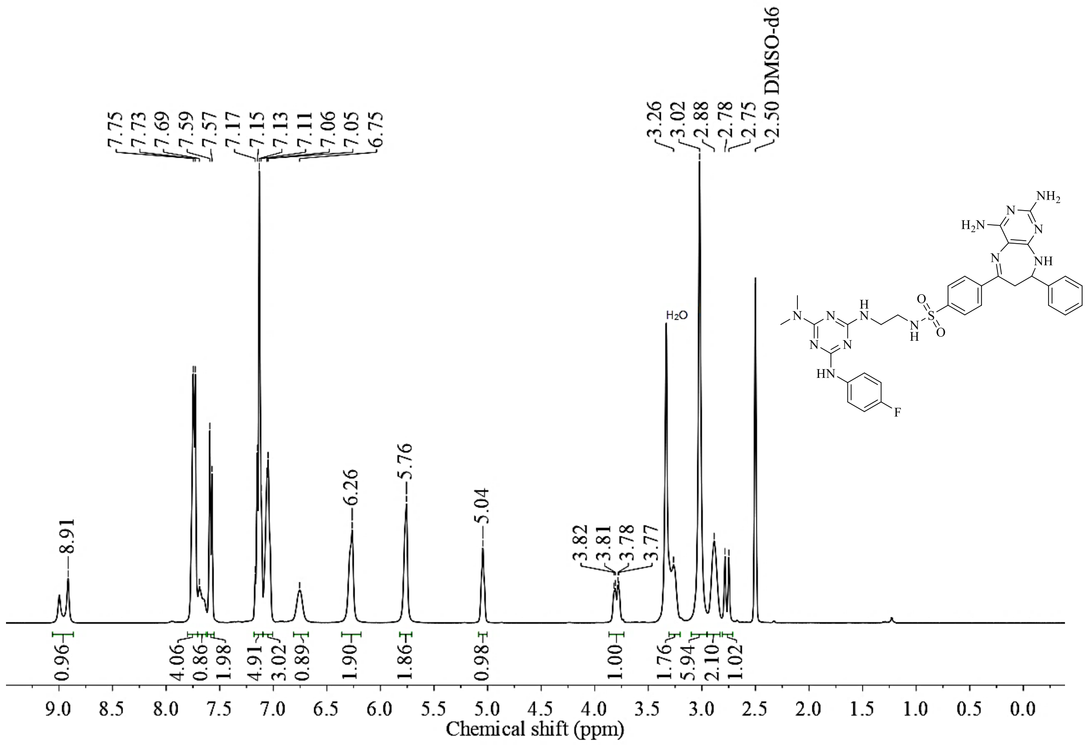

^{1}H NMR (400 MHz, DMSO-d_6) spectra of **31a**.

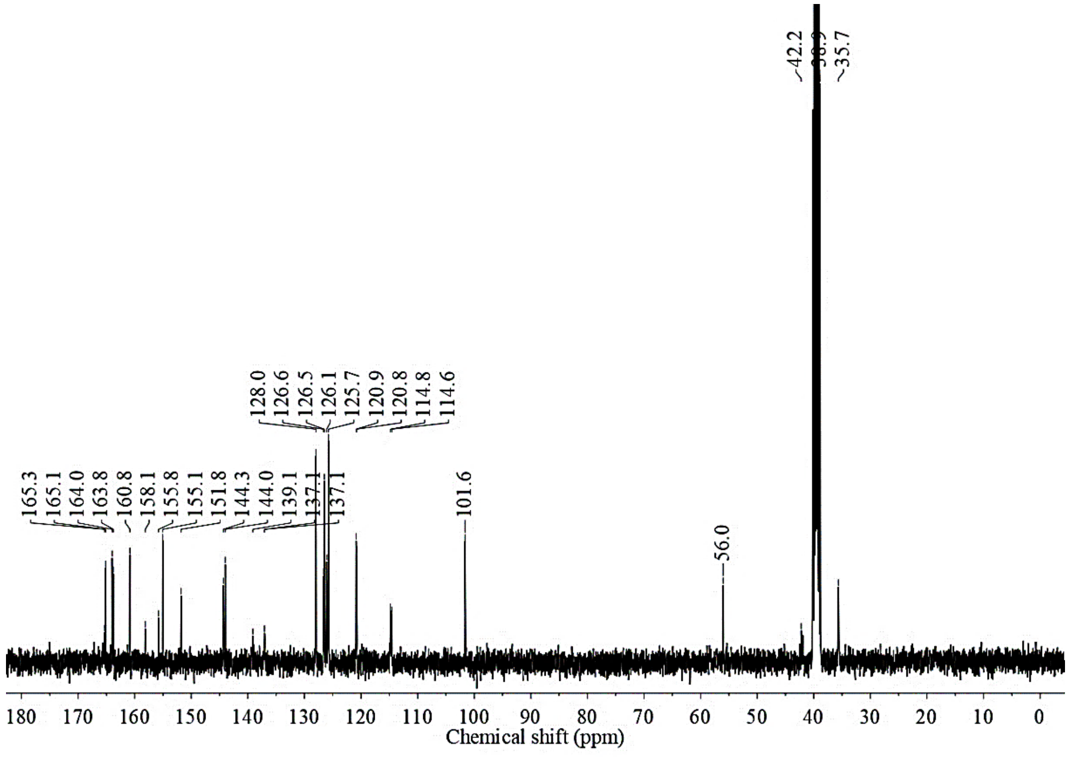

^{13}C NMR (100 MHz, DMSO-d_6) spectra of **31a**.

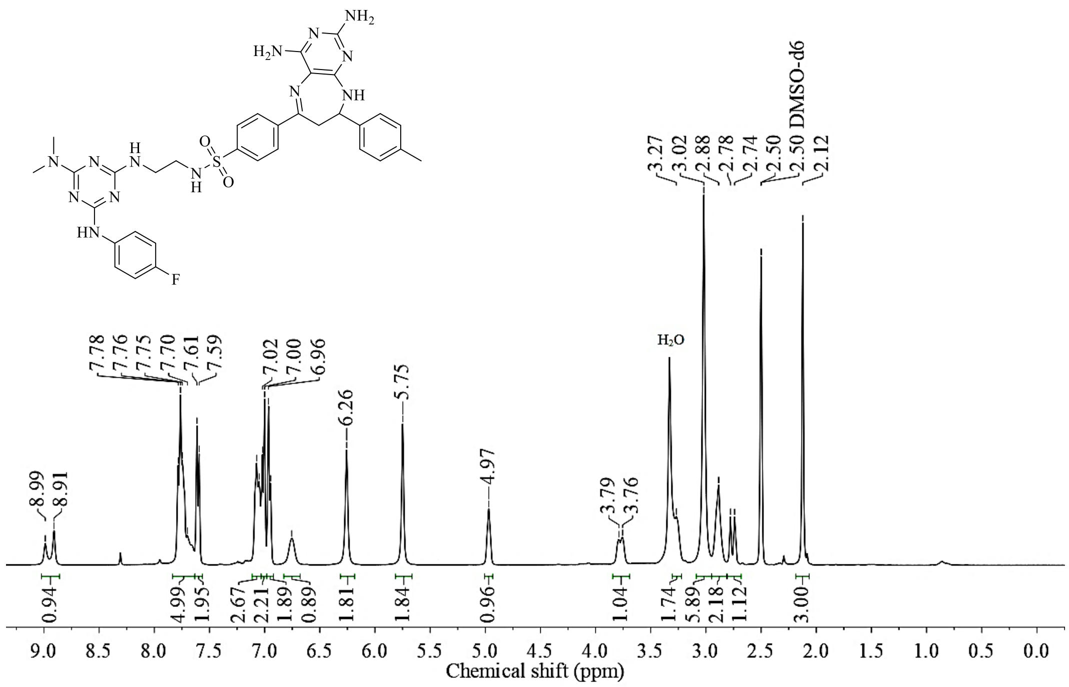

^{1}H NMR (400 MHz, DMSO-d_6) spectra of **31b**.

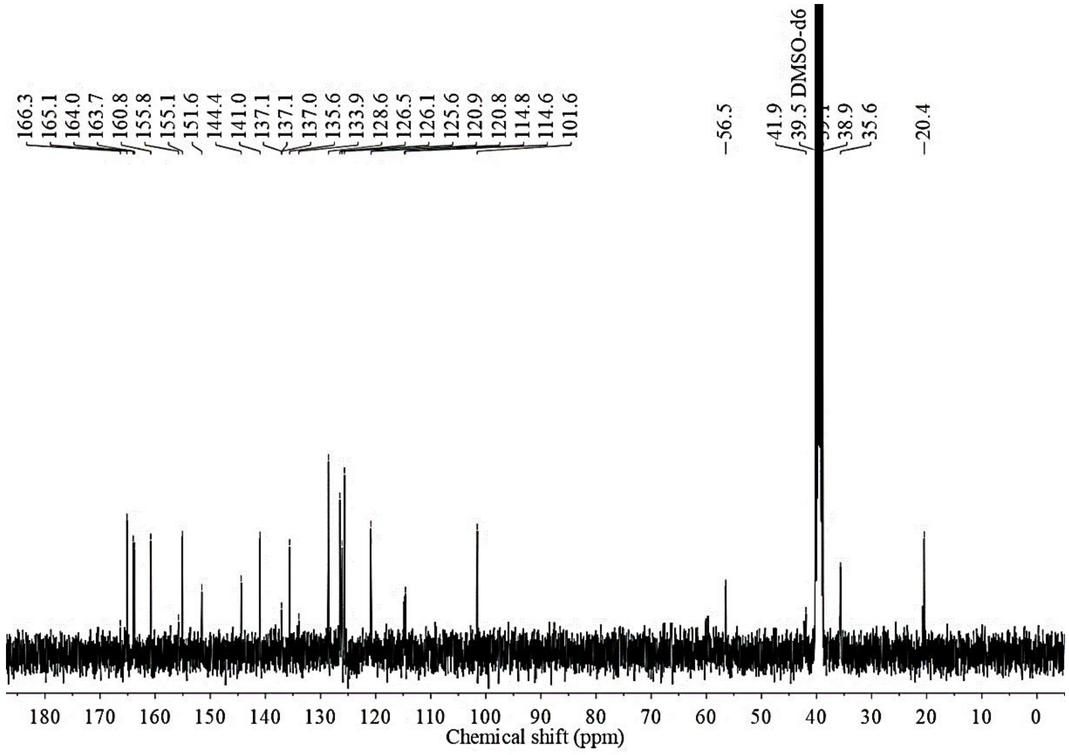

^{13}C NMR (100 MHz, DMSO-d_6) spectra of **31b**.

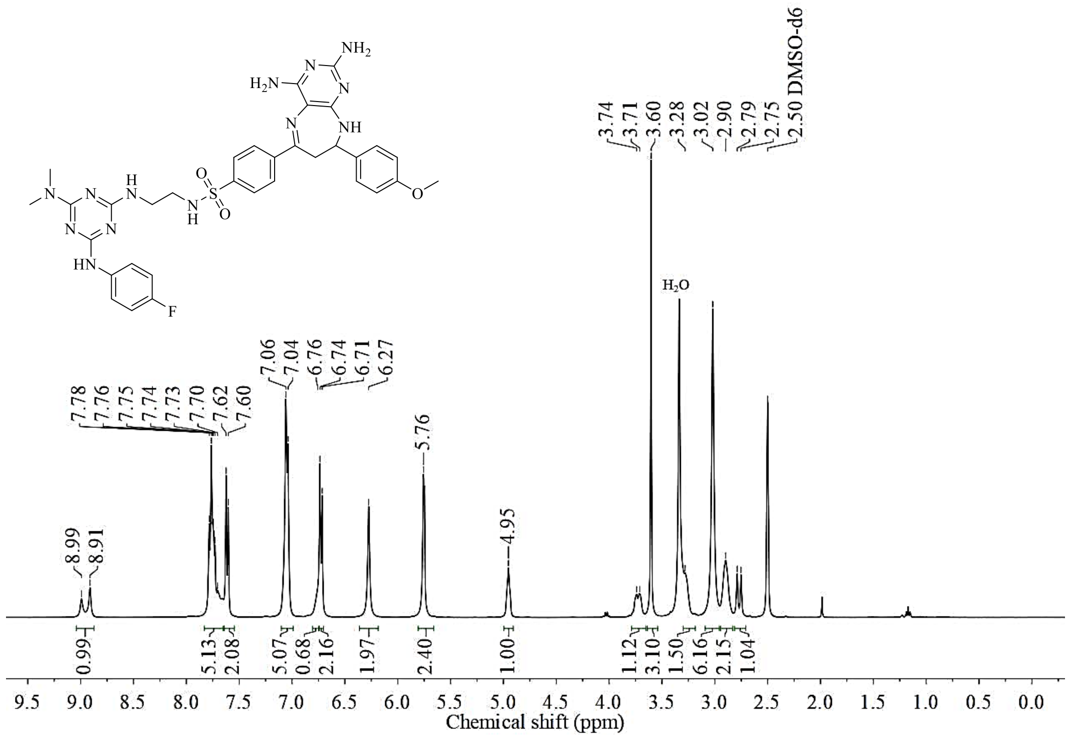

^{1}H NMR (400 MHz, DMSO-d_6) spectra of **31c**.

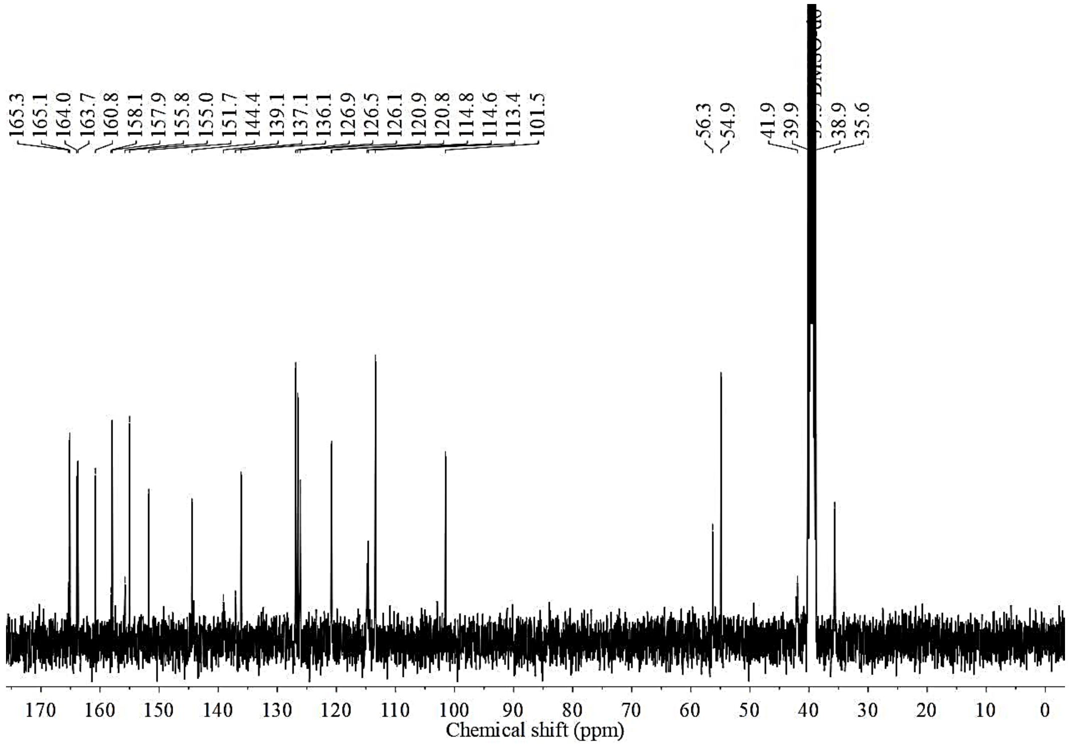

^{13}C NMR (100 MHz, DMSO-d_6) spectra of **31c**.

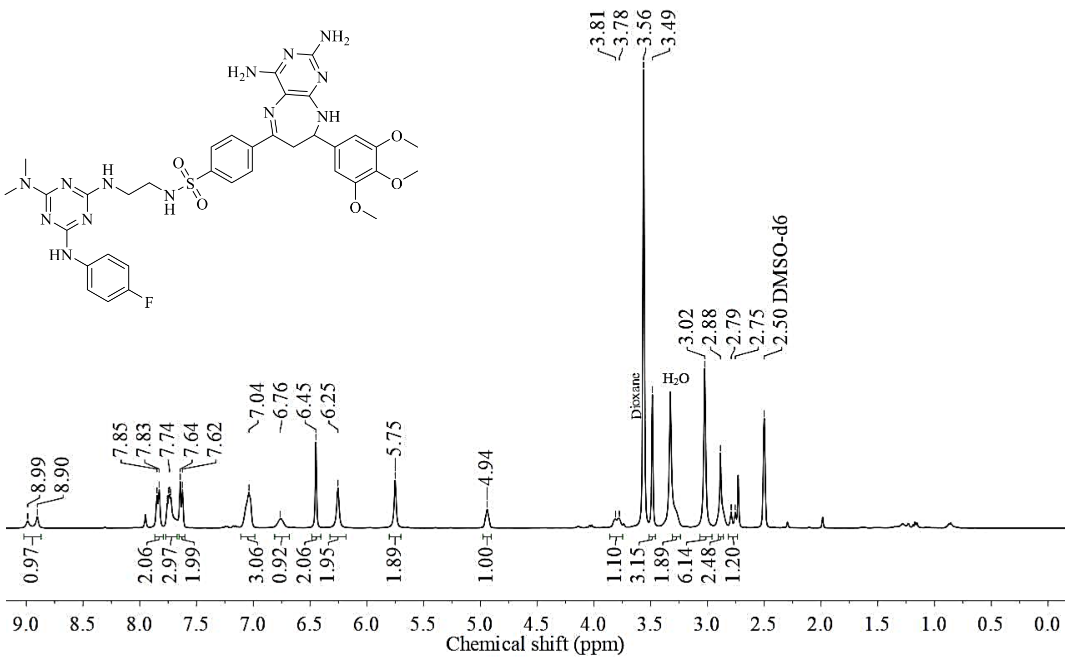

^{1}H NMR (400 MHz, DMSO-d_6) spectra of **31d**.

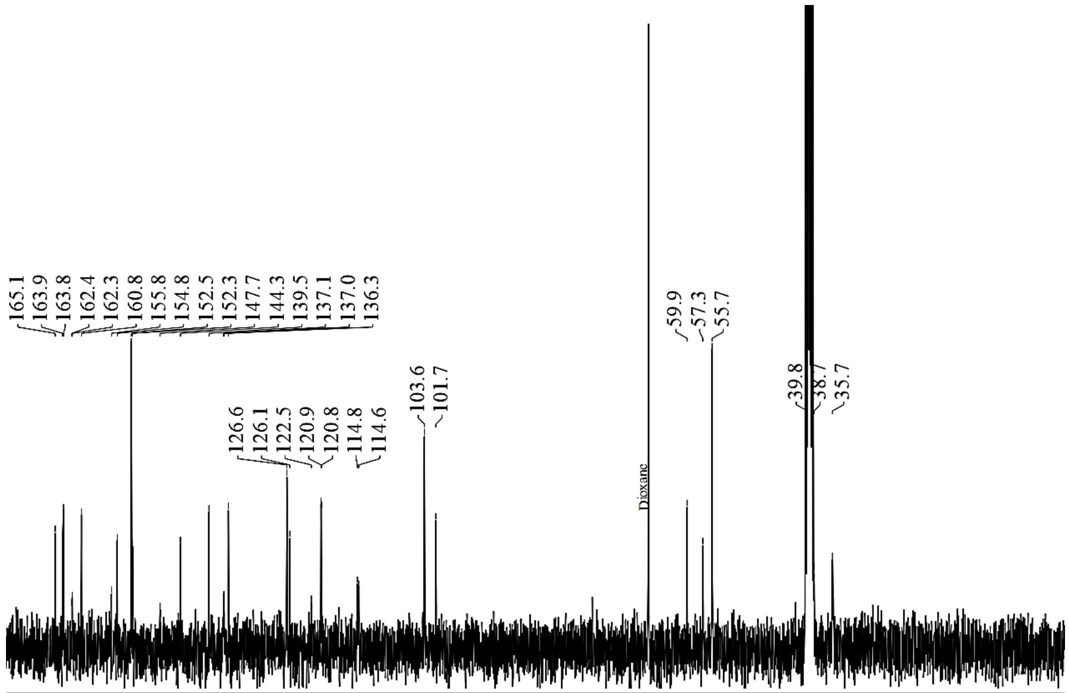

^{13}C NMR (100 MHz, DMSO-d_6) spectra of **31d**.

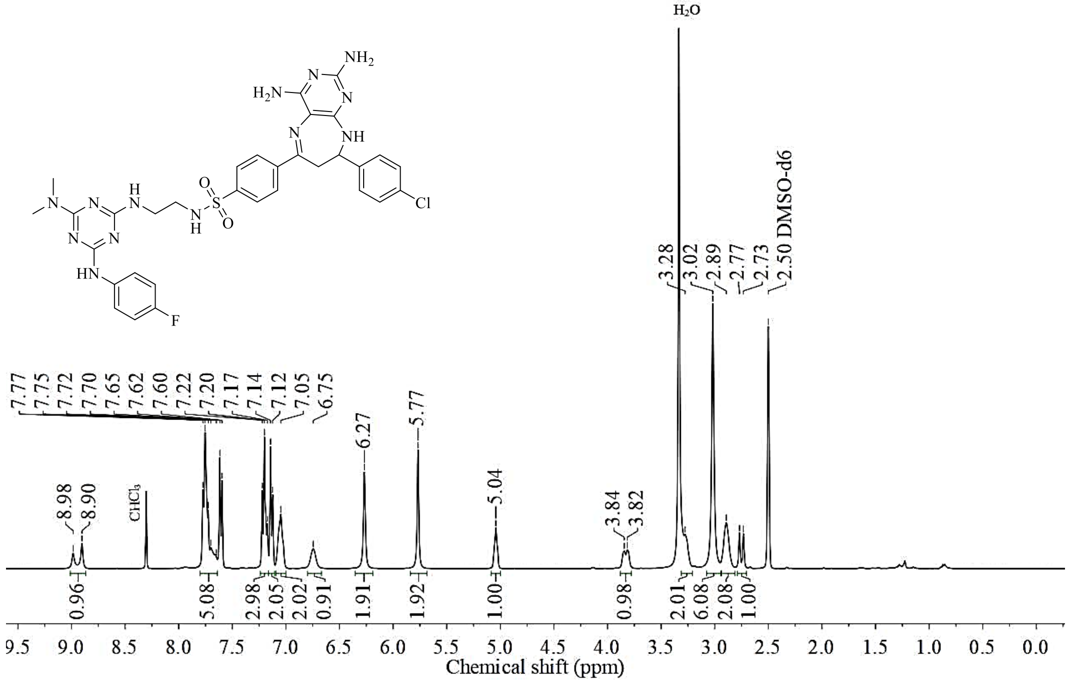

^{1}H NMR (400 MHz, DMSO-*d*6) spectra of **31e**.

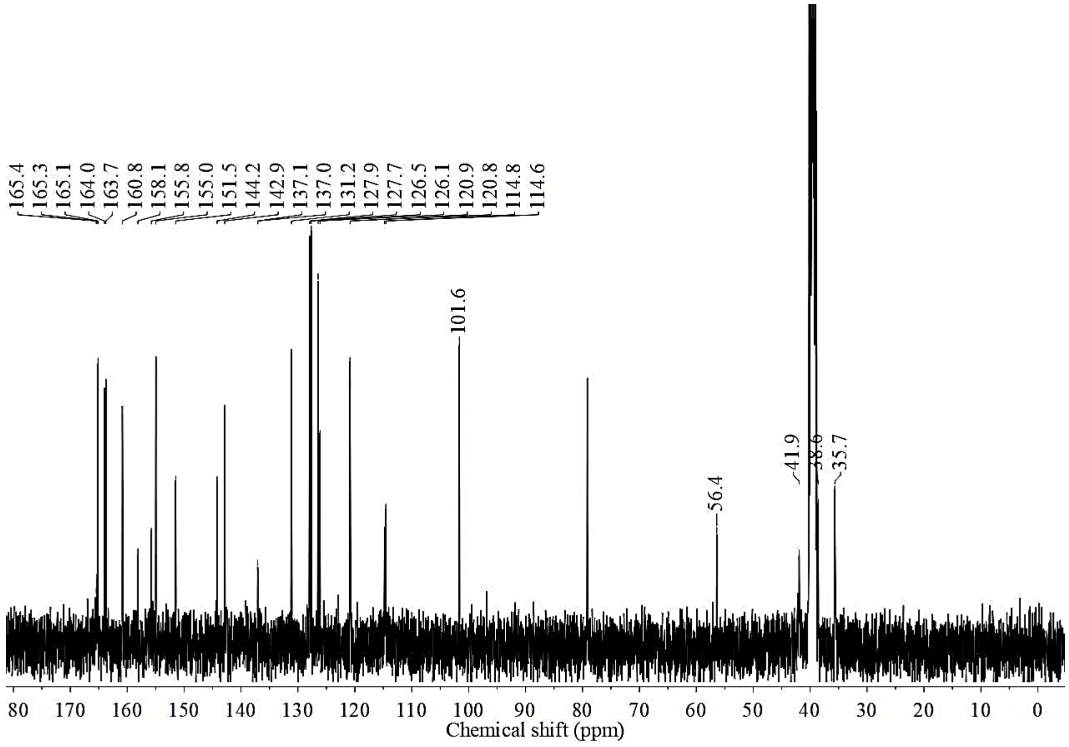

^{13}C NMR (100 MHz, DMSO-*d*6) spectra of **31e**.

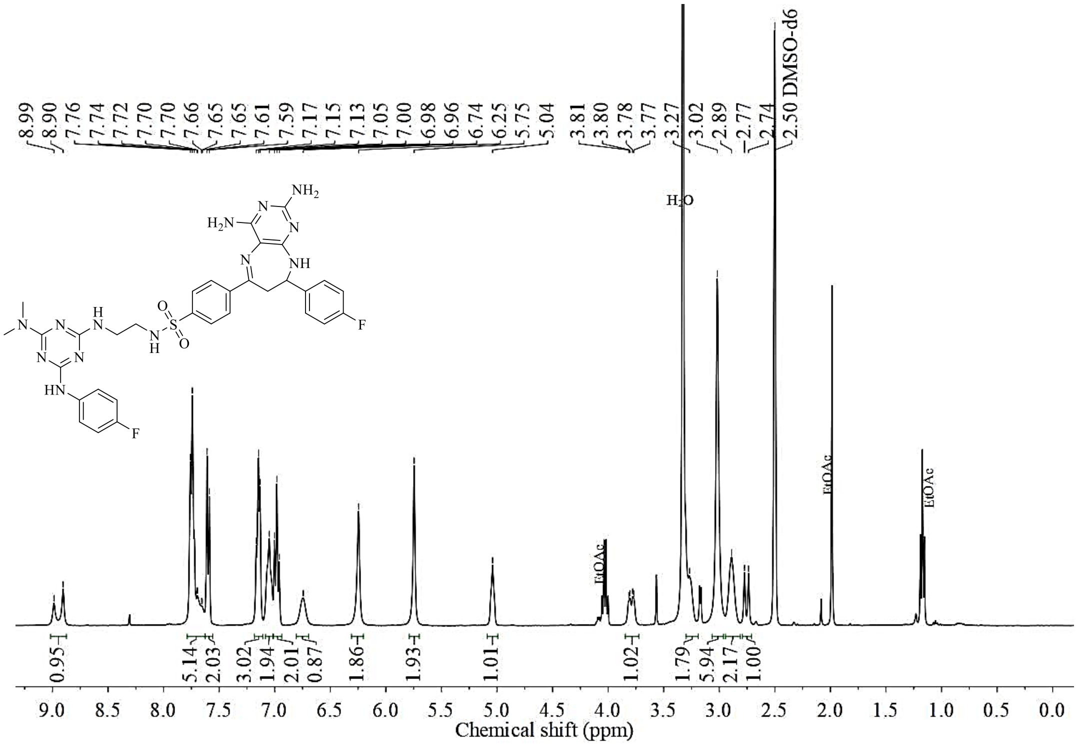

^{1}H NMR (400 MHz, DMSO-d_6) spectra of **31f**.

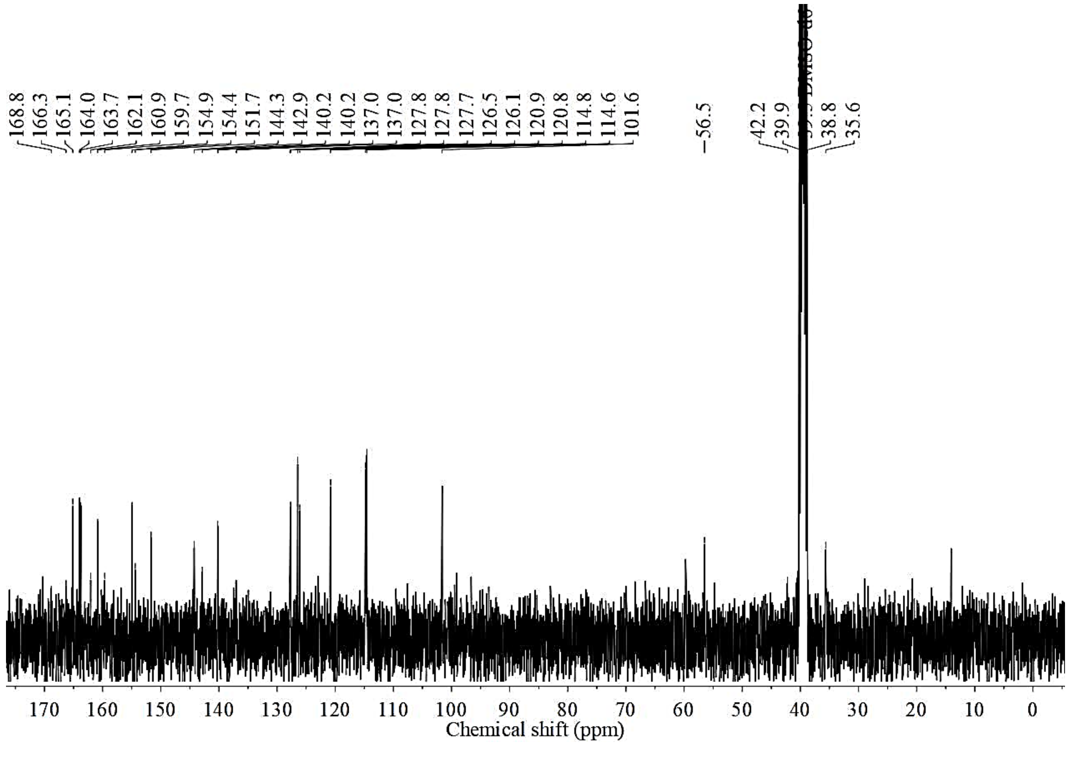

^{13}C NMR (100 MHz, DMSO-d_6) spectra of **31f**.

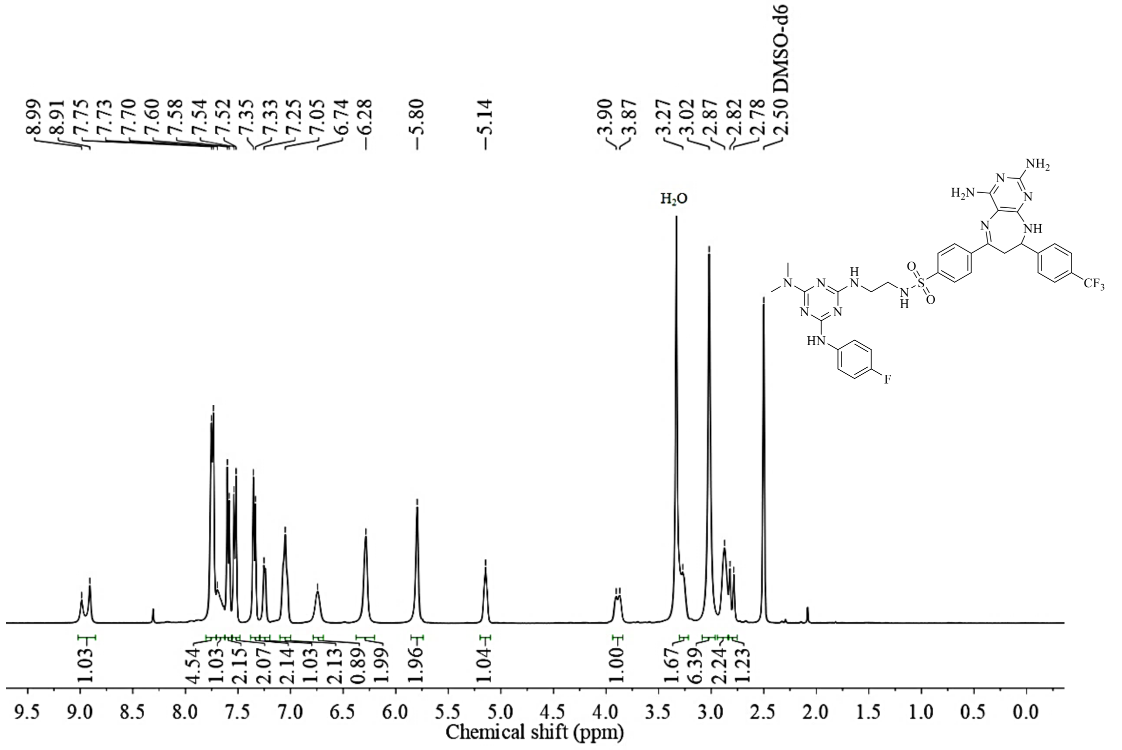

^{1}H NMR (400 MHz, DMSO-d_6) spectra of **31g**.

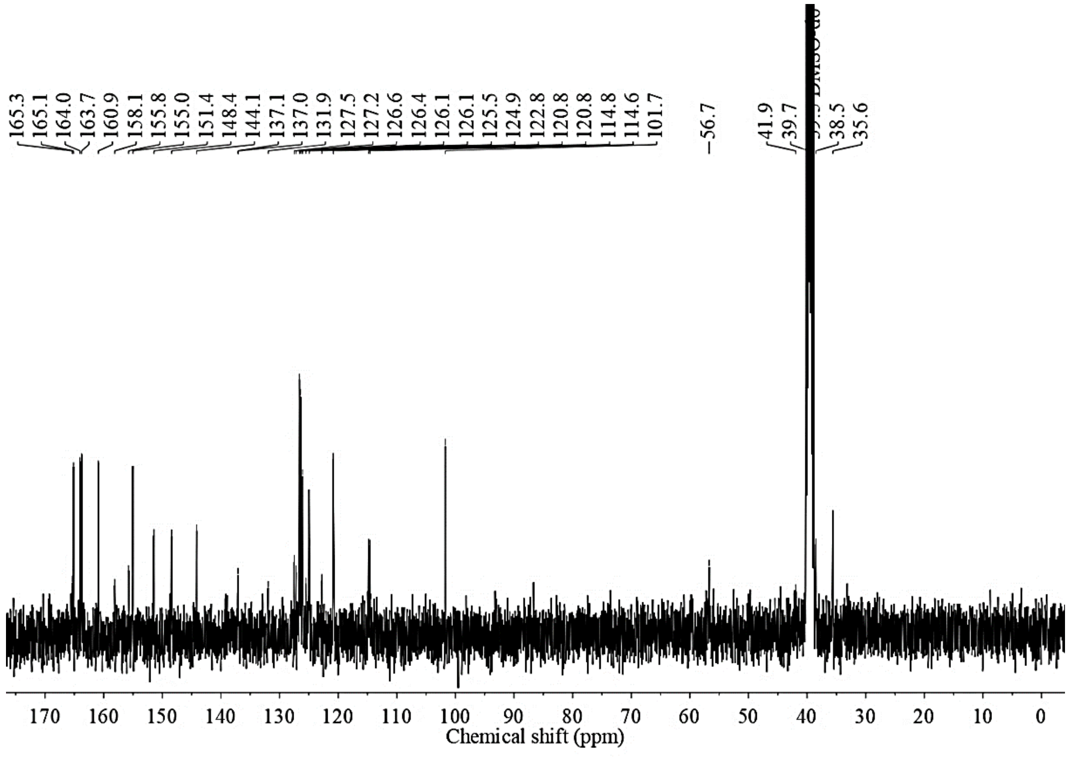

^{13}C NMR (100 MHz, DMSO-d_6) spectra of **31g**.

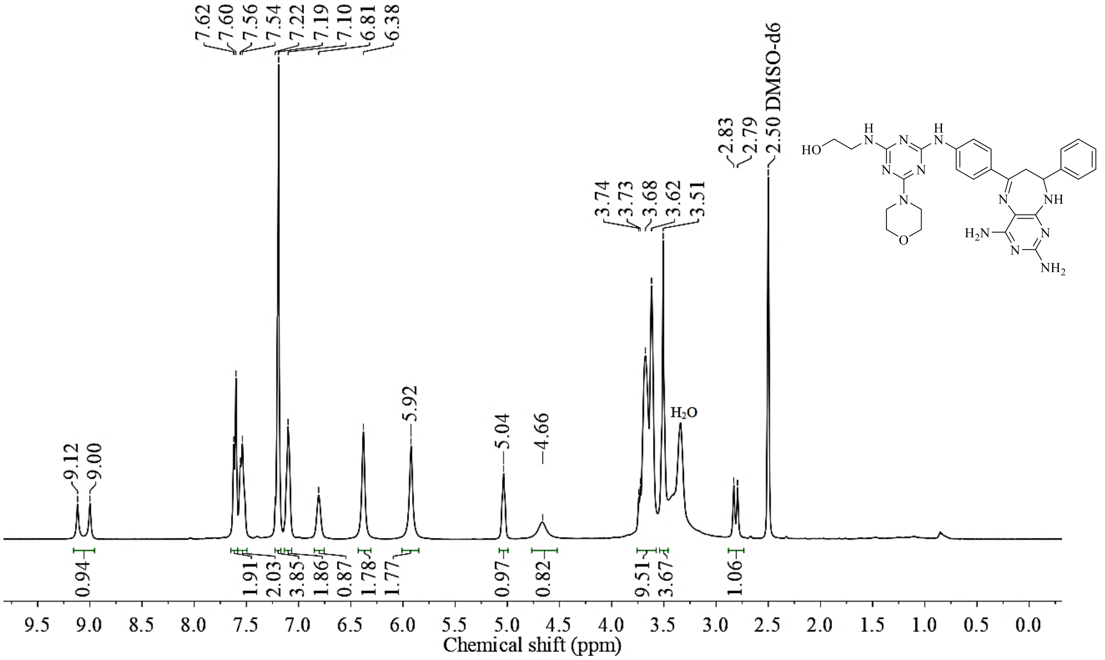

^{1}H NMR (400 MHz, DMSO-d_6) spectra of **32a**.

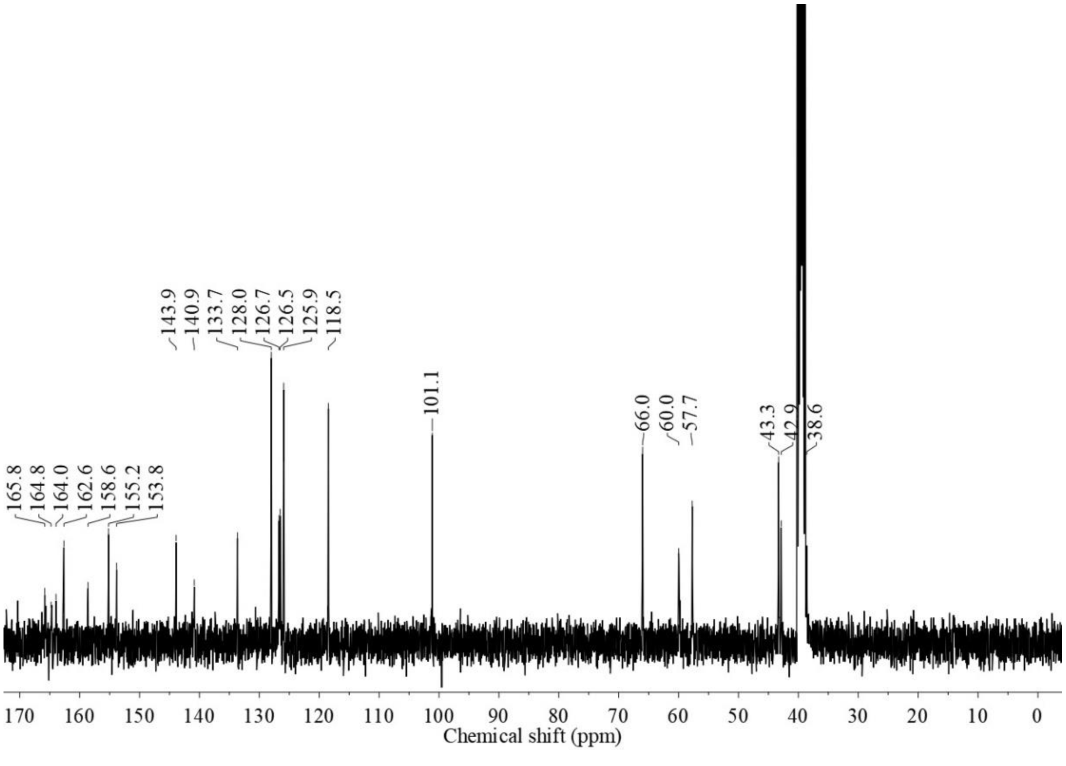

^{13}C NMR (100 MHz, DMSO-d_6) spectra of **32a**.

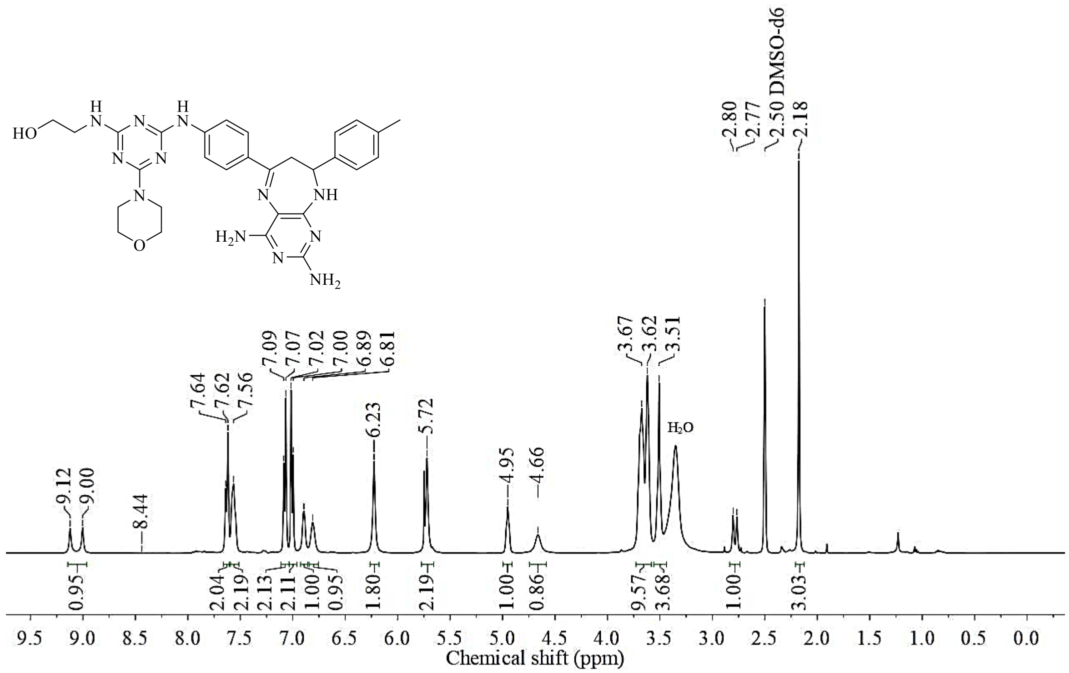

^{1}H NMR (400 MHz, DMSO-d_6) spectra of **32b**.

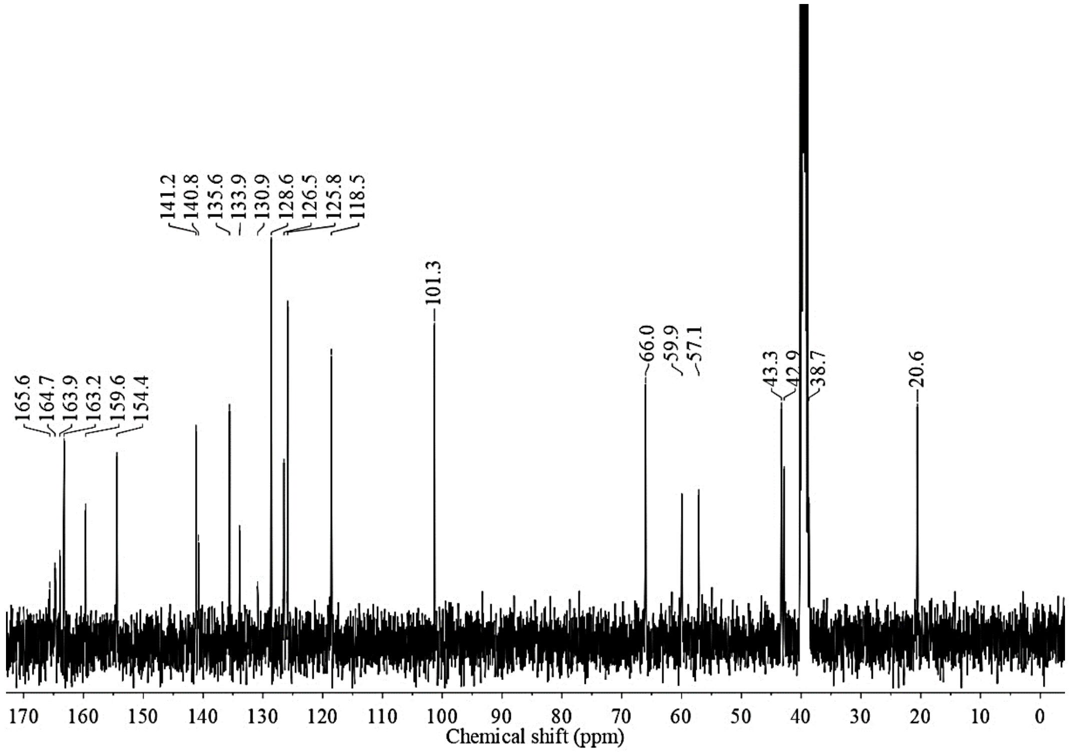

^{13}C NMR (100 MHz, DMSO-d_6) spectra of **32b**.

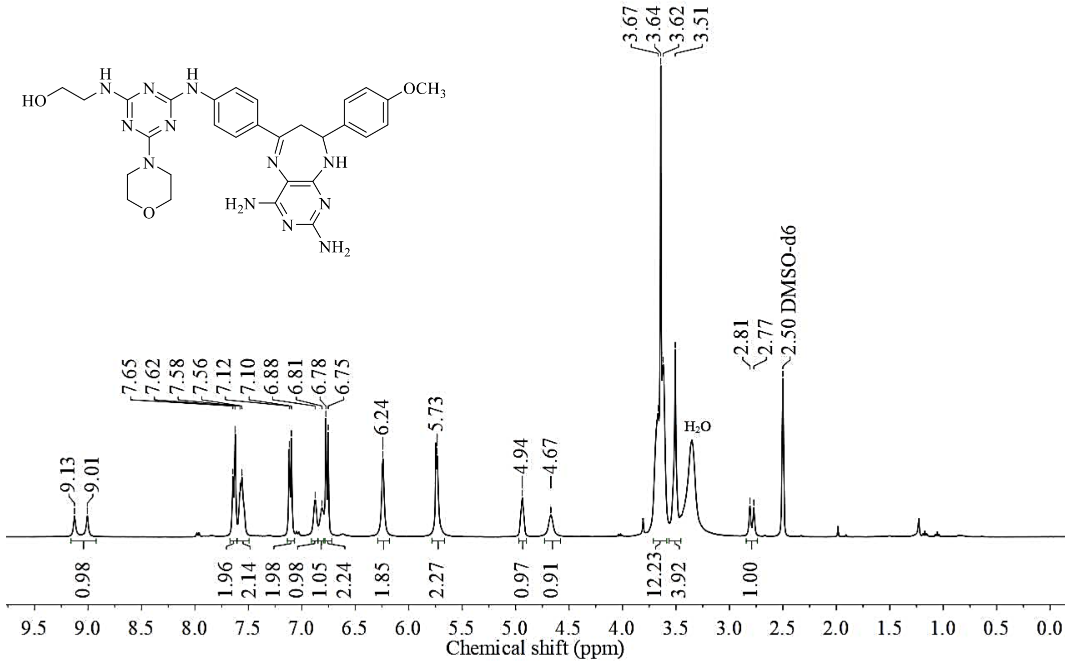

^{1}H NMR (400 MHz, DMSO-d_6) spectra of **32c**.

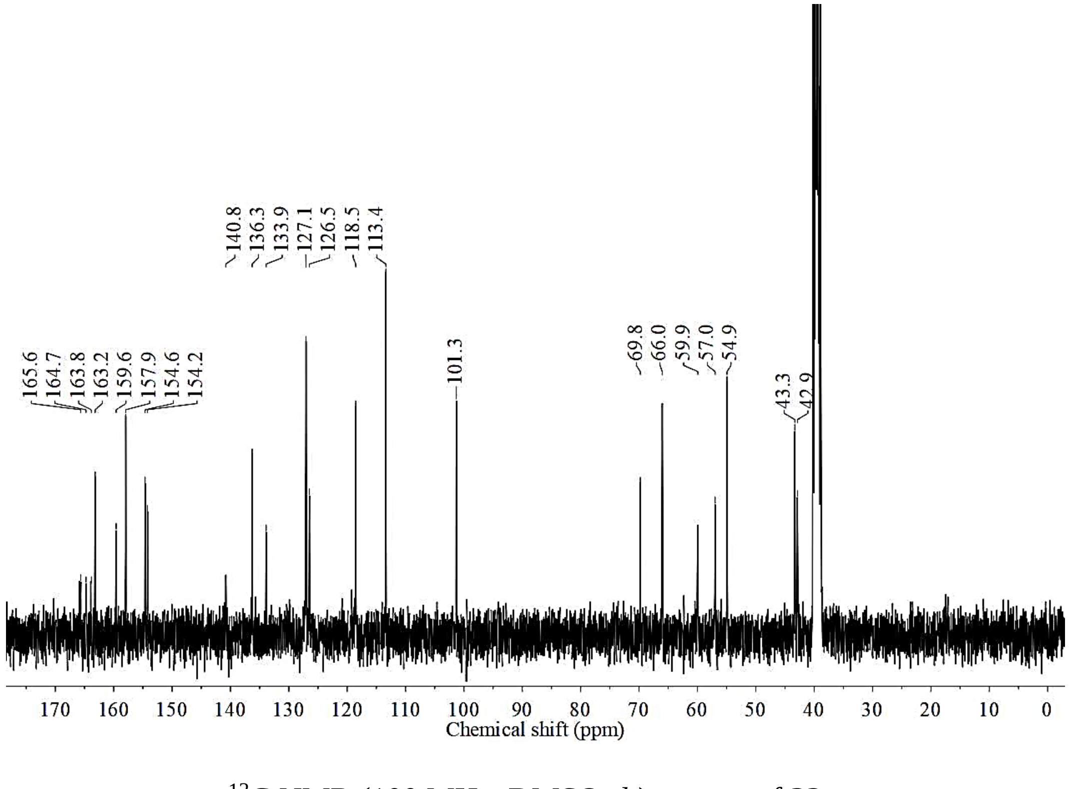

^{13}C NMR (100 MHz, DMSO-d_6) spectra of **32c**.

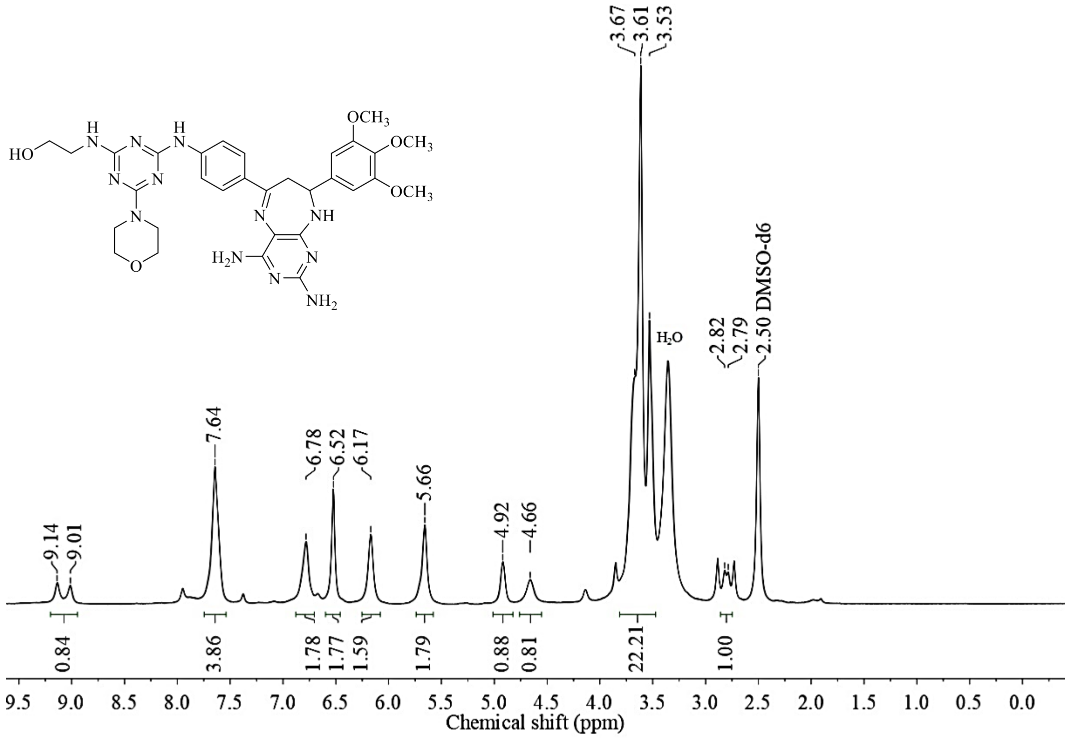

^{1}H NMR (400 MHz, DMSO-d_6) spectra of **32d**.

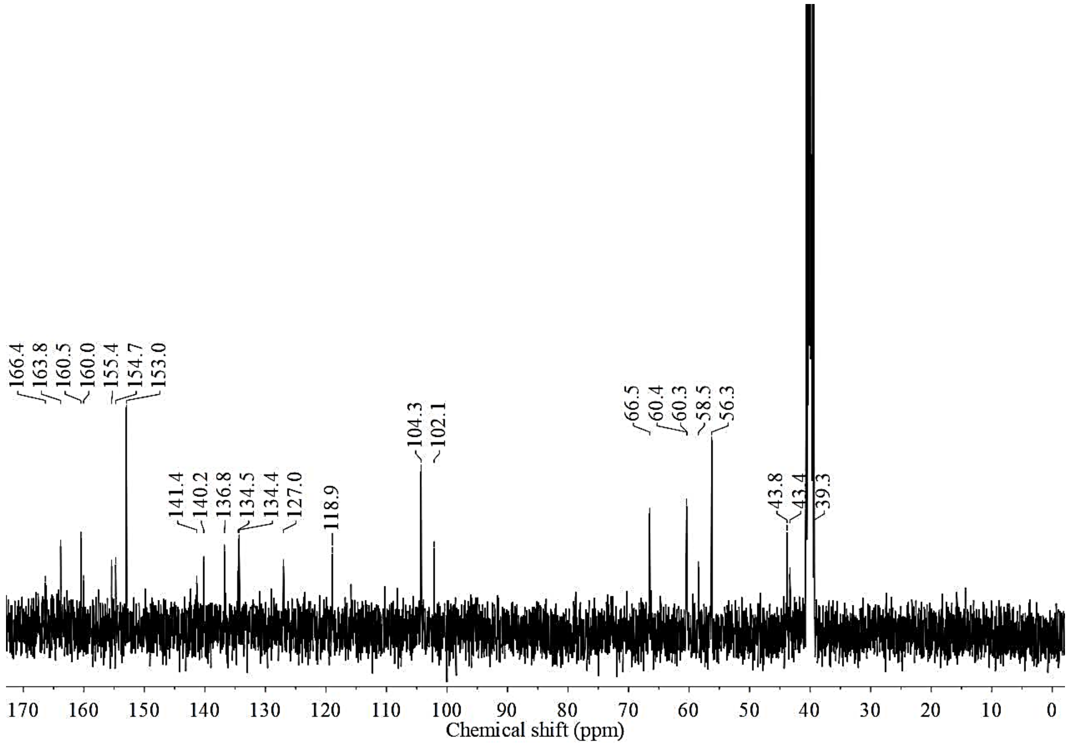

^{13}C NMR (100 MHz, DMSO-d_6) spectra of **32d**.

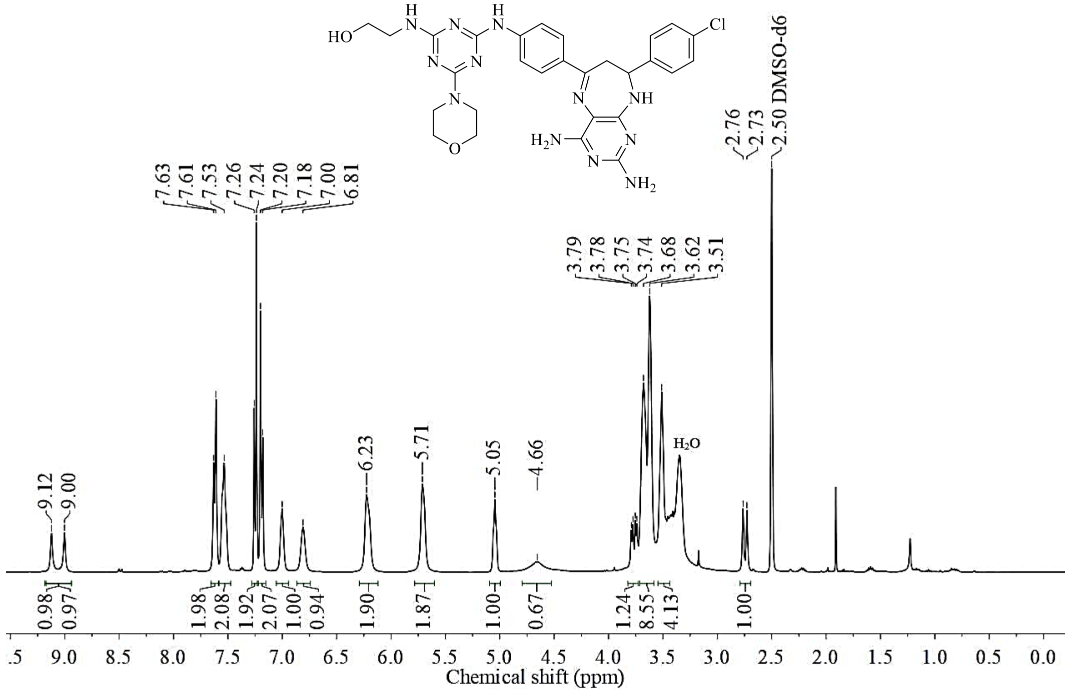

^{1}H NMR (400 MHz, DMSO-d_6) spectra of **32e**.

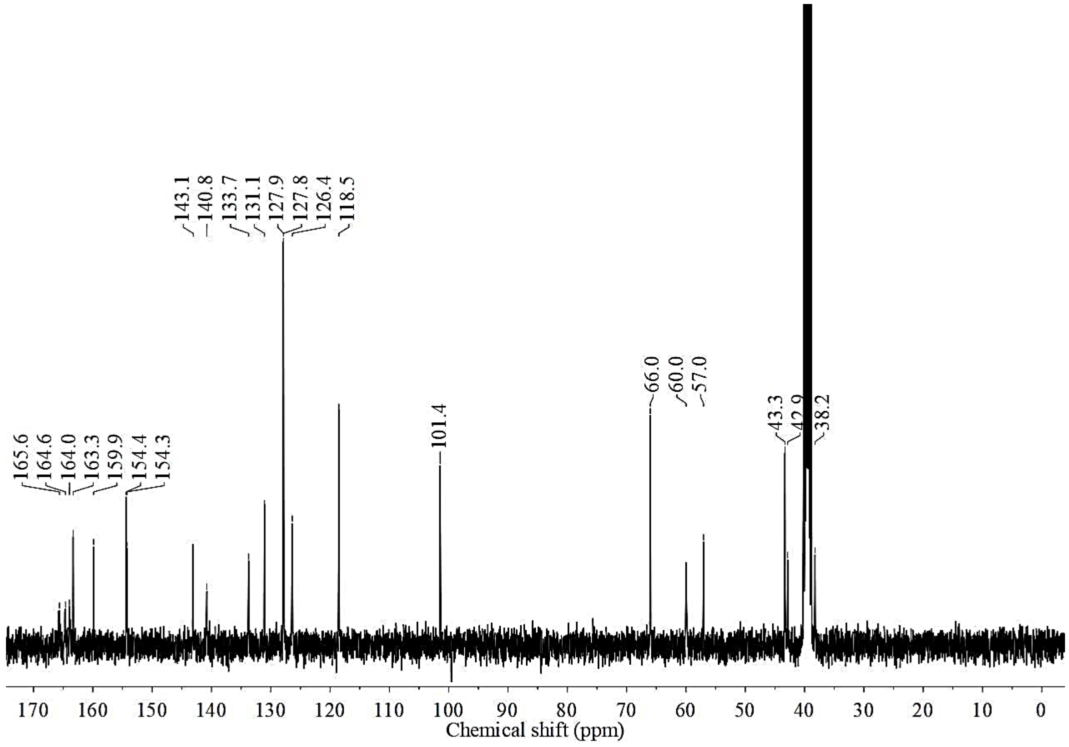

^{13}C NMR (100 MHz, DMSO-d_6) spectra of **32e**.

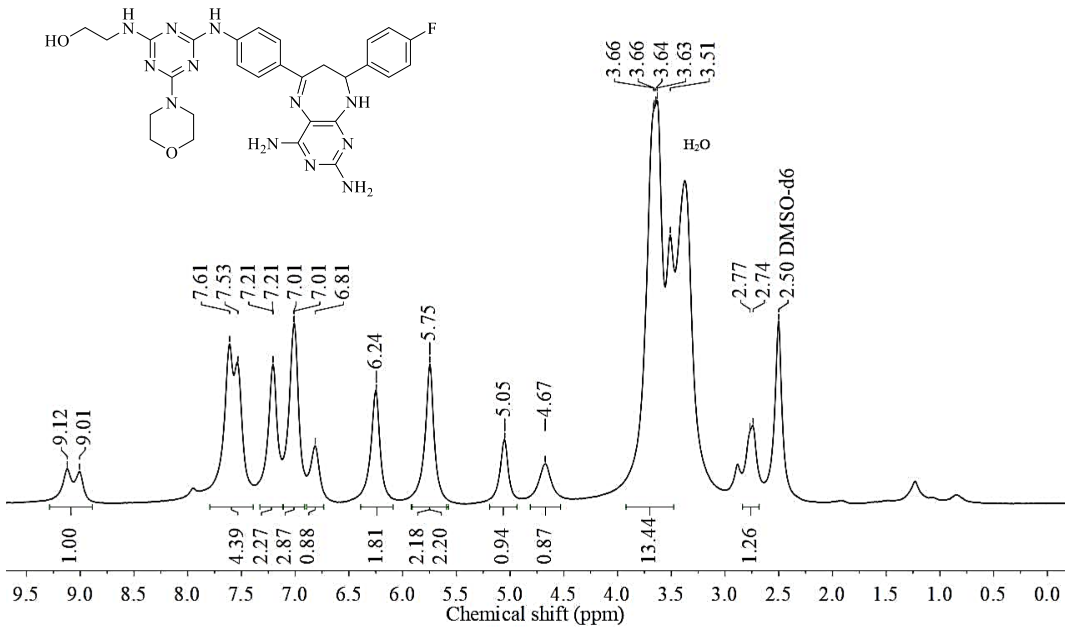

^{1}H NMR (400 MHz, DMSO-d_6) spectra of **32f**.

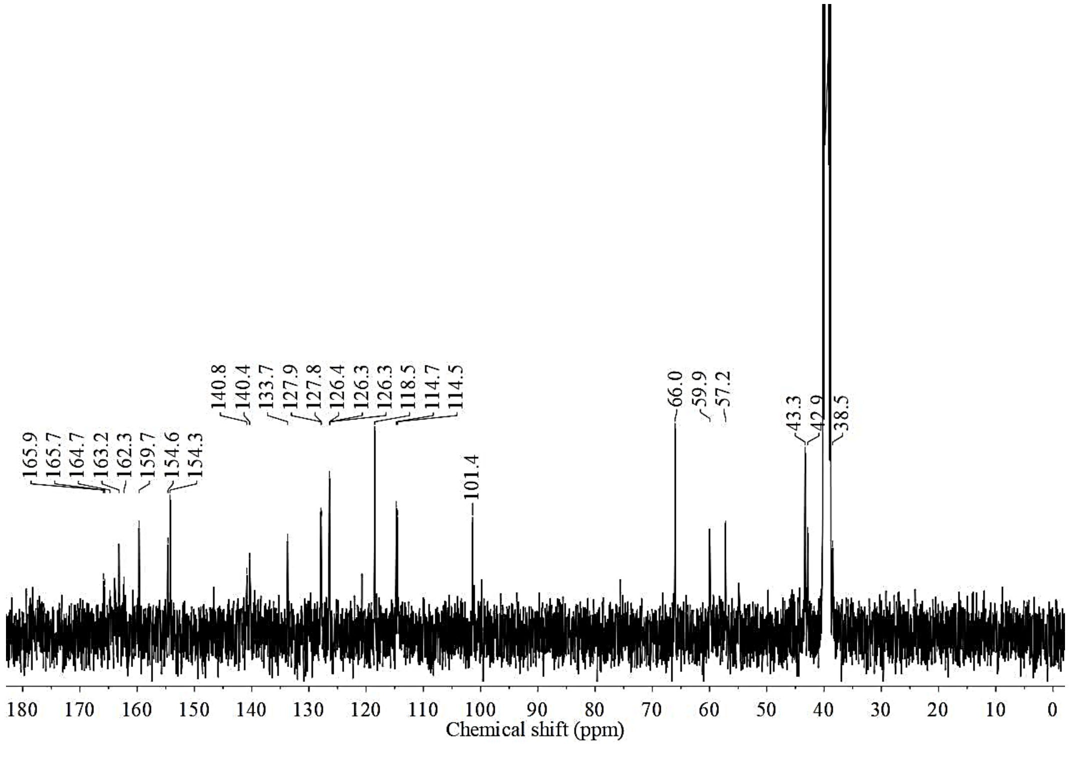

^{13}C NMR (100 MHz, DMSO-d_6) spectra of **32f**.

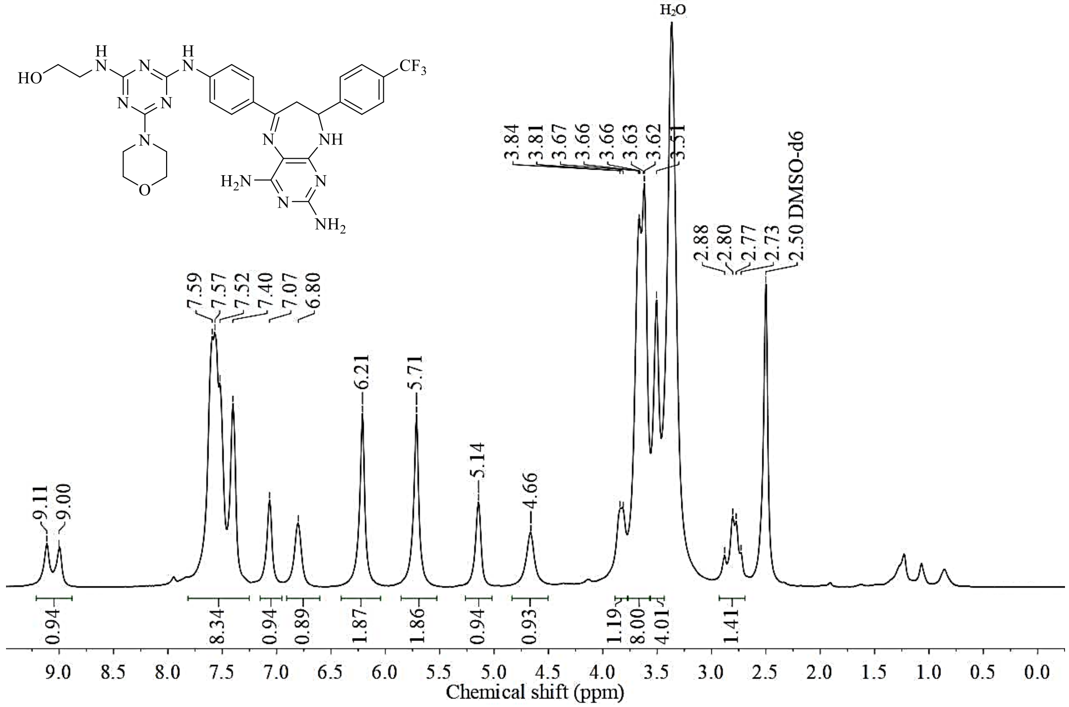

^{1}H NMR (400 MHz, DMSO-d_6) spectra of **32g**.

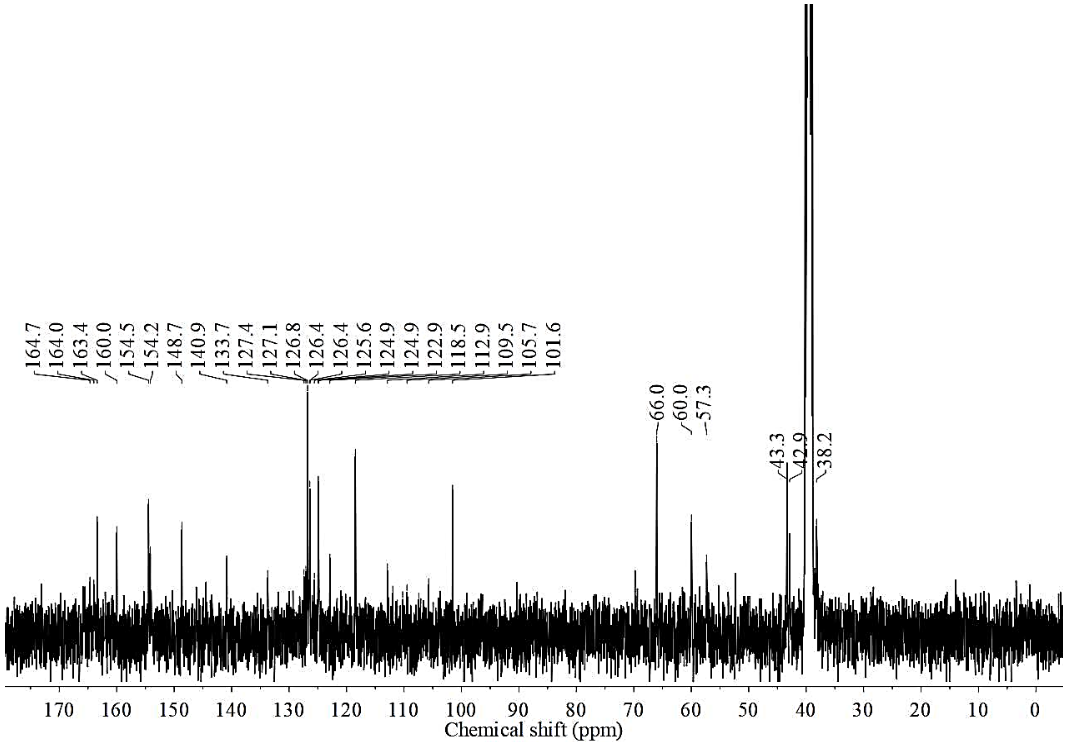

^{13}C NMR (100 MHz, DMSO-d_6) spectra of **32g**.

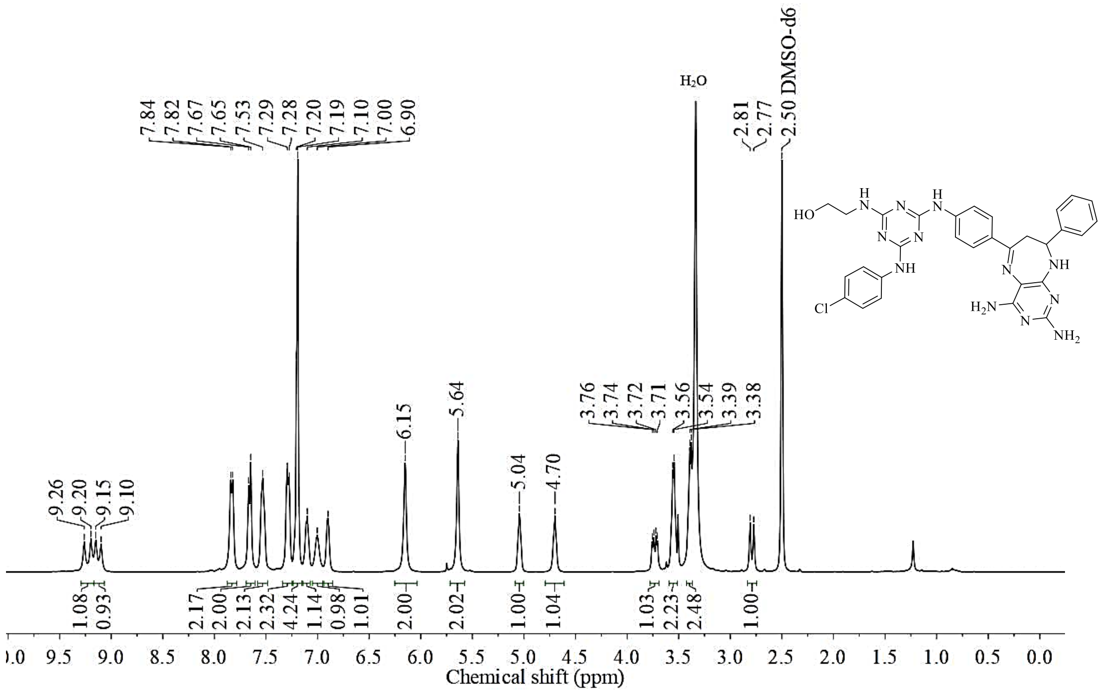

^{1}H NMR (400 MHz, DMSO-*d*6) spectra of **33a**.

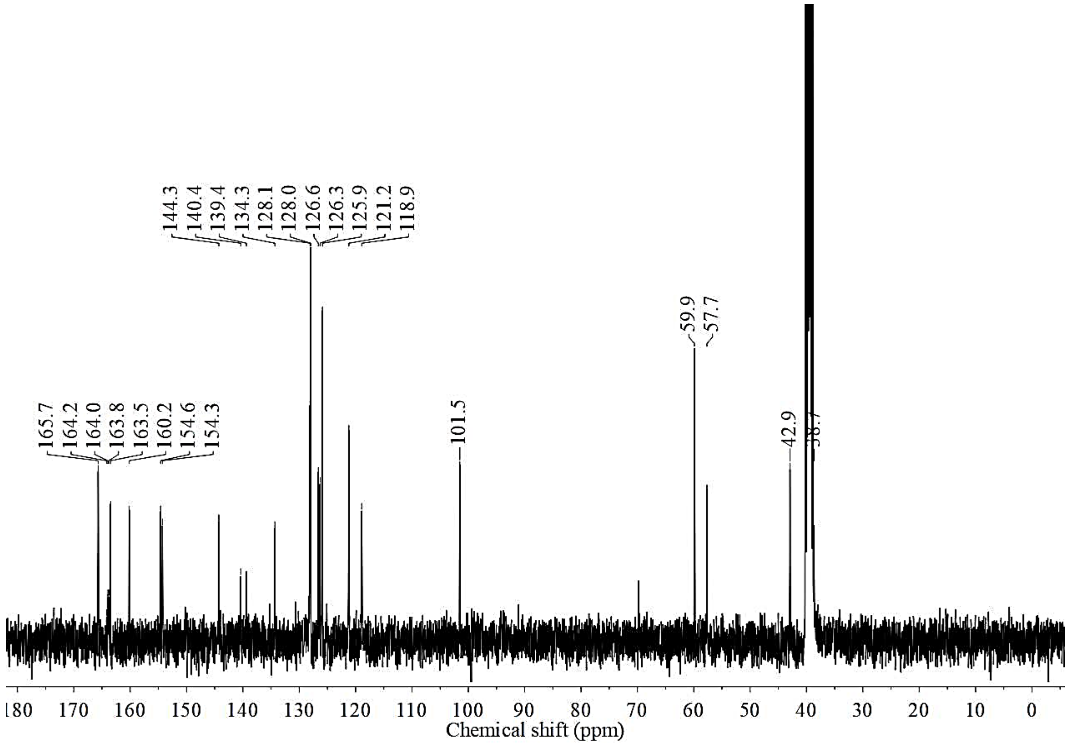

^{13}C NMR (100 MHz, DMSO-*d*6) spectra of **33a**.

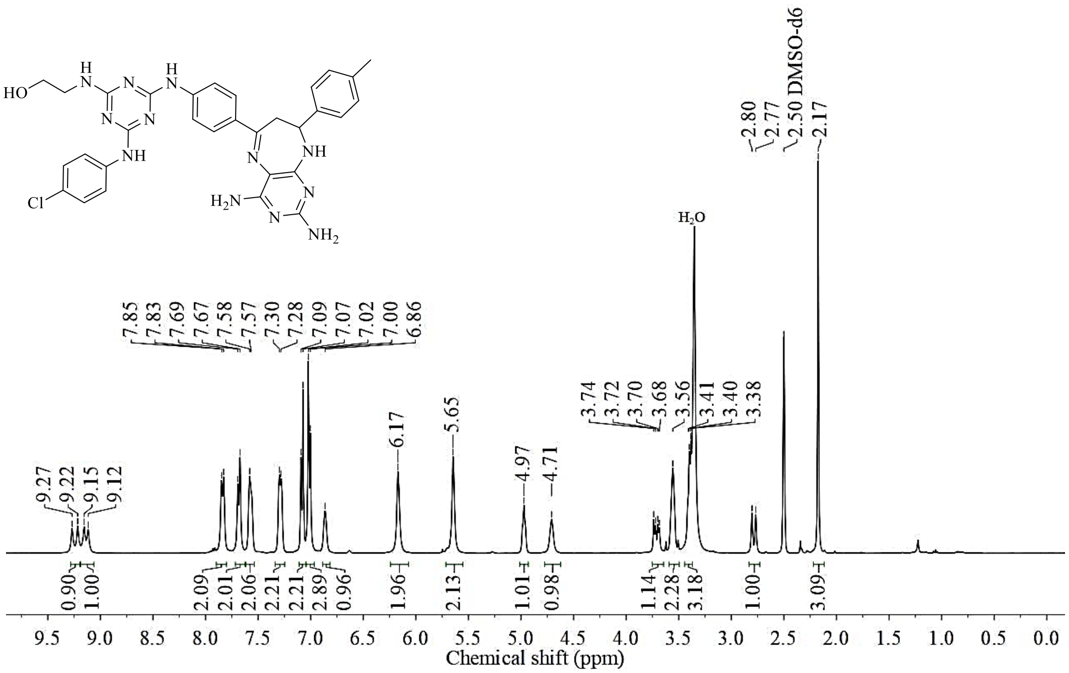

^{1}H NMR (400 MHz, DMSO-d_6) spectra of **33b**.

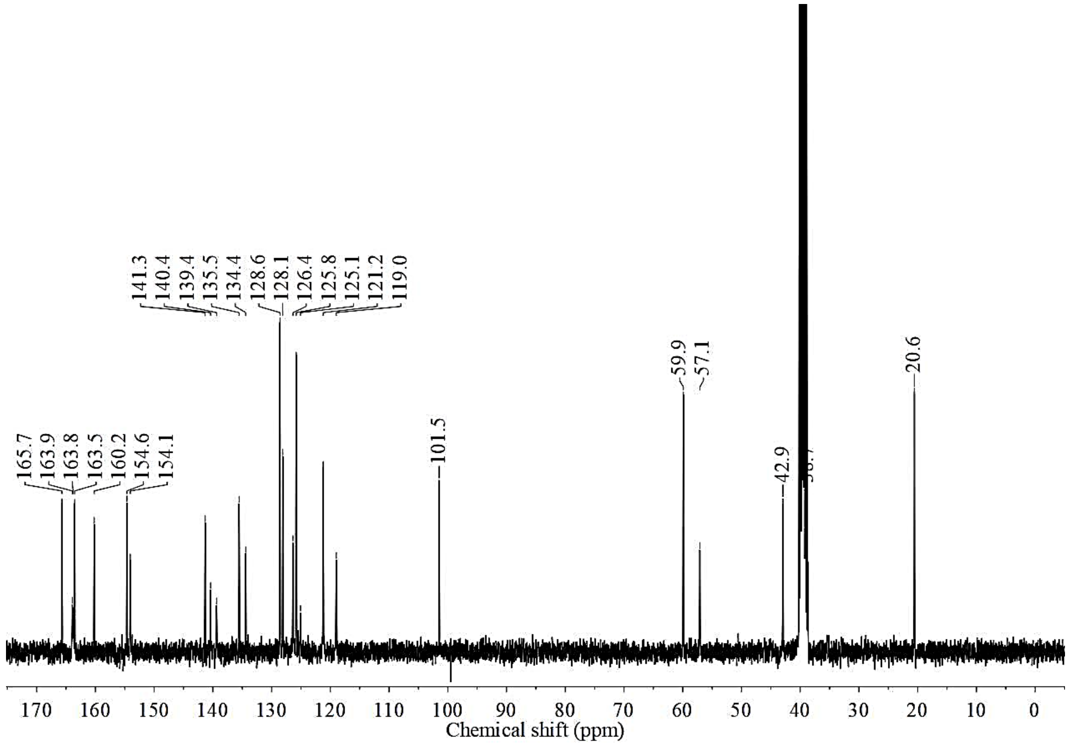

^{13}C NMR (100 MHz, DMSO-d_6) spectra of **33b**.

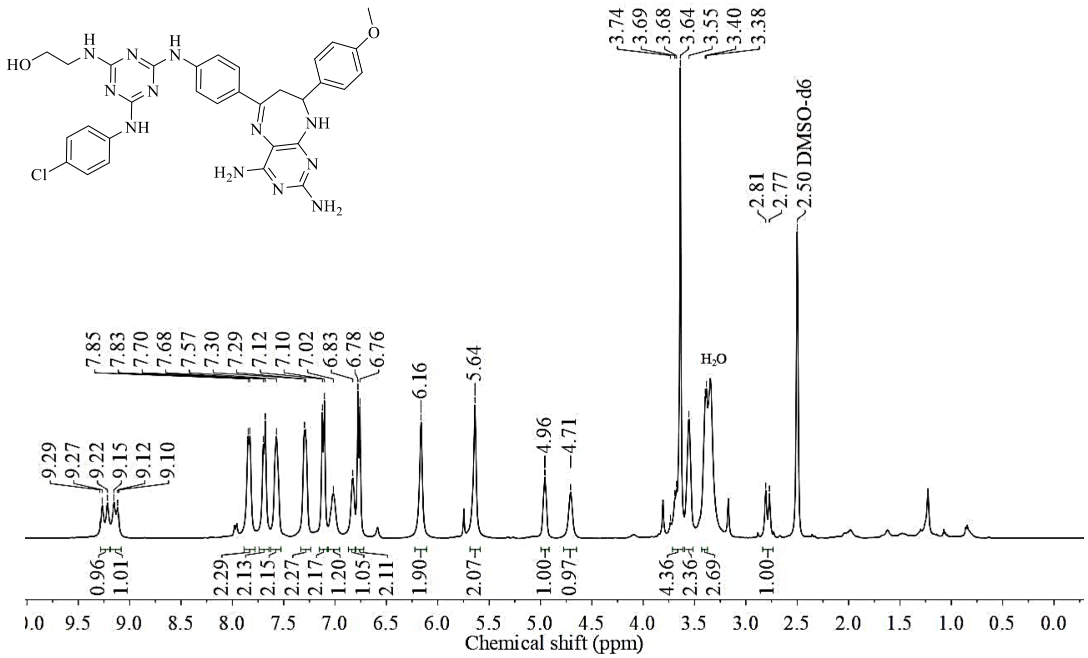

^{1}H NMR (400 MHz, DMSO-d_6) spectra of **33c**.

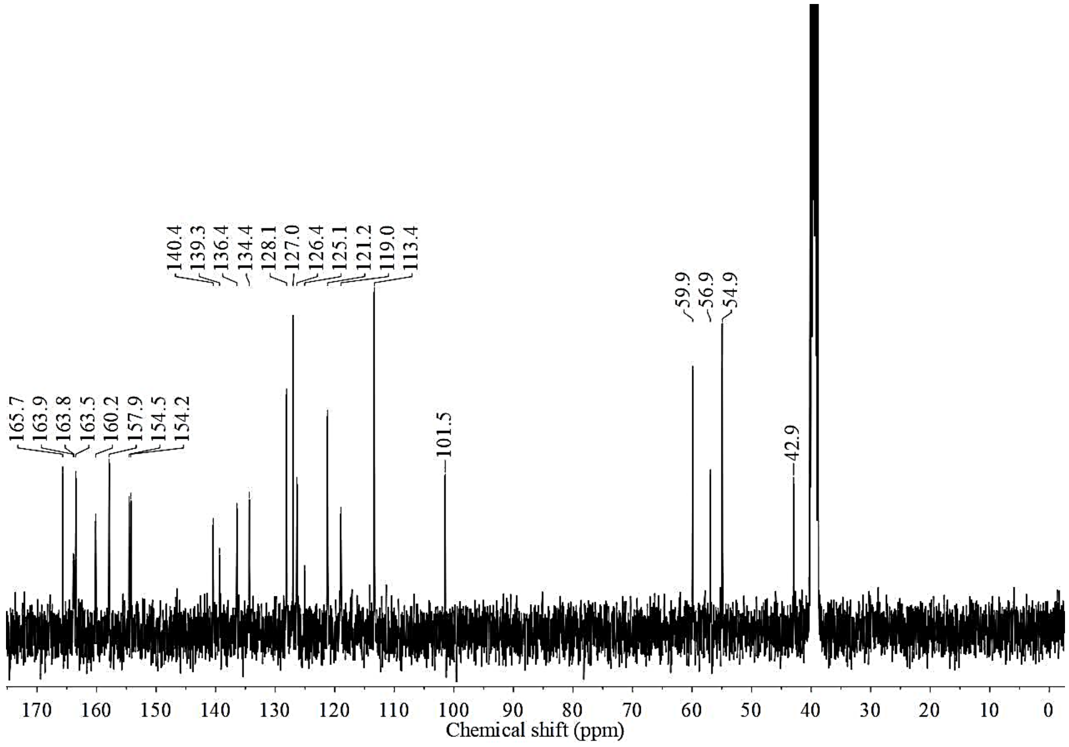

^{13}C NMR (100 MHz, DMSO-d_6) spectra of **33c**.

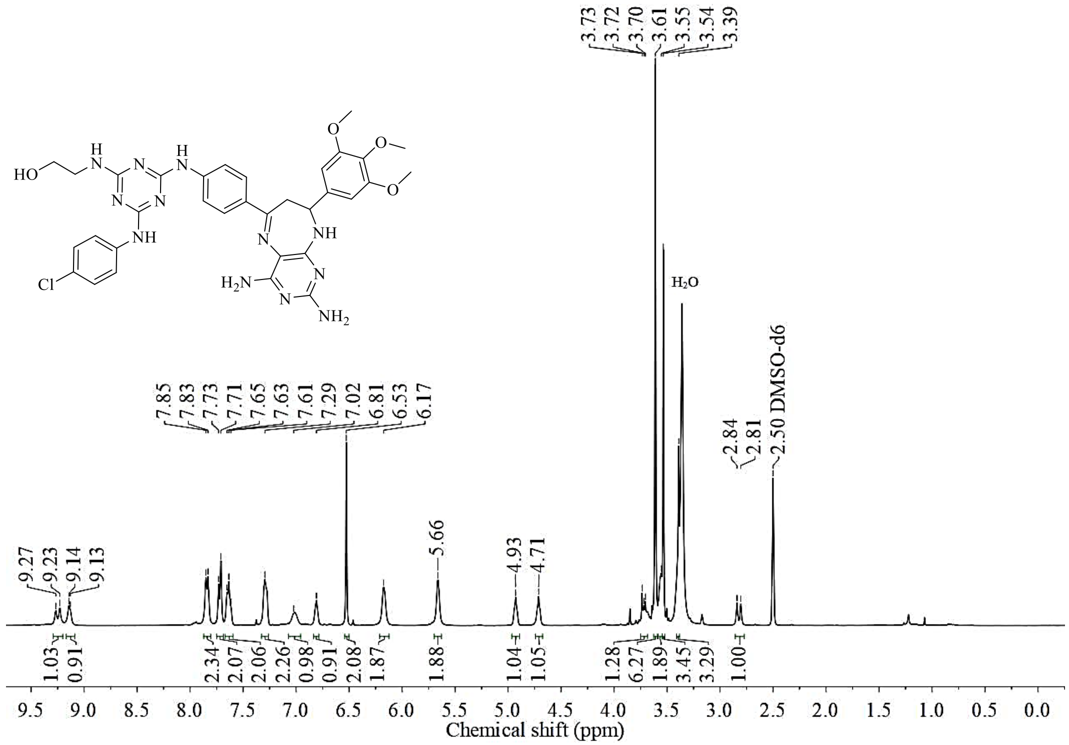

^{1}H NMR (400 MHz, DMSO-d_6) spectra of **33d**.

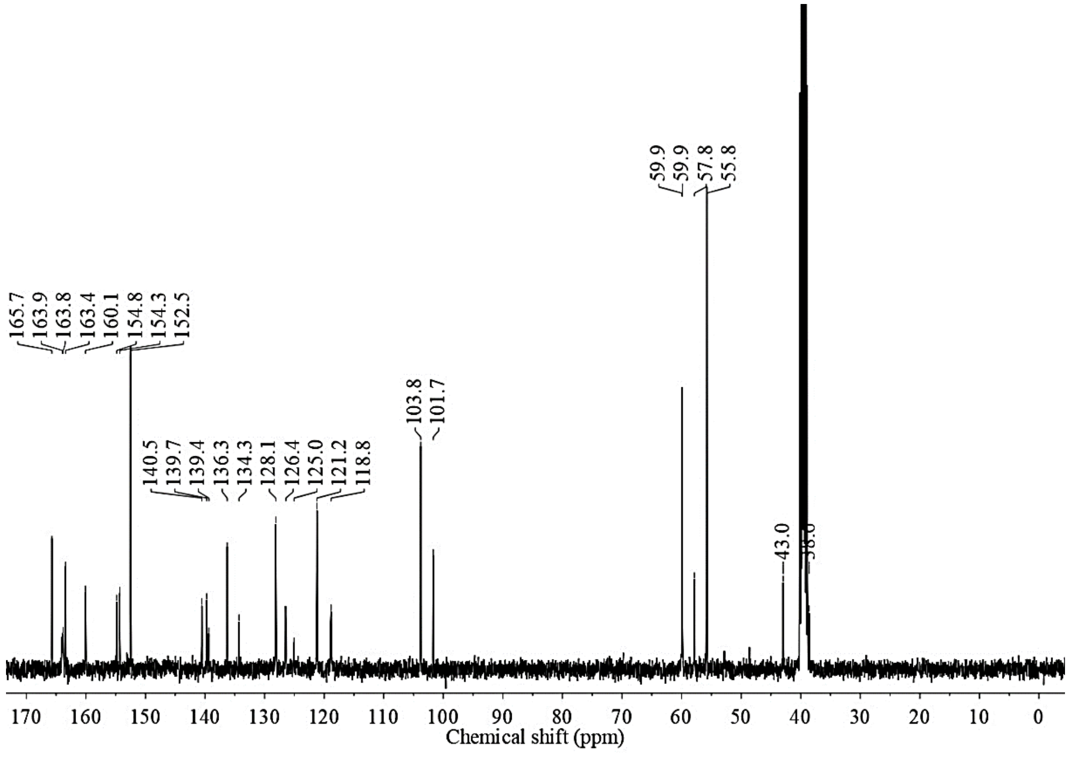

^{13}C NMR (100 MHz, DMSO-d_6) spectra of **33d**.

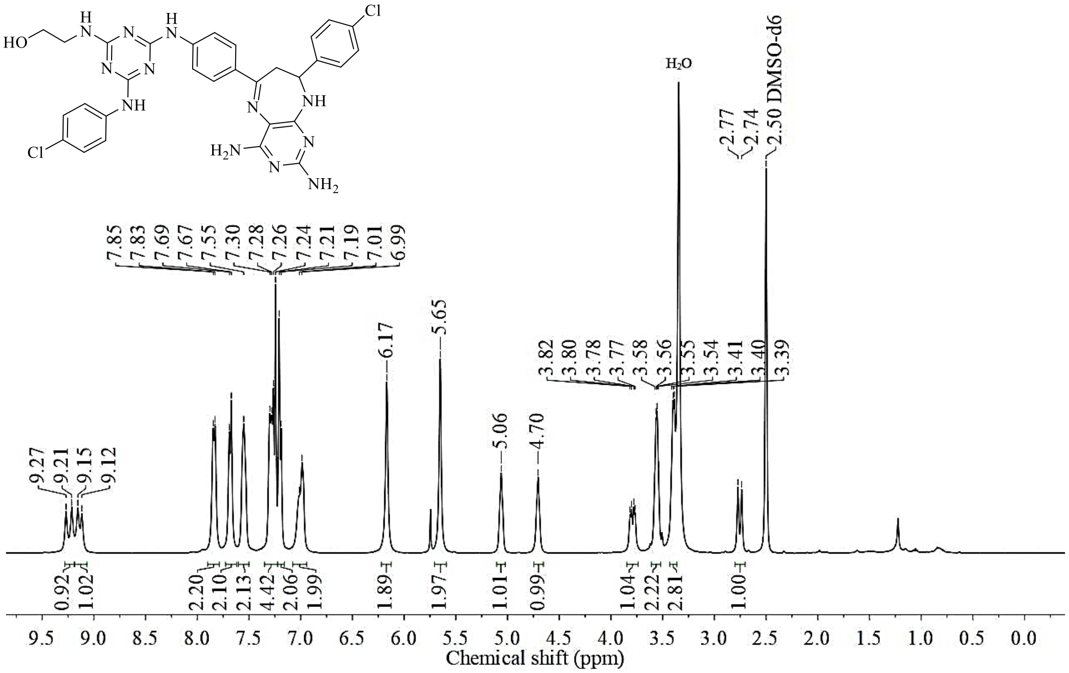

^{1}H NMR (400 MHz, DMSO-d_6) spectra of **33e**.

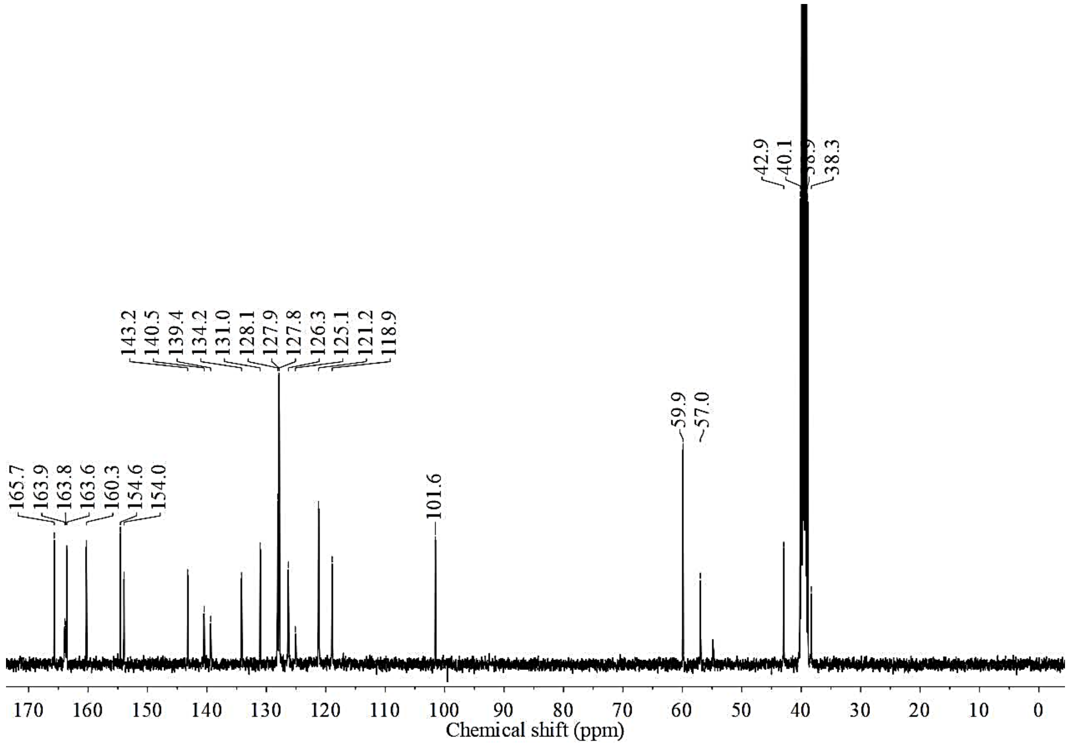

^{13}C NMR (100 MHz, DMSO-d_6) spectra of **33e**.

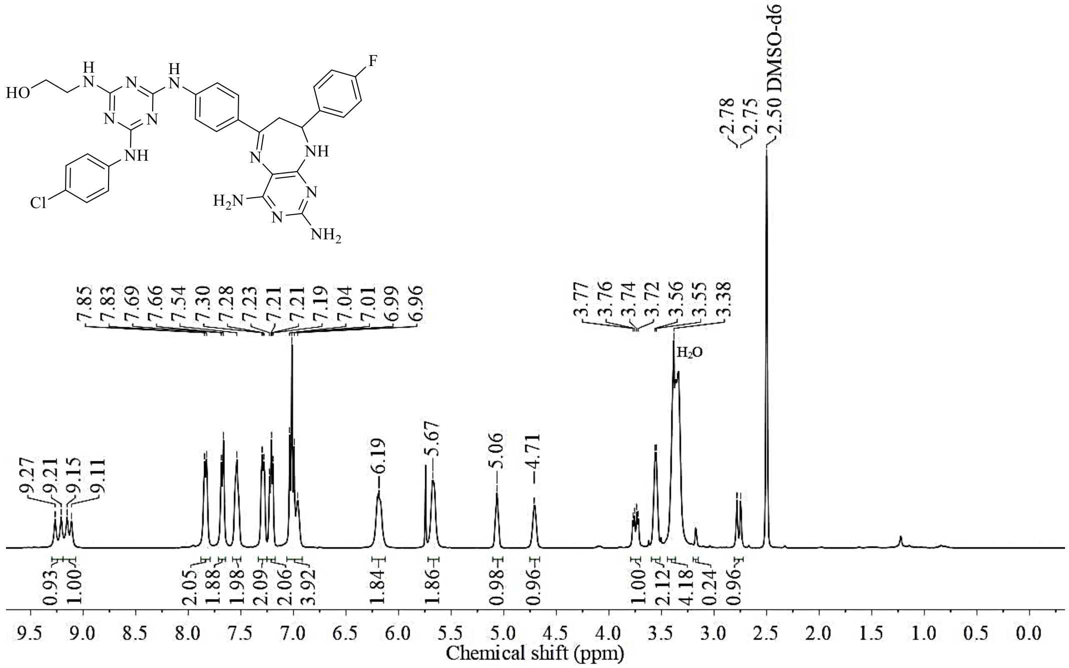

^{1}H NMR (400 MHz, DMSO-d_6) spectra of **33f**.

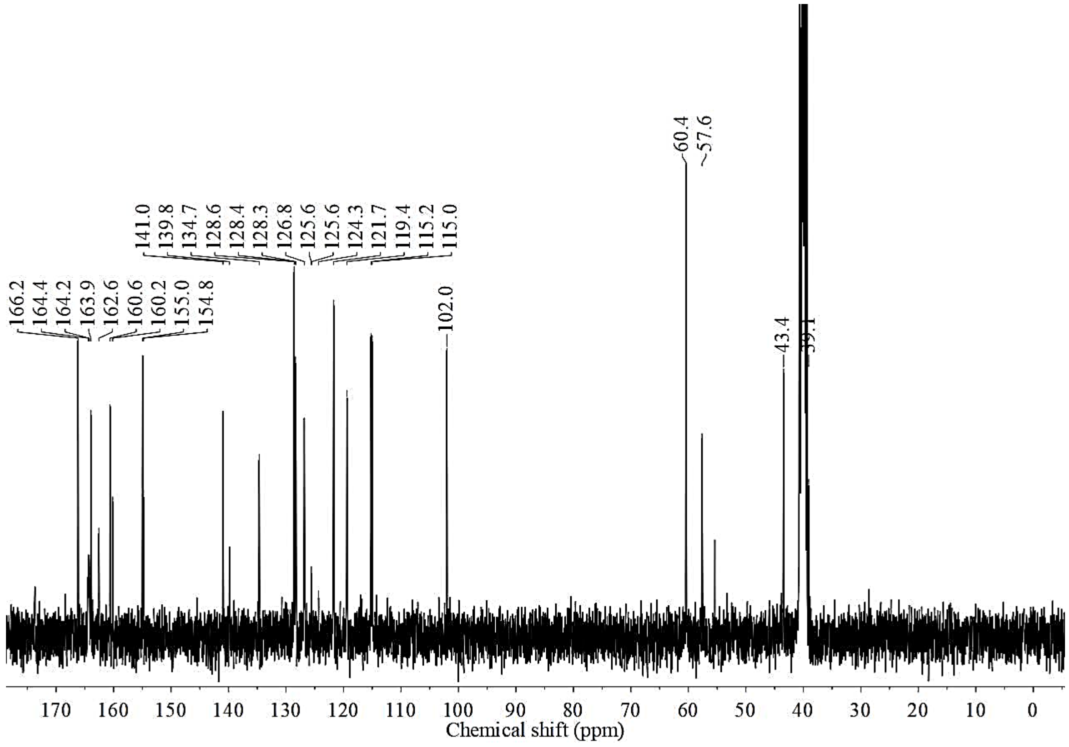

^{13}C NMR (100 MHz, DMSO-d_6) spectra of **33f**.

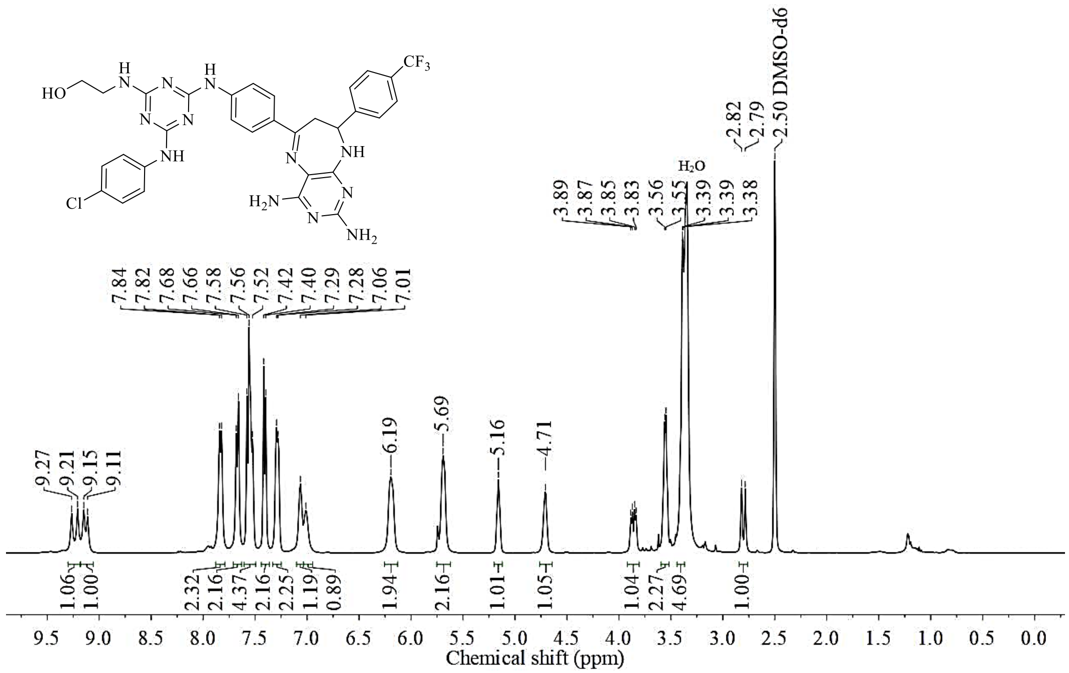

^{1}H NMR (400 MHz, DMSO-d_6) spectra of **33g**.

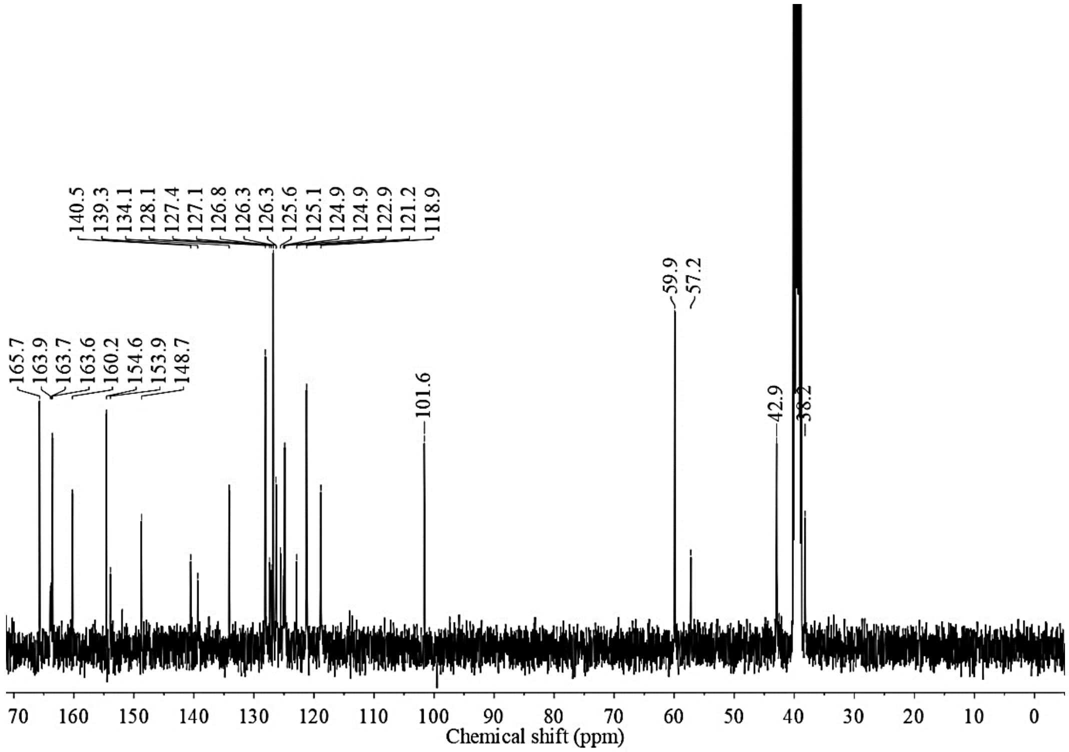

^{13}C NMR (100 MHz, DMSO-d_6) spectra of **33g**.

In vitro anticancer activity expressed as GI_{50} and LC_{50} (μM)

Panel	Cell line	20b NSC: D-832371/1		20d NSC: D-832525/1		21a NSC: D-833552/1		21b NSC: D-833553/1		21d NSC: D-833555/1	
		GI_{50}	LC_{50}	GI_{50}	LC_{50}	GI_{50}	LC_{50}	GI_{50}	LC_{50}	GI_{50}	LC_{50}
Leukemia	CCRF-CEM	2.71	>100	2.38	>100	3.38	>100	3.92	>100	2.19	>100
	HL-60(TB)	2.07	>100	1.54	>100	8.37	>100	19.1	>100	2.21	>100
	K-562	1.53	>100	1.91	>100	3.57	>100	4.2	>100	1.4	>100
	MOLT-4	2.09	>100	1.98	>100	2.56	>100	2.8	>100	0.472	>100
	RPMI-8226	1.72	>100	1.63	>100	2.83	>100	3	>100	0.751	>100
	SR	1.06	>100	0.784	>100	2.9	>100	3.25	>100	0.7	>100
Non-Small Cell Lung Cancer	A549/ATCC	9.47	>100	2.78	42.2	11.7	>100	45.8	>100	4.49	54.7
	EKVX	3.90	>100	2.81	36.6	4.73	>100	6.71	>100	2.15	31.1
	HOP-62	16.60	>100	2.35	22.4	26.6	>100	32.2	>100	1.83	8.22
	HOP-92	8.40	>100	2.75	35.4	2.08	>100	4.57	>100	1.67	50.9
	NCI-H226	6.28	>100	3.69	>100	4.94	64.9	5.22	49.1	1.44	7.24
	NCI-H23			1.99	17.5	12.6	>100	5.52	>100	1.63	8.08
	NCI-H322M	6.24	>100	2.91	29.3	28.7	>100	51.9	>100	2.99	>100
	NCI-H460	2.20	>100	1.87	8.69	9.14	93.2	12.1	>100	1.45	7.53
	NCI-H522	1.56		1.56	6.43	5.15	>100	9.76	>100	1.47	7.11
Colon cancer	COLO 205	2.64	>100	2.1	9.18	15.1	>100	41.7	>100	4.45	47.5
	HCC-2998	2.08		2.23	7.97	12.9	>100	20.5	>100	2.03	8.52
	HCT-116	1.73		1.53	6.78	3.21	45.5	2.28	24.4	1.4	7.24
	HCT-15	1.78	>100	1.6	10.2	3.31	36.2	4.73	43.4	1.18	6.39
	HT29	1.85		2.83	32.9	4.36	59.7	8.15	>100	1.54	8.22
	KM12	1.85		2.99	>100	14.9	>100	19.3	>100	3.63	>100
	SW-620	1.64	>100	2.24	26.4	1.81	8.7	2.36	20.1	1.55	6.89
CNS Cancer	SF-268	2.90	>100	3.02	71.8	12.2	>100	13.9	>100	3.43	>100
	SF-295		>100	2.9	35.2	6.89	>100	8.61	>100	1.98	19.6
	SF-539	1.88		1.88	13	13.8	51.7	7.64	44.9	1.64	5.81
	SNB-19	2.45	>100	2.31	29.9	10.2	74.9	11.1	67.3	1.86	18.5
	SNB-75	>100	>100	1.79	24.2	4.7	>100	15.4	>100	2.54	81.8
	U251	1.62		1.62	6.02	2.84	60.6	3.32	>100	0.503	9.59
Melanoma	LOX IMVI	0.43	4.09	0.642	4.99	1.6	6.23	1.84	6.69	0.478	4.11
	MALME-3M	2.29	>100	2.67	24.3	15.9	98.8	15.4	72.3	1.75	11.1
	M14	2.03	>100	2.38	37.9	13	>100	14.7	>100	2.83	32.8
	MDA-MB-435	3.31	>100	3.1	35.1	7.39	94.3	11.4	70.4	1.63	7.69
	SK-MEL-2	8.17	>100	2.76	33.1	4.68	63.1	8.83	>100	1.69	8.47
	SK-MEL-28	2.50	>100	1.92	7.53	12.2	84.6	12.3	79.6	1.8	9.23
	SK-MEL-5	2.00		1.77	6.19	6.21	>100	5.14	78.5	2.16	12.2
	UACC-257	3.98	>100	2.83	36.7	6.44	>100	8.76	>100	2.83	>100
	UACC-62	2.01		1.69	6.03	4.99	79.4	10.1	>100	1.41	6.38

Ovarian Cancer	IGROV1	3.10	>100	2.45	26.2	18.7	>100	13.8	99.1	1.93	12.2
	OVCAR-3	2.49	>100	3.2	34.4	11.8	>100	13	>100	2.57	>100
	OVCAR-4	3.92	>100	2.92	33.4	5.85	>100	7.94	>100	2.07	>100
	OVCAR-5	3.22	>100	3.14	38.4	13.9	>100	16.5	>100	1.48	11.6
	OVCAR-8	1.76		2.56	34.7	5.9	97.6	7.19	>100	1.55	>100
	NCI/ADR-RES	2.08	>100	4.72	95.9	7.04	58.5	5.97	>100	1.42	8.08
	SK-OV-3	23.00	>100	2.44	22	48.6	>100	48.9	>100	8.42	97.4
Renal cancer	786-0	2.18	>100	3.46	43.6	20.5	>100	13.3	>100	1.45	7.19
	A498	>100	>100	3.58	37	27	>100	41.8	>100	3.99	38.9
	ACHN	1.94		1.64	7.9	8.29	>100	9.58	96.5	2.3	50.6
	CAKI-1	5.02	>100	2.43	30.4	5.72	>100	6.43	>100	1.98	25.1
	RXF 393	2.07		1.75	6.94						
	SN12C	2.35	>100	1.79	23.3	7.32	54.5	3.89	73	1.23	6.04
	TK-10	>100	>100	3.12	30.6	18	>100	18.2	>100	2.53	38.1
	UO-31	1.49		1.66	6.5	4.4	>100	4.2	>100	1.12	4.96
Prostate Cancer	PC-3	2.53	>100	3.11	43.2	10	>100	8.85	>100	2.77	>100
	DU-145	3.79	>100	2.99	35.3	27.3	>100	25.8	>100	3.99	67.9
Breast Cancer	MCF7	1.50	>100	1.72	61.5	3.08	78.2	3.57	>100	1.06	67
	MDA-MB-231/ATCC	2.98	>100	2.86	35.4	6.92	56.1	5.24	50.8	1.48	8.63
	HS 578T	4.60	>100	3.62	>100	5.44	>100	7.24	>100	2.39	>100
	BT-549	1.62		1.6	6.88	12	68.1	6.27	91.3	1.56	6.56
	T-47D	2.98	>100	3.12	39.9	3.36	>100	3.57	>100	1.87	>100
	MDA-MB-468	1.98		1.71	14.3	3.21	>100	4.64	>100	1.35	9.11

**Panel**	**Cell line**	**23a NSC: D-837230/1**		**23d NSC: D-837233/1**		**23e NSC: D-837676/1**		**23f NSC: D- 837677/1**		**23g NSC: D-837678/1**	
		GI_{50}	LC_{50}	GI_{50}	LC_{50}	GI_{50}	LC_{50}	GI_{50}	LC_{50}	GI_{50}	LC_{50}
Leukemia	CCRF-CEM	4.41	>100	2.07	>100	6.86	>100	6.58	>100	3.2	>100
	HL-60(TB)	6.18	>100	1.97	>100	14.7	>100	14.7	>100	5.12	>100
	K-562	2.19	>100	1.46	>100	3.71	>100	3.7	>100	3.21	>100
	MOLT-4	4.27	>100	2.23	>100	3.38	>100	4.69	>100	3.48	>100
	RPMI-8226	3.25	>100	1.39	>100	2.55	>100	1.91	>100	1.77	>100
	SR	3.2	>100	1.88	>100	2.52	>100	3.45	>100	6.31	>100
Non-Small Cell Lung Cancer	A549/ATCC	13.6	59.1	3.55	43.9	14.4	56.4	13.7	56.8	13.8	62.3
	EKVX	14.7	63.8	3.74	82.3	14.8	55.7	14.4	53.8	14.3	52.5
	HOP-62	16.4	62.7	2.66	32.1	17.5	65.8	16.1	61.8	17	61.3
	HOP-92	12.2	58	7.27	76	15.1	67.8	12.7	63.5	15.2	65.2
	NCI-H226	13.5	74.2	11.4	72.4	15.7	73.8	12.3	64.7	16.1	78.9
	NCI-H23	16	58	3.09	40.3	15.7	55.6	14.6	54.3	13	52.6
	NCI-H322M	14.8	56.5	7.82	48.5	14.4	52.4	13.2	50.9	14.2	52.1
	NCI-H460	18.2	80.7	3.17	59	16.8	67.1	15.7	69.4	16.1	65.6
	NCI-H522	13.6	54.1	1.66	6.37	14	53.3	13.3	53.2	15.2	58.2

Colon cancer	COLO 205	20.2	80.7	2.14	16.6	16.7	70.8	14.6	62.4	15	69.8
	HCC-2998	16.7	57.7	1.8	5.9	16.7	56.7	16	56	12.9	52.5
	HCT-116	1.82	7.1	1.56	5.92	11.7	50.7	2.29	16.8	3.19	33.5
	HCT-15	5.21	61.7	1.82	8.38	2.07	9.93	2.15	13.7	1.86	7.07
	HT29	3.8	43.9	1.72	5.93	3.21	36.4	2.84	32.3	2.06	9.88
	KM12	2	8.93	1.7	6.52	16.9	>100	13.4	70.3	8.95	68.3
	SW-620	3.41	42.7	1.9	7.42	8.46	50.2	3.38	40.8	3.3	36.5
CNS Cancer	SF-268	13.7	70.1	3.16	77.2	17.7	>100	15	94	16.3	87.9
	SF-295	15.6	59.5	3.04	57.8	13.3	52.8	11.3	50.1	8.11	45.4
	SF-539	11.1	53.2	1.81	6.29	14.7	52.9	13.2	51.1	13	50.7
	SNB-19	14.8	57.3	4.28	41.7	14.2	52.1	13.4	52.1	13.5	51.3
	SNB-75	5.49	44.6	1.56	41.6						
	U251	11.7	57.2	1.54	5.54	8.16	45.9	4.74	41.5	2.95	40.7
Melanoma	LOX IMVI	1.97	8.68	1.64	5.76	1.71	6.13	1.63	6.19	1.68	5.94
	MALME-3M	15.2	56.5	1.97	9.42	15.1	54.2	14.6	54.4	14.4	53
	M14	14.6	68.5	2.18	26.9	14.1	56.9	15	62.2	9.83	49.5
	MDA-MB-435	14.7	54.4	1.9	8.46	12.8	55.3	11.6	51.9	10.3	51.1
	SK-MEL-2	15.3	56.9	10.7	48.9	16.5	56	13.7	52.7	16.3	56.7
	SK-MEL-28	17.2	63.9	2.12	13	15.5	53.9	14.9	53.4	14.4	52.6
	SK-MEL-5	15.9	54.3	7.9	46.6	16.3	55.6	15.7	54.4	15.8	54.9
	UACC-257	17.9	58.3	5.86	43	16.2	55.7	15.5	54.9	15.3	56.5
	UACC-62	12	52.3	2.14	22.8	11.4	49.1	10.8	49.1	11.2	48.5
Ovarian Cancer	IGROV1	15.3	66.4	2.6	50.1	3.08	36.8	2.27	25.4	1.77	6.97
	OVCAR-3	15.3	53.4	2.02	8.84	17.9	70.5	14.6	55.7	16.4	57.3
	OVCAR-4	14.3	54	2.21	24.8	14.7	57.2	11.9	51.1	14.8	56.5
	OVCAR-5	12.1	55.9	2.83	33.7	15	53.9	13.6	52.6	14.5	53
	OVCAR-8	16.4	61.7	2.3	36.5	14.8	54.5	14.7	54	14.4	54.9
	NCI/ADR-RES										
	SK-OV-3	15.6	56.8	14.3	52.7	15.3	59.5	13.8	55.5	14.7	59
Renal cancer	786-0	14.8	57.1	1.89	7.18	14.8	55.9	14.5	57	14.5	54.9
	A498	14.6	52.9	14.2	52.6	12.4	50.8	11.9	49.9	13.3	53
	ACHN	18.2	72.2	1.78	6.75	13.5	51.3	13.1	51.4	8.6	45.5
	CAKI-1	12.2	49.9	10.6	51	11.2	54.7	9.5	48.6	11.4	51.7
	RXF 393	7.87	55.8	1.22	8.63	12.8	57.1	8.82	46.5	11.5	49
	SN12C	14.6	56.1	2.72	38.1	14.4	53.8	12	50	12.5	50
	TK-10	16.4	55.5	13.1	50.8	15.8	54.7	15.3	53.8	15.5	56
	UO-31	11.2	55.7	1.57	6.32	11.4	48.4	11	47.9	11.9	49.2
Prostate Cancer	PC-3	8.63	58.9	3.76	>100	13	67	11.2	58.5	10.8	60
	DU-145	15.1	53.3	3.31	34.1	17.2	67.3	14.4	53.8	15.4	54.5
Breast Cancer	MCF7	3.49	62	1.64	9.66	2.37	34.6	2.99	46.7	1.68	6.63
	MDA-MB-231/ATCC	13.9	54.7	2.9	36.7	13.3	52.7	12.3	51.8	11.7	50.3
	HS 578T	17.1	>100	2.29	>100	15.8	>100	14.6	>100	17.8	>100
	BT-549	11.2	50	2.01	6.91	15.3	56	15.1	56.7	15.6	54.9

	T-47D	16	87.4	5.74	71.5	16.7	>100	11.9	94.2	5.5	>100
	MDA-MB-468	4.17	49.9	1.69	6.66	11	56	8.08	54.7	5.11	48.8

**Panel**	**Cell line**	**24a NSC: D-835919/1**		**24b NSC: D-835920/1**		**24c NSC: D-835921/1**		**24d NSC: D-835922/1**		**24e NSC: D-836365/1**	
		GI_{50}	LC_{50}	GI_{50}	LC_{50}	GI_{50}	LC_{50}	GI_{50}	LC_{50}	GI_{50}	LC_{50}
Leukemia	CCRF-CEM	6.59	>100	5.28	>100	4.09	>100	3.62	>100	3.75	>100
	HL-60(TB)	3.88	>100	5.58	>100	3.6	>100	2.64	>100	4.39	>100
	K-562	3.34	>100	3.89	>100	3.99	>100	2.73	>100	3.29	>100
	MOLT-4	3.22	>100	3.86	>100	3.12	>100	2.74	>100	3.07	>100
	RPMI-8226	3.04	>100	2.73	>100	2.96	>100	2.5	>100	2.47	7.11
	SR	2.58	>100	2.76	>100	2.97	>100	2.42	>100		
Non-Small Cell Lung Cancer	A549/ATCC	8.33	73.5	10.5	77.7	6.54	>100	12.2	78.6	4.52	17.4
	EKVX	4.48	40.6	4.96	43.3	4.12	>100	4.85	42.2	3.11	14.7
	HOP-62	11.8	60.6	15.3	64.8	17.1	>100	14.1	59.9	12.5	27.5
	HOP-92	8.1	60.1	7.63	54	3.25	>100	11.5	>100	3.83	14.7
	NCI-H226	5.22	57.3	10.4	63.9	3.8	>100	4.47	>100	6.14	24.3
	NCI-H23	10.1	50	11.3	50	10.4	>100	3.03	34.9	10.7	23.4
	NCI-H322M	9.83	76.9	11.8	62.5	10.2	>100	10.1	>100	9.36	24.9
	NCI-H460	4.34	56.5	4.22	52.3	4.11	>100	5.12	50.6	3.38	13.8
	NCI-H522	5.47	53.6	5.24	45.3	2.55	67.1	6.45	50	7.1	22
Colon cancer	COLO 205	5.46	56.7	6.51	55.1	3.46	45.7	4.78	86.1	5.96	18.7
	HCC-2998	2.35	17.9	4.5	41	5.47	43.2	1.62	5.52	2.48	6.9
	HCT-116	1.72	6.1	1.75	6.17	1.86	7.07	1.77	7.66	1.66	3.11
	HCT-15	2.04	7.25	3.92	35.3	2.71	27.4	1.8	6.57	4.62	16.9
	HT29	3.08	73.5	2.38	16.2	1.83	7.63	1.93	6.46	2.15	5.72
	KM12	3.74	54	5.41	99.7	4.01	72	2.07		2.97	9.1
	SW-620	5.56	54.3	7.53	49.4	5.42	52.3	1.95	7.43	2.78	11
CNS Cancer	SF-268	5.65	65.2	8.76	90.9	4.43	>100	11.7	>100	6.21	23.5
	SF-295	6.96	44.5	11.5	49.2	5.05	62.4	2.16	11.1	6.11	19.8
	SF-539	12	50.2	13	51.7	10.3	51.2	1.71	5.93	10.1	22
	SNB-19	10.4	51	11	50.2	5.58	>100	11.2	48.7	9.18	21.3
	SNB-75	3.23	>100	7.59	>100	3.57	>100		91.9	5.6	25.7
	U251	10.4	57.8	11.6	58.7	6.7	55.3	1.81	6.89	5.25	18.6
Melanoma	LOX IMVI	2.47	27.4	4.12	39.3	3.23	34	1.71	5.62	2.74	9.16
	MALME-3M	12.6	54.5	16.4	60.9	10.6	>100	11.9	55.2	12.8	26.4
	M14	5.98	51.5	5.91	49.2	4.27	99.6	3.28	47.4	4.14	15.3
	MDA-MB-435	7.44	59.8	6.03	61.7	5.47	79.6	2.8	93.9	3.8	14
	SK-MEL-2	10.6	55.4	11.2	52.3	8.1	53.4	13.5	59.5	5.47	19.5
	SK-MEL-28	10.2	48.4	11.3	49.6	5.23	47.3	6.13	44.6	6.77	20.1
	SK-MEL-5	4.7	41.6	8.25	45.9	3.5	36.4	3.4	36.2	5.89	18.8
	UACC-257	12.6	61	17.4	62.9	13.3	>100	15.1	65.1	8.36	21.8

	UACC-62	6.68	46.9	8.4	47.1	4.79	51.6	8.28	48.8	5.41	18.7
Ovarian Cancer	IGROV1	11.2	50.6	15.1	63.6	10.8	>100	16.1	59.8	8.58	21.4
	OVCAR-3	8.59	48.7	11.3	54.6	5.01	59.4	6.18	98.4	3.16	11.9
	OVCAR-4	4.39	99.3	7.79	>100	4.12	>100	8.02	>100	5.92	22
	OVCAR-5	13.3	53.2	14.9	53.7	8.43	>100	12.9	55.4	10.7	22.6
	OVCAR-8	12.2	57.1	13	56	9.51	>100	11.1	>100	10.2	23.7
	NCI/ADR-RES	7.33	50.3	8.4	47.1	5.66	>100	3.38	56.3	5.49	18.4
	SK-OV-3	13	68.1	17	>100	13.9	>100	21.8	>100	11.5	27.7
Renal cancer	786-0	11.2	56.9	10.5	51	6.33	>100	2.94	51.9	7.28	20.2
	A498	11.6	49.7	12.6	51.4	10	87.8	11.3	50.3	10.5	23.1
	ACHN	4.92	43.5	6.31	44.1	3.4	>100	5.13	60.4	4.2	15.3
	CAKI-1	4.75	52.6	6.98	58	3.34	>100	5.2	>100	4.93	19.5
	RXF 393	2.94	39.9	4.98	44.9	2.59	41.7	1.45	6.74	3.09	15.1
	SN12C	8.2	46.2	11.2	48.8	3.6	44.5	5.71	63.6	3.79	15.9
	TK-10	14.6	65.5	15.2	61.9	8.09	>100	13.1	>100	11.7	30.3
	UO-31	4.6	41.9	6.77	44.7	3.78	>100	4.45	41.4	4	16.6
Prostate Cancer	PC-3	4.39	>100	3.54	89.5	3.5	>100	3.22	>100	3.74	14.1
	DU-145	12.1	53.2	14	74.8	7.54	>100	10.9	>100	9.7	21.8
Breast Cancer	MCF7	1.99	8.23	2.01	25.2	1.93	46.1	1.96	9.34	2.28	6.33
	MDA-MB-231/ATCC	4.84	45.8	6.56	48	2.73	63	4.41	83.4	4.72	17.4
	HS 578T	7.39	>100	10.7	>100	4.68	>100	8.24	>100	11.4	58.1
	BT-549	13.9	54.3	15.7	57.8	13.2	56.7	2.25	31.2	11.6	23.9
	T-47D	3.45	41.7	3.86	52.7	3.78	>100	3.48	>100	4.37	18
	MDA-MB-468	2.84	37.6	4.66	43	2.57	52.6	2	11.5	3.71	14.8

**Panel**	**Cell line**	**24f NSC: D-836366/1**		**24g NSC: D-836367/1**		**29e NSC: D-837236/1**		**29f NSC: D-837238/1**		**30g NSC: D-837685/1**	
		GI_{50}	LC_{50}	GI_{50}	LC_{50}	GI_{50}	LC_{50}	GI_{50}	LC_{50}	GI_{50}	LC_{50}
Leukemia	CCRF-CEM	3.42	>100	3.79	>100		>100	3.32	>100	25.9	>100
	HL-60(TB)	2.04	6.34	3.81	30	44.7	>100	2.35	>100	27.5	>100
	K-562	2.42	6.79	2.98	30.4	4.5	>100	1.85	>100	29.1	>100
	MOLT-4	2.5	6.49	2.9	9.61	8.54	>100	1.71	>100	17.3	>100
	RPMI-8226	2.42	6.92	3	>100	26.4	>100	2.39	>100	11.2	>100
	SR					3.41	>100	1.89	>100	18.1	>100
Non-Small Cell Lung Cancer	A549/ATCC	3.41	14.2	10	25.4	100	>100	4.78	>100	51.3	>100
	EKVX	2.95	10.9	4.2	15.7	100	>100	4.23	>100	47.2	>100
	HOP-62	8.39	23.5	13.6	27.7	100	>100	17.8	>100	50.7	>100
	HOP-92	2.49	6.51	6.15	20.7	3	>100	2.1	>100	7.48	>100
	NCI-H226	3.41	16.4	7.82	24.7	100	>100		>100	17.6	>100
	NCI-H23	4.17	15.6	11.2	24.2	100	>100	16.9	>100	37.6	>100
	NCI-H322M	4.24	17.8	11.6	24.3	100	>100	100	>100	45.4	>100
	NCI-H460	2.45	6.93	4.09	14.9	42.3	>100	2.61	>100	36.5	>100

	NCI-H522	2.81	8.9	11.2	24.7	100	>100	4.75	82.6	51.7	>100
Colon cancer	COLO 205	2.38	6.56	11.2	24.8	100	>100	3.09	>100	16.5	76.2
	HCC-2998	1.8	3.45	1.77	3.61	45.3	>100	2.26	36.4	20.2	>100
	HCT-116	1.6	3.01	1.68	3.13	12.5	>100	1.76	7.56	41.9	>100
	HCT-15	1.99	4.28	3.22	11.9	76.6	>100	3.4	88.6	30.9	>100
	HT29	1.9	3.65	1.91	3.95	29.4	>100	2.23	84.7	19.8	>100
	KM12	2	4.41	2.38	6	40.1	>100	3.05	85.4	37	>100
	SW-620	1.9	4.02	2.24	5.06	100	>100	5.59	>100	46.9	>100
CNS Cancer	SF-268	3.89	16.4	7.71	22.5	100	>100	6.14	>100	19.9	>100
	SF-295	3.4	13	11.3	23.5	100	>100	7.12	>100	21.1	>100
	SF-539	2.52	6.78	10.7	22.5	100	>100	6.1	59	22.5	>100
	SNB-19	4.58	16.8	12	24.3	100	>100	9.14	>100	30	>100
	SNB-75	2.88	16	8.41	22.7	3.24	>100	2.17	>100		
	U251	1.99	4.41	3.42	13	100	>100	4.63	67.6	24.2	>100
Melanoma	LOX IMVI	1.66	3.07	1.86	3.84	47.8	>100	1.78		17.1	64.1
	MALME-3M	3.42	12.4	14.2	27.5	100	>100	2.84	55	19.9	>100
	M14	2.6	8.28	3.39	12.9	100	>100	4.41	>100	22	>100
	MDA-MB-435	2.32	5.69	2.86	8.49	100	>100	4.59	>100	34.2	>100
	SK-MEL-2	2.77	8.31	9.45	22.3	100	>100	2.94	41.1	15.8	57.8
	SK-MEL-28	3.6	13.7	10.4	22.2	100	>100	28.1	>100	34.1	>100
	SK-MEL-5	2.91	11.1	10.4	22.2	17.2	>100	3.32	50	15.9	57.1
	UACC-257	3.61	15.2	11	25	100	>100	14.1	>100	20.4	>100
	UACC-62	3.57	14.6	6.24	19.4	100	>100	13.1	74.1	16.4	81.1
Ovarian Cancer	IGROV1	4.27	15	12.3	24.9		>100	5	100	27	>100
	OVCAR-3	1.74	3.24	2.04	5.61	100	>100	5.46	66.4	53.9	>100
	OVCAR-4	3.68	15.6	8.78	23.1	18.9	>100	3.92	>100	37	>100
	OVCAR-5	3.39	15	12	24.4	100	>100	35.2	>100	27	>100
	OVCAR-8	3.42	13.9	12.3	30.6	100	>100	5.47	>100	59.5	>100
	NCI/ADR-RES	3.25	11.6	10.7	23.7						
	SK-OV-3	8.23	21.6	13.7	27.8	100	>100	63.5	>100	69.7	>100
Renal cancer	786-0	2.56	6.3	10.6	22.7	100	>100	4	42.5	20.8	>100
	A498	6.21	19.3	13	26.2	100	>100	17	>100	24.1	>100
	ACHN	3.13	10.9	5.08	17.4	100	>100	6.05	>100	64.4	>100
	CAKI-1	3.01	12.7	6.46	19.7	3.38	>100	1.65	>100	25.9	>100
	RXF 393	2.26	7.51	3.7	16	2.46	>100	1.22	34.1	10.3	>100
	SN12C	1.7	3.36	6.36	19.9	100	>100	6.09	>100	35.7	>100
	TK-10	4.62	16.6	17.4	32	100	>100	18	>100	54.3	>100
	UO-31	2.9	11.9	6.41	19.9		>100	3.66	>100	100	>100
Prostate Cancer	PC-3	2.76	10.6	4.15	18.2	6.16	>100	3.2	>100	24.6	>100
	DU-145	3.94	14	11.2	24	100	>100	4.9	>100	72	>100
Breast Cancer	MCF7	1.8	3.46	1.7	3.56	9.9	>100	2.66	>100	18.1	>100
	MDA-MB-231/ATCC	2.64	8.39	8.38	20.8	6.73	>100	2.29	35.1	16.9	>100
	HS 578T	6.3	71.5	12.7	49.5	84.8	>100	4.77	>100	19.2	>100

	BT-549	4.87	17.1	12.7	25.6	35.5	>100	4.41	79.3	13.8	73.1
	T-47D	3.02	11.7	5.16	19.6	100	>100	19	>100	21.3	>100
	MDA-MB-468	2.55	8.09	4.53	17	17.8	>100	2.4	>100	13.7	69.5

**Panel**	**Cell line**	**31a NSC: D-836368/1**		**31b NSC: D-836369/1**		**31e NSC: D-836372/1**		**31f NSC: D-836373/1**		**31g NSC: D-836374/1**	
		GI_{50}	LC_{50}	GI_{50}	LC_{50}	GI_{50}	LC_{50}	GI_{50}	LC_{50}	GI_{50}	LC_{50}
Leukemia	CCRF-CEM	4.02	>100	2.81	>100	2.92	>100	3.99	>100	3.24	86
	HL-60(TB)	4.24	>100	2.94	20.2	2.72	13.3	3.12	>100	4.07	86
	K-562	4.33	>100	2.75		3.08	>100	2.8	>100	3.51	86
	MOLT-4	3.11	>100	2.2	7.53	2.68		2.45		2.74	86
	RPMI-8226	3.32	>100	2.94	>100	3.19	>100	5.25	>100	4	86
	SR										
Non-Small Cell Lung Cancer	A549/ATCC	>100	>100	>100	>100	>100	>100	>100	>100	55	86
	EKVX	6.65	>100	5.84	>100	5.05	>100	5.17	>100	5.52	86
	HOP-62	99.7	>100	>100	>100	>100	>100	96	>100	31.4	86
	HOP-92	2.18	9.96	2.54	21	2.53	13.2	3.09	23.3	2.99	16.9
	NCI-H226	>100	>100	>100	>100	>100	>100	>100	>100	37.3	86
	NCI-H23	>100	>100	78.8	>100	72.7	>100	54.1	>100	27.7	86
	NCI-H322M	>100	>100	>100	>100	>100	>100	>100	>100	42.5	86
	NCI-H460	6.49	>100	4.76	>100	4.49	>100	4.74	>100	5.49	86
	NCI-H522	77.4	>100	21.3	>100	16	>100	34.8	>100	17.8	44.8
Colon cancer	COLO 205	16.4	>100	11.5	>100	5.65	25.6	11.8	>100	9.73	21.8
	HCC-2998	11.7	>100	2.93	41.1	3.14	18.4	4.22	>100	4.73	25.9
	HCT-116	7.37	>100	3.81	>100	3.77	>100	4.54	>100	5.1	86
	HCT-15	>100	>100	4.95	>100	>100	>100	>100	>100	55.1	86
	HT29	5.32	>100	2.27	4.65	2.68	7.54	3.88	>100	4.88	18.3
	KM12	8.8	>100	4.56	>100	3.98	>100	4.69	>100	6.14	86
	SW-620	40.4	>100	8.04	>100	5.95	>100	8.27	>100	14.3	86
CNS Cancer	SF-268	9.85	>100	7.53	>100	5.74	>100	4.83	>100	11.2	86
	SF-295	9.49	>100	6.6	>100	6.2	>100	6.71	>100	12.9	86
	SF-539	12	63.8	3.29	78.9	3.3	>100	3.89	>100	6.22	24.7
	SNB-19	21.8	>100	13.1	>100	5.67	>100				
	SNB-75	3.58	>100	>100	>100	3.76	>100	2.53	74	2.13	86
	U251	18.4	>100	7.08	>100	5.14	>100	6.35	>100	5.52	86
Melanoma	LOX IMVI	9.96	25.7	2.09	5.11	1.77	3.55	2.62	9.13	3.14	12
	MALME-3M	7.34	>100	4.65	33.2	3.86	19.2	6.28	>100	11.3	24.2
	M14	24.4	>100	4.56	>100	3.15	11.5	3.84	25.3	7.87	86
	MDA-MB-435	15	>100	8.79	>100	5.17	>100	5.93	>100	9.13	86
	SK-MEL-2	28.5	>100	11.2	77.7	3.34	14	8.17	29	10.8	24.7
	SK-MEL-28	>100	>100	26.9	>100	8.22	>100	15.7	>100	15	86
	SK-MEL-5	5.81	20.5	4.98	20.6	3.45	14.3	3.36	14.9	10.4	21.3

	UACC-257	87.2	>100	26	>100	8.07	>100	11.4	>100	23.3	86
	UACC-62	31.5	>100	20.9	>100	6.46	>100				
Ovarian Cancer	IGROV1	5.76	>100	64.4	>100	9.96	>100	5.01	>100	11.2	86
	OVCAR-3	69.3	>100	9.93	>100	7.45	>100	8.09	>100	11.9	86
	OVCAR-4	74.6	>100		>100		>100		>100	25	86
	OVCAR-5	>100	>100	>100	>100	>100	>100	>100	>100	19.9	86
	OVCAR-8	61.4	>100		>100	>100	>100	12	>100	26.1	86
	NCI/ADR-RES	>100	>100	>100	>100	>100	>100	>100	>100	86	86
	SK-OV-3	>100	>100	>100	>100	>100	>100	>100	>100	86	86
Renal cancer	786-0	7.88	>100	7.06	>100	6.21	>100	4.77	>100	10.1	39.8
	A498		>100	>100	>100	58.4	>100	22.1	>100	22.2	77.4
	ACHN	>100	>100	>100	>100	>100	>100	>100	>100	86	86
	CAKI-1	9.73	>100	8.06	>100	8.07	>100	6.5	>100	36.8	86
	RXF 393	2.55	8.95	2.27	9.12	2.27	8.75	2.48		2.57	18.8
	SN12C	7.83	>100	>100	>100	7.37	>100				
	TK-10	>100	>100	>100	>100	>100	>100	>100	>100	86	86
	UO-31	>100	>100	>100	>100	>100	>100	>100	>100	86	86
Prostate Cancer	PC-3	3.84	>100	3.33	>100	3.44	>100	5.04	>100	4.37	86
	DU-145	>100	>100	>100	>100	>100	>100	52	>100	38.5	86
Breast Cancer	MCF7	3.85	85.5	3.08	>100	2.89	>100	3.16	43.7	2.87	21
	MDA-MB-231/ATCC	6.16	67.6	3.86	>100	3.73	65.7				
	HS 578T	6.43	>100	19.4	>100	7.15	>100	5.7	>100	9.03	86
	BT-549	8.82	>100	5.46	59.4	3.96	28.7	3.26	18.2	11.8	32.6
	T-47D	5.12	>100	8.75	>100	7.93	>100	4.86	>100	12	86
	MDA-MB-468	3.37	13.7	2.72	8.52	1.92	4.92	2.15	6.24	2.67	11.7

**Panel**	**Cell line**	**33a NSC: D-830631/1**		**33b NSC: D-830632/1**		**33e NSC: D-830635/1**		**33f NSC: D-832368/1**		**33g NSC: D-832369/1**	
		GI_{50}	LC_{50}	GI_{50}	LC_{50}	GI_{50}	LC_{50}	GI_{50}	LC_{50}	GI_{50}	LC_{50}
Leukemia	CCRF-CEM	0.95	>100	1.67	>100	1.14	>100	0.754	>100	1.23	>100
	HL-60(TB)	0.32	>100	1.71	>100	1.15	>100	0.863	>100	1.28	>100
	K-562	1.01	>100	1.34	24.90	0.71	>100	0.489	>100	0.642	>100
	MOLT-4	0.01	>100	1.38	>100	0.79	>100	0.532	>100	0.888	>100
	RPMI-8226	1.38	>100	1.83	>100	1.44		1.01	>100	1.51	>100
	SR	0.55		1.68	>100	0.91	>100	0.672	>100	1.46	>100
Non-Small Cell Lung Cancer	A549/ATCC	2.84	>100	1.74	6.80	4.12	>100	3.12	>100	1.73	7.08
	EKVX	1.64	6.12	1.55	5.71	2.01	28.7	1.66	24.00	1.52	5.75
	HOP-62	1.74	6.07	1.68	6.08	2.83	34.5	1.77	11.70	1.63	6.14
	HOP-92	1.42	6.74	1.3	6.30	1.29	7.54	1.25	10.20	1.48	7.94
	NCI-H226	11.50	85	16.7	88.20	6.47	>100	2.49	37.90	1.84	8.31
	NCI-H23										
	NCI-H322M	1.63	5.67	1.59	5.42	2.02	16.9	2.43	33.50	1.66	5.63

	NCI-H460	1.85	6.51	1.8	6.41	1.72	6.42	1.71	7.73	1.96	8.23
	NCI-H522	1.79	6.77	1.72	7.03	1.56	6.41	1.43	6.16	1.34	5.54
Colon cancer	COLO 205	1.79	6.09	1.67	6.09	1.51	8.55	1.49	5.80	1.33	5.25
	HCC-2998	1.68	5.72	1.55	5.56	1.44	5.36	1.61	5.78	1.31	5.25
	HCT-116	1.51	5.33	1.62	5.45	1.40	5.19	1.2	5.23	1.05	5.54
	HCT-15	1.38	5.87	1.48	5.87	1.29	6.09	1.03	7.87	1.15	5.83
	HT29	1.77	6.28	1.78	7.14	1.70	7.96	1.42	7.01	1.36	6.54
	KM12	1.90	6.61	1.97	7.54	3.18	>100	1.68	8.22	1.37	6.05
	SW-620	1.87	6.48	1.79	6.12	1.62	6.72	1.6	6.68	1.6	7.04
CNS Cancer	SF-268	1.87		1.81	7.35	4.14	>100	2.85	76.00	1.66	7.27
	SF-295	1.57	5.63	1.63	5.63	1.73	7.76	1.96	7.15	1.79	6.14
	SF-539	1.60	5.85	1.57	5.63	1.68	6.56	1.56	6.36	1.56	5.68
	SNB-19	1.58	5.64	1.57	5.75	1.99	21.4	1.76		1.51	5.76
	SNB-75	1.56	6.29	1.62	6.73	1.59	9.94	3.35	45.40	1.7	9.06
	U251	1.53	5.84	1.6	5.65	1.47	5.81	1.37	5.43	1.24	5.16
Melanoma	LOX IMVI	1.54	5.66	1.65	5.69	1.45	5.63	1.52		1.77	
	MALME-3M	1.76		1.7	6.69	1.75		1.66		1.74	
	M14	1.84	6.1	1.84	5.99	1.90	10.5	1.66	6.58	1.64	5.72
	MDA-MB-435	1.74	5.78	1.73	5.68	1.63	6.2	1.7	7.42	1.4	5.3
	SK-MEL-2	1.38	5.6	1.81	6.40	1.45	5.53	1.67	5.75	1.67	5.64
	SK-MEL-28	1.73	5.64	1.77	5.65	1.73	5.61	1.7	5.93	1.53	5.37
	SK-MEL-5	1.42	5.21	1.56	5.43	1.48	5.3	1.46	5.30	1.55	5.37
	UACC-257	1.90	6.43	1.84	6.54	1.91	7.32	1.85	6.69	1.75	5.82
	UACC-62	1.84	6	1.82	6.33	2.03	9.06	1.96		1.68	
Ovarian Cancer	IGROV1	1.56	6.29	1.65	5.98	1.71	7.46	1.7		1.37	
	OVCAR-3							1.58	6.82	1.32	6.57
	OVCAR-4	1.72	8.19	1.85	7.14	1.96	32.7	1.93	>100	2.23	>100
	OVCAR-5	1.94	7.25	1.62	5.59	4.48	74	5.1	>100	1.6	5.64
	OVCAR-8	1.46	6.59	1.8	7.55	1.57	6.81	1.16	6.81	1.5	5.75
	NCI/ADR-RES	4.33	>100	1.73	6.97	4.24	>100	7.73	>100	1.95	
	SK-OV-3	17.70	57.9	2.69	25.60	14.80	58.3	12.9	50.50	1.83	6.52
Renal cancer	786-0	1.63	5.8	1.64	5.70	1.55	5.96	1.36	7.12	1.39	6.09
	A498	1.54	5.96	3.85	42.00	5.90	44.5	3.72	39.10	1.56	5.4
	ACHN	1.95	9.76	1.72	5.56	4.69	>100	3.22	>100	1.5	5.31
	CAKI-1	2.30	24.8	1.64	5.73	5.36	>100	6.14	>100	1.45	5.61
	RXF 393	1.63		1.45		1.54	5.73	1.17	5.90	1.14	5.48
	SN12C	1.74	7.2	1.79	6.39	1.95		2.08	>100	1.76	6.35
	TK-10	2.09	7.16	1.9	6.02	10.60	60.4	4.61	45.50	1.78	5.63
	UO-31	1.87	9.83	1.64	5.51	3.10	>100	3.07	>100	1.43	5.3
Prostate Cancer	PC-3	1.59		1.74	7.43	1.93	>100	1.72	>100	1.54	
	DU-145	1.80	6.34	1.76	5.77	2.22	22.8	1.75	7.00	1.51	5.33
Breast Cancer	MCF7	1.51	6.1	1.46	6.22	1.20	5.47	0.956	6.00	1.26	
	MDA-MB-231/ATCC	1.75	6.94	1.67	6.37	1.75	8.82	1.78		1.88	

	HS 578T	1.98	>100	1.79	>100	2.22	>100	2.04	>100	2.55	>100
	BT-549	1.77	5.81	1.73	5.69	2.25	23.5	1.56	6.47	1.57	6.34
	T-47D	1.52	7.29	1.67	8.01	1.71	54.6	1.27	8.09	1.55	8.75
	MDA-MB-468	1.51	5.93	1.65	6.09	1.39	5.6	1.48	6.70	1.58	6.53

References

1. Osman, S.M.; Alasmary, F.A.; Kenawy, E.R.; Aly, E.S.A.; Khattab, S.N.; El-Faham, A. Synthesis, Characterization and Comparative Thermal Degradation Kinetics of s-Triazine Based Polymers. *J. Polym. Res.* **2021**, *28*, 304. https://doi.org/10.1007/s10965-021-02667-y.
2. Venkata Krishna Reddy, M.; Vasu Govardhana Reddy, P.; Suresh Reddy, C. PEPPSI-SONO-SP2: A New Highly Efficient Ligand-Free Catalyst System for the Synthesis of Tri-Substituted Triazine Derivatives: Via Suzuki-Miyaura and Sonogashira Coupling Reactions under a Green Approach. *New J. Chem.* **2016**, *40*, 5135–5142. https://doi.org/10.1039/c5nj03299g.
3. Anamika, S.; Ghabbour, H.; Khan, S.T.; De la Torre, B.G.; Albericio, F.; El-Faham, A. Novel Pyrazolyl-s-Triazine Derivatives, Molecular Structure Ans Antimicrobial Activity. *J. Mol. Struct.* **2017**, *1145*, 244–253. https://doi.org/10.1016/j.molstruc.2017.05.040.
4. Padilla-Salinas, R.; Sun, L.; Anderson, R.; Yang, X.; Zhang, S.; Chen, Z.J.; Yin, H. Discovery of Small-Molecule Cyclic GMP-AMP Synthase Inhibitors. *J. Org. Chem.* **2020**, *85*, 1579–1600. https://doi.org/10.1021/acs.joc.9b02666.
5. Singla, P.; Luxami, V.; Paul, K. Triazine-Benzimidazole Conjugates: Synthesis, Spectroscopic and Molecular Modelling Studies for Interaction with Calf Thymus DNA. *RSC Adv.* **2016**, *6*, 14741–14750. https://doi.org/10.1039/c5ra24001h.
6. Adhikari, N.; Choudhury, A.A.K.; Shakya, A.; Ghosh, S.K.; Patgiri, S.J.; Singh, U.P.; Bhat, H.R. Design and Development of Novel N-(4-Aminobenzoyl)- l-Glutamic Acid Conjugated 1,3,5-Triazine Derivatives as Pf-DHFR Inhibitor: An in-Silico and in-Vitro Study. *J. Biochem. Mol. Toxicol.* **2023**, *37*, e23290. https://doi.org/10.1002/jbt.23290.
7. Rosenau, T.; Renfrew, A.H.M.; Adelwöhrer, C.; Potthast, A.; Kosma, P. Cellulosics Modified with Slow-Release Reagents. Part I. Synthesis of Triazine-Anchored Reagents for Slow Release of Active Substances from Cellulosic Materials. *Polymer* **2005**, *46*, 1453–1458. https://doi.org/10.1016/j.polymer.2004.12.027.
